# Differentially Expressed Long Noncoding RNAs Involved in FUBP1 Promoting Hepatocellular Carcinoma Cells Proliferation

**DOI:** 10.1155/2021/6664519

**Published:** 2021-04-14

**Authors:** Xianpeng Li, Huaixi Yu, Feng Xu, Yifeng Wu, Jifang Sheng

**Affiliations:** ^1^State Key Laboratory for Diagnosis and Treatment of Infectious Diseases, Collaborative Innovation Center for Diagnosis and Treatment of Infectious Disease, The First Affiliated Hospital, Zhejiang University School of Medicine, Hangzhou, 310003 Zhejiang, China; ^2^Department of Infectious Disease, The Affiliated People's Hospital of Ningbo University, Ningbo, 315000 Zhejiang, China; ^3^Department of Orthopedics, The Affiliated Huai'an Hospital of Xuzhou Medical University, The Second People's Hospital of Huai'an, Huai'an, 223002 Jiangsu, China; ^4^Department of Gastroenterology, Ningbo Medical Center Lihuili Hospital, Ningbo, 315000 Zhejiang, China

## Abstract

**Background:**

Far upstream element-binding protein 1 (FUBP1) is reported to be involved in cancer development by regulating the transcription of c-myc gene through binding to far upstream element. Highly expressed FUBP1 was negatively correlated with survival rate of patients with hepatocellular carcinoma (HCC) and could promote the proliferation of HCC cells. However, the downstream mechanism of FUBP1 has not yet been clearly explained. This study is aimed at identifying the expression profiles of long noncoding RNA (lncRNA) in HCC cells in response to FUBP1 overexpression and at investigating the possible lncRNAs that participated in cell proliferation process regulated by FUBP1.

**Methods:**

The overexpression of FUBP1 was mediated by lentiviral infection on 3 different types of HCC cell lines (MHCC97-H, MHCC97-L, and Huh-7). The expression of target genes was detected by quantitative reverse transcription-PCR (RT-PCR) and western blotting assays. Microarray and quantitative RT-PCR were applied to screen the differentially expressed lncRNAs in HCC cells after FUBP1 overexpression. The Cell Counting Kit-8 assay was used to confirm the growth vitality of HCC cells.

**Results:**

The growth vitality of HCC cells was significantly increased after lentivirus infection. A total of 12 lncRNAs had the same expression trend in the 3 HCC cell lines in response to FUBP1 overexpression, including 3 upregulated lncRNAs and 9 downregulated lncRNAs. Coexpression analysis of dysregulated lncRNAs-mRNAs network showed that lnc-LYZ-2 was the lncRNA most relevant to FUBP1. Inhibition of lnc-LYZ-2 could significantly relieve the proproliferation effect of FUBP1 on HCC cells, suggesting that lnc-LYZ-2 was partially involved in proproliferation regulation of FUBP1.

**Conclusions:**

Our results indicated that FUBP1 induced the abnormal expression of lncRNAs and the FUBP1-lncRNAs coexpression network in HCC cells, which could provide theoretical and experimental basis for FUBP1-lncRNAs network involved in HCC development.

## 1. Introduction

As one of the most common malignant tumors, hepatocellular carcinoma (HCC) is a leading cause of cancer-related death worldwide [1]. Despite much improvement on surgical treatment, chemotherapy, and interventional therapy, the prognosis of HCC was still dissatisfied [2, 3]. Therefore, to acquire an effective treatment for HCC, it is highly meaningful to explore the detailed mechanisms that govern the initiation, development, and progression of this disease.

Far upstream element-binding protein 1 (FUBP1) is a kind of DNA-binding protein, which regulates c-myc gene transcription through binding to the far upstream element (FUSE) [4]. It can affect cell proliferation, differentiation, and apoptosis, leading to the development of various tumors [5, 6]. It is reported that highly expressed FUBP1 is found in 70% of HCC patients and significantly negatively correlated with survival [7]. In addition, in vitro studies have confirmed that FUBP1 can induce HCC cell proliferation [8]. Thus, FUBP1 might be a potential target for HCC treatment, but its downstream mechanism has not yet been elucidated.

Long noncoding RNA (lncRNA) is an important regulatory factor in physiological and pathological process of diverse diseases. It is a noncoding RNA molecule with more than 200 nucleotides that regulates gene expression at multiple expression stages [9]. lncRNA was previously considered “transcriptional noise” of genes and had no biological functions. However, with the application and development of high-throughput sequencing technology, a large number of lncRNAs were found to regulate cellular activities, such as cell proliferation, differentiation, apoptosis, and cell cycle [10]. Although the detailed mechanism has not been elucidated, evidence shows that lncRNA plays a complex and precise regulatory role in the development of organism and disease.

In the present study, we detected the abnormally expressed lncRNAs in 3 HCC cell lines in response to FUBP1 overexpression and explored the potential lncRNAs involved in HCC cells proliferation regulated by FUBP1. The results would provide theoretical and experimental basis for FUBP1-lncRNAs network involved in HCC development.

## 2. Material and Methods

### 2.1. HCC Cell Lines Culture

MHCC97-H and MHCC97-L cell lines were gifted by the Shanghai Liver Cancer Institute [11]. HuH-7 cell line was acquired from China Academy of Science. These HCC cells were routinely cultured in DMEM supplemented with 10% fetal bovine serum (10%) and penicillin-streptomycin (1%) in a cell culture incubator (5% CO_2_ and 37°C).

### 2.2. Lentivirus Construction and Infection

The lentivirus for FUBP1 overexpression (Lv-FUBP1) was purchased from the Shanghai Genechem Company. The lentivirus overexpressing GFP (Lv-GFP) was used as a negative control. Lentivirus infection was performed in HCC cells following the manufacturer's instruction. Puromycin (2 *μ*g/mL) was applied to remove the uninfected cells.

### 2.3. Small Interfering RNA (siRNA) Synthesis and Transfection

The siRNAs targeting lnc-LYZ-2 (si-lnc-LYZ-2) and scrambled control (si-NC) were designed and synthesized by the Guangzhou RiboBio Company. The transient transfection was performed by using the riboFECT CP Transfection Kit.

### 2.4. Quantitative Reverse Transcription-PCR (RT-PCR)

Total RNAs were isolated from HCC cells by the mirVana™ total RNA Isolation Kit (Thermo Fisher Scientific). After quantification by a spectrophotometer, equal amount of RNA samples (200 ng) was used as template to be reverse-transcribed to complementary DNA (cDNA). Terra™ qPCR Direct TB Green™ Premix (Takara) was used for quantitative RT-PCR detection. Rn18s and *β*-actin were used as internal controls for lncRNAs and mRNA detection, respectively.

### 2.5. Western Blotting Assay

The methods for protein purification and quantification were described as reported [12]. Protein electrophoresis separation was performed by 10% SDS-PAGE. The nonfat milk-blocked PVDF membranes were incubated with the primary antibodies against FUBP1 (1: 1000 dilution; Abcam) or *β*-actin (1: 2000 dilution, Boster) overnight at 4°C, then incubated with the secondary antibodies for 2 hours at room temperature. Visualization was achieved by ECL Plus Western Blotting Substrate (Thermo Scientific) on ChemiDoc MP system (Bio-Rad). *β*-Actin was the internal control.

### 2.6. Microarray Assay

lncRNA microarrays were accomplished by using the Affymetrix Human OElncRNA array (OEbiotech). After RNA quality assessment on bioanalyzer system (Agilent Technologies), RNA samples were hybridized onto the microarray after transcription, fragmentation, and Cyanine-3-CTP labeling. The signal values were scanned by Affymetrix Scanner 3000 (Affymetrix).

### 2.7. Data Analysis

The raw data was extracted from microarray by the Affymetrix GeneChip Command Console (Affymetrix) software. After robust multiarray average (RMA) normalization, the gene expression analysis was performed by the GeneSpring software (Agilent Technologies), and alternative splice analysis was performed by the Transcriptome Analysis Console (Affymetrix). The threshold of fold change ≥ 2.0 and *P* value ≤ 0.05 was selected for dysregulated genes and lncRNAs.

### 2.8. Coexpression Analysis

The coexpression network of lncRNA-mRNA was established according to the interactions between dysregulated lncRNAs and mRNAs. Pearson's correlation coefficient was calculated as a reference for coexpression analysis. The coexpression network was accomplished by Cytoscape with parameters of correlation > 0.7 and *P* value < 0.05.

### 2.9. Cell Vitality Assay

The growth vitality of HCC cells was detected by the Cell Counting Kit-8 (CCK-8, Beyotime). Briefly, HCC cells were seeded in a 96-well plate and incubated overnight (at 37°C, 5% CO_2_), followed by lentivirus infection or siRNA transfection for 48 hours. After adding 10 *μ*L CCK-8 solution, HCC cells were incubated at room temperature for 2 hours. The relative growth vitality was calculated according to the absorbance value at 450 nm on a microplate reader (BioTek).

### 2.10. Statistical Analysis

The data was presented as mean ± standard deviation and analyzed by the SPSS software (version 22.0) for statistical analysis. The comparison between two-group data was analyzed by Student' *t* test, and comparison among multigroup data was analyzed by one-way ANOVA. A *P* value less than 0.05 was defined as statistically significant difference.

## 3. Results

### 3.1. Overexpression of FUBP1 Promoted the Proliferation of HCC Cells

**Figure 1:**
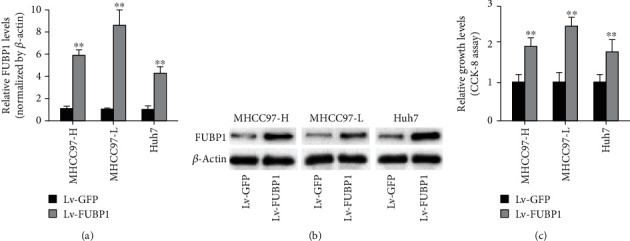
FUBP1 Overexpression promoted HCC cells proliferation. The overexpression of FUBP1 was mediated by lentivirus on 3 types of HCC cell lines (MHCC97-H, MHCC97-L, and Huh-7). (a, b) Quantitative RT-PCR and western blotting assays were applied to detect the increased expression of FUBP1 mRNA and protein in HCC cells. ∗∗*P* < 0.01 vs. the Lv-GFP group. (c) The proliferation levels of HCC cells were detected by the CCK-8 assay. FUBP1 overexpression promoted the growth vitality of HCC cells. ∗∗*P* < 0.01 vs. the Lv-GFP group.

To explore the function of FUBP1 on proliferation of HCC cells, the overexpression of FUBP1 on HCC cells was constructed by lentiviral infection. Quantitative RT-PCR and western blotting results demonstrated that the mRNA and protein levels of FUBP1 were notably increased in the 3 HCC cell lines after Lv-FUBP1 infection (Figures 1(a) and 1(b)). Compared with the Lv-GFP group, the growth vitality of FUBP1-overexpressing HCC cells was notably increased (Figure 1(c)). The results proved that overexpression of FUBP1 could promote proliferation of HCC cells.

### 3.2. Detection of the Dysregulated lncRNAs in FUBP1-Overexpressed HCC Cells

**Figure 2:**
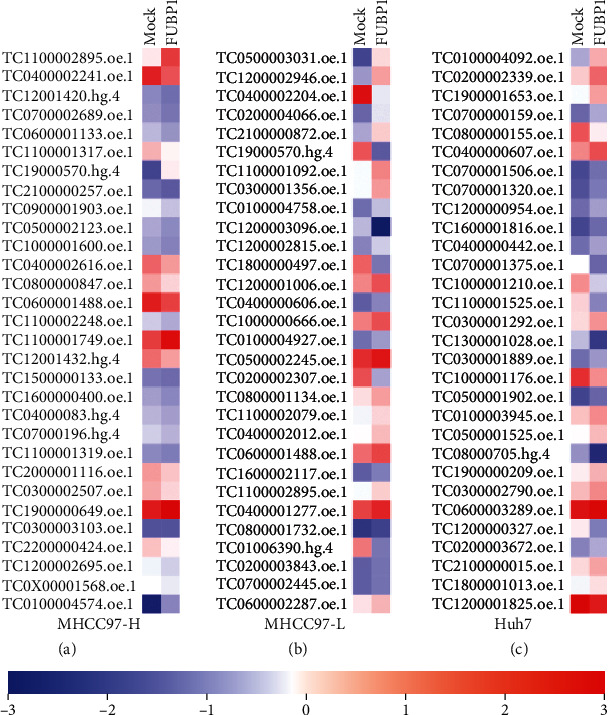
Detection of the dysregulated lncRNAs in HCC cells overexpressing FUBP1. The dysregulated lncRNAs were filtered out by microarray. The top 30 differentially expressed lncRNAs in MHCC97-H (a), MHCC97-L (b), and HuH-7 (c) cell lines were presented as heat map. The probe names of lncRNAs were listed on the *Y*-axis.

To detect the dysregulated lncRNAs involved in the process of proproliferation on HCC cells mediated by FUBP1, the microarray was applied on 3 HCC cell lines with and without FUBP1 overexpression. There were 544, 1090, and 736 differentially expressed lncRNAs in Lv-FUBP1-infected MHCC97-H, MHCC97-L, and Huh7 HCC cell lines, respectively (Supplementary 1–3). The top 30 differentially expressed lncRNAs in each HCC cell line are listed in Figure 2. Unexpectedly, most of the dysregulated lncRNAs were different among the 3 HCC cell lines. A total of 12 lncRNAs had the same expression trend in response to FUBP1 overexpression, including 3 upregulated lncRNAs and 9 downregulated lncRNAs (Table 1).

### 3.3. Validation of Microarray Result by Quantitative RT-PCR

**Figure 3:**
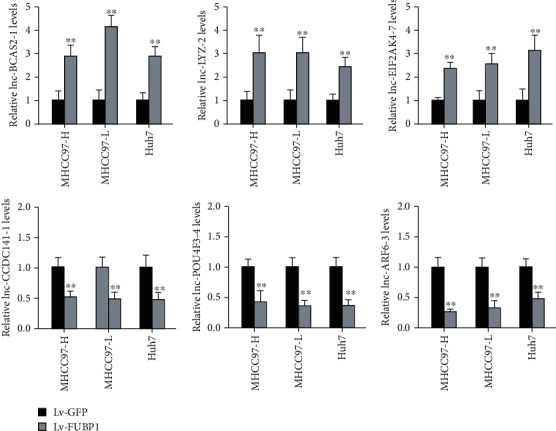
Validation of microarray result by quantitative RT-PCR. Six differentially expressed lncRNAs were detected in 3 HCC cell lines with or without FUBP1 overexpression. ∗∗*P* < 0.01 vs. the Lv-GFP group.

To confirm the lncRNAs profile obtained from microarray, 6 differentially expressed lncRNAs were detected by quantitative RT-PCR in 3 HCC cell lines with or without FUBP1 overexpression. The expression of lnc-BCAS2-1, lnc-LYZ-2, and lnc-EIF2AK4-7 was found significantly increased in FUBP1-overexpressed HCC cells, while the expression of lnc-CCDC141-1, lnc-POU4F3-4, and lnc-ARF6-3 was significantly decreased (Figure 3). The consistent expression pattern between lncRNA microarray and quantitative RT-PCR assays indicated the reliability of analysis result.

### 3.4. Coexpression Analysis of the lncRNAs-mRNAs Network

**Figure 4:**
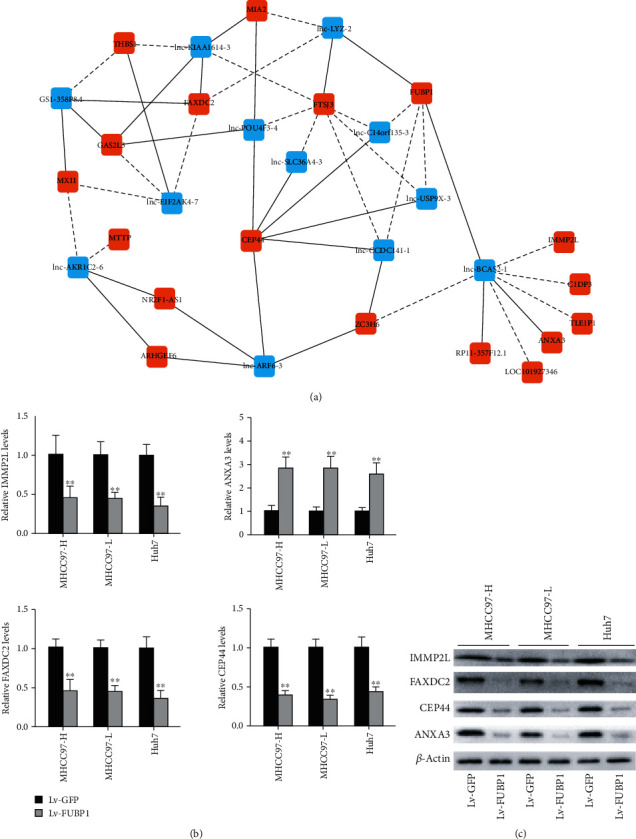
Coexpression analysis of lncRNAs-mRNAs network. (a) There were 12 lncRNAs and 14 mRNAs constituted the coexpression network, including 23 positive interactions and 20 negative interactions between the dysregulated lncRNAs and mRNAs. (b) Confirmation of mRNA expression change in HCC cells in response to FUBP1 overexpression. Quantitative RT-PCR assay was used for result validation on the selected 4 mRNAs. ∗∗*P* < 0.01 vs. the Lv-GFP group. (c) Confirmation of protein expression change in HCC cells in response to FUBP1 overexpression. Western blot assay was used for result validation.

To investigate the correlation between the dysregulated lncRNAs and mRNAs in response to FUBP1 overexpression, the lncRNAs-mRNA coexpression analysis was performed. There were 12 lncRNAs and 14 mRNAs in the coexpression network, including 23 positive interactions and 20 negative interactions between the dysregulated lncRNAs and mRNAs (Figure 4(a)). Among them, 2 upregulated lncRNAs (lnc-LYZ-2 and lnc-BCAS2-1) positively interacted with FUBP1, while 3 downregulated lncRNAs (lnc-USP9X-3, lnc-CCDC141-1, and lnc-C14orf135-3) negatively interacted with FUBP1. Quantitative RT-PCR verification on the 4 mRNAs was also performed in 3 HCC cell lines. The results showed that the mRNA expression of IMMP2L, CEP44, and FAXDC2 decreased, while the mRNA expression of ANXA3 increased in HCC cells in response to FUBP1 overexpression (Figure 4(b)).

### 3.5. lnc-LYZ-2 Was Involved in HCC Cells Proliferation Promoted by FUBP1

**Figure 5:**
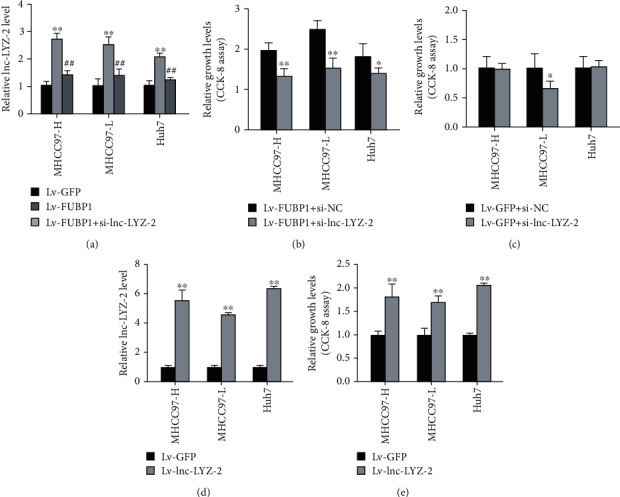
Inhibition of lnc-LYZ-2 alleviate progrowth effect of FUBP1 on HCC cells. (a) Quantitative RT-PCR assay proved that lnc-LYZ-2 expression was obviously increased in HCC cells infected byLv-FUBP1 but decreased after of lnc-LYZ-2 inhibition. ∗∗*P* < 0.01 vs. the Lv-GFP group and ^##^*P* < 0.01 vs. the Lv-FUBP1 group. (b) CCK-8 assay showed the growth vitality of HCC cells transfected with si-lnc-LYZ-2 was significantly reduced on FUBP1-overexpression condition. ∗∗*P* < 0.01 vs. the Lv-FUBP1+si-NC group. (c) CCK-8 assay showed that inhibition of lnc-LYZ-2 decreased the growth vitality of MHCC97-L cell lines on normal condition but had no effect on MHCC97-H and Huh7 HCC cell lines. ∗*P* < 0.05 vs. the si-NC group. (d) Quantitative RT-PCR assay proved that lnc-LYZ-2 expression was obviously increased in HCC cells infected by Lv-lnc-LYZ-2. ∗∗*P* < 0.01 vs. the Lv-GFP group. (e) CCK-8 assay showed that overexpression of lnc-LYZ-2 increased the growth vitality of HCC cell lines. ∗∗*P* < 0.01 vs. the Lv-GFP group.

According to the Pearson test result on lncRNAs-mRNA coexpression network analysis, lnc-LYZ-2 was the most relevant lncRNA in response to FUBP1 overexpression (*r* = 0.956, *P* < 0.01, Supplementary 4). Therefore, we further explored the role of lnc-LYZ-2 on HCC cells proliferation. Quantitative RT-PCR proved that the lnc-LYZ-2 expression level obviously increased in FUBP1-overexpressed HCC cells but decreased after transfection of siRNA (Figure 5(a)). CCK-8 assay confirmed that lnc-LYZ-2 inhibition could alleviate the progrowth effect of FUBP1 on HCC cells (Figure 5(b)). However, inhibition of lnc-LYZ-2 had no stable antiproliferation effect on HCC cells without FUBP1 overexpression, although it decreased the growth vitality of MHCC97-L cells (Figure 5(c)). The results suggested that lnc-LYZ-2 was partially involved in proproliferation regulation of FUBP1.

## 4. Discussion

In the present study, the differentially expressed lncRNAs in FUBP1-overexpressed HCC cells were identified by microarray and quantitative RT-PCR. The FUBP1-lncRNAs coexpression network was determined by bioinformatics algorithms. lnc-LYZ-2 was proved to be involved in proproliferation regulation of FUBP1 on HCC cells. These findings indicated that FUBP1 induced the disorder expression of lncRNAs and the FUBP1-lncRNAs coexpression network would be closely related to HCC development.

FUBP1 is a DNA-binding protein regulating c-myc gene transcription through interacting with FUSE, which affects cell growth, proliferation, differentiation, and apoptosis, and leads to the development of various tumors [6]. From the molecular structure analysis, FUBP1 is composed of 3 highly conserved domains that are joined by a variable junction region that regulates c-myc through the binding of the KH domain to the ligand site [13]. Overexpression of FUBP1 resulted in differentially expressed lncRNAs in HCC cells, which might also be closely related to the transcriptional regulation of c-myc.

It was found that FUBP1 is overexpressed in both fully differentiated and poorly differentiated liver cancer cells. Western blotting assay confirmed that c-myc expression was also increased in HCC tissues, suggesting that upregulated FUBP1 was one of the reasons for the increase of c-myc expression during the formation of HCC [14, 15]. Previous evidences have proved that FUBP1 is closely related to prognosis. FUBP1 level was found correlated with pathological stage and survival in glioma patients [16]. The cumulative survival rate of HCC patients with highly expressed FUBP1/2 was significantly worse [8]. Our results also suggested that the effect of FUBP1 on HCC development might also be achieved by differentially expressed lncRNAs.

The relationship between lncRNA and HCC development is one of the hotspots in research of HCC pathogenesis. The abnormal expression of lncRNAs in HCC cells was closely related to autophagy. Overexpression of lncRNA HULC significantly reduced the autophagy activity, thus participating in the pathogenesis of HCC [17]. lncRNA MALAT1 participated in the regulation of ZEB1 expression through binding with miR-143-3p in HCC progression [18]. Yuan et al. found that the shear factor MBNL3 increased the expression of PXN by regulating the variable shear of lncRNA-PXN-AS1, thus promoting the pathogenesis of HCC [19]. In this study, the expression of 12 lncRNAs was changed by the same trend in 3 types of HCC cell lines in response to FUBP1 overexpression. In-depth functional study of these lncRNAs would be of great significance for HCC diagnosis and therapy.

Recent studies have reported the function of lncRNA on X chromosome silencing, genomic imprinting, chromatin modification, transcriptional activation and interference, nuclear transport, and other processes [20]. lncRNA plays an important role in HCC pathological process, but its regulatory mechanism has not been elucidated. LincGET and FUBP1 complex could promote the cis regulatory activity of long terminal repeats [21]. The lncRNA PVT1 expression level was significantly correlated with the FUBP1 level, suggesting that it might be regulated by the transcription of FUBP1/c-myc [22]. We found that the increased expression of lnc-LYZ-2 was induced by FUBP1. Inhibition of lnc-LYZ-2 could partially neutralize the progrowth effect of FUBP1 on HCC cells. It suggested that lnc-LYZ-2 might be involved in the network of FUBP1-regulating HCC development.

There was an important limitation in the present study: the detailed mechanism of lnc-LYZ-2 and FUBP1. Recent reports had investigated the regulation mechanism of lncRNA on FUBP1 function or expression. lncRNA NORAD could attenuate FUBP1 nuclear localization and thus impaired the occupancies of FUBP1 on its target gene promoters [23]. lncRNA small nucleolar RNA host gene 6 (SNHG6) could upregulate FUBP1 expression by sponging microRNA-26a-5p [24]. The nuclear-enriched lncRNA SNHG1 could antagonize the binding of FBP-interacting repressor to FUBP1 by directly interacting with central domain of FUBP1, thereby coordinating the expression of the oncogene MYC [25]. Therefore, whether or how lnc-LYZ-2 regulates FUBP1 expression or function should also be investigated in future.

## 5. Conclusions

In summary, the present work identified the abnormally expressed lncRNAs in FUBP1-overexpressing HCC cells. The coexpression network constituted by these dysregulated lncRNAs and mRNAs might be closely related to HCC development and would be potential targets for diagnosis and treatment. Further exploration on FUBP1-lncRNAs regulatory mechanism would provide important clues for the development of HCC targeted drugs.

## Figures and Tables

**Table 1:** Differentially expressed lncRNAs in FUBP1-overexpressing hepatocellular carcinoma cells.

Gene symbol	Regulation	Fold change (abs)			*P*
		MHCC97-H	MHCC97-L	Huh7	
lnc-KIAA1614-3	Down	2.01	2.43	1.97	0.008
lnc-BCAS2-1	Up	2.55	2.06	2.17	0.006
lnc-CCDC141-1	Down	1.75	2.71	1.84	0.035
lnc-POU4F3-4	Down	2.04	2.62	1.69	0.028
lnc-PDK3-1	Down	1.56	2.00	2.62	0.044
lnc-USP9X-3	Down	1.99	1.88	2.53	0.015
lnc-AKR1C2-6	Down	2.39	1.95	1.72	0.018
lnc-SLC36A4-3	Down	1.71	1.89	2.89	0.044
lnc-LYZ-2	Up	2.10	2.62	1.99	0.011
lnc-ARF6-3	Down	2.00	2.53	3.12	0.019
lnc-C14orf135-3	Down	2.97	1.83	2.36	0.026
lnc-EIF2AK4-7	Up	2.35	2.97	2.48	0.006

## Data Availability

The data that support the findings of this study are available from the corresponding author upon reasonable request.

## Supplementary Materials

Supplementary 1 Differentially expressed long noncoding RNAs in MHCC97-H cells with or without FUBP1 overexpression.

Supplementary 2 Differentially expressed long noncoding RNAs in MHCC97-L cells with or without FUBP1 overexpression.

Supplementary 3 Differentially expressed long noncoding RNAs in Huh-7 cells with or without FUBP1 overexpression.

Supplementary 4 Pearson's test result on lncRNAs-mRNA coexpression network analysis.
