# Supplementary material for: Differentially Expressed Long Noncoding RNAs Involved in FUBP1 Promoting Hepatocellular Carcinoma Cells Proliferation
Source: Biomed Res Int. 2021 Apr 14;2021:6664519. doi: 10.1155/2021/6664519 (PMC8063849; doi:10.1155/2021/6664519)
Supplement: Supplementary 1 — Differentially expressed long noncoding RNAs in MHCC97-H cells with or without FUBP1 overexpression. [file 6664519.f1.pdf]

Probe Set ID	FC ([97H	Log FC ([9	FC (abs) ([9	Regulatior	97H-FUE	97H-mocl	GeneSymbo	IncipediaID	NonCodeID	Chr	start	stop	strand
TC0100000144.	-2.01	-1.01	2.01	down	3.41	4.42	lnc-VAMP3-	lnc-VAMP3-	NONHSAT0007	chr1	7785523	7788570	+
TC0100000145.	-2.21	-1.14	2.21	down	5.71	6.86	lnc-VAMP3-	lnc-VAMP3-	NONHSAT0007	chr1	7803051	7810347	+
TC0100000514.	-2.24	-1.16	2.24	down	10.55	11.72	lnc-PTPRU-	lnc-PTPRU-	NONHSAT0020	chr1	29058604	29097849	+
TC0100000714.	-2.18	-1.12	2.18	down	3.40	4.53	lnc-DEM1-2	lnc-DEM1-2	---	chr1	40542029	40542308	+
TC0100001044.	2.24	1.16	2.24	up	5.03	3.87	lnc-RAVER2	lnc-RAVER2	NONHSAT0037	chr1	64664200	64666140	+
TC0100001282.	2.10	1.07	2.10	up	4.01	2.94	RP11-57H12	lnc-RWDD3-	NONHSAT0046	chr1	95120147	95138554	+
TC0100001427.	-2.08	-1.06	2.08	down	3.49	4.55	lnc-GSTM4-	lnc-GSTM4-	NONHSAT0050	chr1	109651370	109652099	+
TC0100002076.	2.13	1.09	2.13	up	4.85	3.75	lnc-DARS2-	lnc-DARS2-	NONHSAT0076	chr1	173862152	173863941	+
TC0100002141.	-2.01	-1.01	2.01	down	5.87	6.88	lnc-KIAA161	lnc-KIAA161	NONHSAT0082	chr1	180887572	180888448	+
TC0100002170.	2.00	1.00	2.00	up	4.20	3.20	lnc-NPL-4	lnc-NPL-4:1	NONHSAT0083	chr1	182733792	182734779	+
TC0100002177.	-2.22	-1.15	2.22	down	4.51	5.66	lnc-DHX9-4	lnc-DHX9-4	---	chr1	183024754	183027621	+
TC0100002210.	-2.67	-1.42	2.67	down	5.05	6.46	lnc-PRG4-3	lnc-PRG4-3:	NONHSAT0084	chr1	186153826	186172118	+
TC0100002450.	-2.17	-1.12	2.17	down	8.47	9.59	lnc-C4BPB-	lnc-C4BPB-	NONHSAT0091	chr1	207113020	207114201	+
TC0100002545.	-2.47	-1.30	2.47	down	6.94	8.24	lnc-CENPF-	lnc-CENPF-	NONHSAT0094	chr1	214603195	214615190	+
TC0100002820.	-2.32	-1.21	2.32	down	4.22	5.44	lnc-FMN2-6	lnc-FMN2-6	---	chr1	239490915	239500115	+
TC0100003278.	-2.65	-1.41	2.65	down	4.52	5.92	lnc-HNRNPF	lnc-HNRNPF	---	chr1	23294917	23295117	-
TC0100003288.	-2.20	-1.14	2.20	down	4.85	5.99	lnc-E2F2-1	lnc-E2F2-1:1	NONHSAT0015	chr1	23557934	23559075	-
TC0100003810.	-2.55	-1.35	2.55	down	4.24	5.59	lnc-TACSTD	lnc-TACSTD	NONHSAT0035	chr1	58654740	58669291	-
TC0100004035.	-2.31	-1.21	2.31	down	7.17	8.38	lnc-GBP3-1	lnc-GBP3-1:	NONHSAT0043	chr1	89053437	89056059	-
TC0100004124.	-2.21	-1.14	2.21	down	5.06	6.20	lnc-LPPR5.1	lnc-LPPR5.1	NONHSAT0047	chr1	97855030	97920997	-
TC0100004216.	-2.07	-1.05	2.07	down	3.64	4.69	lnc-HBXIP-1	lnc-HBXIP-1	---	chr1	110368399	110373003	-
TC0100004224.	2.08	1.06	2.08	up	4.44	3.38	lnc-LRIF1-1/	lnc-LRIF1-1:	NONHSAT0051	chr1	110936369	110942353	-
TC0100004267.	2.03	1.02	2.03	up	8.33	7.31	lnc-RSBN1-	lnc-RSBN1-	NONHSAT0053	chr1	113699875	113705981	-
TC0100004282.	2.55	1.35	2.55	up	4.24	2.89	lnc-BCAS2-	lnc-BCAS2-	---	chr1	114558476	114558681	-
TC0100004320.	2.12	1.08	2.12	up	4.30	3.22	lnc-GDAP2-	lnc-GDAP2-	NONHSAT0055	chr1	118031746	118039342	-
TC0100004353.	-2.34	-1.23	2.34	down	15.82	17.04	lnc-FCGR1B	lnc-FCGR1B	NONHSAT0056	chr1	121742259	121743636	-
TC0100004366.	-2.04	-1.03	2.04	down	14.09	15.12	lnc-PPIAL4G	lnc-PPIAL4G	NONHSAT0057	chr1	143964912	143971749	-
TC0100004566.	-2.14	-1.10	2.14	down	3.70	4.80	lnc-PKLR-6	lnc-PKLR-6:	---	chr1	155390130	155390878	-
TC0100004574.	3.19	1.67	3.19	up	4.52	2.85	RP11-243J1	lnc-YY1AP1-	---	chr1	155710098	155710563	-
TC0100004678.	-2.38	-1.25	2.38	down	4.07	5.32	lnc-MPZ-1	lnc-MPZ-1:1	NONHSAT0071	chr1	161289361	161292479	-
TC0100004759.	2.29	1.19	2.29	up	3.95	2.76	lnc-SELL-1	lnc-SELL-1:1	NONHSAT0074	chr1	169731491	169732718	-
TC0100004896.	2.23	1.16	2.23	up	8.48	7.32	lnc-NMNAT	lnc-NMNAT	NONHSAT0083	chr1	183149709	183150022	-
TC0100004927.	-2.17	-1.12	2.17	down	5.56	6.68	lnc-PDC-2	lnc-PDC-2:1	NONHSAT0084	chr1	186678270	186680423	-
TC0100005207.	-2.10	-1.07	2.10	down	3.33	4.40	lnc-GPATCH	lnc-GPATCH	NONHSAT0095	chr1	218344196	218346024	-
TC0100005437.	-2.31	-1.21	2.31	down	6.25	7.45	lnc-TOMM2	lnc-TOMM2	NONHSAT0103	chr1	235155088	235160962	-
TC0100005452.	-2.20	-1.14	2.20	down	7.87	9.01	lnc-GNG4-8	lnc-GNG4-8	NONHSAT0103	chr1	235737990	235744032	-
TC0100005468.	-2.45	-1.29	2.45	down	4.31	5.61	lnc-ZP4-1//	lnc-ZP4-1:1/	NONHSAT0104	chr1	238480384	238486023	-
TC01000760.hg	-2.03	-1.02	2.03	down	4.65	5.67	ZRANB2-AS	---	---	chr1	71046506	71067184	+
TC01002614.hg	-2.77	-1.47	2.77	down	4.14	5.61	RPS15AP10	---	---	chr1	45645780	45646685	-
TC01003552.hg	-2.14	-1.10	2.14	down	5.41	6.51	SCARNA3	---	---	chr1	175968397	175968540	-
TC01006390.hg	-2.03	-1.02	2.03	down	9.53	10.56	HNRNPU-AS	---	---	chr1	244840638	244846941	-
TC0200000058.	2.36	1.24	2.36	up	9.26	8.02	lnc-DCDC2C	lnc-DCDC2C	NONHSAT0687	chr2	3971137	3972278	+
TC0200000120.	2.13	1.09	2.13	up	8.10	7.01	lnc-HPCAL1	lnc-HPCAL1	NONHSAT0690	chr2	10433225	10433830	+
TC0200000289.	2.05	1.03	2.05	up	5.08	4.05	lnc-FAM59B	lnc-FAM59B	---	chr2	26066569	26068517	+
TC0200000335.	-2.75	-1.46	2.75	down	5.12	6.58	RP11-373D2	lnc-BRE-2:1	NONHSAT0698	chr2	28396815	28397110	+
TC0200000422.	-2.03	-1.02	2.03	down	4.75	5.77	lnc-AC0073	lnc-AC0073	NONHSAT0701	chr2	37213467	37216193	+
TC0200000425.	-2.15	-1.10	2.15	down	5.69	6.79	lnc-C2orf56	lnc-C2orf56	NONHSAT0701	chr2	37344728	37360053	+
TC0200000426.	-2.07	-1.05	2.07	down	11.79	12.84	lnc-C2orf56	lnc-C2orf56	NONHSAT0701	chr2	37367123	37373030	+

TC0200001002.	-2.22	-1.15	2.22	down	6.61	7.76	lnc-AC0734	lnc-AC0734	NONHSAT0723	chr2	92085371	92106501	+
TC0200001130.	2.22	1.15	2.22	up	9.91	8.76	lnc-TMEM1	lnc-TMEM1	NONHSAT0728	chr2	102684298	102686768	+
TC0200001350.	3.12	1.64	3.12	up	4.78	3.15	AC079988.3	lnc-GLI2-5:1	NONHSAT0740	chr2	121178327	121182580	+
TC0200001529.	-2.08	-1.06	2.08	down	5.21	6.27	AC092620.2	lnc-HNMT-	NONHSAT0746	chr2	138569090	138574458	+
TC0200001552.	-2.41	-1.27	2.41	down	3.14	4.41	lnc-KYNU-1	lnc-KYNU-1	---	chr2	143673873	143674279	+
TC0200001559.	-2.03	-1.02	2.03	down	3.50	4.52	lnc-ARHGA	lnc-ARHGA	---	chr2	144147257	144147504	+
TC0200001651.	-2.27	-1.18	2.27	down	4.98	6.16	lnc-GPD2-7	lnc-GPD2-7	NONHSAT0750	chr2	157300803	157302597	+
TC0200001694.	2.13	1.09	2.13	up	3.71	2.62	lnc-GCA-7	lnc-GCA-7:1	---	chr2	161896758	161896964	+
TC0200001725.	2.41	1.27	2.41	up	5.14	3.87	lnc-NOSTRI	lnc-NOSTRI	---	chr2	168482916	168483272	+
TC0200001880.	2.16	1.11	2.16	up	4.07	2.95	lnc-UBE2E3-	lnc-UBE2E3-	---	chr2	180114178	180121819	+
TC0200001913.	-2.27	-1.18	2.27	down	3.99	5.17	lnc-ZC3H15	lnc-ZC3H15	NONHSAT0759	chr2	185739286	185745067	+
TC0200001988.	-2.20	-1.14	2.20	down	4.78	5.91	lnc-COQ10	lnc-COQ10	NONHSAT0761	chr2	196701109	196712711	+
TC0200002123.	-2.03	-1.02	2.03	down	5.34	6.36	lnc-CREB1-	lnc-CREB1-	NONHSAT0766	chr2	207602618	207605558	+
TC0200002140.	-2.14	-1.10	2.14	down	5.94	7.04	lnc-RPE-3	lnc-RPE-3:1	NONHSAT0766	chr2	210022407	210024522	+
TC0200002188.	2.23	1.15	2.23	up	5.41	4.25	lnc-XRCC5-	lnc-XRCC5-	NONHSAT0767	chr2	216209817	216220192	+
TC0200002248.	2.16	1.11	2.16	up	3.92	2.81	lnc-WNT10	lnc-WNT10	NONHSAT0769	chr2	218903962	218904353	+
TC0200002711.	2.18	1.12	2.18	up	9.84	8.72	lnc-NOL10-	lnc-NOL10-	NONHSAT0691	chr2	10444095	10448309	-
TC0200002987.	-2.84	-1.51	2.84	down	6.84	8.35	lnc-ATL2-4	lnc-ATL2-4:	NONHSAT0701	chr2	38071176	38076154	-
TC0200003023.	-2.42	-1.27	2.42	down	4.82	6.10	lnc-CDKL4-	lnc-CDKL4-	---	chr2	39323328	39323804	-
TC0200003305.	-2.19	-1.13	2.19	down	5.59	6.72	lnc-RAB1A-	lnc-RAB1A-	NONHSAT0711	chr2	65341978	65343774	-
TC0200003467.	2.06	1.04	2.06	up	4.10	3.06	lnc-REG3A-	lnc-REG3A-	NONHSAT0718	chr2	79492419	79493364	-
TC0200003567.	-2.10	-1.07	2.10	down	2.74	3.81	lnc-EIF2AK3	lnc-EIF2AK3	NONHSAT0722	chr2	88922328	88922753	-
TC0200003593.	2.39	1.26	2.39	up	10.55	9.29	lnc-AC2332	lnc-AC2332	NONHSAT0723	chr2	91988793	91991465	-
TC0200003780.	2.06	1.04	2.06	up	3.79	2.75	lnc-EDAR-8	lnc-EDAR-8:	NONHSAT0730	chr2	108049200	108052755	-
TC0200003944.	-2.03	-1.02	2.03	down	4.25	5.28	lnc-MAP3K2	lnc-MAP3K2	NONHSAT0741	chr2	127501097	127502163	-
TC0200004100.	-2.30	-1.20	2.30	down	5.38	6.58	lnc-GTDC1-	lnc-GTDC1-	NONHSAT0747	chr2	144385475	144385990	-
TC0200004194.	-2.27	-1.19	2.27	down	3.74	4.92	OTTHUMG0	lnc-ACVR1-	NONHSAT0750	chr2	157725708	157736005	-
TC0200004237.	-2.05	-1.03	2.05	down	2.83	3.87	lnc-FAP-3	lnc-FAP-3:1	NONHSAT0752	chr2	162307095	162308459	-
TC0200004245.	-2.33	-1.22	2.33	down	6.69	7.91	lnc-COBLL1	lnc-COBLL1	NONHSAT0752	chr2	164508663	164568484	-
TC0200004305.	-2.43	-1.28	2.43	down	4.76	6.04	lnc-METTL8	lnc-METTL8	NONHSAT0754	chr2	171318653	171321011	-
TC0200004363.	2.28	1.19	2.28	up	3.98	2.78	lnc-ATF2-2	lnc-ATF2-2:	NONHSAT0755	chr2	175051859	175060971	-
TC0200004462.	-2.07	-1.05	2.07	down	14.94	15.99	lnc-CALCRL	lnc-CALCRL	NONHSAT0760	chr2	187464233	187466042	-
TC0200004622.	-2.02	-1.01	2.02	down	5.07	6.08	AC083900.1	lnc-FZD5-1:	NONHSAT0766	chr2	207868582	207869915	-
TC0200004634.	-2.07	-1.05	2.07	down	9.96	11.01	lnc-ERBB4-1	lnc-ERBB4-1	NONHSAT0766	chr2	211298941	211299189	-
TC0200004783.	2.33	1.22	2.33	up	3.98	2.75	lnc-PID1-4	lnc-PID1-4:1	---	chr2	229474976	229475189	-
TC02001247.hg	-2.01	-1.01	2.01	down	2.98	3.99	CPS1-IT1	---	---	chr2	210617571	210619875	+
TC02002013.hg	2.12	1.08	2.12	up	4.27	3.19	SNAR-H	---	---	chr2	77954907	77955026	-
TC0300000063.	-2.01	-1.01	2.01	down	6.28	7.29	lnc-THUMP	lnc-THUMP	NONHSAT0877	chr3	9470667	9475705	+
TC0300000168.	2.05	1.04	2.05	up	3.95	2.91	RP11-415F2	lnc-OXNAD	---	chr3	16339308	16339871	+
TC0300000258.	-2.74	-1.45	2.74	down	3.28	4.73	lnc-RBMS3-	lnc-RBMS3-	NONHSAT0888	chr3	30665503	30666074	+
TC0300000317.	-2.07	-1.05	2.07	down	5.33	6.38	lnc-C3orf35	lnc-C3orf35	NONHSAT0890	chr3	37255863	37256470	+
TC0300000377.	-2.59	-1.37	2.59	down	3.31	4.69	lnc-VIPR1-5	lnc-VIPR1-5	---	chr3	42062116	42062409	+
TC0300000787.	-2.27	-1.18	2.27	down	9.87	11.05	lnc-EPHA3-	lnc-EPHA3-	NONHSAT0907	chr3	90414967	90427984	+
TC0300000799.	-2.52	-1.34	2.52	down	3.44	4.78	lnc-AC1104	lnc-AC1104	NONHSAT0907	chr3	97793372	97801107	+
TC0300000986.	2.97	1.57	2.97	up	4.78	3.21	lnc-C3orf30	lnc-C3orf30	NONHSAT0913	chr3	117648753	117655970	+
TC0300001013.	2.01	1.01	2.01	up	4.27	3.27	lnc-PLA1A-	lnc-PLA1A-	NONHSAT0913	chr3	119594727	119596361	+
TC0300001177.	2.12	1.08	2.12	up	11.05	9.97	lnc-TMEM1	lnc-TMEM1	NONHSAT0921	chr3	132881939	132882300	+
TC0300001299.	-2.36	-1.24	2.36	down	3.20	4.44	lnc-AC1070	lnc-AC1070	NONHSAT0925	chr3	146162870	146163801	+
TC0300001321.	-2.58	-1.37	2.58	down	10.11	11.48	lnc-GYG1-4	lnc-GYG1-4	NONHSAT0926	chr3	149167215	149168467	+

TC0300001339.	-3.07	-1.62	3.07	down	3.13	4.75	lnc-TSC22D	lnc-TSC22D	NONHSAT0926	chr3	150463032	150466431	+
TC0300001371.	2.02	1.01	2.02	up	4.23	3.22	lnc-P2RY1-1	lnc-P2RY1-1	NONHSAT0927	chr3	152839390	152841439	+
TC0300001439.	2.24	1.16	2.24	up	4.12	2.96	lnc-MFSD1-	lnc-MFSD1-	NONHSAT0929	chr3	158868484	158885184	+
TC0300001463.	-2.15	-1.11	2.15	down	8.27	9.38	lnc-OTOL1-	lnc-OTOL1-	NONHSAT0930	chr3	161723213	161723607	+
TC0300001662.	-2.21	-1.14	2.21	down	10.70	11.84	lnc-CHRD-3	lnc-CHRD-3	NONHSAT0937	chr3	184365007	184365523	+
TC0300002088.	-2.13	-1.09	2.13	down	7.70	8.79	lnc-EOMES-	lnc-EOMES-	NONHSAT0887	chr3	28331691	28332437	-
TC0300002187.	-2.02	-1.02	2.02	down	3.90	4.92	lnc-ULK4-1	lnc-ULK4-1:	NONHSAT0891	chr3	41220847	41225051	-
TC0300002314.	-2.02	-1.01	2.02	down	5.57	6.58	lnc-QRICH1	lnc-QRICH1	NONHSAT0896	chr3	49026545	49029398	-
TC0300002507.	-3.32	-1.73	3.32	down	7.13	8.86	lnc-LMOD3-	lnc-LMOD3-	NONHSAT0903	chr3	69196719	69250248	-
TC0300002561.	-2.41	-1.27	2.41	down	5.75	7.02	lnc-GBE1-1	lnc-GBE1-1	NONHSAT0905	chr3	78938005	78938292	-
TC0300002596.	2.05	1.03	2.05	up	4.72	3.69	lnc-MINA-1	lnc-MINA-1	NONHSAT0907	chr3	96244732	96245335	-
TC0300002628.	-2.03	-1.02	2.03	down	2.95	3.97	lnc-ZBTB11-	lnc-ZBTB11-	NONHSAT0908	chr3	101599243	101599644	-
TC0300002656.	-2.11	-1.08	2.11	down	5.90	6.97	lnc-CD47-4	lnc-CD47-4:	NONHSAT0910	chr3	108043298	108054739	-
TC0300002713.	2.44	1.29	2.44	up	4.70	3.41	lnc-DRD3-2	lnc-DRD3-2	NONHSAT0912	chr3	113978495	114001771	-
TC0300002790.	2.01	1.01	2.01	up	11.00	9.99	lnc-ITGB5-2	lnc-ITGB5-2	NONHSAT0915	chr3	124920167	124927771	-
TC0300002977.	-2.08	-1.06	2.08	down	4.71	5.77	RP11-383G	lnc-XRN1-1:	NONHSAT0924	chr3	142465315	142472337	-
TC0300003024.	3.02	1.59	3.02	up	5.18	3.59	lnc-CP-1	lnc-CP-1:1	NONHSAT0926	chr3	149229445	149241439	-
TC0300003077.	-2.09	-1.06	2.09	down	5.22	6.28	lnc-GPR149	lnc-GPR149	NONHSAT0928	chr3	154309796	154312248	-
TC0300003103.	-3.27	-1.71	3.27	down	3.49	5.20	lnc-SSR3-8	lnc-SSR3-8:	---	chr3	156772926	156776376	-
TC0300003180.	2.19	1.13	2.19	up	5.03	3.90	lnc-GOLIM4	lnc-GOLIM4	NONHSAT0931	chr3	167956311	167956616	-
TC0300003212.	-2.02	-1.02	2.02	down	2.95	3.96	lnc-TNIK-4	lnc-TNIK-4:	---	chr3	171623304	171624862	-
TC0300003216.	-2.03	-1.02	2.03	down	5.77	6.79	NONHSAG0	lnc-PLD1-1:	NONHSAT0932	chr3	171876352	171900740	-
TC0300003275.	-2.55	-1.35	2.55	down	4.74	6.10	lnc-DNAJC1	lnc-DNAJC1	NONHSAT0934	chr3	180608444	180609247	-
TC0300003381.	-2.24	-1.16	2.24	down	3.08	4.25	lnc-CLDN1-	lnc-CLDN1-	NONHSAT0939	chr3	189974573	189983484	-
TC0400000306.	-2.19	-1.13	2.19	down	4.74	5.87	lnc-STIM2-1	lnc-STIM2-1	NONHSAT0959	chr4	30728313	30729985	+
TC0400000312.	-2.02	-1.02	2.02	down	7.43	8.45	NONHSAG0	lnc-PCDH7-	NONHSAT0959	chr4	31997379	32155406	+
TC0400000314.	-2.14	-1.10	2.14	down	6.04	7.13	lnc-PCDH7-	lnc-PCDH7-	NONHSAT0959	chr4	32225022	32229895	+
TC0400000370.	2.08	1.05	2.08	up	8.27	7.22	lnc-UBE2K-	lnc-UBE2K-	NONHSAT0961	chr4	39812590	39814219	+
TC0400000414.	-2.06	-1.04	2.06	down	4.39	5.43	lnc-GUF1-5	lnc-GUF1-5:	---	chr4	44660625	44680348	+
TC0400000416.	-2.64	-1.40	2.64	down	5.33	6.73	RP11-700J1	lnc-GUF1-4:	---	chr4	44704405	44704965	+
TC0400000855.	-2.00	-1.00	2.00	down	9.33	10.33	lnc-C4orf32	lnc-C4orf32	NONHSAT0978	chr4	111551854	111552173	+
TC0400000956.	-2.44	-1.29	2.44	down	4.27	5.56	NONHSAG0	lnc-ADAD1-	NONHSAT0981	chr4	122268356	122270971	+
TC0400001052.	-2.82	-1.50	2.82	down	4.86	6.36	lnc-RAB33B	lnc-RAB33B	NONHSAT0984	chr4	139370639	139371247	+
TC0400001074.	-2.35	-1.23	2.35	down	2.85	4.08	lnc-ZNF330	lnc-ZNF330	NONHSAT0985	chr4	141719131	141720767	+
TC0400001169.	-2.16	-1.11	2.16	down	5.68	6.79	lnc-FHDC1-	lnc-FHDC1-	NONHSAT0988	chr4	152779993	152810388	+
TC0400001192.	-2.07	-1.05	2.07	down	3.12	4.17	lnc-LRAT-1	lnc-LRAT-1:	NONHSAT0988	chr4	154626945	154749420	+
TC0400001254.	-2.23	-1.16	2.23	down	5.71	6.86	lnc-C4orf43	lnc-C4orf43	---	chr4	163529771	163530697	+
TC0400001289.	2.13	1.09	2.13	up	9.35	8.26	lnc-CLCN3-	lnc-CLCN3-	NONHSAT0991	chr4	169821120	169821740	+
TC0400001305.	-2.41	-1.27	2.41	down	9.59	10.86	lnc-GALNT7	lnc-GALNT7	NONHSAT0992	chr4	173141643	173141936	+
TC0400001372.	-2.35	-1.24	2.35	down	6.99	8.22	lnc-CDKN2A	lnc-CDKN2A	NONHSAT0994	chr4	183271212	183281386	+
TC0400001570.	-2.03	-1.02	2.03	down	4.80	5.82	lnc-LYAR-2	lnc-LYAR-2:	NONHSAT0949	chr4	4267701	4268932	-
TC0400001732.	2.37	1.25	2.37	up	4.31	3.07	lnc-LGI2-3	lnc-LGI2-3:1	NONHSAT0958	chr4	24821057	24823045	-
TC0400001804.	-2.18	-1.13	2.18	down	9.60	10.72	lnc-C4orf34	lnc-C4orf34	NONHSAT0960	chr4	39547301	39549586	-
TC0400001841.	-2.39	-1.26	2.39	down	4.32	5.58	lnc-GNPDA2	lnc-GNPDA2	---	chr4	44693946	44694386	-
TC0400001916.	-2.04	-1.03	2.04	down	5.97	7.00	lnc-PPAT-2	lnc-PPAT-2:	NONHSAT0964	chr4	56355300	56387458	-
TC0400001957.	-2.10	-1.07	2.10	down	3.07	4.14	lnc-CENPC1	lnc-CENPC1	---	chr4	67100569	67104596	-
TC0400002012.	-2.42	-1.28	2.42	down	9.35	10.62	lnc-CXCL5-1	lnc-CXCL5-1	NONHSAT0968	chr4	73995642	73996738	-
TC0400002015.	-2.11	-1.08	2.11	down	4.53	5.61	lnc-CXCL3-	lnc-CXCL3-	NONHSAT0968	chr4	74097676	74099195	-
TC0400002226.	-2.68	-1.42	2.68	down	4.19	5.61	lnc-MANBA	lnc-MANBA	NONHSAT0975	chr4	102262110	102262601	-

TC0400002241.	-4.40	-2.14	4.40	down	9.59	11.73	lnc-BDH2-3	lnc-BDH2-3	NONHSAT0976	chr4	103138370	103140407	-
TC0400002242.	-2.92	-1.54	2.92	down	8.03	9.57	lnc-BDH2-4	lnc-BDH2-4	NONHSAT0976	chr4	103145303	103145781	-
TC0400002343.	-2.26	-1.17	2.26	down	5.93	7.10	lnc-PRSS12-	lnc-PRSS12-	NONHSAT0980	chr4	118723521	118728847	-
TC0400002411.	-2.11	-1.08	2.11	down	4.11	5.19	lnc-SCLT1-4	lnc-SCLT1-4	NONHSAT0982	chr4	128864921	128936704	-
TC0400002574.	2.43	1.28	2.43	up	6.56	5.28	lnc-FBXW7-	lnc-FBXW7-	NONHSAT0988	chr4	152628249	152641425	-
TC0400002585.	-2.34	-1.23	2.34	down	5.34	6.57	lnc-FGG-2	lnc-FGG-2:1	NONHSAT0988	chr4	154586679	154590749	-
TC0400002616.	-3.75	-1.91	3.75	down	8.05	9.96	lnc-C4orf46	lnc-C4orf46	NONHSAT0989	chr4	158130800	158173023	-
TC0400002631.	-2.05	-1.03	2.05	down	6.62	7.65	lnc-NPY1R-	lnc-NPY1R-	NONHSAT0990	chr4	163129120	163133642	-
TC0400002827.	-2.10	-1.07	2.10	down	4.45	5.52	RP11-237D	lnc-TRIML2-	NONHSAT0996	chr4	187516153	187517208	-
TC04000083.hg	-3.52	-1.82	3.52	down	4.94	6.75	AFAP1-AS1	---	---	chr4	7754090	7778927	+
TC04000297.hg	-2.68	-1.42	2.68	down	6.84	8.26	DANCR	---	---	chr4	52712430	52720351	+
TC0500000003.	2.03	1.02	2.03	up	4.47	3.45	lnc-LRRC14E	lnc-LRRC14E	NONHSAT0998	chr5	195778	196341	+
TC0500000029.	-2.10	-1.07	2.10	down	6.87	7.94	lnc-RP11-66	lnc-RP11-66	NONHSAT0999	chr5	915928	917900	+
TC0500000254.	-2.02	-1.01	2.02	down	4.57	5.59	lnc-PRDM9-	lnc-PRDM9-	---	chr5	20647584	20718223	+
TC0500000339.	-2.50	-1.32	2.50	down	6.65	7.98	lnc-BRIX1-2	lnc-BRIX1-2	NONHSAT1010	chr5	34953526	34954855	+
TC0500000526.	2.62	1.39	2.62	up	4.48	3.09	lnc-NDUFAF	lnc-NDUFAF	NONHSAT1016	chr5	61191550	61194023	+
TC0500000555.	-2.26	-1.18	2.26	down	5.44	6.61	lnc-CTC-53	lnc-CTC-53	NONHSAT1017	chr5	65655634	65664766	+
TC0500000599.	-2.10	-1.07	2.10	down	10.42	11.49	lnc-CENPH-	lnc-CENPH-	NONHSAT1018	chr5	69177061	69177697	+
TC0500000684.	-2.42	-1.27	2.42	down	7.81	9.09	lnc-ANKDD1	lnc-ANKDD1	NONHSAT1022	chr5	75597114	75598040	+
TC0500000791.	-2.01	-1.01	2.01	down	4.87	5.88	lnc-COX7C-	lnc-COX7C-	NONHSAT1025	chr5	85663232	85664684	+
TC0500000792.	-2.18	-1.12	2.18	down	4.40	5.53	lnc-COX7C-	lnc-COX7C-	NONHSAT1025	chr5	85805306	85821594	+
TC0500001078.	2.05	1.04	2.05	up	4.29	3.25	lnc-PRDM6-	lnc-PRDM6-	NONHSAT1035	chr5	123236079	123236370	+
TC0500001106.	-2.25	-1.17	2.25	down	5.11	6.28	RP11-517I3.	lnc-GRAMD	NONHSAT1035	chr5	126492938	126498604	+
TC0500001223.	-2.13	-1.09	2.13	down	6.96	8.05	lnc-TGFBI-3	lnc-TGFBI-3	NONHSAT1039	chr5	136132904	136177386	+
TC0500001242.	-2.01	-1.01	2.01	down	6.95	7.96	lnc-WNT8A-	lnc-WNT8A-	NONHSAT1040	chr5	138179168	138181896	+
TC0500001353.	-2.15	-1.10	2.15	down	5.53	6.63	lnc-RBM27-	lnc-RBM27-	NONHSAT1043	chr5	145997218	145998003	+
TC0500001365.	-2.05	-1.03	2.05	down	6.46	7.49	lnc-POU4F3	lnc-POU4F3	---	chr5	146463010	146463380	+
TC0500001502.	-2.31	-1.21	2.31	down	7.39	8.60	lnc-FABP6-	lnc-FABP6-	NONHSAT1048	chr5	160421869	160424777	+
TC0500001525.	-2.08	-1.06	2.08	down	6.74	7.79	lnc-CCNG1-	lnc-CCNG1-	NONHSAT1049	chr5	163460653	163469910	+
TC0500001867.	2.09	1.06	2.09	up	4.31	3.25	RP11-315A1	---	---	chr5	8717684	8717883	-
TC0500002090.	2.83	1.50	2.83	up	4.93	3.43	lnc-C5orf42	lnc-C5orf42	---	chr5	36738088	36741534	-
TC0500002123.	-3.85	-1.94	3.85	down	4.63	6.57	lnc-FYB-1	lnc-FYB-1:1	NONHSAT1011	chr5	39105224	39105755	-
TC0500002554.	-2.00	-1.00	2.00	down	7.30	8.30	lnc-AC0273	lnc-AC0273	NONHSAT1027	chr5	91375000	91381020	-
TC0500002715.	-2.05	-1.04	2.05	down	10.28	11.32	lnc-ATG12-	lnc-ATG12-	NONHSAT1033	chr5	116051910	116052289	-
TC0500002861.	2.05	1.03	2.05	up	4.25	3.22	lnc-FSTL4-4	lnc-FSTL4-4	NONHSAT1038	chr5	133720495	133720934	-
TC0500002910.	2.40	1.26	2.40	up	3.68	2.42	lnc-BRD8-2	lnc-BRD8-2	NONHSAT1040	chr5	138115407	138129435	-
TC0500002966.	2.29	1.19	2.29	up	4.27	3.08	AC005609.2	---	---	chr5	140867513	140867959	-
TC05000369.hg	2.38	1.25	2.38	up	4.20	2.95	NCRUPAR	---	---	chr5	76711938	76712861	+
TC05000379.hg	2.08	1.06	2.08	up	4.38	3.32	ZBED3-AS1	---	---	chr5	77086740	77166909	+
TC05001459.hg	-2.20	-1.14	2.20	down	6.26	7.40	SMA4//SMA	---	---	chr5	71126661	71289696	-
TC0600000310.	-2.28	-1.19	2.28	down	4.91	6.10	lnc-EDN1-1	lnc-EDN1-1	---	chr6	12322288	12334804	+
TC0600000363.	-2.34	-1.23	2.34	down	3.26	4.48	lnc-RNF144	lnc-RNF144	---	chr6	19043749	19060836	+
TC0600000829.	2.38	1.25	2.38	up	4.01	2.75	lnc-ENPP4-	lnc-ENPP4-	NONHSAT1130	chr6	46215075	46217183	+
TC0600000845.	-2.26	-1.17	2.26	down	10.48	11.65	lnc-GPR111	lnc-GPR111	NONHSAT1130	chr6	47607872	47609513	+
TC0600000848.	3.08	1.62	3.08	up	5.55	3.93	lnc-OPN5-1	lnc-OPN5-1	NONHSAT1130	chr6	47857041	47870362	+
TC0600000863.	2.10	1.07	2.10	up	3.92	2.85	RP3-335N17	lnc-IL17A-2	NONHSAT1130	chr6	51599723	51622884	+
TC0600000867.	-2.19	-1.13	2.19	down	3.29	4.43	lnc-EFHC1-	lnc-EFHC1-	---	chr6	52392289	52393551	+
TC0600000970.	2.17	1.12	2.17	up	3.79	2.67	lnc-OGFRL1	lnc-OGFRL1	NONHSAT1135	chr6	71475859	71479484	+
TC0600001133.	-4.00	-2.00	4.00	down	4.80	6.80	lnc-MANEA	lnc-MANEA	NONHSAT1140	chr6	92616829	92636951	+

TC0600001305.	-2.52	-1.33	2.52	down	4.48	5.81	RP11-346D1	lnc-MARCKS	NONHSAT1145	chr6	113531117	113543793	+
TC0600001466.	2.05	1.04	2.05	up	5.61	4.57	lnc-PDE7B-7	lnc-PDE7B-7	NONHSAT1150	chr6	135190128	135190347	+
TC0600001488.	-3.72	-1.90	3.72	down	10.08	11.98	lnc-KIAA124	lnc-KIAA124	NONHSAT1151	chr6	137874861	137875744	+
TC0600001593.	-2.17	-1.12	2.17	down	4.80	5.91	lnc-SUMO4-	lnc-SUMO4-	NONHSAT1154	chr6	149383833	149385871	+
TC0600001942.	-2.03	-1.02	2.03	down	4.45	5.48	lnc-TUBB2B-	lnc-TUBB2B-	NONHSAT1070	chr6	3318745	3319393	-
TC0600001984.	-2.73	-1.45	2.73	down	4.15	5.60	lnc-C6orf14	lnc-C6orf14	NONHSAT1072	chr6	3999188	3999469	-
TC0600002070.	-2.10	-1.07	2.10	down	5.76	6.83	lnc-NEDD9-	lnc-NEDD9-	NONHSAT1077	chr6	11713675	11736714	-
TC0600002362.	-2.56	-1.36	2.56	down	3.67	5.03	NONHSAG0	lnc-AGER-1:	NONHSAT1088	chr6	32184733	32185882	-
TC0600002803.	-2.07	-1.05	2.07	down	5.11	6.16	lnc-GPR63-9	lnc-GPR63-9	NONHSAT1140	chr6	96486802	96487037	-
TC0600002836.	-2.16	-1.11	2.16	down	4.23	5.34	lnc-SIM1-3	lnc-SIM1-3:	---	chr6	100632828	100633057	-
TC0600003013.	-2.20	-1.14	2.20	down	7.65	8.78	lnc-C6orf17	lnc-C6orf17	NONHSAT1148	chr6	127289780	127315860	-
TC0600003020.	-2.14	-1.10	2.14	down	6.63	7.73	lnc-THEMIS-	lnc-THEMIS-	NONHSAT1148	chr6	128062066	128067677	-
TC0600003062.	2.14	1.10	2.14	up	3.84	2.74	lnc-SLC2A12	lnc-SLC2A12	NONHSAT1150	chr6	133913374	133913680	-
TC0600003105.	-2.37	-1.24	2.37	down	3.98	5.22	lnc-IFNGR1-	lnc-IFNGR1-	NONHSAT1151	chr6	137206642	137219449	-
TC0600003358.	2.05	1.04	2.05	up	5.86	4.83	lnc-RNASET	lnc-RNASET	NONHSAT1160	chr6	166933375	166957191	-
TC0600003380.	2.17	1.12	2.17	up	7.88	6.76	lnc-FRMD1-	lnc-FRMD1-	NONHSAT1161	chr6	167999884	168000704	-
TC0700000155.	-2.04	-1.03	2.04	down	7.82	8.85	lnc-ARL4A-4	lnc-ARL4A-4	NONHSAT1192	chr7	12595549	12625071	+
TC0700000246.	-2.50	-1.32	2.50	down	4.19	5.51	lnc-C7orf30-	lnc-C7orf30-	NONHSAT1195	chr7	23270043	23274550	+
TC0700000261.	-2.55	-1.35	2.55	down	6.14	7.49	lnc-NPY-3	lnc-NPY-3:1	---	chr7	24660137	24660535	+
TC0700000454.	2.40	1.26	2.40	up	5.78	4.52	lnc-STK17A-	lnc-STK17A-	NONHSAT1202	chr7	43479608	43484615	+
TC0700000512.	2.37	1.24	2.37	up	4.28	3.03	lnc-ABCA13	lnc-ABCA13	---	chr7	48814482	48816241	+
TC0700000571.	-2.17	-1.11	2.17	down	7.08	8.19	lnc-MRPS17	lnc-MRPS17	NONHSAT1207	chr7	55981771	55983790	+
TC0700000616.	2.08	1.06	2.08	up	4.40	3.34	lnc-AC0064	lnc-AC0064	NONHSAT1208	chr7	63209219	63211822	+
TC0700000652.	-2.63	-1.40	2.63	down	4.08	5.48	lnc-ZNF273-	lnc-ZNF273-	NONHSAT1210	chr7	64933273	64933616	+
TC0700001045.	-2.11	-1.08	2.11	down	8.30	9.38	lnc-PRKRIP1	lnc-PRKRIP1	NONHSAT1224	chr7	102353233	102355362	+
TC0700001127.	2.20	1.14	2.20	up	4.38	3.24	lnc-C7orf53-	lnc-C7orf53-	NONHSAT1227	chr7	112616479	112617262	+
TC0700001149.	-2.18	-1.13	2.18	down	11.41	12.53	lnc-CAPZA2	lnc-CAPZA2	NONHSAT1229	chr7	116736098	116736353	+
TC0700001249.	2.56	1.36	2.56	up	4.25	2.89	NONHSAG0	lnc-FAM71F	NONHSAT1232	chr7	128667043	128668156	+
TC0700001268.	-2.05	-1.03	2.05	down	4.44	5.48	lnc-SMO-2	lnc-SMO-2:	---	chr7	129289715	129300167	+
TC0700001471.	-2.03	-1.02	2.03	down	3.79	4.81	lnc-C7orf33-	lnc-C7orf33-	NONHSAT1239	chr7	148580401	148580799	+
TC0700001571.	-2.36	-1.24	2.36	down	5.12	6.36	lnc-HTR5A-	lnc-HTR5A-	NONHSAT1243	chr7	155106400	155106651	+
TC0700001616.	-2.70	-1.43	2.70	down	4.53	5.96	lnc-DNAJB6	lnc-DNAJB6	NONHSAT1244	chr7	157233867	157236135	+
TC0700001777.	-2.56	-1.36	2.56	down	5.62	6.97	lnc-VWDE-5	lnc-VWDE-5	---	chr7	12320554	12321017	-
TC0700002036.	2.13	1.09	2.13	up	4.85	3.76	lnc-C7orf11-	lnc-C7orf11-	NONHSAT1202	chr7	39788967	39789304	-
TC0700002096.	2.89	1.53	2.89	up	10.76	9.23	lnc-AC0112	lnc-AC0112	NONHSAT1204	chr7	45913773	45915325	-
TC0700002416.	-2.17	-1.12	2.17	down	3.50	4.62	NONHSAG0	lnc-C7orf62-	NONHSAT1219	chr7	90266034	90270216	-
TC0700002495.	-2.18	-1.12	2.18	down	6.19	7.31	lnc-CYP3A7-	lnc-CYP3A7-	NONHSAT1222	chr7	99658833	99663582	-
TC0700002508.	-2.03	-1.02	2.03	down	11.03	12.05	lnc-GATS-1	lnc-GATS-1:	---	chr7	100310318	100310676	-
TC0700002629.	-2.12	-1.08	2.12	down	3.01	4.10	lnc-DOCK4-	lnc-DOCK4-	---	chr7	111262233	111262497	-
TC0700002632.	2.22	1.15	2.22	up	10.59	9.44	lnc-IMMP2L	lnc-IMMP2L	NONHSAT1227	chr7	111778327	111788920	-
TC0700002644.	2.06	1.05	2.06	up	7.13	6.08	lnc-TMEM16	lnc-TMEM16	---	chr7	112903961	112905818	-
TC0700002689.	-4.00	-2.00	4.00	down	4.23	6.23	lnc-TSPAN1	lnc-TSPAN1	NONHSAT1229	chr7	119680992	119682812	-
TC0700002690.	-2.32	-1.22	2.32	down	5.92	7.13	NONHSAG0	lnc-TSPAN1	NONHSAT1229	chr7	119704556	119907375	-
TC0700002783.	-2.06	-1.04	2.06	down	3.45	4.49	lnc-AC0080	lnc-AC0080	NONHSAT1234	chr7	132957766	132959571	-
TC0700002803.	-2.40	-1.26	2.40	down	4.56	5.83	lnc-WDR91-	lnc-WDR91-	NONHSAT1235	chr7	135385014	135386828	-
TC0700002823.	-2.03	-1.02	2.03	down	4.66	5.68	lnc-ZC3HAV	lnc-ZC3HAV	NONHSAT1236	chr7	139049456	139050006	-
TC07000196.hg	-3.50	-1.81	3.50	down	5.32	7.12	DKFZP586I1	---	---	chr7	30370050	30372794	+
TC07000381.hg	-2.16	-1.11	2.16	down	2.65	3.76	YWHAEP1	---	---	chr7	64433693	64435517	+
TC07001115.hg	2.35	1.23	2.35	up	3.73	2.50	ZNF890P	---	---	chr7	5121310	5144546	-

TC07001262.hg	-2.79	-1.48	2.79	down	6.92	8.40	RP9P	---	---	chr7	32916815	32943176	-
TC0800000101.	-2.01	-1.01	2.01	down	4.54	5.55	lnc-MTMR9-	lnc-MTMR9-	NONHSAT12504	chr8	11256256	11256603	+
TC0800000200.	-2.20	-1.14	2.20	down	4.51	5.65	lnc-NPM2-3	lnc-NPM2-3	NONHSAT12543	chr8	21924797	21926965	+
TC0800000347.	-2.27	-1.19	2.27	down	7.82	9.01	lnc-GS1-211	lnc-GS1-211	NONHSAT12590	chr8	31119956	31124965	+
TC0800000493.	-2.20	-1.14	2.20	down	5.32	6.46	lnc-RP11-11	lnc-RP11-11	NONHSAT12643	chr8	47164870	47165524	+
TC0800000535.	-2.01	-1.01	2.01	down	3.92	4.93	CTD-3214K2	lnc-NPBWR1	NONHSAT12657	chr8	52722903	52723141	+
TC0800000610.	-2.53	-1.34	2.53	down	5.33	6.67	lnc-CLVS1-5	lnc-CLVS1-5	NONHSAT12687	chr8	61662818	61663398	+
TC0800000690.	-2.36	-1.24	2.36	down	5.07	6.32	lnc-KCNB2-	lnc-KCNB2-	NONHSAT12720	chr8	71895991	71896330	+
TC0800000847.	-3.72	-1.90	3.72	down	7.14	9.03	lnc-DPY19L4	lnc-DPY19L4	NONHSAT12779	chr8	94793492	94793843	+
TC0800000970.	-2.08	-1.06	2.08	down	5.94	6.99	lnc-ZFPM2-	lnc-ZFPM2-	NONHSAT12825	chr8	106740243	106742484	+
TC0800001122.	-2.06	-1.04	2.06	down	4.81	5.85	lnc-PHF20L1	lnc-PHF20L1	NONHSAT12917	chr8	131997877	131999700	+
TC0800001371.	-2.10	-1.07	2.10	down	4.19	5.27	RP11-556O5	---	---	chr8	8188535	8189195	-
TC0800001815.	-3.14	-1.65	3.14	down	6.84	8.49	lnc-MOS-2	lnc-MOS-2:1	NONHSAT12668	chr8	56168306	56179536	-
TC0800001856.	-3.07	-1.62	3.07	down	8.27	9.88	lnc-CA8-12	lnc-CA8-12:	NONHSAT12687	chr8	61631680	61632012	-
TC0800001881.	2.06	1.04	2.06	up	4.46	3.42	lnc-ARMC1-	lnc-ARMC1-	---	chr8	64747127	64747328	-
TC0800001935.	-2.11	-1.08	2.11	down	3.61	4.69	lnc-PRDM14	lnc-PRDM14	NONHSAT12715	chr8	70119879	70125628	-
TC0800002023.	2.25	1.17	2.25	up	5.82	4.66	lnc-ZNF704-	lnc-ZNF704-	NONHSAT12743	chr8	81029791	81070176	-
TC0800002082.	-2.16	-1.11	2.16	down	5.91	7.02	lnc-CALB1-2	lnc-CALB1-2	NONHSAT12763	chr8	89982404	89984665	-
TC0800002133.	-3.05	-1.61	3.05	down	6.72	8.33	RP11-267M2	lnc-KIAA142	NONHSAT12777	chr8	94427712	94429888	-
TC0800002167.	2.07	1.05	2.07	up	8.50	7.45	lnc-HRSP12-	lnc-HRSP12-	NONHSAT12791	chr8	98195691	98205176	-
TC0800002187.	-2.43	-1.28	2.43	down	3.78	5.06	lnc-FBXO43-	lnc-FBXO43-	NONHSAT12796	chr8	100038523	100042768	-
TC0800002266.	-2.55	-1.35	2.55	down	6.13	7.48	lnc-DPYS-4	lnc-DPYS-4:	NONHSAT12821	chr8	104504244	104588995	-
TC0800002289.	-2.02	-1.02	2.02	down	13.47	14.49	lnc-RSPO2-2	lnc-RSPO2-2	NONHSAT12826	chr8	108228177	108229398	-
TC0800002326.	-2.59	-1.37	2.59	down	4.62	5.99	lnc-EXT1-2	lnc-EXT1-2:1	NONHSAT12840	chr8	117712391	117713058	-
TC0800002331.	-2.18	-1.12	2.18	down	3.98	5.10	SAMD12	---	---	chr8	118189455	118189970	-
TC0800002341.	-2.23	-1.16	2.23	down	4.45	5.61	lnc-ENPP2-3	lnc-ENPP2-3	NONHSAT12843	chr8	119480279	119480775	-
TC0800002374.	-2.16	-1.11	2.16	down	9.67	10.78	lnc-ZHX1-6	lnc-ZHX1-6:	NONHSAT12854	chr8	123372662	123380914	-
TC0800002375.	-3.10	-1.63	3.10	down	6.01	7.64	lnc-ATAD2-	lnc-ATAD2-	NONHSAT12855	chr8	123497890	123502818	-
TC0800002376.	-2.19	-1.13	2.19	down	3.92	5.06	lnc-ATAD2-	lnc-ATAD2-	NONHSAT12855	chr8	123523800	123525468	-
TC0800002444.	2.61	1.39	2.61	up	9.33	7.94	lnc-GSDMC-	lnc-GSDMC-	NONHSAT12912	chr8	129706110	129707182	-
TC0800002481.	2.12	1.08	2.12	up	10.66	9.57	lnc-NDRG1-	lnc-NDRG1-	NONHSAT12923	chr8	133454853	133458864	-
TC08001644.hg	-3.16	-1.66	3.16	down	3.06	4.72	ASAP1-IT1	---	---	chr8	130295355	130296533	-
TC0900000093.	-2.02	-1.01	2.02	down	10.51	11.52	lnc-KDM4C-	lnc-KDM4C-	NONHSAT13014	chr9	6639139	6639604	+
TC0900000187.	-2.28	-1.19	2.28	down	3.51	4.70	lnc-C9orf53-	lnc-C9orf53-	NONHSAT13040	chr9	21907093	21935636	+
TC0900000270.	-2.46	-1.30	2.46	down	2.83	4.13	lnc-ANKRD1	lnc-ANKRD1	NONHSAT13073	chr9	33500947	33502753	+
TC0900000415.	-2.02	-1.02	2.02	down	6.59	7.61	lnc-FAM27A	lnc-FAM27A	NONHSAT13142	chr9	41404033	41404967	+
TC0900000459.	-2.02	-1.02	2.02	down	3.30	4.31	lnc-FAM27D	lnc-FAM27D	NONHSAT13148	chr9	62690033	62690534	+
TC0900000462.	2.15	1.11	2.15	up	4.74	3.64	lnc-RP11-26	lnc-RP11-26	NONHSAT13153	chr9	62817551	62817849	+
TC0900000470.	2.84	1.51	2.84	up	17.75	16.24	lnc-ANKRD2	lnc-ANKRD2	NONHSAT13158	chr9	63398685	63400433	+
TC0900000678.	2.02	1.02	2.02	up	4.80	3.79	lnc-CKS2-3	lnc-CKS2-3:	---	chr9	89156609	89174673	+
TC0900000691.	-2.30	-1.20	2.30	down	3.98	5.18	lnc-SYK-9	lnc-SYK-9:1	NONHSAT13300	chr9	90325336	90331622	+
TC0900000736.	-2.23	-1.16	2.23	down	5.25	6.41	lnc-PHF2-1	lnc-PHF2-1:	NONHSAT13327	chr9	93538928	93560578	+
TC0900000944.	-2.14	-1.09	2.14	down	4.82	5.91	lnc-SNX30-2	lnc-SNX30-2	NONHSAT13402	chr9	112628470	112629703	+
TC0900001591.	-2.60	-1.38	2.60	down	2.90	4.28	lnc-UBAP2-5	lnc-UBAP2-5	---	chr9	33722975	33728416	-
TC0900001629.	-2.10	-1.07	2.10	down	3.10	4.17	lnc-GBA2-2	lnc-GBA2-2:	---	chr9	35716828	35717160	-
TC0900001703.	-2.01	-1.01	2.01	down	10.25	11.26	lnc-FAM27B	lnc-FAM27B	NONHSAT13155	chr9	40725871	40728205	-
TC0900001709.	-2.15	-1.10	2.15	down	7.74	8.84	lnc-FOXD4L	lnc-FOXD4L	NONHSAT13160	chr9	41131356	63975474	-
TC0900001778.	-2.13	-1.09	2.13	down	5.81	6.90	lnc-FOXD4L	lnc-FOXD4L	NONHSAT13132	chr9	65288041	65291364	-
TC0900001845.	-2.11	-1.08	2.11	down	2.89	3.97	lnc-TRPM6-	lnc-TRPM6-	NONHSAT13191	chr9	74251370	74275021	-

TC0900001903.	-3.90	-1.96	3.90	down	5.54	7.51	lnc-KIF27-2	lnc-KIF27-2:	NONHSAT1327	chr9	83784794	83789105	-
TC0900001997.	-2.41	-1.27	2.41	down	5.64	6.91	lnc-ZNF484	lnc-ZNF484	---	chr9	92839884	92843519	-
TC0900002011.	2.11	1.07	2.11	up	3.97	2.89	lnc-FBP2-3	lnc-FBP2-3:	NONHSAT1333	chr9	94485452	94511578	-
TC09000606.hg	-2.03	-1.02	2.03	down	5.20	6.22	PSMD5-AS1	---	---	chr9	120843042	120854373	+
TC0X00000059	2.06	1.04	2.06	up	4.19	3.15	lnc-HCCS-1	lnc-HCCS-1	NONHSAT1362	chrX	11021402	11028013	+
TC0X00000176	2.09	1.06	2.09	up	4.13	3.07	lnc-MAGEB1	lnc-MAGEB1	---	chrX	27500334	27543716	+
TC0X00000238	-2.70	-1.43	2.70	down	4.27	5.71	lnc-USP9X-	lnc-USP9X-	NONHSAT1367	chrX	41079535	41079880	+
TC0X00000330	-2.13	-1.09	2.13	down	4.85	5.94	RP11-1148L	---	---	chrX	48580741	48581165	+
TC0X00000365	-2.20	-1.14	2.20	down	10.01	11.15	lnc-GSPT2-	lnc-GSPT2-	NONHSAT1370	chrX	51893640	51901560	+
TC0X00000473	-2.09	-1.06	2.09	down	3.86	4.92	lnc-EFNB1-1	lnc-EFNB1-1	NONHSAT1373	chrX	69030978	69037112	+
TC0X00000606	-2.48	-1.31	2.48	down	3.24	4.55	lnc-TGIF2LX	lnc-TGIF2LX	NONHSAT1378	chrX	90112650	90113706	+
TC0X00000632	-2.02	-1.01	2.02	down	8.64	9.65	NONHSAG0	lnc-DIAPH2-	NONHSAT1378	chrX	98573801	98866800	+
TC0X00000669	-2.01	-1.01	2.01	down	4.54	5.55	lnc-BHLHB9	lnc-BHLHB9	NONHSAT1379	chrX	102785667	102786604	+
TC0X00000673	-2.29	-1.19	2.29	down	3.55	4.74	lnc-BHLHB9	lnc-BHLHB9	NONHSAT1380	chrX	102799563	102799883	+
TC0X00000675	-2.15	-1.11	2.15	down	6.18	7.29	lnc-BHLHB9	lnc-BHLHB9	NONHSAT1380	chrX	102803041	102806138	+
TC0X00000676	-2.68	-1.42	2.68	down	5.00	6.42	lnc-BHLHB9	lnc-BHLHB9	NONHSAT1380	chrX	102806741	102807824	+
TC0X00000733	-2.50	-1.32	2.50	down	6.03	7.35	lnc-PAK3-5	lnc-PAK3-5:	NONHSAT1381	chrX	111745586	111747473	+
TC0X00000931	-2.64	-1.40	2.64	down	6.31	7.72	lnc-SPANXB	lnc-SPANXB	NONHSAT1388	chrX	141138478	141139174	+
TC0X00001026	-2.05	-1.04	2.05	down	4.98	6.02	lnc-FAM50A	lnc-FAM50A	NONHSAT1391	chrX	154470587	154471320	+
TC0X00001131	-2.07	-1.05	2.07	down	7.52	8.58	lnc-ASB11-1	lnc-ASB11-1	NONHSAT1363	chrX	15321713	15335525	-
TC0X00001187	-2.07	-1.05	2.07	down	8.46	9.51	lnc-ACOT9-	lnc-ACOT9-	NONHSAT1364	chrX	23833360	23840405	-
TC0X00001279	-2.04	-1.03	2.04	down	7.85	8.87	RP6-99M1.3	lnc-CXorf36	NONHSAT1368	chrX	45764772	45765299	-
TC0X00001446	2.06	1.04	2.06	up	3.84	2.80	NONHSAG0	lnc-OPHN1-	NONHSAT1373	chrX	68551263	68573108	-
TC0X00001477	-2.41	-1.27	2.41	down	2.83	4.10	lnc-CXCR3-	lnc-CXCR3-	---	chrX	71462930	71463326	-
TC0X00001513	-2.45	-1.29	2.45	down	4.33	5.63	RP13-204A1	---	---	chrX	74122134	74122725	-
TC0X00001531	-2.01	-1.01	2.01	down	5.36	6.37	lnc-CYSLTR1	lnc-CYSLTR1	NONHSAT1377	chrX	78129749	78130129	-
TC0X00001550	-2.27	-1.19	2.27	down	4.86	6.05	lnc-RPS6KA	lnc-RPS6KA	NONHSAT1377	chrX	83748825	83749939	-
TC0X00001562	-2.13	-1.09	2.13	down	11.18	12.27	lnc-CHM-1	lnc-CHM-1:	NONHSAT1377	chrX	87724061	87728512	-
TC0X00001568	-3.19	-1.67	3.19	down	6.03	7.70	lnc-NAP1L3	lnc-NAP1L3	NONHSAT1378	chrX	90039213	90039834	-
TC0X00001590	2.12	1.08	2.12	up	4.64	3.56	lnc-TSPAN6	lnc-TSPAN6	NONHSAT1378	chrX	100642177	100643703	-
TC0X00001848	2.07	1.05	2.07	up	4.42	3.37	lnc-IDS-13	lnc-IDS-13:1	---	chrX	147717123	147729850	-
TC0X000246.hg	-2.28	-1.19	2.28	down	5.01	6.20	SSX6	---	---	chrX	48107980	48120691	+
TC0X001309.hg	-2.02	-1.01	2.02	down	3.25	4.26	SLC25A5-AS	---	---	chrX	119466033	119469120	-
TC0Y00000097.	-2.15	-1.10	2.15	down	2.87	3.98	lnc-CDY2B-	lnc-CDY2B-	NONHSAT1395	chrY	17628513	17629402	+
TC1000000369.	2.08	1.05	2.08	up	4.02	2.96	lnc-C10orf6	lnc-C10orf6	NONHSAT0126	chr10	32481711	32499381	+
TC1000000372.	-2.03	-1.02	2.03	down	3.18	4.20	lnc-C10orf6	lnc-C10orf6	NONHSAT0126	chr10	32944481	32944999	+
TC1000000395.	2.04	1.03	2.04	up	5.50	4.47	lnc-CCNY-1	lnc-CCNY-1	NONHSAT0127	chr10	35210416	35210750	+
TC1000000551.	-2.15	-1.10	2.15	down	5.13	6.23	lnc-ZFAND4	lnc-ZFAND4	NONHSAT0133	chr10	49991567	49994556	+
TC1000000597.	-2.59	-1.38	2.59	down	4.12	5.49	lnc-CDK1-5	lnc-CDK1-5:	NONHSAT0135	chr10	60684505	60685209	+
TC1000000627.	2.25	1.17	2.25	up	3.90	2.73	lnc-LRRTM3	lnc-LRRTM3	NONHSAT0136	chr10	65784866	65785526	+
TC1000000642.	-2.05	-1.03	2.05	down	8.32	9.35	lnc-MYPN-	lnc-MYPN-	NONHSAT0136	chr10	67885181	67906879	+
TC1000000696.	2.12	1.09	2.12	up	4.66	3.57	RP11-327E2	lnc-SLC29A	NONHSAT0138	chr10	71364243	71366374	+
TC1000000889.	-2.22	-1.15	2.22	down	8.71	9.86	lnc-PAPSS2-	lnc-PAPSS2-	---	chr10	87868872	87869144	+
TC1000000893.	-2.11	-1.08	2.11	down	5.31	6.39	lnc-PAPSS2-	lnc-PAPSS2-	---	chr10	87936599	87937078	+
TC1000000920.	-2.03	-1.02	2.03	down	4.29	5.32	lnc-KIF20B-	lnc-KIF20B-	NONHSAT0155	chr10	89978717	89979070	+
TC1000000952.	-2.52	-1.33	2.52	down	6.45	7.78	lnc-O3FAR1	lnc-O3FAR1	NONHSAT0156	chr10	93517207	93520277	+
TC1000000968.	-2.47	-1.30	2.47	down	8.91	10.22	lnc-TBC1D1	lnc-TBC1D1	NONHSAT0157	chr10	94590742	94592514	+
TC1000000979.	2.29	1.20	2.29	up	3.96	2.76	lnc-CCNJ-7	lnc-CCNJ-7:	NONHSAT0157	chr10	95961574	95965953	+
TC1000001114.	-2.73	-1.45	2.73	down	4.02	5.47	lnc-TAF5-1	lnc-TAF5-1:	NONHSAT0161	chr10	103388869	103389079	+

TC1000001362	2.16	1.11	2.16	up	4.02	2.91	lnc-MGMT-	lnc-MGMT-	NONHSAT0169	chr10	129366185	129368688	+
TC1000001421	2.34	1.23	2.34	up	4.01	2.79	lnc-CYP2E1-	lnc-CYP2E1-	NONHSAT0171	chr10	133574398	133588982	+
TC1000001431	-2.09	-1.06	2.09	down	5.49	6.55	lnc-CYP2E1-	lnc-CYP2E1-	NONHSAT0171	chr10	133779170	133785043	+
TC1000001500	-2.40	-1.26	2.40	down	10.39	11.65	lnc-AKR1C2	lnc-AKR1C2	NONHSAT0111	chr10	5005995	5018031	-
TC1000001533	-2.40	-1.26	2.40	down	3.56	4.82	lnc-PRKCQ-	lnc-PRKCQ-	NONHSAT0112	chr10	6293100	6294684	-
TC1000001590	-2.15	-1.11	2.15	down	5.80	6.91	lnc-CCDC3-	lnc-CCDC3-	NONHSAT0114	chr10	13159477	13159777	-
TC1000001600	-3.83	-1.94	3.83	down	4.44	6.38	lnc-FRMD4	lnc-FRMD4	NONHSAT0115	chr10	14524483	14527860	-
TC1000001637	-2.31	-1.21	2.31	down	5.66	6.87	lnc-PTPLA-	lnc-PTPLA-	NONHSAT0116	chr10	18546062	18551822	-
TC1000001676	-2.01	-1.01	2.01	down	3.94	4.95	lnc-ENKUR-	lnc-ENKUR-	NONHSAT0117	chr10	24908522	24908812	-
TC1000001968	-2.92	-1.55	2.92	down	6.15	7.70	lnc-C10orf4	lnc-C10orf4	NONHSAT0135	chr10	60061921	60064292	-
TC1000001969	-2.06	-1.04	2.06	down	8.26	9.30	lnc-C10orf4	lnc-C10orf4	NONHSAT0135	chr10	60082508	60084715	-
TC1000001972	-2.08	-1.06	2.08	down	6.51	7.57	lnc-ANK3-1	lnc-ANK3-1	NONHSAT0135	chr10	60871174	60875043	-
TC1000001987	-2.45	-1.29	2.45	down	5.30	6.59	lnc-EGR2-4	lnc-EGR2-4	NONHSAT0135	chr10	63192939	63194694	-
TC1000001997	-2.29	-1.19	2.29	down	12.00	13.19	lnc-JMJD1C	lnc-JMJD1C	NONHSAT0136	chr10	64168954	64170850	-
TC1000002430	-2.29	-1.20	2.29	down	5.43	6.62	lnc-WDR96-	lnc-WDR96-	NONHSAT0162	chr10	104070461	104080654	-
TC1000002473	2.36	1.24	2.36	up	4.50	3.26	lnc-BBIP1-9	lnc-BBIP1-9	NONHSAT0163	chr10	111498191	111498941	-
TC1000002527	-2.20	-1.14	2.20	down	5.77	6.91	RP11-427L1	lnc-PRLHR-	NONHSAT0165	chr10	118692361	118693535	-
TC1000002619	-2.20	-1.14	2.20	down	3.48	4.62	lnc-DHX32-	lnc-DHX32-	NONHSAT0169	chr10	126012387	126014767	-
TC1000002675	2.13	1.09	2.13	up	8.28	7.19	lnc-NKX6-2	lnc-NKX6-2	NONHSAT0170	chr10	132821249	132824347	-
TC1000002685	2.39	1.26	2.39	up	8.77	7.51	lnc-ADAM8	lnc-ADAM8	NONHSAT0171	chr10	133111723	133112028	-
TC10001661.h	-2.58	-1.37	2.58	down	11.85	13.21	RPL13AP6	---	---	chr10	110936603	110937255	-
TC1100000217	-2.35	-1.23	2.35	down	3.91	5.14	lnc-MICAL2	lnc-MICAL2	---	chr11	11862185	11862412	+
TC1100000372	2.06	1.04	2.06	up	4.62	3.58	lnc-BBOX1-	lnc-BBOX1-	NONHSAT0185	chr11	28295270	28295600	+
TC1100000710	-2.42	-1.28	2.42	down	4.24	5.52	lnc-TMEM1	lnc-TMEM1	NONHSAT0216	chr11	61371271	61372643	+
TC1100001001	2.23	1.15	2.23	up	5.48	4.32	lnc-FAM86C	lnc-FAM86C	---	chr11	71681837	71682156	+
TC1100001045	-2.47	-1.31	2.47	down	8.59	9.90	lnc-ATG16L	lnc-ATG16L	NONHSAT0228	chr11	72940498	72941112	+
TC1100001317	-3.99	-2.00	3.99	down	6.72	8.72	lnc-BIRC2-2	lnc-BIRC2-2	NONHSAT0238	chr11	102336521	102337291	+
TC1100001319	-3.44	-1.78	3.44	down	4.36	6.14	lnc-BIRC2-3	lnc-BIRC2-3	NONHSAT0238	chr11	102396336	102396551	+
TC1100001532	2.39	1.26	2.39	up	4.13	2.88	lnc-UBASH3	lnc-UBASH3	NONHSAT0248	chr11	122197584	122197801	+
TC1100001749	3.68	1.88	3.68	up	12.69	10.81	NONHSAG0	lnc-C11orf8	NONHSAT0174	chr11	1995130	2001710	-
TC1100002020	-2.13	-1.09	2.13	down	4.99	6.08	OTTHUMG0	lnc-CCDC34	NONHSAT0184	chr11	27047186	27220113	-
TC1100002025	-2.20	-1.14	2.20	down	3.72	4.86	lnc-CCDC34	lnc-CCDC34	---	chr11	27386604	27386916	-
TC1100002041	2.28	1.19	2.28	up	4.24	3.05	lnc-KIF18A-	lnc-KIF18A-	NONHSAT0185	chr11	28839415	28843016	-
TC1100002248	-3.71	-1.89	3.71	down	5.16	7.05	lnc-OR4C46	lnc-OR4C46	NONHSAT0213	chr11	54543571	54559115	-
TC1100002576	-2.04	-1.03	2.04	down	3.37	4.40	lnc-SHANK2	lnc-SHANK2	NONHSAT0226	chr11	70358198	70358677	-
TC1100002613	-2.66	-1.41	2.66	down	4.91	6.32	lnc-STARD1	lnc-STARD1	---	chr11	73082360	73082635	-
TC1100002627	-2.07	-1.05	2.07	down	4.06	5.11	lnc-UCP3-4	lnc-UCP3-4	NONHSAT0228	chr11	73975670	73977064	-
TC1100002760	-2.10	-1.07	2.10	down	4.93	6.00	lnc-CCDC89	lnc-CCDC89	---	chr11	85694324	85694600	-
TC1100002894	2.83	1.50	2.83	up	8.71	7.21	lnc-MMP10-	lnc-MMP10-	NONHSAT0238	chr11	102797044	102797339	-
TC1100002895	4.54	2.18	4.54	up	10.23	8.05	lnc-MMP10-	lnc-MMP10-	NONHSAT0238	chr11	102797397	102798146	-
TC11000067.h	2.07	1.05	2.07	up	4.22	3.17	KCNQ1DN	---	---	chr11	2870033	2872106	+
TC11000177.h	-2.04	-1.03	2.04	down	3.60	4.63	SBF2-AS1	---	---	chr11	9758292	9811319	+
TC1200000154	2.04	1.03	2.04	up	5.70	4.68	lnc-ACSM4-	lnc-ACSM4-	NONHSAT0262	chr12	7496541	7499355	+
TC1200000314	-2.19	-1.13	2.19	down	5.42	6.55	NONHSAG0	lnc-AEBP2-	NONHSAT0271	chr12	19147074	19154659	+
TC1200000319	-2.06	-1.04	2.06	down	9.76	10.81	lnc-PLEKHA	lnc-PLEKHA	NONHSAT0272	chr12	19413153	19413461	+
TC1200000409	-2.20	-1.14	2.20	down	5.71	6.85	RP11-425D1	---	---	chr12	28236227	28236828	+
TC1200000446	-2.35	-1.23	2.35	down	7.05	8.28	lnc-C12orf3	lnc-C12orf3	NONHSAT0275	chr12	31959370	31993103	+
TC1200000814	2.02	1.01	2.02	up	4.33	3.32	lnc-XRCC6B	lnc-XRCC6B	NONHSAT0290	chr12	58103907	58105935	+
TC1200000840	-2.01	-1.01	2.01	down	4.64	5.65	lnc-MON2-	lnc-MON2-	NONHSAT0290	chr12	62658823	62659225	+

TC1200000882.	-2.41	-1.27	2.41	down	5.91	7.18	lnc-IRAK3-3	lnc-IRAK3-3	NONHSAT0292	chr12	65958627	65958854	+
TC1200000915.	-2.70	-1.43	2.70	down	5.81	7.25	lnc-NUP107	lnc-NUP107	---	chr12	68618365	68618589	+
TC1200000916.	-2.01	-1.00	2.01	down	7.13	8.13	lnc-NUP107	lnc-NUP107	NONHSAT0293	chr12	68625270	68625651	+
TC1200000918.	-2.01	-1.01	2.01	down	7.22	8.23	lnc-RAP1B-2	lnc-RAP1B-2	NONHSAT0293	chr12	68686971	68689910	+
TC1200000930.	2.10	1.07	2.10	up	12.44	11.37	lnc-LYZ-2	lnc-LYZ-2:1	NONHSAT0293	chr12	69239620	69258189	+
TC1200000931.	-2.82	-1.49	2.82	down	3.92	5.41	lnc-CPSF6-2	lnc-CPSF6-2	NONHSAT1400	chr12	69282933	69286071	+
TC1200000957.	-2.55	-1.35	2.55	down	6.85	8.20	lnc-TPH2-2	lnc-TPH2-2:	NONHSAT0294	chr12	71897847	71907762	+
TC1200001015.	2.90	1.53	2.90	up	4.82	3.29	lnc-OTOGL-	lnc-OTOGL-	---	chr12	79287284	79294843	+
TC1200001057.	-2.99	-1.58	2.99	down	3.31	4.90	lnc-TMTC3-	lnc-TMTC3-	NONHSAT0297	chr12	88418138	88419307	+
TC1200001092.	2.01	1.01	2.01	up	4.14	3.13	lnc-PLEKHG	lnc-PLEKHG	NONHSAT0298	chr12	92948193	92948491	+
TC1200001148.	2.02	1.01	2.02	up	4.49	3.48	lnc-NEDD1-	lnc-NEDD1-	---	chr12	97123766	97124015	+
TC1200001159.	-2.04	-1.03	2.04	down	4.80	5.83	lnc-APAF1-3	lnc-APAF1-3	NONHSAT0301	chr12	98599635	98599884	+
TC1200001175.	-2.17	-1.12	2.17	down	5.18	6.30	lnc-NR1H4-	lnc-NR1H4-	NONHSAT0302	chr12	100573723	100591801	+
TC1200001238.	-2.18	-1.12	2.18	down	6.67	7.79	lnc-POLR3B	lnc-POLR3B	NONHSAT0304	chr12	106303493	106311232	+
TC1200001266.	-2.12	-1.08	2.12	down	5.12	6.20	lnc-ACACB-	lnc-ACACB-	NONHSAT0305	chr12	109103967	109108004	+
TC1200001315.	-2.00	-1.00	2.00	down	4.80	5.80	lnc-ALDH2-	lnc-ALDH2-	NONHSAT0307	chr12	111843032	111843423	+
TC1200001393.	-2.47	-1.30	2.47	down	5.57	6.88	RP11-103B5	---	---	chr12	117002463	117003152	+
TC1200001400.	2.00	1.00	2.00	up	4.39	3.39	RP11-33N1	---	---	chr12	118037869	118038081	+
TC1200001682.	-2.06	-1.04	2.06	down	6.07	7.11	NONHSAG0	lnc-KDM5A-	NONHSAT0253	chr12	273954	277123	-
TC1200001829.	-2.30	-1.20	2.30	down	6.66	7.86	lnc-C3AR1-	lnc-C3AR1-	NONHSAT0262	chr12	7992688	8017180	-
TC1200001886.	-2.20	-1.14	2.20	down	5.25	6.39	lnc-MAGOH	lnc-MAGOH	NONHSAT1400	chr12	10588479	10599835	-
TC1200001892.	-2.20	-1.14	2.20	down	4.30	5.43	lnc-TAS2R4	lnc-TAS2R4	NONHSAT0269	chr12	11048332	11049256	-
TC1200002030.	2.06	1.04	2.06	up	4.27	3.23	RP11-996F1	---	---	chr12	29277397	29277882	-
TC1200002055.	2.07	1.05	2.07	up	8.54	7.48	lnc-FAM60A	lnc-FAM60A	NONHSAT0275	chr12	31424569	31426751	-
TC1200002151.	2.05	1.04	2.05	up	10.18	9.15	RP11-474P2	---	---	chr12	46239106	46239473	-
TC1200002566.	-2.62	-1.39	2.62	down	3.48	4.87	RP11-274M	lnc-KCNC2-	NONHSAT0295	chr12	74248637	74283669	-
TC1200002650.	-2.43	-1.28	2.43	down	6.55	7.83	lnc-KITLG-1	lnc-KITLG-1	NONHSAT0297	chr12	88381898	88419252	-
TC1200002695.	-3.26	-1.71	3.26	down	5.76	7.46	lnc-RP11-11	lnc-RP11-11	---	chr12	94316033	94319794	-
TC1200002763.	-2.01	-1.01	2.01	down	2.76	3.77	lnc-UHRF1B	lnc-UHRF1B	NONHSAT0301	chr12	99504598	99706624	-
TC1200002767.	-2.19	-1.13	2.19	down	4.53	5.66	lnc-ANKS1B	lnc-ANKS1B	NONHSAT0301	chr12	100054091	100054526	-
TC1200002787.	-2.04	-1.03	2.04	down	11.59	12.62	lnc-GNPTAB	lnc-GNPTAB	NONHSAT0303	chr12	101873316	101873623	-
TC1200002879.	-2.07	-1.05	2.07	down	3.72	4.77	lnc-TRPV4-2	lnc-TRPV4-2	NONHSAT0306	chr12	109859767	109860420	-
TC1200003019.	-2.09	-1.06	2.09	down	5.73	6.79	lnc-RAB35-3	lnc-RAB35-3	NONHSAT0310	chr12	119782156	119804491	-
TC1200003096.	-2.02	-1.02	2.02	down	6.62	7.64	RP11-214K3	lnc-DNAH1	NONHSAT0316	chr12	123966077	123966629	-
TC1200003135.	2.01	1.01	2.01	up	3.93	2.92	lnc-DHX37-	lnc-DHX37-	NONHSAT0317	chr12	126111496	126114789	-
TC1200003249.	-2.70	-1.43	2.70	down	3.59	5.02	lnc-CHFR-1	lnc-CHFR-1:	NONHSAT0321	chr12	132911519	132914732	-
TC12001154.hg	-2.35	-1.23	2.35	down	7.51	8.75	SCARNA12	---	---	chr12	6967337	6967606	-
TC12001224.hg	-2.79	-1.48	2.79	down	3.94	5.43	KLRAP1	---	---	chr12	10588478	10599835	-
TC12001418.hg	-2.44	-1.29	2.44	down	4.14	5.43	RACGAP1P	---	---	chr12	45062618	45065411	-
TC12001420.hg	-4.24	-2.08	4.24	down	4.06	6.14	RNY5	---	---	chr12	45187091	45187469	-
TC12001432.hg	-3.55	-1.83	3.55	down	7.99	9.82	PCED1B-AS	---	---	chr12	47205898	47216456	-
TC12001859.hg	-2.09	-1.06	2.09	down	5.24	6.30	TMPO-AS1	---	---	chr12	98512973	98516422	-
TC12001975.hg	-2.50	-1.32	2.50	down	6.28	7.60	MAPKAPK5-	---	---	chr12	111839767	111842902	-
TC1300000436.	-2.02	-1.01	2.02	down	9.43	10.44	lnc-HNRNP	lnc-HNRNP	NONHSAT0339	chr13	52455482	52461308	+
TC1300000460.	-2.03	-1.02	2.03	down	5.43	6.46	lnc-PRR20A	lnc-PRR20A	NONHSAT0340	chr13	55570028	55579839	+
TC1300000778.	2.46	1.30	2.46	up	4.21	2.92	lnc-DAOA-9	lnc-DAOA-9	---	chr13	105174561	105176591	+
TC1300000850.	2.11	1.08	2.11	up	4.81	3.73	lnc-SPACA7	lnc-SPACA7	NONHSAT0352	chr13	112437571	112438632	+
TC1300000960.	-2.10	-1.07	2.10	down	7.18	8.25	lnc-EFHA1-4	lnc-EFHA1-4	NONHSAT0323	chr13	21483834	21484174	-
TC1400000285.	-2.01	-1.01	2.01	down	7.67	8.67	lnc-GEMIN2	lnc-GEMIN2	NONHSAT0365	chr14	39175282	39176590	+

TC1400000324.	-2.10	-1.07	2.10	down	5.31	6.38	lnc-PRPF39-	lnc-PRPF39-	NONHSAT0366	chr14	45136514	45137959	+
TC1400000432.	-2.19	-1.13	2.19	down	4.24	5.38	lnc-LGALS3-	lnc-LGALS3-	NONHSAT0369	chr14	55272211	55355084	+
TC1400000437.	-2.00	-1.00	2.00	down	9.57	10.58	lnc-FBXO34	lnc-FBXO34	NONHSAT0370	chr14	55671862	55674304	+
TC1400000511.	-2.97	-1.57	2.97	down	8.00	9.57	lnc-C14orf1	lnc-C14orf1	NONHSAT0372	chr14	60296402	60299085	+
TC1400001219.	-2.02	-1.02	2.02	down	4.63	5.65	lnc-NOVA1-	lnc-NOVA1-	NONHSAT0361	chr14	27258717	27845286	-
TC1400001252.	-2.08	-1.05	2.08	down	7.38	8.44	lnc-HECTD1	lnc-HECTD1	NONHSAT0362	chr14	30912066	30932398	-
TC1400001375.	-2.14	-1.10	2.14	down	7.69	8.79	lnc-FKBP3-4	lnc-FKBP3-4	NONHSAT0366	chr14	45203837	45206444	-
TC1400001412.	-2.21	-1.14	2.21	down	5.94	7.08	RP11-247L2	lnc-CDKL1-	NONHSAT0367	chr14	50326526	50327909	-
TC1400001580.	-2.46	-1.30	2.46	down	3.49	4.79	lnc-ESR2-4	lnc-ESR2-4:	NONHSAT0373	chr14	64345022	64345453	-
TC1400001623.	-2.12	-1.08	2.12	down	4.77	5.85	lnc-VTI1B-2	lnc-VTI1B-2	NONHSAT0374	chr14	67589755	67592776	-
TC1400002137.	2.10	1.07	2.10	up	5.49	4.42	lnc-AL90160	lnc-AL90160	NONHSAT0404	chr14	106344395	106344833	-
TC14001213.hg	2.03	1.02	2.03	up	3.69	2.66	TEX21P	---	---	chr14	64345473	64347611	-
TC1500000133.	-3.55	-1.83	3.55	down	3.97	5.80	NONHSAG0	lnc-GOLGA8	NONHSAT0413	chr15	30648797	30649529	+
TC1500000235.	2.35	1.23	2.35	up	11.37	10.14	lnc-EIF2AK4	lnc-EIF2AK4	NONHSAT0417	chr15	39594068	39595551	+
TC1500000236.	2.06	1.04	2.06	up	7.35	6.31	lnc-EIF2AK4	lnc-EIF2AK4	---	chr15	39595680	39597832	+
TC1500000248.	-2.09	-1.06	2.09	down	4.14	5.21	RP11-133K1	---	---	chr15	40226386	40227069	+
TC1500000361.	-2.33	-1.22	2.33	down	5.50	6.73	lnc-SLC28A2	lnc-SLC28A2	NONHSAT0422	chr15	45271774	45275951	+
TC1500000820.	-2.00	-1.00	2.00	down	3.48	4.49	lnc-TMED3-	lnc-TMED3-	NONHSAT0476	chr15	79416783	79417331	+
TC1500000825.	2.22	1.15	2.22	up	3.70	2.55	lnc-AC0158	lnc-AC0158	NONHSAT0476	chr15	79984151	79985724	+
TC1500001511.	-2.22	-1.15	2.22	down	3.84	4.99	NONHSAG0	lnc-FBN1-2:	NONHSAT0423	chr15	48725138	48729844	-
TC1500001582.	-2.02	-1.01	2.02	down	7.97	8.98	lnc-ONECUT	lnc-ONECUT	NONHSAT0439	chr15	53665692	53684627	-
TC1500001614.	-2.47	-1.30	2.47	down	5.57	6.88	lnc-MNS1-5	lnc-MNS1-5	NONHSAT0440	chr15	56631843	56645339	-
TC1500001636.	-2.16	-1.11	2.16	down	5.73	6.84	lnc-SLTM-5	lnc-SLTM-5:	NONHSAT0441	chr15	58703852	58704154	-
TC1500001658.	-2.05	-1.04	2.05	down	3.62	4.66	AC092755.4	lnc-GTF2A2	---	chr15	59644298	59672302	-
TC1500001802.	-2.22	-1.15	2.22	down	3.84	4.99	lnc-TLE3-8	lnc-TLE3-8:1	---	chr15	69452821	69454086	-
TC1500001838.	-2.34	-1.23	2.34	down	4.78	6.00	lnc-GRAMD	lnc-GRAMD	NONHSAT0470	chr15	71822299	71823622	-
TC1500001842.	-2.02	-1.01	2.02	down	8.06	9.08	lnc-GRAMD	lnc-GRAMD	NONHSAT0470	chr15	72027764	72045864	-
TC1500002242.	2.20	1.14	2.20	up	4.95	3.81	lnc-LINS-1/	lnc-LINS-1:2	NONHSAT0511	chr15	100547752	100559852	-
TC15000150.hg	-2.14	-1.10	2.14	down	5.44	6.54	WHAMMP2	---	---	chr15	28737583	28779139	+
TC1600000015.	-2.02	-1.01	2.02	down	8.89	9.90	lnc-DECR2-	lnc-DECR2-	NONHSAT0517	chr16	398410	400749	+
TC1600000400.	-3.53	-1.82	3.53	down	4.59	6.41	lnc-AC0043	lnc-AC0043	NONHSAT1409	chr16	20783264	20786152	+
TC1600000755.	2.77	1.47	2.77	up	5.49	4.03	lnc-LONP2-	lnc-LONP2-	NONHSAT1423	chr16	48610270	48610696	+
TC1600000832.	-2.05	-1.04	2.05	down	4.04	5.08	RP11-454F8	---	---	chr16	53227146	53227694	+
TC1600001051.	-2.02	-1.01	2.02	down	4.37	5.38	lnc-TMCO7-	lnc-TMCO7-	NONHSAT1433	chr16	68812083	68813413	+
TC1600001300.	2.05	1.04	2.05	up	5.70	4.67	NONHSAG0	lnc-USP10-2	NONHSAT1440	chr16	84828263	84829242	+
TC1600001831.	-2.29	-1.19	2.29	down	3.93	5.12	lnc-ZP2-1	lnc-ZP2-1:1	NONHSAT1409	chr16	21227154	21233594	-
TC1600002100.	-2.55	-1.35	2.55	down	3.21	4.56	lnc-SHCBP1	lnc-SHCBP1	NONHSAT1422	chr16	46660696	46661591	-
TC1600002293.	2.11	1.08	2.11	up	4.43	3.35	RP11-229O3	---	---	chr16	64981123	64983227	-
TC17_ctg5_hap	2.04	1.03	2.04	up	4.02	2.99	KANSL1-AS	---	---	chr17_GL	590243	593393	-
TC1700000052.	-2.55	-1.35	2.55	down	4.57	5.92	lnc-SRR-4	lnc-SRR-4:1	NONHSAT1449	chr17	2361777	2363045	+
TC1700000063.	-2.49	-1.32	2.49	down	5.23	6.54	RP11-135N5	---	---	chr17	2639297	2642418	+
TC1700000278.	2.04	1.03	2.04	up	5.33	4.30	lnc-TBC1D2	lnc-TBC1D2	NONHSAT1458	chr17	15717285	15720452	+
TC1700000524.	-2.60	-1.38	2.60	down	3.17	4.55	lnc-TP53I13	lnc-TP53I13	---	chr17	29477175	29477628	+
TC1700000564.	-2.69	-1.43	2.69	down	4.41	5.84	RP11-142O6	lnc-RNF135	NONHSAT0528	chr17	31133182	31331946	+
TC1700000602.	-2.07	-1.05	2.07	down	6.51	7.56	lnc-RHBDL3	lnc-RHBDL3	NONHSAT0529	chr17	32368832	32378446	+
TC1700000612.	2.01	1.00	2.01	up	4.27	3.27	lnc-CDK5R1	lnc-CDK5R1	NONHSAT0529	chr17	32627739	32686484	+
TC1700000833.	-3.01	-1.59	3.01	down	3.25	4.84	lnc-NBR2-4	lnc-NBR2-4:	NONHSAT0538	chr17	43079302	43079780	+
TC1700001254.	-2.18	-1.12	2.18	down	6.98	8.10	lnc-PITPNC1	lnc-PITPNC1	NONHSAT0554	chr17	67224728	67225041	+
TC1700001297.	-2.45	-1.29	2.45	down	3.72	5.01	RP1-193H1	lnc-MAP2K6	NONHSAT0555	chr17	69551358	69553861	+

TC1700001485.	2.06	1.04	2.06	up	6.85	5.81	lnc-RPTOR-	lnc-RPTOR-	NONHSAT0564	chr17	80503999	80504512	+
TC1700001688.	-2.48	-1.31	2.48	down	5.00	6.31	lnc-C17orf8	lnc-C17orf8	---	chr17	3861362	3861592	-
TC1700001888.	-2.35	-1.23	2.35	down	3.86	5.09	lnc-TEKT3-1	lnc-TEKT3-1	NONHSAT1457	chr17	15240427	15244396	-
TC1700001898.	-2.07	-1.05	2.07	down	5.25	6.30	lnc-AC0058	lnc-AC0058	NONHSAT1458	chr17	15530773	15531089	-
TC1700002340.	-2.33	-1.22	2.33	down	6.31	7.53	lnc-GSDMB-	lnc-GSDMB-	NONHSAT0534	chr17	39921043	39921749	-
TC1700002735.	-2.02	-1.01	2.02	down	7.63	8.64	lnc-INTS2-3	lnc-INTS2-3	NONHSAT0551	chr17	61775878	61780277	-
TC1700002803.	-2.18	-1.12	2.18	down	3.39	4.52	lnc-AXIN2-2	lnc-AXIN2-2	NONHSAT0553	chr17	65805972	65808621	-
TC1700002837.	2.56	1.36	2.56	up	4.58	3.23	lnc-ABCA9-	lnc-ABCA9-	NONHSAT0555	chr17	69085654	69087618	-
TC1700002846.	2.63	1.40	2.63	up	4.55	3.16	lnc-ABCA5-	lnc-ABCA5-	NONHSAT0555	chr17	69357143	69359219	-
TC17001807.hg	2.56	1.36	2.56	up	7.62	6.27	AMZ2P1	---	---	chr17	64966550	64975585	-
TC17002871.hg	-2.09	-1.06	2.09	down	3.94	5.01	USP32P1	---	---	chr17	16786489	16816540	+
TC1800000041.	-2.15	-1.11	2.15	down	6.77	7.88	lnc-EMILIN2	lnc-EMILIN2	NONHSAT0569	chr18	2751379	2753129	+
TC1800000120.	-2.41	-1.27	2.41	down	6.61	7.88	RP11-888D1	lnc-TWSG1-	---	chr18	9259388	9260390	+
TC1800000224.	-2.17	-1.12	2.17	down	4.37	5.49	lnc-SNRPD1	lnc-SNRPD1	NONHSAT0585	chr18	21630138	21633524	+
TC1800000406.	2.61	1.38	2.61	up	4.23	2.85	lnc-SETBP1-	lnc-SETBP1-	NONHSAT0590	chr18	45168570	45169292	+
TC1800000903.	-2.16	-1.11	2.16	down	14.13	15.25	lnc-MC2R-2	lnc-MC2R-2	NONHSAT0584	chr18	13919603	13919923	-
TC1800000915.	2.24	1.16	2.24	up	4.45	3.28	lnc-POTEC-	lnc-POTEC-	NONHSAT0585	chr18	15159724	15164467	-
TC1800001013.	2.10	1.07	2.10	up	4.00	2.93	lnc-DSC3-2	lnc-DSC3-2:	NONHSAT0588	chr18	30157571	30175990	-
TC1900000289.	2.15	1.10	2.15	up	3.83	2.73	lnc-RDH8-3	lnc-RDH8-3	NONHSAT0610	chr19	10040140	10041229	+
TC1900000327.	2.01	1.01	2.01	up	4.91	3.90	CTC-510F12	---	---	chr19	11221083	11221573	+
TC1900000638.	-2.05	-1.04	2.05	down	3.24	4.27	lnc-ZNF254	lnc-ZNF254	NONHSAT0639	chr19	23935220	23936173	+
TC1900000649.	3.30	1.72	3.30	up	13.80	12.08	NONHSAG0	lnc-VSTM2B	NONHSAT0640	chr19	28606688	28615229	+
TC1900001388.	2.21	1.14	2.21	up	4.05	2.91	lnc-ZNF71-	lnc-ZNF71-	NONHSAT0682	chr19	56840851	56848556	+
TC1900001437.	-2.03	-1.02	2.03	down	7.55	8.58	lnc-ZNF584	lnc-ZNF584	NONHSAT0683	chr19	58387275	58388594	+
TC1900001819.	-2.05	-1.04	2.05	down	4.07	5.11	lnc-STX10-	lnc-STX10-	NONHSAT0613	chr19	13139617	13141147	-
TC1900001990.	2.06	1.04	2.06	up	4.38	3.34	lnc-ZNF682	lnc-ZNF682	NONHSAT0636	chr19	20175013	20177063	-
TC1900001993.	-2.02	-1.01	2.02	down	3.77	4.78	CTC-260E6.	lnc-ZNF682	NONHSAT0636	chr19	20220597	20222186	-
TC1900002038.	2.10	1.07	2.10	up	4.50	3.43	lnc-ZNF98-	lnc-ZNF98-	NONHSAT0637	chr19	22447934	22448777	-
TC1900002764.	2.02	1.01	2.02	up	4.69	3.68	lnc-ZNF835	lnc-ZNF835	NONHSAT0681	chr19	56810091	56811283	-
TC19000570.hg	3.91	1.97	3.91	up	6.84	4.87	CYP2B7P	---	---	chr19	40924265	40950660	+
TC2000000040.	-2.56	-1.36	2.56	down	10.02	11.37	lnc-TMC2-2	lnc-TMC2-2	---	chr20	2656939	2657010	+
TC2000000107.	-2.06	-1.04	2.06	down	8.20	9.24	lnc-PLCB4-4	lnc-PLCB4-4	NONHSAT0785	chr20	8649391	8652011	+
TC2000000137.	-2.13	-1.09	2.13	down	5.34	6.42	lnc-SPTLC3-	lnc-SPTLC3-	NONHSAT0786	chr20	13008979	13051018	+
TC2000000230.	2.25	1.17	2.25	up	4.50	3.33	lnc-SSTR4-1	lnc-SSTR4-1	---	chr20	22996824	22997331	+
TC2000001023.	2.36	1.24	2.36	up	4.39	3.15	lnc-FAM182	lnc-FAM182	---	chr20	25949536	25950299	-
TC2000001116.	-3.36	-1.75	3.36	down	7.31	9.06	lnc-NFS1-1	lnc-NFS1-1:	NONHSAT0795	chr20	35626044	35627340	-
TC2000001188.	-2.32	-1.21	2.32	down	4.11	5.32	RP4-620E11	---	---	chr20	41485571	41486225	-
TC20000157.hg	2.09	1.06	2.09	up	3.82	2.76	CST13P	---	---	chr20	23519146	23542018	+
TC20000872.hg	-2.02	-1.01	2.02	down	2.83	3.84	STK4-AS1	---	---	chr20	44963794	44966458	-
TC2100000016.	-2.01	-1.01	2.01	down	4.91	5.92	CH507-528	---	---	chr21	8383777	8410645	+
TC2100000032.	-2.16	-1.11	2.16	down	7.12	8.23	lnc-POTED-	lnc-POTED-	NONHSAT1471	chr21	13065573	13066975	+
TC2100000074.	-2.74	-1.46	2.74	down	4.00	5.46	lnc-USP25-	lnc-USP25-	---	chr21	16490978	16491248	+
TC2100000257.	-3.91	-1.97	3.91	down	3.62	5.58	LINC01436/	lnc-CBR1-3:	NONHSAT0819	chr21	36005320	36008295	+
TC2100000445.	-2.09	-1.06	2.09	down	4.85	5.92	lnc-FAM207	lnc-FAM207	NONHSAT0828	chr21	44849589	44850088	+
TC2100000550.	-2.19	-1.13	2.19	down	5.21	6.34	lnc-LIPI-5	lnc-LIPI-5:1	NONHSAT1471	chr21	13916917	13917802	-
TC2100000602.	-2.17	-1.12	2.17	down	3.31	4.42	lnc-TMPRSS	lnc-TMPRSS	---	chr21	18815146	18821622	-
TC2100000661.	-2.20	-1.13	2.20	down	3.10	4.23	lnc-ATP5J-1	lnc-ATP5J-1	NONHSAT0815	chr21	25762938	25763333	-
TC21000425.hg	-2.06	-1.04	2.06	down	4.12	5.16	RUNX1-IT1	---	---	chr21	35037936	35039426	-
TC2200000012.	-2.25	-1.17	2.25	down	5.87	7.04	lnc-AP00052	lnc-AP00052	NONHSAT0830	chr22	15850340	15877446	+

TC2200000248.	-2.50	-1.32	2.50	down	6.44	7.76	lnc-GGT1-4	lnc-GGT1-4:	NONHSAT0843	chr22	24686923	24688087	+
TC2200000277.	2.02	1.02	2.02	up	4.45	3.44	lnc-SEZ6L-1	lnc-SEZ6L-1	---	chr22	26161834	26164303	+
TC2200000424.	-3.27	-1.71	3.27	down	6.75	8.46	lnc-APOL6-:	lnc-APOL6-:	NONHSAT0850	chr22	35666257	35668386	+
TC2200000586.	2.02	1.01	2.02	up	6.57	5.55	lnc-BIK-2	lnc-BIK-2:1	NONHSAT0870	chr22	43095624	43097152	+
TC2200000597.	-2.23	-1.16	2.23	down	5.42	6.58	lnc-PARVB-:	lnc-PARVB-:	NONHSAT0871	chr22	43957027	43957348	+
TC2200000765.	2.02	1.01	2.02	up	3.80	2.79	XXbac-B33L	---	---	chr22	18501794	18512187	-
