# Supplementary material for: Differentially Expressed Long Noncoding RNAs Involved in FUBP1 Promoting Hepatocellular Carcinoma Cells Proliferation
Source: Biomed Res Int. 2021 Apr 14;2021:6664519. doi: 10.1155/2021/6664519 (PMC8063849; doi:10.1155/2021/6664519)
Supplement: Supplementary 2 — Differentially expressed long noncoding RNAs in MHCC97-L cells with or without FUBP1 overexpression. [file 6664519.f2.pdf]

Probe Set ID	FC ([97L-	Log FC ([	FC (abs)	Regulatio	97L-FUBF	97L-mo	GeneSymbol	Description	lncipediaID	NonCodeID	Chr	start	stop	strand
TC0100000152.oe.1	-2.41	-1.27	2.41	down	3.13	4.40	OTTHUMG0	N/A//unchar	lnc-SLC45A1	NONHSAT0	chr1	8208672	8215210	+
TC0100000238.oe.1	2.11	1.08	2.11	up	5.29	4.21	lnc-AADACL	NONCODE	lnc-AADACL	NONHSAT0	chr1	12497226	12506907	+
TC0100000356.oe.1	2.37	1.24	2.37	up	4.33	3.08	lnc-NBPF3-	gene_id XLC	lnc-NBPF3-	NONHSAT0	chr1	21418289	21420016	+
TC0100000378.oe.1	2.07	1.05	2.07	up	5.14	4.09	lnc-EPHB2-	LNCipedia l	lnc-EPHB2-	NONHSAT0	chr1	23080873	23083682	+
TC0100000390.oe.1	2.18	1.13	2.18	up	7.97	6.84	lnc-PITHD1-	LNCipedia l	lnc-PITHD1-	NONHSAT0	chr1	23744034	23750944	+
TC0100000392.oe.1	2.39	1.26	2.39	up	8.86	7.60	lnc-PITHD1-	NONCODE	lnc-PITHD1-	NONHSAT0	chr1	23794092	23795081	+
TC0100000412.oe.1	3.52	1.82	3.52	up	8.40	6.58	lnc-C1orf13	NONCODE	lnc-C1orf13	NONHSAT0	chr1	24666555	24669264	+
TC0100000454.oe.1	2.05	1.03	2.05	up	5.27	4.24	lnc-HMGN2	NONCODE	lnc-HMGN2	NONHSAT0	chr1	26551446	26554135	+
TC0100000480.oe.1	-2.16	-1.11	2.16	down	3.41	4.51	lnc-GPR3-1	NONCODE	lnc-GPR3-1:	NONHSAT0	chr1	27457198	27459582	+
TC0100000635.oe.1	-2.32	-1.21	2.32	down	3.37	4.59	lnc-DNALI1-	NONCODE	lnc-DNALI1-	NONHSAT0	chr1	37604422	37606253	+
TC0100000723.oe.1	2.36	1.24	2.36	up	11.36	10.12	lnc-KCNQ4-	NONCODE	lnc-KCNQ4-	NONHSAT0	chr1	41000813	41003176	+
TC0100000738.oe.1	2.70	1.43	2.70	up	4.74	3.30	lnc-PPCS-2	LNCipedia l	lnc-PPCS-2:	---	chr1	42451273	42454245	+
TC0100000757.oe.1	2.06	1.04	2.06	up	11.53	10.49	lnc-MPL-1	NONCODE	lnc-MPL-1:1	NONHSAT0	chr1	43361058	43363066	+
TC0100000781.oe.1	2.04	1.03	2.04	up	5.95	4.93	lnc-DMAP1-	NONCODE	lnc-DMAP1-	NONHSAT0	chr1	44412698	44419872	+
TC0100000893.oe.1	-2.04	-1.03	2.04	down	11.55	12.58	lnc-BTF3L4-	LNCipedia l	lnc-BTF3L4-	NONHSAT0	chr1	51745597	51759546	+
TC0100001095.oe.1	2.30	1.20	2.30	up	7.22	6.02	lnc-HHLA3-	NONCODE	lnc-HHLA3-	NONHSAT0	chr1	70434851	70439232	+
TC0100001119.oe.1	-2.37	-1.25	2.37	down	4.28	5.52	lnc-FPGT-TN	LNCipedia l	lnc-FPGT-TN	NONHSAT0	chr1	74198253	74249518	+
TC0100001157.oe.1	-2.32	-1.21	2.32	down	6.45	7.67	lnc-IFI44-8	NONCODE	lnc-IFI44-8:1	NONHSAT0	chr1	79696440	79700695	+
TC0100001374.oe.1	2.81	1.49	2.81	up	4.76	3.27	lnc-AMY2B-	NONCODE	lnc-AMY2B-	NONHSAT0	chr1	103577387	103579534	+
TC0100001406.oe.1	-2.24	-1.16	2.24	down	7.32	8.48	lnc-STXBP3-	NONCODE	lnc-STXBP3-	NONHSAT0	chr1	108746674	108757005	+
TC0100001509.oe.1	2.09	1.07	2.09	up	4.16	3.10	lnc-OLFML3	LNCipedia l	lnc-OLFML3	---	chr1	114007950	114063801	+
TC0100001609.oe.1	3.09	1.63	3.09	up	12.66	11.03	RNVU1-14	RNA, variant	---	---	chr1	145281116	145281462	+
TC0100001658.oe.1	2.15	1.11	2.15	up	5.37	4.26	OTTHUMG0	NONCODE	lnc-PDE4DIF	NONHSAT0	chr1	149048576	149051394	+
TC0100001705.oe.1	-2.17	-1.12	2.17	down	6.90	8.02	lnc-SETDB1-	LNCipedia l	lnc-SETDB1-	NONHSAT0	chr1	150982249	150984371	+
TC0100001759.oe.1	-2.01	-1.00	2.01	down	4.60	5.61	lnc-LELP1-2	LNCipedia l	lnc-LELP1-2	NONHSAT0	chr1	153140120	153150040	+
TC0100001801.oe.1	2.08	1.06	2.08	up	9.44	8.38	lnc-FLAD1-	LNCipedia l	lnc-FLAD1-	NONHSAT0	chr1	154990213	154993111	+
TC0100001942.oe.1	2.22	1.15	2.22	up	6.09	4.94	lnc-DDR2-2	LNCipedia l	lnc-DDR2-2	NONHSAT0	chr1	162790721	162812526	+
TC0100001984.oe.1	-2.38	-1.25	2.38	down	2.81	4.06	lnc-MAEL-3	LNCipedia l	lnc-MAEL-3	NONHSAT0	chr1	166981536	166982083	+
TC0100002062.oe.1	-2.05	-1.04	2.05	down	6.45	7.48	lnc-PRDX6-	LNCipedia l	lnc-PRDX6-	---	chr1	173261644	173261877	+
TC0100002080.oe.1	2.78	1.48	2.78	up	7.26	5.78	lnc-GPR52-	LNCipedia l	lnc-GPR52-	NONHSAT0	chr1	174220712	174243167	+
TC0100002107.oe.1	2.61	1.38	2.61	up	4.62	3.24	lnc-C1orf22	NONCODE	lnc-C1orf22	NONHSAT0	chr1	178569477	178570415	+
TC0100002119.oe.1	2.06	1.04	2.06	up	9.86	8.81	lnc-SOAT1-	NONCODE	lnc-SOAT1-	NONHSAT0	chr1	179201705	179201926	+
TC0100002133.oe.1	2.27	1.18	2.27	up	4.03	2.85	lnc-CEP350-	LNCipedia l	lnc-CEP350-	NONHSAT0	chr1	180131729	180134851	+
TC0100002141.oe.1	-2.43	-1.28	2.43	down	4.46	5.74	lnc-KIAA161	NONCODE	lnc-KIAA161	NONHSAT0	chr1	180887572	180888448	+
TC0100002178.oe.1	2.43	1.28	2.43	up	5.52	4.24	lnc-LAMC2-	LNCipedia l	lnc-LAMC2-	NONHSAT0	chr1	183138379	183141304	+
TC0100002276.oe.1	-2.48	-1.31	2.48	down	8.94	10.25	lnc-CFH-2	LNCipedia l	lnc-CFH-2:1	NONHSAT0	chr1	196676988	196747504	+
TC0100002285.oe.1	-2.68	-1.42	2.68	down	7.82	9.25	lnc-CFHR5-	LNCipedia l	lnc-CFHR5-	NONHSAT0	chr1	197363817	197364078	+
TC0100002288.oe.1	2.42	1.28	2.42	up	8.19	6.92	lnc-LHX9-2	LNCipedia l	lnc-LHX9-2:	NONHSAT0	chr1	197905517	197907364	+
TC0100002396.oe.1	2.24	1.16	2.24	up	5.54	4.38	lnc-MDM4-	LNCipedia l	lnc-MDM4-	NONHSAT0	chr1	204403390	204404742	+
TC0100002431.oe.1	-2.31	-1.21	2.31	down	3.11	4.32	lnc-C1orf18	LNCipedia l	lnc-C1orf18	---	chr1	206215584	206219787	+
TC0100002434.oe.1	2.76	1.47	2.76	up	5.16	3.70	lnc-IKBKE-2	NONCODE	lnc-IKBKE-2:	NONHSAT0	chr1	206453345	206454509	+
TC0100002486.oe.1	2.91	1.54	2.91	up	9.19	7.65	lnc-HSD11B	LNCipedia l	lnc-HSD11B	NONHSAT0	chr1	209675686	209675993	+
TC0100002578.oe.1	-2.30	-1.20	2.30	down	4.29	5.49	OTTHUMG0	LNCipedia l	lnc-LYPLAL1	---	chr1	219557192	219557701	+
TC0100002593.oe.1	-2.37	-1.24	2.37	down	5.59	6.83	lnc-MARC1-	NONCODE	lnc-MARC1-	NONHSAT0	chr1	220756278	220761764	+
TC0100003108.oe.1	-2.11	-1.08	2.11	down	3.69	4.77	lnc-DFFA-4	NONCODE	lnc-DFFA-4:	NONHSAT0	chr1	10637121	10638763	-
TC0100003170.oe.1	-2.37	-1.25	2.37	down	3.15	4.39	lnc-ZBTB17-	LNCipedia l	lnc-ZBTB17-	NONHSAT0	chr1	15917698	15917908	-
TC0100003209.oe.1	2.20	1.14	2.20	up	10.89	9.75	lnc-KIAA009	NONCODE	lnc-KIAA009	NONHSAT0	chr1	19144852	19146128	-
TC0100003280.oe.1	-2.09	-1.06	2.09	down	3.89	4.95	lnc-ASAP3-	LNCipedia l	lnc-ASAP3-	NONHSAT0	chr1	23408560	23419236	-

TC0100003462.oe.1	-2.07	-1.05	2.07	down	15.04	16.09	lnc-ZMYM6-	LNCipedia lo	lnc-ZMYM6-	---	chr1	34957213	34957413	-
TC0100003515.oe.1	2.23	1.16	2.23	up	5.64	4.48	lnc-MTF1-1	LNCipedia lo	lnc-MTF1-1:	NONHSAT0(	chr1	37809577	37813320	-
TC0100003645.oe.1	2.04	1.03	2.04	up	7.45	6.42	lnc-PIK3R3-	LNCipedia lo	lnc-PIK3R3-	NONHSAT0(	chr1	46193383	46194651	-
TC0100003802.oe.1	2.04	1.03	2.04	up	4.87	3.84	lnc-OMA1-3	LNCipedia lo	lnc-OMA1-3	NONHSAT0(	chr1	58228682	58229003	-
TC0100003889.oe.1	-2.03	-1.02	2.03	down	6.98	8.00	lnc-RPE65-1	NONCODE (	lnc-RPE65-1	NONHSAT0(	chr1	68475574	68482238	-
TC0100003946.oe.1	3.19	1.67	3.19	up	5.48	3.80	lnc-FUBP1-3	LNCipedia lo	lnc-FUBP1-3	---	chr1	78004346	78004554	-
TC0100004051.oe.1	-2.30	-1.20	2.30	down	3.20	4.41	lnc-BARHL2-	LNCipedia lo	lnc-BARHL2-	---	chr1	90283497	90306059	-
TC0100004134.oe.1	2.28	1.19	2.28	up	11.66	10.47	lnc-SASS6-5	LNCipedia lo	lnc-SASS6-5	NONHSAT0(	chr1	99710825	99715659	-
TC0100004273.oe.1	3.36	1.75	3.36	up	8.35	6.60	lnc-BCL2L15	NONCODE (	lnc-BCL2L15	NONHSAT0(	chr1	113878168	113887459	-
TC0100004282.oe.1	2.06	1.04	2.06	up	3.73	2.69	lnc-BCAS2-1	LNCipedia lo	lnc-BCAS2-1	---	chr1	114558476	114558681	-
TC0100004330.oe.1	2.08	1.06	2.08	up	8.92	7.86	lnc-WARS2-	LNCipedia lo	lnc-WARS2-	NONHSAT0(	chr1	119219314	119220096	-
TC0100004353.oe.1	-2.68	-1.42	2.68	down	14.28	15.70	lnc-FCGR1B-	gene_id XLC	lnc-FCGR1B-	NONHSAT0(	chr1	121742259	121743636	-
TC0100004378.oe.1	-2.57	-1.36	2.57	down	4.48	5.84	lnc-PDZK1-1	LNCipedia lo	lnc-PDZK1-1	NONHSAT0(	chr1	145670985	145672848	-
TC0100004380.oe.1	-2.53	-1.34	2.53	down	4.12	5.46	lnc-PIAS3-1	LNCipedia lo	lnc-PIAS3-1	---	chr1	145796993	145798540	-
TC0100004389.oe.1	-2.11	-1.08	2.11	down	7.16	8.24	lnc-NBPF10-	LNCipedia lo	lnc-NBPF10-	---	chr1	146224791	146225057	-
TC0100004522.oe.1	-2.10	-1.07	2.10	down	3.57	4.64	lnc-C1orf43-	LNCipedia lo	lnc-C1orf43-	---	chr1	154239837	154240157	-
TC0100004569.oe.1	-2.17	-1.12	2.17	down	4.10	5.21	lnc-YY1AP1-	LNCipedia lo	lnc-YY1AP1-	---	chr1	155498854	155503759	-
TC0100004604.oe.1	2.20	1.14	2.20	up	8.31	7.17	NONHSAG0	LNCipedia lo	lnc-NES-1:1	NONHSAT0(	chr1	156646507	156661424	-
TC0100004633.oe.1	-3.16	-1.66	3.16	down	2.46	4.12	lnc-OR10X1	LNCipedia lo	lnc-OR10X1	---	chr1	158594664	158594871	-
TC0100004725.oe.1	2.33	1.22	2.33	up	3.81	2.59	RP11-276E1	LNCipedia lo	lnc-FAM78B	NONHSAT0(	chr1	166475772	166490039	-
TC0100004758.oe.1	8.10	3.02	8.10	up	7.57	4.55	lnc-SELE-1	LNCipedia lo	lnc-SELE-1:2	NONHSAT0(	chr1	169691681	169708456	-
TC0100004800.oe.1	-2.35	-1.23	2.35	down	5.14	6.37	lnc-RC3H1-2	LNCipedia lo	lnc-RC3H1-2	NONHSAT0(	chr1	173931214	173936175	-
TC0100004818.oe.1	-2.01	-1.01	2.01	down	3.17	4.18	OTTHUMG0(	LNCipedia lo	lnc-RFWD2-	NONHSAT0(	chr1	175538775	175556818	-
TC0100004823.oe.1	2.07	1.05	2.07	up	11.00	9.95	lnc-TNR-6	NONCODE (	lnc-TNR-6:1	NONHSAT0(	chr1	175944831	176081256	-
TC0100004927.oe.1	5.49	2.46	5.49	up	7.06	4.60	lnc-PDC-2	NONCODE (	lnc-PDC-2:1	NONHSAT0(	chr1	186678270	186680423	-
TC0100004954.oe.1	-2.10	-1.07	2.10	down	3.24	4.31	lnc-UCHL5-1	LNCipedia lo	lnc-UCHL5-1	---	chr1	191950869	191955052	-
TC0100004961.oe.1	-2.13	-1.09	2.13	down	4.45	5.54	lnc-GLRX2-1	LNCipedia lo	lnc-GLRX2-1	---	chr1	193090866	193091556	-
TC0100005027.oe.1	2.34	1.22	2.34	up	9.56	8.34	lnc-SYT2-2	LNCipedia lo	lnc-SYT2-2:1	NONHSAT0(	chr1	202745877	202746595	-
TC0100005072.oe.1	-2.94	-1.56	2.94	down	6.20	7.76	lnc-SLC45A3	LNCipedia lo	lnc-SLC45A3	NONHSAT0(	chr1	205607945	205609670	-
TC0100005073.oe.1	-2.06	-1.04	2.06	down	7.85	8.89	lnc-SLC45A3	LNCipedia lo	lnc-SLC45A3	NONHSAT0(	chr1	205609716	205612016	-
TC0100005081.oe.1	2.26	1.18	2.26	up	6.15	4.97	lnc-AVPR1B-	LNCipedia lo	lnc-AVPR1B-	NONHSAT0(	chr1	206012331	206014130	-
TC0100005133.oe.1	-2.35	-1.23	2.35	down	3.02	4.25	lnc-C1orf13	LNCipedia lo	lnc-C1orf13	---	chr1	210040660	210040860	-
TC0100005214.oe.1	3.94	1.98	3.94	up	4.91	2.93	lnc-SLC30A1	NONCODE l	lnc-SLC30A1	NONHSAT0(	chr1	219080975	219173961	-
TC0100005254.oe.1	2.95	1.56	2.95	up	6.56	4.99	lnc-TAF1A-2	LNCipedia lo	lnc-TAF1A-2	NONHSAT0(	chr1	222538729	222540103	-
TC0100005255.oe.1	2.01	1.01	2.01	up	8.88	7.87	lnc-HHIPL2-	LNCipedia lo	lnc-HHIPL2-	NONHSAT0(	chr1	222568829	222570563	-
TC0100005328.oe.1	2.13	1.09	2.13	up	9.25	8.15	lnc-WNT9A-	NONCODE (	lnc-WNT9A-	NONHSAT0(	chr1	227733619	227734371	-
TC0100005439.oe.1	-2.69	-1.43	2.69	down	6.06	7.49	lnc-RBM34-	NONCODE (	lnc-RBM34-	NONHSAT0:	chr1	235172647	235175643	-
TC0100005497.oe.1	-3.36	-1.75	3.36	down	7.11	8.85	lnc-CHML-1	LNCipedia lo	lnc-CHML-1	NONHSAT0:	chr1	241628853	241631422	-
TC0100005536.oe.1	-2.04	-1.03	2.04	down	5.71	6.74	lnc-HNRNPU	NONCODE (	lnc-HNRNPU	NONHSAT0:	chr1	244840243	244855497	-
TC0100005559.oe.1	-2.36	-1.24	2.36	down	5.35	6.59	lnc-AHCTF1	gene_id XLC	lnc-AHCTF1	NONHSAT0:	chr1	246785650	246786104	-
TC0100005568.oe.1	-2.04	-1.03	2.04	down	4.80	5.83	NONHSAG0	NONCODE (	lnc-ZNF124-	NONHSAT0:	chr1	247187281	247188526	-
TC01001053.hg.4	2.05	1.04	2.05	up	4.46	3.43	EMBP1	embigin pse	---	---	chr1	121519112	121571888	+
TC01001487.hg.4	2.11	1.08	2.11	up	4.06	2.99	FMO6P	flavin contai	---	---	chr1	171137740	171161568	+
TC01001517.hg.4	-2.28	-1.19	2.28	down	3.67	4.86	GAS5-AS1	GAS5 antiser	---	---	chr1	173862152	173863941	+
TC01003095.hg.4	-2.70	-1.43	2.70	down	3.66	5.09	PFN1P2	profilin 1 pse	---	---	chr1	149084551	149086462	+
TC01006390.hg.4	-4.64	-2.22	4.64	down	6.49	8.71	HNRNPU-AS	non-protein	---	---	chr1	244840638	244846941	-
TC01006409.hg.4	-2.04	-1.03	2.04	down	3.78	4.81	C1ORF220//	chromosome	---	---	chr1	178542752	178548889	+
TC01006415.hg.4	-2.09	-1.06	2.09	down	3.34	4.40	RP11-267N1	regulator of	---	---	chr1	163244505	163321894	-
TC0200000007.oe.1	-2.87	-1.52	2.87	down	3.27	4.80	lnc-ACP1-1	LNCipedia lo	lnc-ACP1-1	---	chr2	307912	332118	+

TC0200000133.oe.1	2.04	1.03	2.04	up	6.72	5.68	lnc-C2orf50-	LNCipedia lc	lnc-C2orf50-	NONHSAT0	chr2	11172012	11178168	+
TC0200000210.oe.1	-3.18	-1.67	3.18	down	3.16	4.83	lnc-KCNS3-:	gene_id XLC	lnc-KCNS3-:	NONHSAT0	chr2	18550772	18553219	+
TC0200000212.oe.1	2.10	1.07	2.10	up	4.00	2.94	lnc-KCNS3-(	LNCipedia lc	lnc-KCNS3-(	---	chr2	18648288	18651233	+
TC0200000355.oe.1	-2.05	-1.04	2.05	down	3.12	4.16	lnc-LBH-4	LNCipedia lc	lnc-LBH-4:1	---	chr2	30200163	30205935	+
TC0200000495.oe.1	-2.30	-1.20	2.30	down	4.60	5.81	lnc-SLC3A1-	LNCipedia lc	lnc-SLC3A1-	NONHSAT0	chr2	44395310	44396393	+
TC0200000511.oe.1	-2.65	-1.41	2.65	down	3.00	4.41	lnc-RHOQ-3	LNCipedia lc	lnc-RHOQ-3	---	chr2	46522374	46524282	+
TC0200000577.oe.1	-2.76	-1.46	2.76	down	2.74	4.20	lnc-C2orf73-	LNCipedia lc	lnc-C2orf73-	---	chr2	54516636	54517678	+
TC0200000610.oe.1	2.01	1.01	2.01	up	11.96	10.96	lnc-VRK2-8	NONCODE (	lnc-VRK2-8:	NONHSAT0	chr2	58046642	58159920	+
TC0200000659.oe.1	-2.03	-1.02	2.03	down	3.89	4.91	lnc-OTX1-7	LNCipedia lc	lnc-OTX1-7:	NONHSAT0	chr2	62766549	62766968	+
TC0200000731.oe.1	2.06	1.05	2.06	up	7.73	6.68	lnc-PNO1-1	LNCipedia lc	lnc-PNO1-1	---	chr2	68179833	68180532	+
TC0200000750.oe.1	2.30	1.20	2.30	up	5.37	4.17	lnc-SNRNP2	LNCipedia lc	lnc-SNRNP2	NONHSAT0	chr2	69938152	69942942	+
TC0200000787.oe.1	-2.55	-1.35	2.55	down	5.65	7.00	lnc-DYSF-8	NONCODE (	lnc-DYSF-8:	NONHSAT0	chr2	71379516	71398757	+
TC0200000788.oe.1	-2.10	-1.07	2.10	down	9.96	11.03	lnc-DYSF-7	NONCODE (	lnc-DYSF-7:	NONHSAT0	chr2	71403850	71426606	+
TC0200000859.oe.1	-2.90	-1.54	2.90	down	5.08	6.62	RP11-342K6	LNCipedia lc	lnc-POLE4-5	NONHSAT0	chr2	75660462	75662208	+
TC0200000951.oe.1	2.13	1.09	2.13	up	10.91	9.81	lnc-PLGLB2-	LNCipedia lc	lnc-PLGLB2-	NONHSAT0	chr2	87704443	87714232	+
TC0200000967.oe.1	-2.02	-1.02	2.02	down	4.95	5.97	lnc-RPIA-1	NONCODE (	lnc-RPIA-1:1	NONHSAT0	chr2	88765807	88806612	+
TC0200000993.oe.1	2.19	1.13	2.19	up	3.64	2.51	CH17-132F2	N/A	---	---	chr2	90365737	90367699	+
TC0200001005.oe.1	-2.05	-1.04	2.05	down	4.20	5.24	lnc-AC07341	LNCipedia lc	lnc-AC07341	NONHSAT0	chr2	92130438	92136243	+
TC0200001131.oe.1	2.06	1.05	2.06	up	8.58	7.53	lnc-TMEM18	LNCipedia lc	lnc-TMEM18	NONHSAT0	chr2	102689694	102702451	+
TC0200001200.oe.1	-2.50	-1.32	2.50	down	5.34	6.66	lnc-LIMS1-4	NONCODE (	lnc-LIMS1-4	NONHSAT0	chr2	108471814	108475161	+
TC0200001278.oe.1	2.21	1.15	2.21	up	5.55	4.41	lnc-PSD4-2	LNCipedia lc	lnc-PSD4-2:	NONHSAT0	chr2	113205926	113209396	+
TC0200001346.oe.1	2.04	1.03	2.04	up	4.30	3.27	lnc-GLI2-6	NONCODE (	lnc-GLI2-6:1	NONHSAT0	chr2	120735623	120951763	+
TC0200001435.oe.1	2.23	1.16	2.23	up	7.46	6.30	lnc-PTPN18-	LNCipedia lc	lnc-PTPN18-	NONHSAT0	chr2	130342897	130345020	+
TC0200001470.oe.1	-2.07	-1.05	2.07	down	12.17	13.22	lnc-GPR39-1	LNCipedia lc	lnc-GPR39-1	NONHSAT0	chr2	132255493	132255912	+
TC0200001516.oe.1	-2.06	-1.04	2.06	down	2.67	3.72	lnc-THSD7B	LNCipedia lc	lnc-THSD7B	---	chr2	137693347	137693561	+
TC0200001544.oe.1	3.49	1.80	3.49	up	12.94	11.13	lnc-ARHGAF	NONCODE (	lnc-ARHGAF	NONHSAT0	chr2	142918679	142958075	+
TC0200001574.oe.1	2.16	1.11	2.16	up	3.99	2.88	lnc-ACVR2A	NONCODE (	lnc-ACVR2A	NONHSAT0	chr2	146477427	146478113	+
TC0200001586.oe.1	-2.34	-1.23	2.34	down	4.24	5.46	RP11-567F1	N/A	---	---	chr2	148062154	148062577	+
TC0200001740.oe.1	-2.97	-1.57	2.97	down	5.03	6.60	lnc-PHOSPH	LNCipedia lc	lnc-PHOSPH	NONHSAT0	chr2	169639482	169641403	+
TC0200001795.oe.1	-2.62	-1.39	2.62	down	4.42	5.81	RP11-394I13	LNCipedia lc	lnc-SP9-7:1	NONHSAT0	chr2	173968351	173969418	+
TC0200001798.oe.1	-2.16	-1.11	2.16	down	3.14	4.25	lnc-SP9-5	LNCipedia lc	lnc-SP9-5:1	---	chr2	174195610	174196656	+
TC0200001847.oe.1	2.36	1.24	2.36	up	6.66	5.42	lnc-OSBPL6-	NONCODE (	lnc-OSBPL6-	NONHSAT0	chr2	178121004	178124290	+
TC0200001876.oe.1	2.42	1.28	2.42	up	3.88	2.61	lnc-PLEKHA3	NONCODE (	lnc-PLEKHA3	NONHSAT0	chr2	179657779	179660393	+
TC0200001944.oe.1	2.01	1.01	2.01	up	5.67	4.66	lnc-MFSD6-	NONCODE (	lnc-MFSD6-	NONHSAT0	chr2	190343930	190366730	+
TC0200001956.oe.1	-2.04	-1.03	2.04	down	5.76	6.79	lnc-OBFC2A	NONCODE (	lnc-OBFC2A	NONHSAT0	chr2	191407658	191409073	+
TC0200002074.oe.1	3.25	1.70	3.25	up	5.25	3.55	lnc-ALS2CR8	NONCODE (	lnc-ALS2CR8	NONHSAT0	chr2	203083499	203085008	+
TC0200002125.oe.1	2.01	1.01	2.01	up	11.25	10.24	lnc-CREB1-6	NONCODE (	lnc-CREB1-6	NONHSAT0	chr2	207750685	207753875	+
TC0200002135.oe.1	2.97	1.57	2.97	up	6.85	5.29	lnc-UNC80-	LNCipedia lc	lnc-UNC80-	NONHSAT0	chr2	209661465	209710812	+
TC0200002202.oe.1	-2.37	-1.25	2.37	down	2.97	4.22	lnc-AC00755	LNCipedia lc	lnc-AC00755	NONHSAT0	chr2	216821131	216831633	+
TC0200002307.oe.1	-5.39	-2.43	5.39	down	7.15	9.58	lnc-KCNE4-1	LNCipedia lc	lnc-KCNE4-1	NONHSAT0	chr2	222942942	222944636	+
TC0200002328.oe.1	-2.18	-1.12	2.18	down	3.59	4.72	NONHSAG0	NONCODE (	lnc-RHBDD1	NONHSAT0	chr2	226804036	226805061	+
TC0200002337.oe.1	2.03	1.02	2.03	up	9.42	8.40	lnc-COL4A3	LNCipedia lc	lnc-COL4A3	NONHSAT0	chr2	227355298	227357181	+
TC0200002378.oe.1	2.90	1.54	2.90	up	5.99	4.45	lnc-PSMD1-	LNCipedia lc	lnc-PSMD1-	NONHSAT0	chr2	231258965	231278427	+
TC0200002771.oe.1	2.07	1.05	2.07	up	4.53	3.49	lnc-RDH14-	NONCODE (	lnc-RDH14-	NONHSAT0	chr2	18547386	18548204	-
TC0200002831.oe.1	2.03	1.02	2.03	up	7.19	6.17	lnc-TP53I3-2	LNCipedia lc	lnc-TP53I3-2	NONHSAT0	chr2	24073649	24076373	-
TC0200002936.oe.1	2.42	1.27	2.42	up	4.82	3.55	lnc-CAPN14	LNCipedia lc	lnc-CAPN14	NONHSAT0	chr2	31383082	31384395	-
TC0200003023.oe.1	2.43	1.28	2.43	up	5.31	4.03	lnc-CDKL4-6	LNCipedia lc	lnc-CDKL4-6	---	chr2	39323328	39323804	-
TC0200003146.oe.1	-2.15	-1.10	2.15	down	2.87	3.97	lnc-GPR75-5	LNCipedia lc	lnc-GPR75-5	---	chr2	53616465	53618384	-
TC0200003413.oe.1	2.04	1.03	2.04	up	8.33	7.30	lnc-SLC4A5-	LNCipedia lc	lnc-SLC4A5-	NONHSAT0	chr2	74361155	74369395	-

TC0200003458.oe.1	-2.48	-1.31	2.48	down	2.77	4.08	lnc-REG1B-4	gene_id XLC	lnc-REG1B-4	NONHSAT07	chr2	78597911	78599406	-
TC0200003631.oe.1	-3.42	-1.78	3.42	down	10.66	12.43	lnc-GPAT2-6	LNCipedia lc	lnc-GPAT2-6	NONHSAT07	chr2	95857340	95867471	-
TC0200003632.oe.1	-2.18	-1.12	2.18	down	5.34	6.46	lnc-GPAT2-5	LNCipedia lc	lnc-GPAT2-5	---	chr2	95891657	95896022	-
TC0200003633.oe.1	-2.02	-1.01	2.02	down	4.46	5.47	lnc-GPAT2-4	LNCipedia lc	lnc-GPAT2-4	NONHSAT07	chr2	95936811	95939208	-
TC0200003634.oe.1	-2.54	-1.35	2.54	down	7.58	8.92	lnc-GPAT2-1	LNCipedia lc	lnc-GPAT2-1	NONHSAT07	chr2	95945114	95960664	-
TC0200003635.oe.1	-2.07	-1.05	2.07	down	9.25	10.30	lnc-GPAT2-3	LNCipedia lc	lnc-GPAT2-3	NONHSAT07	chr2	95978127	95986839	-
TC0200003669.oe.1	-2.61	-1.38	2.61	down	4.29	5.68	NONHSAG0	LNCipedia lc	lnc-ACTR1B-	NONHSAT07	chr2	97416067	97433527	-
TC0200003670.oe.1	-2.22	-1.15	2.22	down	8.14	9.29	lnc-ACTR1B-	LNCipedia lc	lnc-ACTR1B-	NONHSAT07	chr2	97492514	97511249	-
TC0200003712.oe.1	2.16	1.11	2.16	up	4.28	3.17	lnc-RFX8-1	gene_id XLC	lnc-RFX8-1:1	NONHSAT07	chr2	101551603	101567908	-
TC0200003823.oe.1	2.22	1.15	2.22	up	8.04	6.90	NONHSAG0	NONCODE c	lnc-AC10846	NONHSAT07	chr2	111491943	111494811	-
TC0200003843.oe.1	4.51	2.17	4.51	up	6.41	4.24	lnc-IL1A-3	LNCipedia lc	lnc-IL1A-3:1	NONHSAT07	chr2	112835181	112836779	-
TC0200003860.oe.1	-2.49	-1.32	2.49	down	6.77	8.09	lnc-AC01674	NONCODE c	lnc-AC01674	NONHSAT07	chr2	113706793	113710239	-
TC0200004024.oe.1	2.36	1.24	2.36	up	5.89	4.65	RP11-725P1	LNCipedia lc	lnc-ANKRD3	---	chr2	132345616	132347297	-
TC0200004066.oe.1	12.06	3.59	12.06	up	7.94	4.35	lnc-LRP1B-4	NONCODE c	lnc-LRP1B-4	NONHSAT07	chr2	142870588	142871067	-
TC0200004139.oe.1	-2.59	-1.37	2.59	down	4.99	6.37	lnc-AC02346	NONCODE c	lnc-AC02346	NONHSAT07	chr2	150955591	150956046	-
TC0200004217.oe.1	-2.30	-1.20	2.30	down	3.01	4.21	lnc-RBMS1-	LNCipedia lc	lnc-RBMS1-	NONHSAT07	chr2	161048606	161050546	-
TC0200004250.oe.1	-2.19	-1.13	2.19	down	3.08	4.21	lnc-COBLL1-	NONCODE c	lnc-COBLL1-	NONHSAT07	chr2	164898528	164939503	-
TC0200004334.oe.1	-2.16	-1.11	2.16	down	7.21	8.31	lnc-OLA1-5	LNCipedia lc	lnc-OLA1-5:	NONHSAT07	chr2	173908532	173916832	-
TC0200004364.oe.1	-2.12	-1.09	2.12	down	7.21	8.30	lnc-CHN1-5	NONCODE c	lnc-CHN1-5	NONHSAT07	chr2	175072252	175074044	-
TC0200004413.oe.1	-2.72	-1.44	2.72	down	5.14	6.58	lnc-CCDC14	LNCipedia lc	lnc-CCDC14	NONHSAT07	chr2	179102280	179104484	-
TC0200004433.oe.1	2.01	1.00	2.01	up	3.48	2.47	lnc-NEUROD	LNCipedia lc	lnc-NEUROD	NONHSAT07	chr2	182140483	182141627	-
TC0200004512.oe.1	-2.09	-1.06	2.09	down	5.10	6.16	lnc-C2orf66-	LNCipedia lc	lnc-C2orf66-	NONHSAT07	chr2	196837871	196842825	-
TC0200004522.oe.1	-2.16	-1.11	2.16	down	10.77	11.88	lnc-ANKRD4	LNCipedia lc	lnc-ANKRD4	NONHSAT07	chr2	197392255	197393351	-
TC0200004524.oe.1	-2.04	-1.03	2.04	down	8.81	9.84	lnc-HSPD1-1	LNCipedia lc	lnc-HSPD1-1	NONHSAT07	chr2	197409783	197435047	-
TC0200004619.oe.1	-2.23	-1.16	2.23	down	3.18	4.34	lnc-METTL21	LNCipedia lc	lnc-METTL21	NONHSAT07	chr2	207673744	207673959	-
TC0200004621.oe.1	3.00	1.58	3.00	up	5.67	4.09	OTTHUMG0	NONCODE c	lnc-PLEKHM	NONHSAT07	chr2	207821290	207822769	-
TC0200004872.oe.1	-2.33	-1.22	2.33	down	5.19	6.41	lnc-AC11272	LNCipedia lc	lnc-AC11272	NONHSAT07	chr2	237338940	237340602	-
TC0200004907.oe.1	-2.05	-1.04	2.05	down	6.18	7.21	lnc-AC01702	LNCipedia lc	lnc-AC01702	NONHSAT07	chr2	239345520	239346621	-
TC02000912.hg.4	-2.46	-1.30	2.46	down	4.32	5.62	TEX41//LOC	uncharacteri	---	---	chr2	144667967	145182649	+
TC0300000020.oe.1	-2.82	-1.50	2.82	down	5.46	6.96	lnc-TRNT1-1	LNCipedia lc	lnc-TRNT1-1	NONHSAT08	chr3	3149140	3154190	+
TC0300000058.oe.1	-2.10	-1.07	2.10	down	5.03	6.10	lnc-THUMPD	LNCipedia lc	lnc-THUMPD	---	chr3	9405780	9407229	+
TC0300000059.oe.1	-2.00	-1.00	2.00	down	6.08	7.09	lnc-THUMPD	LNCipedia lc	lnc-THUMPD	---	chr3	9408357	9408669	+
TC0300000062.oe.1	-2.40	-1.27	2.40	down	5.20	6.47	lnc-THUMPD	LNCipedia lc	lnc-THUMPD	---	chr3	9443472	9443729	+
TC0300000087.oe.1	-2.05	-1.04	2.05	down	6.42	7.46	RP11-1020A	LNCipedia lc	lnc-CRELD1-	NONHSAT08	chr3	9958717	9962539	+
TC0300000088.oe.1	2.23	1.16	2.23	up	8.81	7.65	lnc-FANCD2	NONCODE c	lnc-FANCD2	NONHSAT08	chr3	9986893	10007003	+
TC0300000158.oe.1	-2.00	-1.00	2.00	down	2.48	3.48	lnc-BTD-3	LNCipedia lc	lnc-BTD-3:1	NONHSAT08	chr3	15740220	15762186	+
TC0300000194.oe.1	-2.92	-1.54	2.92	down	3.16	4.71	lnc-KAT2B-5	LNCipedia lc	lnc-KAT2B-5	---	chr3	20161384	20169413	+
TC0300000201.oe.1	-2.15	-1.11	2.15	down	4.77	5.88	lnc-KAT2B-1	LNCipedia lc	lnc-KAT2B-1	NONHSAT08	chr3	20969528	20970379	+
TC0300000353.oe.1	3.08	1.62	3.08	up	7.77	6.15	lnc-SLC25A3	NONCODE c	lnc-SLC25A3	NONHSAT08	chr3	39406718	39408195	+
TC0300000424.oe.1	-2.03	-1.02	2.03	down	2.84	3.86	lnc-ZNF35-4	LNCipedia lc	lnc-ZNF35-4	NONHSAT08	chr3	44682202	44684044	+
TC0300000495.oe.1	2.62	1.39	2.62	up	5.50	4.11	lnc-CCDC36	LNCipedia lc	lnc-CCDC36	NONHSAT08	chr3	49174933	49176402	+
TC0300000532.oe.1	2.43	1.28	2.43	up	5.77	4.50	lnc-RAD54L2	NONCODE c	lnc-RAD54L2	NONHSAT08	chr3	51541144	51541870	+
TC0300000576.oe.1	2.11	1.08	2.11	up	4.80	3.72	lnc-IL17RB-2	NONCODE c	lnc-IL17RB-2	NONHSAT09	chr3	54272774	54320541	+
TC0300000625.oe.1	-2.23	-1.16	2.23	down	3.09	4.25	lnc-PTPRG-4	NONCODE c	lnc-PTPRG-4	NONHSAT09	chr3	61409560	61413179	+
TC0300000685.oe.1	-2.00	-1.00	2.00	down	2.85	3.85	lnc-MITF-12	LNCipedia lc	lnc-MITF-12	NONHSAT09	chr3	70751123	70751350	+
TC0300000787.oe.1	-2.03	-1.02	2.03	down	8.05	9.07	lnc-EPHA3-2	NONCODE c	lnc-EPHA3-2	NONHSAT09	chr3	90414967	90427984	+
TC0300000795.oe.1	-2.67	-1.42	2.67	down	13.11	14.53	lnc-EPHA6-1	NONCODE c	lnc-EPHA6-1	NONHSAT09	chr3	96617185	96618195	+
TC0300000838.oe.1	3.17	1.66	3.17	up	6.05	4.39	lnc-GPR128-	LNCipedia lc	lnc-GPR128-	NONHSAT09	chr3	100519239	100556972	+
TC0300000839.oe.1	2.62	1.39	2.62	up	4.84	3.45	lnc-GPR128-	NONCODE c	lnc-GPR128-	NONHSAT09	chr3	100571030	100577065	+

TC0300000956.oe.1	2.04	1.03	2.04	up	7.30	6.27	lnc-ZDHHC2	NONCODE (	lnc-ZDHHC2	NONHSAT0!	chr3	114056740	114070806	+
TC0300001035.oe.1	2.23	1.16	2.23	up	4.19	3.03	lnc-SLC15A2	LNCipedia lc	lnc-SLC15A2	NONHSAT0!	chr3	121835209	121837776	+
TC0300001188.oe.1	-2.13	-1.09	2.13	down	3.21	4.30	lnc-SRPRB-3	LNCipedia lc	lnc-SRPRB-3	NONHSAT0!	chr3	133760300	133762363	+
TC0300001355.oe.1	3.10	1.63	3.10	up	7.24	5.61	lnc-AADACL	NONCODE (	lnc-AADACL	NONHSAT0!	chr3	151770428	151784894	+
TC0300001356.oe.1	8.66	3.11	8.66	up	9.72	6.61	NONHSAG0	NONCODE (	lnc-AADAC-	NONHSAT0!	chr3	151797484	151808249	+
TC0300001364.oe.1	-2.37	-1.24	2.37	down	9.04	10.28	lnc-MBNL1-	LNCipedia lc	lnc-MBNL1-	NONHSAT0!	chr3	152457691	152465780	+
TC0300001400.oe.1	-2.12	-1.08	2.12	down	4.35	5.43	lnc-MME-6	LNCipedia lc	lnc-MME-6:	NONHSAT0!	chr3	155277709	155283378	+
TC0300001516.oe.1	-2.64	-1.40	2.64	down	9.84	11.23	lnc-SKIL-4	LNCipedia lc	lnc-SKIL-4:1	NONHSAT0!	chr3	170394617	170397227	+
TC0300001544.oe.1	2.16	1.11	2.16	up	3.75	2.64	lnc-FNDC3B	LNCipedia lc	lnc-FNDC3B	NONHSAT0!	chr3	172522228	172522444	+
TC0300001659.oe.1	2.29	1.20	2.29	up	11.25	10.05	lnc-FAM131	LNCipedia lc	lnc-FAM131	NONHSAT0!	chr3	184328669	184331853	+
TC0300001678.oe.1	-2.04	-1.03	2.04	down	5.23	6.26	lnc-MAP3K1	LNCipedia lc	lnc-MAP3K1	NONHSAT0!	chr3	184982418	184996501	+
TC0300001680.oe.1	2.31	1.21	2.31	up	5.82	4.62	lnc-SENP2-1	LNCipedia lc	lnc-SENP2-1	NONHSAT0!	chr3	185443475	185447957	+
TC0300001767.oe.1	-2.34	-1.22	2.34	down	6.12	7.34	lnc-HES1-6	LNCipedia lc	lnc-HES1-6:	NONHSAT0!	chr3	194145674	194145967	+
TC0300001883.oe.1	2.63	1.39	2.63	up	6.01	4.62	lnc-AC01881	NONCODE (	lnc-AC01881	NONHSAT0!	chr3	4814294	4887293	-
TC0300001939.oe.1	2.09	1.06	2.09	up	14.45	13.39	lnc-GHRL-1	LNCipedia lc	lnc-GHRL-1:	NONHSAT0!	chr3	10300932	10301332	-
TC0300002063.oe.1	-2.15	-1.10	2.15	down	5.09	6.19	lnc-TOP2B-!	LNCipedia lc	lnc-TOP2B-!	---	chr3	25358112	25359922	-
TC0300002076.oe.1	-3.40	-1.76	3.40	down	3.53	5.30	lnc-SLC4A7-	LNCipedia lc	lnc-SLC4A7-	NONHSAT0!	chr3	27278688	27284901	-
TC0300002376.oe.1	3.55	1.83	3.55	up	10.89	9.06	lnc-ABHD14	NONCODE (	lnc-ABHD14	NONHSAT0!	chr3	51994935	51995895	-
TC0300002507.oe.1	-2.38	-1.25	2.38	down	4.78	6.03	lnc-LMOD3-	LNCipedia lc	lnc-LMOD3-	NONHSAT0!	chr3	69196719	69250248	-
TC0300002591.oe.1	3.05	1.61	3.05	up	4.84	3.23	lnc-STX19-1	NONCODE (	lnc-STX19-1	NONHSAT0!	chr3	93906040	93911157	-
TC0300002607.oe.1	3.46	1.79	3.46	up	11.45	9.65	lnc-CLDND1	NONCODE (	lnc-CLDND1	NONHSAT0!	chr3	98525061	98525274	-
TC0300002717.oe.1	-2.39	-1.26	2.39	down	5.84	7.10	lnc-ZBTB20-	NONCODE (	lnc-ZBTB20-	NONHSAT0!	chr3	114314501	114333920	-
TC0300002745.oe.1	-2.00	-1.00	2.00	down	4.27	5.27	lnc-POPDC2	LNCipedia lc	lnc-POPDC2	NONHSAT0!	chr3	119670764	119672338	-
TC0300002751.oe.1	-2.26	-1.18	2.26	down	7.67	8.84	lnc-GPR156-	LNCipedia lc	lnc-GPR156-	NONHSAT0!	chr3	120325331	120328350	-
TC0300002780.oe.1	-2.20	-1.14	2.20	down	5.82	6.96	lnc-PTPLB-4	LNCipedia lc	lnc-PTPLB-4	NONHSAT0!	chr3	123640379	123649064	-
TC0300002861.oe.1	2.29	1.20	2.29	up	7.88	6.68	lnc-PLXND1	LNCipedia lc	lnc-PLXND1	NONHSAT0!	chr3	129847048	129847957	-
TC0300002892.oe.1	-2.12	-1.09	2.12	down	7.79	8.87	lnc-RAB6B-2	NONCODE (	lnc-RAB6B-2	NONHSAT0!	chr3	133616821	133617526	-
TC0300002936.oe.1	-2.09	-1.06	2.09	down	5.59	6.65	lnc-CEP70-1	LNCipedia lc	lnc-CEP70-1	NONHSAT0!	chr3	138657283	138663933	-
TC0300003010.oe.1	2.58	1.37	2.58	up	4.48	3.11	lnc-ZIC4-2//	gene_id XLC	lnc-ZIC4-2:1	NONHSAT0!	chr3	147844123	147846657	-
TC0300003019.oe.1	-3.25	-1.70	3.25	down	12.02	13.72	lnc-CP-3	NONCODE (	lnc-CP-3:1	NONHSAT0!	chr3	149050270	149060800	-
TC0300003022.oe.1	-2.93	-1.55	2.93	down	12.92	14.47	lnc-HLTF-5	LNCipedia lc	lnc-HLTF-5:	NONHSAT0!	chr3	149173484	149176714	-
TC0300003023.oe.1	-2.14	-1.10	2.14	down	12.16	13.26	lnc-HLTF-6	LNCipedia lc	lnc-HLTF-6:2	NONHSAT0!	chr3	149185163	149188096	-
TC0300003037.oe.1	2.29	1.20	2.29	up	6.19	4.99	lnc-ANKUB1	NONCODE (	lnc-ANKUB1	NONHSAT0!	chr3	150017256	150022750	-
TC0300003098.oe.1	-3.08	-1.62	3.08	down	3.55	5.18	lnc-SSR3-5	LNCipedia lc	lnc-SSR3-5:	NONHSAT0!	chr3	156535066	156536901	-
TC0300003139.oe.1	-2.26	-1.18	2.26	down	4.48	5.66	lnc-TRIM59-	LNCipedia lc	lnc-TRIM59-	NONHSAT0!	chr3	160258403	160277478	-
TC0300003149.oe.1	-2.59	-1.37	2.59	down	3.63	5.00	lnc-B3GALN	LNCipedia lc	lnc-B3GALN	NONHSAT0!	chr3	161359299	161371517	-
TC0300003194.oe.1	-3.69	-1.88	3.69	down	5.95	7.83	lnc-LRRC31-	NONCODE (	lnc-LRRC31-	NONHSAT0!	chr3	170087584	170088850	-
TC0300003202.oe.1	2.45	1.29	2.45	up	10.50	9.20	lnc-EIF5A2-1	NONCODE (	lnc-EIF5A2-1	NONHSAT0!	chr3	170865794	170868181	-
TC0300003241.oe.1	2.34	1.22	2.34	up	4.06	2.84	lnc-TBL1XR1	LNCipedia lc	lnc-TBL1XR1	NONHSAT0!	chr3	176287186	176325154	-
TC0300003247.oe.1	-2.40	-1.26	2.40	down	9.40	10.66	lnc-ZMAT3-	LNCipedia lc	lnc-ZMAT3-	NONHSAT0!	chr3	177020754	177027830	-
TC0300003284.oe.1	2.24	1.16	2.24	up	4.35	3.19	lnc-DNAJC1	LNCipedia lc	lnc-DNAJC1	NONHSAT0!	chr3	181563539	181565659	-
TC0300003358.oe.1	-2.04	-1.03	2.04	down	12.06	13.08	lnc-MASP1-	LNCipedia lc	lnc-MASP1-	---	chr3	187413517	187413755	-
TC0300003379.oe.1	-2.13	-1.09	2.13	down	2.86	3.95	lnc-LEPREL1	LNCipedia lc	lnc-LEPREL1	---	chr3	189448461	189459100	-
TC0300003382.oe.1	2.05	1.03	2.05	up	6.37	5.34	lnc-TMEM2(	NONCODE (	lnc-TMEM2(	NONHSAT0!	chr3	190310169	190313218	-
TC0300003402.oe.1	-3.34	-1.74	3.34	down	6.13	7.86	lnc-ATP13A4	NONCODE l	lnc-ATP13A4	NONHSAT0!	chr3	193840828	193844024	-
TC0300003410.oe.1	-2.02	-1.01	2.02	down	8.41	9.42	lnc-GP5-1	LNCipedia lc	lnc-GP5-1:1	NONHSAT0!	chr3	194403465	194413063	-
TC03000749.hg.4	-2.23	-1.16	2.23	down	3.27	4.43	BPESC1	blepharophi	---	---	chr3	139104185	139125167	+
TC03001476.hg.4	-2.38	-1.25	2.38	down	2.31	3.56	ESRG	embryonic s	---	---	chr3	54632124	54639857	-
TC0400000003.oe.1	-2.27	-1.18	2.27	down	4.49	5.67	CH17-262A2	N/A	---	---	chr4	149738	150317	+

TC0400000137.oe.1	2.34	1.23	2.34	up	8.49	7.27	NONHSAG0	NONCODE (	lnc-SORCS2	NONHSAT09	chr4	7754090	7778928	+
TC0400000283.oe.1	-2.37	-1.24	2.37	down	8.76	10.00	lnc-RBPJ-2	LNCipedia lc	lnc-RBPJ-2:1	NONHSAT09	chr4	26432993	26434919	+
TC0400000284.oe.1	-2.11	-1.08	2.11	down	4.67	5.74	lnc-RBPJ-3	LNCipedia lc	lnc-RBPJ-3:1	---	chr4	26466833	26468033	+
TC0400000424.oe.1	2.28	1.19	2.28	up	5.70	4.51	lnc-GABRB1	LNCipedia lc	lnc-GABRB1	NONHSAT09	chr4	47485297	47512846	+
TC0400000459.oe.1	2.00	1.00	2.00	up	9.29	8.29	lnc-RASL11E	LNCipedia lc	lnc-RASL11E	NONHSAT09	chr4	53442253	53453131	+
TC0400000475.oe.1	-2.20	-1.13	2.20	down	3.14	4.28	lnc-SRD5A3	NONCODE (	lnc-SRD5A3	NONHSAT09	chr4	55053060	55092534	+
TC0400000597.oe.1	-2.39	-1.26	2.39	down	4.51	5.77	lnc-AFP-2	LNCipedia lc	lnc-AFP-2:1	NONHSAT09	chr4	73418006	73419545	+
TC0400000598.oe.1	-2.02	-1.02	2.02	down	4.11	5.13	lnc-AFP-1	NONCODE (	lnc-AFP-1:1	NONHSAT09	chr4	73419281	73421219	+
TC0400000606.oe.1	5.68	2.51	5.68	up	6.74	4.23	lnc-CXCL6-2	LNCipedia lc	lnc-CXCL6-2	NONHSAT09	chr4	73740569	73741986	+
TC0400000607.oe.1	4.20	2.07	4.20	up	5.46	3.39	lnc-CXCL6-3	LNCipedia lc	lnc-CXCL6-3	NONHSAT09	chr4	73743139	73743351	+
TC0400000616.oe.1	2.58	1.36	2.58	up	9.46	8.10	lnc-EPGN-3	NONCODE (	lnc-EPGN-3:	NONHSAT09	chr4	74365173	74382013	+
TC0400000617.oe.1	3.26	1.71	3.26	up	10.20	8.49	lnc-AREG-1	NONCODE (	lnc-AREG-1:	NONHSAT09	chr4	74382423	74384952	+
TC0400000618.oe.1	2.92	1.55	2.92	up	6.57	5.02	lnc-EREG-1	NONCODE (	lnc-EREG-1:	NONHSAT09	chr4	74448639	74450532	+
TC0400000628.oe.1	2.07	1.05	2.07	up	6.49	5.44	lnc-C4orf26-	LNCipedia lc	lnc-C4orf26-	NONHSAT09	chr4	75539859	75546761	+
TC0400000662.oe.1	-3.66	-1.87	3.66	down	4.65	6.53	lnc-ANXA3-	LNCipedia lc	lnc-ANXA3-	NONHSAT09	chr4	78913230	78916363	+
TC0400000673.oe.1	-2.13	-1.09	2.13	down	3.03	4.12	lnc-PRDM8-	LNCipedia lc	lnc-PRDM8-	---	chr4	79819752	79842448	+
TC0400000694.oe.1	2.02	1.02	2.02	up	11.37	10.36	lnc-MRPS18	NONCODE (	lnc-MRPS18	NONHSAT09	chr4	83588258	83595335	+
TC0400000695.oe.1	2.32	1.21	2.32	up	13.13	11.91	lnc-MRPS18	NONCODE (	lnc-MRPS18	NONHSAT09	chr4	83598529	83605139	+
TC0400000863.oe.1	-2.06	-1.04	2.06	down	3.92	4.96	lnc-AP1AR-1	LNCipedia lc	lnc-AP1AR-1	NONHSAT09	chr4	112321704	112322667	+
TC0400000928.oe.1	-2.16	-1.11	2.16	down	7.40	8.51	lnc-MYOZ2-	LNCipedia lc	lnc-MYOZ2-	NONHSAT09	chr4	119267381	119273330	+
TC0400000929.oe.1	2.05	1.04	2.05	up	5.73	4.69	lnc-AC11037	LNCipedia lc	lnc-AC11037	NONHSAT09	chr4	119378132	119405615	+
TC0400000973.oe.1	-2.10	-1.07	2.10	down	3.65	4.72	lnc-SPRY1-5	LNCipedia lc	lnc-SPRY1-5	---	chr4	123709458	123716405	+
TC0400000993.oe.1	-2.04	-1.03	2.04	down	3.07	4.10	lnc-LARP1B-	LNCipedia lc	lnc-LARP1B-	---	chr4	128057217	128060178	+
TC0400001087.oe.1	2.40	1.26	2.40	up	4.41	3.15	lnc-SMARCA	LNCipedia lc	lnc-SMARCA	---	chr4	143601398	143615169	+
TC0400001163.oe.1	2.07	1.05	2.07	up	4.48	3.43	NONHSAG0	NONCODE (	lnc-ARFIP1-1	NONHSAT09	chr4	152337655	152338098	+
TC0400001191.oe.1	-2.18	-1.12	2.18	down	7.60	8.72	lnc-LRAT-2	LNCipedia lc	lnc-LRAT-2:	NONHSAT09	chr4	154563011	154566749	+
TC0400001277.oe.1	5.11	2.35	5.11	up	12.28	9.93	lnc-PALLD-5	NONCODE (	lnc-PALLD-5	NONHSAT09	chr4	168165241	168178315	+
TC0400001288.oe.1	2.04	1.03	2.04	up	6.91	5.88	lnc-PALLD-8	NONCODE (	lnc-PALLD-8	NONHSAT09	chr4	169721190	169723183	+
TC0400001335.oe.1	-2.03	-1.02	2.03	down	3.02	4.04	lnc-ADAM29	LNCipedia lc	lnc-ADAM29	---	chr4	174760024	174766808	+
TC0400001345.oe.1	-2.29	-1.19	2.29	down	6.99	8.18	lnc-SPCS3-3	LNCipedia lc	lnc-SPCS3-3	NONHSAT09	chr4	176296368	176296825	+
TC0400001498.oe.1	-2.03	-1.02	2.03	down	3.45	4.47	lnc-CPLX1-5	LNCipedia lc	lnc-CPLX1-5	---	chr4	759255	759618	-
TC0400001584.oe.1	2.17	1.12	2.17	up	5.41	4.29	lnc-EVC2-2	NONCODE (	lnc-EVC2-2:	NONHSAT09	chr4	5820784	5826861	-
TC0400001692.oe.1	-2.13	-1.09	2.13	down	5.32	6.41	lnc-LDB2-3	NONCODE (	lnc-LDB2-3:	NONHSAT09	chr4	17000658	17003611	-
TC0400001705.oe.1	-2.11	-1.07	2.11	down	4.90	5.98	lnc-DCAF16	LNCipedia lc	lnc-DCAF16	NONHSAT09	chr4	17884122	17884763	-
TC0400001804.oe.1	-2.33	-1.22	2.33	down	9.74	10.96	lnc-C4orf34-	LNCipedia lc	lnc-C4orf34-	NONHSAT09	chr4	39547301	39549586	-
TC0400001809.oe.1	-2.54	-1.34	2.54	down	10.05	11.39	lnc-C4orf34-	LNCipedia lc	lnc-C4orf34-	NONHSAT09	chr4	39824798	39825359	-
TC0400001810.oe.1	-3.08	-1.63	3.08	down	10.13	11.76	lnc-C4orf34-	NONCODE (	lnc-C4orf34-	NONHSAT09	chr4	39872792	39874400	-
TC0400001811.oe.1	-2.57	-1.36	2.57	down	10.30	11.67	lnc-C4orf34-	LNCipedia lc	lnc-C4orf34-	NONHSAT09	chr4	39910065	39913687	-
TC0400001906.oe.1	-2.46	-1.30	2.46	down	6.42	7.72	lnc-PDCL2-4	LNCipedia lc	lnc-PDCL2-4	NONHSAT09	chr4	55427907	55431424	-
TC0400001934.oe.1	-2.08	-1.06	2.08	down	3.11	4.17	NONHSAG0	LNCipedia lc	lnc-IGFBP7-	NONHSAT09	chr4	59047020	59075256	-
TC0400002005.oe.1	2.06	1.04	2.06	up	7.29	6.25	lnc-COX18-4	NONCODE (	lnc-COX18-4	NONHSAT09	chr4	73100861	73104080	-
TC0400002012.oe.1	5.32	2.41	5.32	up	9.22	6.81	lnc-CXCL5-1	LNCipedia lc	lnc-CXCL5-1	NONHSAT09	chr4	73995642	73996738	-
TC0400002120.oe.1	-2.26	-1.17	2.26	down	3.25	4.42	lnc-NKX6-1-	LNCipedia lc	lnc-NKX6-1-	---	chr4	84746003	84749139	-
TC0400002126.oe.1	2.16	1.11	2.16	up	6.45	5.34	lnc-SLC10A6	NONCODE (	lnc-SLC10A6	NONHSAT09	chr4	86095135	86101922	-
TC0400002141.oe.1	-2.10	-1.07	2.10	down	3.45	4.52	lnc-HSD17B	LNCipedia lc	lnc-HSD17B	NONHSAT09	chr4	87495008	87529209	-
TC0400002204.oe.1	-15.97	-4.00	15.97	down	7.98	11.98	lnc-ADH1B-	LNCipedia lc	lnc-ADH1B-	NONHSAT09	chr4	99344888	99352744	-
TC0400002230.oe.1	2.06	1.04	2.06	up	7.41	6.37	lnc-UBE2D3	LNCipedia lc	lnc-UBE2D3	NONHSAT09	chr4	102727274	102730721	-
TC0400002291.oe.1	2.04	1.03	2.04	up	8.39	7.36	lnc-CASP6-2	LNCipedia lc	lnc-CASP6-2	NONHSAT09	chr4	109717548	109718761	-
TC0400002309.oe.1	-2.04	-1.03	2.04	down	3.90	4.92	RP11-269F2	NONCODE (	lnc-TIFA-2:1	NONHSAT09	chr4	111826881	112072698	-

TC0400002344.oe.1	2.65	1.40	2.65	up	8.52	7.12	lnc-PRSS12-	NONCODE	lnc-PRSS12-	NONHSAT0	chr4	118738104	118739223	-
TC0400002494.oe.1	-2.48	-1.31	2.48	down	3.00	4.31	lnc-GYPE-1	NONCODE l	lnc-GYPE-1:	NONHSAT0	chr4	143817789	143829352	-
TC0400002509.oe.1	-2.77	-1.47	2.77	down	10.92	12.39	lnc-OTUD4-	LNCipedia l	lnc-OTUD4-	---	chr4	145399817	145400043	-
TC0400002520.oe.1	2.16	1.11	2.16	up	8.71	7.60	lnc-SLC10A7	LNCipedia l	lnc-SLC10A7	NONHSAT0	chr4	145870301	145892415	-
TC0400002521.oe.1	2.23	1.16	2.23	up	5.63	4.48	lnc-SLC10A7	NONCODE	lnc-SLC10A7	NONHSAT0	chr4	145876496	145885748	-
TC0400002586.oe.1	-2.25	-1.17	2.25	down	7.80	8.97	lnc-FGA-1	LNCipedia l	lnc-FGA-1:1	NONHSAT0	chr4	154608508	154609691	-
TC0400002616.oe.1	2.41	1.27	2.41	up	7.43	6.16	lnc-C4orf46	LNCipedia l	lnc-C4orf46	NONHSAT0	chr4	158130800	158173023	-
TC0400002645.oe.1	2.37	1.25	2.37	up	5.11	3.86	lnc-TMEM19	LNCipedia l	lnc-TMEM19	---	chr4	165070608	165071004	-
TC0400002654.oe.1	-2.20	-1.14	2.20	down	5.74	6.88	lnc-DDX60-	NONCODE	lnc-DDX60-	NONHSAT0	chr4	167922865	167925989	-
TC0400002656.oe.1	2.10	1.07	2.10	up	5.55	4.48	lnc-DDX60L	LNCipedia l	lnc-DDX60L	NONHSAT0	chr4	168236029	168237488	-
TC04000083.hg.4	2.01	1.01	2.01	up	7.39	6.38	AFAP1-AS1	AFAP1 antis	---	---	chr4	7754090	7778927	+
TC04001655.hg.4	2.16	1.11	2.16	up	9.72	8.61	ANXA2P1	annexin A2	---	---	chr4	153307469	153308811	-
TC0500000022.oe.1	-3.56	-1.83	3.56	down	8.05	9.88	lnc-EXOC3-	LNCipedia l	lnc-EXOC3-	NONHSAT0	chr5	649317	651579	+
TC0500000221.oe.1	2.41	1.27	2.41	up	11.03	9.76	lnc-FBXL7-5	LNCipedia l	lnc-FBXL7-5	NONHSAT1	chr5	15451996	15452553	+
TC0500000407.oe.1	-2.21	-1.14	2.21	down	5.57	6.72	lnc-ZNF131	NONCODE	lnc-ZNF131	NONHSAT1	chr5	43054729	43055305	+
TC0500000453.oe.1	-2.57	-1.36	2.57	down	3.78	5.14	lnc-ITGA1-4	LNCipedia l	lnc-ITGA1-4	NONHSAT1	chr5	52931152	52959207	+
TC0500000561.oe.1	-3.28	-1.71	3.28	down	9.10	10.81	lnc-SREK1-8	LNCipedia l	lnc-SREK1-8	NONHSAT1	chr5	66022872	66024373	+
TC0500000562.oe.1	-2.05	-1.04	2.05	down	7.43	8.47	lnc-SREK1-7	LNCipedia l	lnc-SREK1-7	NONHSAT1	chr5	66025306	66025977	+
TC0500000574.oe.1	2.00	1.00	2.00	up	5.67	4.67	lnc-SREK1-1	NONCODE	lnc-SREK1-1	NONHSAT1	chr5	67142164	67143020	+
TC0500000644.oe.1	-2.60	-1.38	2.60	down	4.46	5.84	lnc-FCHO2-	LNCipedia l	lnc-FCHO2-	NONHSAT1	chr5	72869728	72872332	+
TC0500000684.oe.1	-3.00	-1.59	3.00	down	5.30	6.89	lnc-ANKDD1	LNCipedia l	lnc-ANKDD1	NONHSAT1	chr5	75597114	75598040	+
TC0500000727.oe.1	2.22	1.15	2.22	up	7.99	6.84	lnc-BHMT-1	LNCipedia l	lnc-BHMT-1	NONHSAT1	chr5	79082392	79083198	+
TC0500000761.oe.1	-2.23	-1.16	2.23	down	2.86	4.01	lnc-ZCCHC9	LNCipedia l	lnc-ZCCHC9	NONHSAT1	chr5	81318174	81319269	+
TC0500000906.oe.1	-3.93	-1.97	3.93	down	5.25	7.22	lnc-CTD-22	NONCODE	lnc-CTD-22	NONHSAT1	chr5	97035882	97037510	+
TC0500000941.oe.1	-2.75	-1.46	2.75	down	7.45	8.91	lnc-C5orf30	LNCipedia l	lnc-C5orf30	NONHSAT1	chr5	103173254	103174191	+
TC0500000942.oe.1	-2.32	-1.21	2.32	down	3.77	4.99	lnc-C5orf30	NONCODE	lnc-C5orf30	NONHSAT1	chr5	103184243	103191058	+
TC0500000962.oe.1	-2.03	-1.02	2.03	down	5.70	6.73	lnc-MAN2A1	NONCODE	lnc-MAN2A1	NONHSAT1	chr5	108748399	108832820	+
TC0500001054.oe.1	-2.08	-1.06	2.08	down	4.72	5.78	lnc-TNFAIP8	LNCipedia l	lnc-TNFAIP8	NONHSAT1	chr5	119197758	119206892	+
TC0500001070.oe.1	-2.16	-1.11	2.16	down	7.85	8.96	lnc-SNX24-5	LNCipedia l	lnc-SNX24-5	NONHSAT1	chr5	122803494	122808711	+
TC0500001127.oe.1	-2.96	-1.57	2.96	down	9.21	10.78	lnc-SLC27A6	LNCipedia l	lnc-SLC27A6	NONHSAT1	chr5	128186798	128187086	+
TC0500001138.oe.1	-2.06	-1.04	2.06	down	5.88	6.93	lnc-LYRM7-	LNCipedia l	lnc-LYRM7-	NONHSAT1	chr5	131204459	131205427	+
TC0500001207.oe.1	2.92	1.54	2.92	up	4.92	3.37	lnc-CATSPEI	LNCipedia l	lnc-CATSPEI	NONHSAT1	chr5	135265437	135267895	+
TC0500001263.oe.1	-2.79	-1.48	2.79	down	3.86	5.34	lnc-PAIP2-2	LNCipedia l	lnc-PAIP2-2	---	chr5	139309812	139310433	+
TC0500001278.oe.1	-3.26	-1.71	3.26	down	4.53	6.23	lnc-PURA-2	NONCODE l	lnc-PURA-2:	NONHSAT1	chr5	140117258	140122167	+
TC0500001282.oe.1	-2.51	-1.33	2.51	down	3.50	4.83	lnc-IGIP-2//	NONCODE	lnc-IGIP-2:1	NONHSAT1	chr5	140200163	140203187	+
TC0500001365.oe.1	-2.60	-1.38	2.60	down	4.37	5.76	lnc-POU4F3	LNCipedia l	lnc-POU4F3	---	chr5	146463010	146463380	+
TC0500001390.oe.1	2.72	1.45	2.72	up	6.79	5.34	lnc-AFAP1L1	LNCipedia l	lnc-AFAP1L1	NONHSAT1	chr5	149183549	149200620	+
TC0500001392.oe.1	2.19	1.13	2.19	up	6.69	5.56	lnc-AFAP1L1	LNCipedia l	lnc-AFAP1L1	NONHSAT1	chr5	149252344	149258574	+
TC0500001420.oe.1	2.07	1.05	2.07	up	6.43	5.38	lnc-IRGM-3	LNCipedia l	lnc-IRGM-3:	NONHSAT1	chr5	151020592	151027490	+
TC0500001504.oe.1	-2.73	-1.45	2.73	down	2.85	4.30	lnc-PTTG1-5	NONCODE	lnc-PTTG1-5	NONHSAT1	chr5	160447681	160448341	+
TC0500001778.oe.1	2.28	1.19	2.28	up	8.43	7.24	lnc-ZDHHC1	LNCipedia l	lnc-ZDHHC1	NONHSAT0	chr5	767397	768931	-
TC0500001827.oe.1	2.06	1.04	2.06	up	4.62	3.58	lnc-MED10-	NONCODE	lnc-MED10-	NONHSAT1	chr5	5395972	5396335	-
TC0500002075.oe.1	-2.29	-1.20	2.29	down	3.29	4.48	lnc-PRLR-1	LNCipedia l	lnc-PRLR-1:	NONHSAT1	chr5	35318218	35318651	-
TC0500002096.oe.1	-2.46	-1.30	2.46	down	3.38	4.68	lnc-NUP155	LNCipedia l	lnc-NUP155	NONHSAT1	chr5	37169532	37176099	-
TC0500002097.oe.1	-2.03	-1.02	2.03	down	3.58	4.60	lnc-NUP155	LNCipedia l	lnc-NUP155	NONHSAT1	chr5	37246175	37246479	-
TC0500002113.oe.1	2.42	1.28	2.42	up	7.54	6.26	lnc-AC0914	NONCODE	lnc-AC0914	NONHSAT1	chr5	38485597	38489245	-
TC0500002122.oe.1	-2.17	-1.12	2.17	down	7.60	8.72	lnc-FYB-3	NONCODE	lnc-FYB-3:1	NONHSAT1	chr5	38962951	38971961	-
TC0500002123.oe.1	2.07	1.05	2.07	up	6.56	5.51	lnc-FYB-1	NONCODE l	lnc-FYB-1:1	NONHSAT1	chr5	39105224	39105755	-
TC0500002128.oe.1	2.04	1.03	2.04	up	8.59	7.56	lnc-FYB-2	LNCipedia l	lnc-FYB-2:1	NONHSAT1	chr5	39374745	39376042	-

TC0500002178.oe.1	-2.40	-1.26	2.40	down	3.09	4.36	lnc-EMB-6	LNCipedia lc	lnc-EMB-6:1	NONHSAT1(	chr5	50403256	50443248	-
TC0500002204.oe.1	2.94	1.56	2.94	up	7.56	6.00	lnc-RP11-45	uncharacteri	lnc-RP11-45	NONHSAT1(	chr5	54660158	54744258	-
TC0500002219.oe.1	-2.24	-1.16	2.24	down	10.01	11.17	lnc-ANKRD5	LNCipedia lc	lnc-ANKRD5	NONHSAT1(	chr5	55936653	55939493	-
TC0500002221.oe.1	-2.03	-1.02	2.03	down	11.03	12.05	lnc-ANKRD5	LNCipedia lc	lnc-ANKRD5	NONHSAT1(	chr5	55955980	55960480	-
TC0500002229.oe.1	-2.07	-1.05	2.07	down	10.49	11.54	lnc-CTC-23(	LNCipedia lc	lnc-CTC-23(	---	chr5	56488360	56493753	-
TC0500002244.oe.1	4.04	2.02	4.04	up	10.89	8.87	lnc-CTD-21:	NONCODE (	lnc-CTD-21:	NONHSAT1(	chr5	58453985	58460086	-
TC0500002245.oe.1	5.46	2.45	5.46	up	13.01	10.56	lnc-CTD-21:	NONCODE (	lnc-CTD-21:	NONHSAT1(	chr5	58454706	58455116	-
TC0500002336.oe.1	-3.31	-1.73	3.31	down	4.28	6.01	lnc-TAF9-9	LNCipedia lc	lnc-TAF9-9:	NONHSAT1(	chr5	70132532	70134832	-
TC0500002377.oe.1	2.24	1.16	2.24	up	6.43	5.26	lnc-ZNF366-	LNCipedia lc	lnc-ZNF366-	NONHSAT1(	chr5	73552531	73554430	-
TC0500002553.oe.1	-2.58	-1.37	2.58	down	6.88	8.25	lnc-AC02732	NONCODE (	lnc-AC02732	NONHSAT1(	chr5	91370798	91373845	-
TC0500002575.oe.1	-3.33	-1.74	3.33	down	5.61	7.34	lnc-SPATA9-	LNCipedia lc	lnc-SPATA9-	NONHSAT1(	chr5	95503850	95513000	-
TC0500002576.oe.1	-2.12	-1.08	2.12	down	6.83	7.92	lnc-SPATA9-	LNCipedia lc	lnc-SPATA9-	NONHSAT1(	chr5	95513132	95516768	-
TC0500002577.oe.1	-2.32	-1.22	2.32	down	6.28	7.50	lnc-SPATA9-	NONCODE (	lnc-SPATA9-	NONHSAT1(	chr5	95528013	95537107	-
TC0500002578.oe.1	-3.65	-1.87	3.65	down	8.16	10.02	lnc-SPATA9-	LNCipedia lc	lnc-SPATA9-	NONHSAT1(	chr5	95546974	95550757	-
TC0500002608.oe.1	-2.22	-1.15	2.22	down	7.21	8.36	lnc-RIOK2-6	LNCipedia lc	lnc-RIOK2-6	NONHSAT1(	chr5	98856425	98858664	-
TC0500002610.oe.1	-2.64	-1.40	2.64	down	7.39	8.79	lnc-RIOK2-8	NONCODE (	lnc-RIOK2-8	NONHSAT1(	chr5	98881247	98888186	-
TC0500002611.oe.1	-2.50	-1.32	2.50	down	5.33	6.65	lnc-RIOK2-9	LNCipedia lc	lnc-RIOK2-9	---	chr5	98896941	98897293	-
TC0500002613.oe.1	-3.01	-1.59	3.01	down	7.45	9.04	lnc-RIOK2-1	LNCipedia lc	lnc-RIOK2-1	NONHSAT1(	chr5	98901151	98926517	-
TC0500002705.oe.1	-2.25	-1.17	2.25	down	5.83	7.00	lnc-PGGT1B	NONCODE (	lnc-PGGT1B	NONHSAT1(	chr5	115275339	115296623	-
TC0500002861.oe.1	-2.41	-1.27	2.41	down	3.27	4.54	lnc-FSTL4-4	NONCODE (	lnc-FSTL4-4	NONHSAT1(	chr5	133720495	133720934	-
TC0500002877.oe.1	2.68	1.42	2.68	up	8.28	6.86	lnc-CTC-34!	LNCipedia lc	lnc-CTC-34!	NONHSAT1(	chr5	135029287	135032010	-
TC0500003031.oe.1	36.21	5.18	36.21	up	8.90	3.72	lnc-JAKMIP2	NONCODE (	lnc-JAKMIP2	NONHSAT1(	chr5	147824572	147828130	-
TC0500003141.oe.1	-2.26	-1.17	2.26	down	3.99	5.16	lnc-CLINT1-	NONCODE (	lnc-CLINT1-	NONHSAT1(	chr5	157945315	157951960	-
TC0500003297.oe.1	2.15	1.10	2.15	up	8.42	7.31	lnc-HK3-3	NONCODE (	lnc-HK3-3:1	NONHSAT1(	chr5	176975416	176980276	-
TC05000111.hg.4	2.21	1.14	2.21	up	3.61	2.46	PMCHL1	pro-melanin	---	---	chr5	22142352	22152555	+
TC05000127.hg.4	-2.36	-1.24	2.36	down	3.58	4.82	LSP1P3	lymphocyte-	---	---	chr5	28926870	28927313	+
TC05000462.hg.4	-2.44	-1.28	2.44	down	3.43	4.72	C5orf27	chromosome	---	---	chr5	95852232	95860133	+
TC05001554.hg.4	2.09	1.06	2.09	up	4.00	2.94	SCARNA18	small Cajal b	---	---	chr5	83064204	83064337	-
TC0600000025.oe.1	3.10	1.63	3.10	up	7.69	6.06	lnc-AL03338	NONCODE (	lnc-AL03338	NONHSAT1(	chr6	861046	863352	+
TC0600000140.oe.1	2.08	1.06	2.08	up	5.76	4.70	lnc-BPHL-1	LNCipedia lc	lnc-BPHL-1:	---	chr6	3156308	3157544	+
TC0600000205.oe.1	-2.09	-1.07	2.09	down	2.96	4.02	lnc-C6orf20:	NONCODE I	lnc-C6orf20:	NONHSAT1(	chr6	4312514	4328826	+
TC0600000261.oe.1	2.02	1.01	2.02	up	8.08	7.06	lnc-DSP-2	NONCODE (	lnc-DSP-2:1	NONHSAT1(	chr6	7506203	7506549	+
TC0600000379.oe.1	2.34	1.23	2.34	up	13.73	12.50	lnc-SOX4-2	LNCipedia lc	lnc-SOX4-2:	NONHSAT1(	chr6	21597430	21598619	+
TC0600000430.oe.1	2.25	1.17	2.25	up	5.92	4.76	lnc-HIST1H3	LNCipedia lc	lnc-HIST1H3	---	chr6	26022061	26022589	+
TC0600000537.oe.1	2.12	1.09	2.12	up	5.26	4.18	lnc-TRIM40-	NONCODE (	lnc-TRIM40-	NONHSAT1(	chr6	30170313	30171097	+
TC0600000691.oe.1	-2.33	-1.22	2.33	down	3.28	4.50	OTTHUMG0(	LNCipedia lc	lnc-KCTD20-	NONHSAT1:	chr6	36386831	36393462	+
TC0600000900.oe.1	-2.70	-1.43	2.70	down	4.78	6.21	lnc-BAG2-8	LNCipedia lc	lnc-BAG2-8:	NONHSAT1:	chr6	57160808	57168833	+
TC0600001011.oe.1	-2.34	-1.23	2.34	down	2.72	3.95	lnc-MYO6-8	LNCipedia lc	lnc-MYO6-8	---	chr6	76579170	76650342	+
TC0600001127.oe.1	-2.23	-1.16	2.23	down	6.05	7.21	lnc-GJA10-1	LNCipedia lc	lnc-GJA10-1	NONHSAT1:	chr6	90672453	90688912	+
TC0600001209.oe.1	2.96	1.56	2.96	up	6.18	4.61	lnc-AIM1-4	LNCipedia lc	lnc-AIM1-4:	NONHSAT1:	chr6	106088354	106096167	+
TC0600001290.oe.1	-2.29	-1.19	2.29	down	2.83	4.03	lnc-WISP3-2	NONCODE (	lnc-WISP3-2	NONHSAT1:	chr6	111873664	111876523	+
TC0600001304.oe.1	2.06	1.04	2.06	up	4.05	3.01	lnc-MARCKS	LNCipedia lc	lnc-MARCKS	---	chr6	113291211	113304369	+
TC0600001414.oe.1	2.07	1.05	2.07	up	4.21	3.15	lnc-L3MBTL:	NONCODE (	lnc-L3MBTL:	NONHSAT1:	chr6	129490683	129502672	+
TC0600001488.oe.1	5.31	2.41	5.31	up	11.34	8.93	lnc-KIAA124	LNCipedia lc	lnc-KIAA124	NONHSAT1:	chr6	137874861	137875744	+
TC0600001524.oe.1	2.57	1.36	2.57	up	4.48	3.12	lnc-VTA1-3	NONCODE (	lnc-VTA1-3:	NONHSAT1:	chr6	142402778	142404246	+
TC0600001579.oe.1	2.15	1.11	2.15	up	6.00	4.90	lnc-SAMD5-	LNCipedia lc	lnc-SAMD5-	---	chr6	147245336	147245815	+
TC0600001583.oe.1	-2.01	-1.01	2.01	down	8.49	9.49	lnc-STXBP5-	LNCipedia lc	lnc-STXBP5-	NONHSAT1:	chr6	147565729	147566713	+
TC0600001593.oe.1	-2.21	-1.14	2.21	down	3.45	4.59	lnc-SUMO4-	LNCipedia lc	lnc-SUMO4-	NONHSAT1:	chr6	149383833	149385871	+
TC0600001610.oe.1	2.59	1.37	2.59	up	6.55	5.18	lnc-PLEKHG:	NONCODE (	lnc-PLEKHG:	NONHSAT1:	chr6	150934968	150935566	+

TC0600001614.oe.1	-2.08	-1.06	2.08	down	3.91	4.97	lnc-AKAP12-	LNCipedia lc	lnc-AKAP12-	NONHSAT1:	chr6	151391544	151404853	+
TC0600001703.oe.1	2.02	1.01	2.02	up	6.36	5.35	lnc-SLC22A3	LNCipedia lc	lnc-SLC22A3	NONHSAT1:	chr6	160272617	160276130	+
TC0600001704.oe.1	-2.51	-1.33	2.51	down	3.92	5.25	lnc-SLC22A1	LNCipedia lc	lnc-SLC22A1	NONHSAT1:	chr6	160403634	160404866	+
TC0600001723.oe.1	-2.65	-1.41	2.65	down	3.82	5.23	lnc-PACRG-	LNCipedia lc	lnc-PACRG-	---	chr6	163506573	163508155	+
TC0600002185.oe.1	-2.22	-1.15	2.22	down	13.97	15.13	lnc-FAM65B	LNCipedia lc	lnc-FAM65B	---	chr6	25161606	25165052	-
TC0600002275.oe.1	-2.14	-1.10	2.14	down	3.71	4.81	lnc-ZFP57-1	LNCipedia lc	lnc-ZFP57-1	---	chr6	29751965	29752207	-
TC0600002286.oe.1	2.16	1.11	2.16	up	7.31	6.19	lnc-TRIM31-	NONCODE (	lnc-TRIM31-	NONHSAT1(	chr6	30102897	30108199	-
TC0600002287.oe.1	4.48	2.16	4.48	up	9.34	7.18	lnc-RNF39-5	LNCipedia lc	lnc-RNF39-5	NONHSAT1(	chr6	30111218	30112475	-
TC0600002336.oe.1	2.11	1.08	2.11	up	6.83	5.75	lnc-C6orf47-	LNCipedia lc	lnc-C6orf47-	NONHSAT1(	chr6	31644494	31645185	-
TC0600002352.oe.1	2.02	1.01	2.02	up	7.21	6.20	lnc-DOM3Z-	LNCipedia lc	lnc-DOM3Z-	NONHSAT1(	chr6	31952227	31959038	-
TC0600002512.oe.1	2.19	1.13	2.19	up	11.12	9.99	lnc-YIPF3-3	NONCODE (	lnc-YIPF3-3:	NONHSAT1:	chr6	43547686	43548470	-
TC0600002543.oe.1	2.54	1.35	2.54	up	4.62	3.27	lnc-GPR110-	LNCipedia lc	lnc-GPR110-	---	chr6	46866255	46869117	-
TC0600002546.oe.1	2.03	1.02	2.03	up	5.82	4.80	lnc-GPR116-	NONCODE (	lnc-GPR116-	NONHSAT1:	chr6	47016617	47042270	-
TC0600002585.oe.1	-2.04	-1.03	2.04	down	12.76	13.79	lnc-GCLC-2	LNCipedia lc	lnc-GCLC-2:	---	chr6	53490296	53490693	-
TC0600002840.oe.1	-2.35	-1.23	2.35	down	2.94	4.17	lnc-ASCC3-(	LNCipedia lc	lnc-ASCC3-(	NONHSAT1:	chr6	101452964	101454237	-
TC0600002850.oe.1	-2.09	-1.07	2.09	down	3.37	4.44	RP11-809N1	LNCipedia lc	lnc-BVES-2:	---	chr6	104831129	104845820	-
TC0600002903.oe.1	2.03	1.02	2.03	up	8.93	7.91	lnc-SLC22A1	LNCipedia lc	lnc-SLC22A1	NONHSAT1:	chr6	110706362	110706882	-
TC0600002984.oe.1	-2.24	-1.16	2.24	down	3.35	4.51	lnc-C6orf17(	LNCipedia lc	lnc-C6orf17(	---	chr6	120344730	120356967	-
TC0600003077.oe.1	-2.39	-1.26	2.39	down	3.97	5.23	lnc-ALDH8A	LNCipedia lc	lnc-ALDH8A	NONHSAT1:	chr6	135032228	135033747	-
TC0600003090.oe.1	-2.00	-1.00	2.00	down	5.86	6.86	lnc-FAM54A	NONCODE (	lnc-FAM54A	NONHSAT1:	chr6	136260746	136273550	-
TC0600003115.oe.1	-2.15	-1.10	2.15	down	3.26	4.37	lnc-PERP-5	LNCipedia lc	lnc-PERP-5:	---	chr6	137908360	137908573	-
TC0600003140.oe.1	-2.14	-1.10	2.14	down	3.34	4.44	lnc-NMBR-3	LNCipedia lc	lnc-NMBR-3	NONHSAT1:	chr6	142058330	142075922	-
TC0600003146.oe.1	2.01	1.01	2.01	up	8.63	7.62	lnc-ADAT2-.	NONCODE (	lnc-ADAT2-.	NONHSAT1:	chr6	142779543	142837047	-
TC0600003256.oe.1	-2.73	-1.45	2.73	down	4.07	5.52	lnc-SERAC1-	NONCODE (	lnc-SERAC1-	NONHSAT1:	chr6	157310590	157312707	-
TC0600003285.oe.1	-2.05	-1.04	2.05	down	2.84	3.88	lnc-SOD2-7	LNCipedia lc	lnc-SOD2-7	---	chr6	159559297	159560091	-
TC0600003289.oe.1	2.16	1.11	2.16	up	11.88	10.76	lnc-TCP1-5	LNCipedia lc	lnc-TCP1-5:	NONHSAT1:	chr6	159682610	159692650	-
TC0600003306.oe.1	-2.65	-1.41	2.65	down	3.24	4.65	lnc-LPA-2	LNCipedia lc	lnc-LPA-2:1	NONHSAT1:	chr6	160815550	160817351	-
TC0600003340.oe.1	2.01	1.00	2.01	up	4.14	3.13	lnc-C6orf11{	LNCipedia lc	lnc-C6orf11{	NONHSAT1:	chr6	165327486	165328978	-
TC0600003425.oe.1	2.20	1.14	2.20	up	6.29	5.15	lnc-PSMB1-	LNCipedia lc	lnc-PSMB1-	NONHSAT1:	chr6	170575594	170576937	-
TC06002076.hg.4	-2.09	-1.07	2.09	down	3.21	4.28	STL//RP11-5	novel transc	---	---	chr6	124908246	124963039	-
TC06002292.hg.4	-2.39	-1.26	2.39	down	3.76	5.02	CAHM	colon adeno	---	---	chr6	163413065	163413950	-
TC06004094.hg.4	-2.06	-1.04	2.06	down	2.62	3.66	C6orf164	chromosome	---	---	chr6	87382002	87399749	+
TC0700000182.oe.1	-2.10	-1.07	2.10	down	3.30	4.37	lnc-AHR-5	NONCODE (	lnc-AHR-5:1	NONHSAT1:	chr7	17080598	17082573	+
TC0700000183.oe.1	-2.26	-1.17	2.26	down	13.83	15.00	lnc-TSPAN1:	LNCipedia lc	lnc-TSPAN1:	NONHSAT1:	chr7	17329858	17333950	+
TC0700000214.oe.1	2.06	1.04	2.06	up	6.03	4.99	lnc-ABCB5-:	LNCipedia lc	lnc-ABCB5-:	NONHSAT1:	chr7	20588990	20590085	+
TC0700000260.oe.1	-2.53	-1.34	2.53	down	3.51	4.85	lnc-NPY-2	LNCipedia lc	lnc-NPY-2:1	---	chr7	24654726	24654954	+
TC0700000282.oe.1	2.28	1.19	2.28	up	7.11	5.92	lnc-C7orf71-	NONCODE (	lnc-C7orf71-	NONHSAT1:	chr7	26667384	26668467	+
TC0700000302.oe.1	-2.29	-1.20	2.29	down	7.34	8.54	lnc-EVX1-23	NONCODE (	lnc-EVX1-23	NONHSAT1:	chr7	27769678	27772539	+
TC0700000395.oe.1	-2.33	-1.22	2.33	down	14.51	15.73	lnc-EEPD1-3	NONCODE (	lnc-EEPD1-3	NONHSAT1:	chr7	36403493	36417148	+
TC0700000454.oe.1	2.00	1.00	2.00	up	6.25	5.25	lnc-STK17A-	NONCODE (	lnc-STK17A-	NONHSAT1:	chr7	43479608	43484615	+
TC0700000473.oe.1	2.30	1.20	2.30	up	5.45	4.24	lnc-PPIA-5	NONCODE (	lnc-PPIA-5:1	NONHSAT1:	chr7	44756188	44756992	+
TC0700000557.oe.1	2.06	1.04	2.06	up	9.38	8.34	lnc-EGFR-2	LNCipedia lc	lnc-EGFR-2:	NONHSAT1:	chr7	55428056	55431406	+
TC0700000649.oe.1	2.27	1.18	2.27	up	5.43	4.25	lnc-ZNF138-	LNCipedia lc	lnc-ZNF138-	NONHSAT1:	chr7	64681133	64686885	+
TC0700000693.oe.1	2.22	1.15	2.22	up	6.31	5.15	lnc-TYW1-1	LNCipedia lc	lnc-TYW1-1	NONHSAT1:	chr7	66995173	66998855	+
TC0700000819.oe.1	-2.55	-1.35	2.55	down	7.76	9.11	lnc-RSBN1L-	LNCipedia lc	lnc-RSBN1L-	NONHSAT1:	chr7	77940199	77954894	+
TC0700000833.oe.1	-2.13	-1.09	2.13	down	5.34	6.43	lnc-CD36-3	LNCipedia lc	lnc-CD36-3:	NONHSAT1:	chr7	80218678	80219288	+
TC0700000936.oe.1	-2.47	-1.30	2.47	down	6.64	7.94	lnc-DYNC1I:	NONCODE (	lnc-DYNC1I:	NONHSAT1:	chr7	96117188	96121442	+
TC0700001023.oe.1	2.62	1.39	2.62	up	9.89	8.51	lnc-TRIP6-3	NONCODE (	lnc-TRIP6-3:	NONHSAT1:	chr7	100887002	100887496	+
TC0700001033.oe.1	3.69	1.88	3.69	up	11.50	9.62	lnc-AP1S1-1	LNCipedia lc	lnc-AP1S1-1	NONHSAT1:	chr7	101127105	101128534	+

TC0700001098.oe.1	-2.64	-1.40	2.64	down	2.34	3.74	lnc-GPR22-1	NONCODE (	lnc-GPR22-1	NONHSAT12	chr7	107470018	107471549	+
TC0700001149.oe.1	-2.03	-1.02	2.03	down	7.83	8.85	lnc-CAPZA2	LNCipedia l	lnc-CAPZA2	NONHSAT12	chr7	116736098	116736353	+
TC0700001228.oe.1	2.00	1.00	2.00	up	8.38	7.38	lnc-FSCN3-4	LNCipedia l	lnc-FSCN3-4	NONHSAT12	chr7	127652181	127652422	+
TC0700001294.oe.1	-3.71	-1.89	3.71	down	3.74	5.63	lnc-MEST-6	LNCipedia l	lnc-MEST-6:	NONHSAT12	chr7	131339965	131344277	+
TC0700001513.oe.1	-2.02	-1.01	2.02	down	3.44	4.45	lnc-GIMAP2	LNCipedia l	lnc-GIMAP2	NONHSAT12	chr7	150685697	150687518	+
TC0700001553.oe.1	2.14	1.10	2.14	up	9.33	8.23	lnc-GALNT1	NONCODE l	lnc-GALNT1	NONHSAT12	chr7	152783193	152816522	+
TC0700001668.oe.1	2.19	1.13	2.19	up	10.44	9.31	lnc-C7orf50-	LNCipedia l	lnc-C7orf50-	NONHSAT11	chr7	1152908	1155677	-
TC0700001683.oe.1	2.81	1.49	2.81	up	7.21	5.71	lnc-AC07438	NONCODE (	lnc-AC07438	NONHSAT11	chr7	1738630	1742310	-
TC0700001774.oe.1	-2.54	-1.34	2.54	down	7.10	8.45	lnc-THSD7A	LNCipedia l	lnc-THSD7A	NONHSAT11	chr7	11370435	11371320	-
TC0700001926.oe.1	-2.39	-1.26	2.39	down	4.41	5.67	lnc-HOXA13	LNCipedia l	lnc-HOXA13	NONHSAT11	chr7	27193503	27195600	-
TC0700001928.oe.1	-3.47	-1.80	3.47	down	7.08	8.88	lnc-HOXA11	LNCipedia l	lnc-HOXA11	NONHSAT11	chr7	27196880	27198353	-
TC0700002081.oe.1	2.48	1.31	2.48	up	8.53	7.22	lnc-TMED4-	NONCODE (	lnc-TMED4-	NONHSAT12	chr7	44565807	44567782	-
TC0700002091.oe.1	2.01	1.01	2.01	up	10.93	9.92	lnc-NACAD-	LNCipedia l	lnc-NACAD-	NONHSAT12	chr7	45107751	45111697	-
TC0700002096.oe.1	-2.98	-1.58	2.98	down	12.89	14.47	lnc-AC01129	LNCipedia l	lnc-AC01129	NONHSAT12	chr7	45913773	45915325	-
TC0700002126.oe.1	2.11	1.08	2.11	up	8.83	7.76	lnc-DDC-1	LNCipedia l	lnc-DDC-1:1	NONHSAT12	chr7	50595470	50607068	-
TC0700002190.oe.1	4.21	2.07	4.21	up	5.12	3.05	lnc-ZNF680-	LNCipedia l	lnc-ZNF680-	NONHSAT12	chr7	62308860	62313387	-
TC0700002331.oe.1	2.02	1.01	2.02	up	10.08	9.07	lnc-HIP1-4	LNCipedia l	lnc-HIP1-4:1	NONHSAT12	chr7	75423102	75424809	-
TC0700002332.oe.1	2.07	1.05	2.07	up	11.61	10.56	lnc-HIP1-3/	LNCipedia l	lnc-HIP1-3:1	NONHSAT12	chr7	75425532	75437668	-
TC0700002383.oe.1	2.36	1.24	2.36	up	4.52	3.28	lnc-CACNA2	LNCipedia l	lnc-CACNA2	NONHSAT12	chr7	81946444	81949661	-
TC0700002435.oe.1	-2.14	-1.10	2.14	down	6.05	7.15	lnc-ERVW-1	NONCODE (	lnc-ERVW-1	NONHSAT12	chr7	92510865	92513967	-
TC0700002445.oe.1	4.49	2.17	4.49	up	6.41	4.25	lnc-BET1-3	LNCipedia l	lnc-BET1-3:1	NONHSAT12	chr7	93886820	93890321	-
TC0700002500.oe.1	2.22	1.15	2.22	up	8.61	7.46	lnc-MCM7-1	LNCipedia l	lnc-MCM7-1	NONHSAT12	chr7	100112120	100112896	-
TC0700002555.oe.1	2.46	1.30	2.46	up	10.11	8.82	lnc-NAPEPLI	NONCODE (	lnc-NAPEPLI	NONHSAT12	chr7	102926283	102933735	-
TC0700002600.oe.1	-2.18	-1.12	2.18	down	2.90	4.02	lnc-COG5-6	LNCipedia l	lnc-COG5-6	---	chr7	106973011	106973828	-
TC0700002632.oe.1	3.23	1.69	3.23	up	8.76	7.07	lnc-IMMP2L	LNCipedia l	lnc-IMMP2L	NONHSAT12	chr7	111778327	111788920	-
TC0700002633.oe.1	2.88	1.52	2.88	up	7.29	5.76	lnc-IMMP2L	LNCipedia l	lnc-IMMP2L	NONHSAT12	chr7	111869460	111872347	-
TC0700002739.oe.1	2.36	1.24	2.36	up	11.22	9.99	lnc-PRRT4-1	NONCODE (	lnc-PRRT4-1	NONHSAT12	chr7	128335547	128337204	-
TC0700002781.oe.1	2.52	1.33	2.52	up	8.74	7.41	lnc-PLXNA4	NONCODE (	lnc-PLXNA4	NONHSAT12	chr7	131509069	131510898	-
TC0700002798.oe.1	3.58	1.84	3.58	up	5.85	4.01	lnc-AC08386	LNCipedia l	lnc-AC08386	---	chr7	135159883	135160161	-
TC0700002799.oe.1	2.18	1.12	2.18	up	6.55	5.42	lnc-WDR91-	LNCipedia l	lnc-WDR91-	NONHSAT12	chr7	135166344	135168179	-
TC0700002836.oe.1	3.29	1.72	3.29	up	6.72	5.00	lnc-JHDM1D	NONCODE (	lnc-JHDM1D	NONHSAT12	chr7	140057407	140062551	-
TC07000072.hg.4	2.63	1.40	2.63	up	6.76	5.37	PMS2CL	PMS2 C-terr	---	---	chr7	6710126	6753862	+
TC07000955.hg.4	2.49	1.32	2.49	up	6.71	5.39	OR2A9P//OI	olfactory rec	---	---	chr7	144294480	144300931	+
TC07001275.hg.4	-2.57	-1.36	2.57	down	2.77	4.13	DPY19L2P1	DPY19L2 pse	---	---	chr7	35079989	35186162	-
TC07001735.hg.4	2.57	1.36	2.57	up	5.29	3.92	EFCAB10	EF-hand calc	---	---	chr7	105565120	105600875	-
TC07003333.hg.4	2.13	1.09	2.13	up	5.72	4.63	NSUN5P1	NOP2/Sun d	---	---	chr7	75410341	75416786	+
TC0800000101.oe.1	2.02	1.02	2.02	up	6.31	5.30	lnc-MTMR9-	LNCipedia l	lnc-MTMR9-	NONHSAT12	chr8	11256256	11256603	+
TC0800000164.oe.1	-3.03	-1.60	3.03	down	6.95	8.56	lnc-NAT1-5	LNCipedia l	lnc-NAT1-5:	NONHSAT12	chr8	17960094	17964770	+
TC0800000165.oe.1	-3.14	-1.65	3.14	down	6.68	8.33	lnc-NAT1-4	LNCipedia l	lnc-NAT1-4:	NONHSAT12	chr8	17972673	17986263	+
TC0800000167.oe.1	-2.17	-1.11	2.17	down	5.51	6.63	lnc-NAT1-2	NONCODE (	lnc-NAT1-2:	NONHSAT12	chr8	18006054	18009665	+
TC0800000168.oe.1	-3.12	-1.64	3.12	down	5.32	6.97	lnc-NAT1-1	NONCODE (	lnc-NAT1-1:	NONHSAT12	chr8	18013882	18025611	+
TC0800000230.oe.1	2.82	1.49	2.82	up	6.62	5.13	lnc-TNFRSF1	LNCipedia l	lnc-TNFRSF1	NONHSAT12	chr8	23007839	23010641	+
TC0800000341.oe.1	2.51	1.33	2.51	up	9.73	8.41	lnc-GS1-211	NONCODE (	lnc-GS1-211	NONHSAT12	chr8	30545200	30547519	+
TC0800000360.oe.1	2.17	1.12	2.17	up	5.52	4.40	lnc-NRG1-3	LNCipedia l	lnc-NRG1-3	---	chr8	32766125	32767959	+
TC0800000623.oe.1	-2.83	-1.50	2.83	down	3.60	5.10	NONHSAG0	LNCipedia l	lnc-NKAIN3-	NONHSAT12	chr8	63215981	63218034	+
TC0800000688.oe.1	-2.50	-1.32	2.50	down	2.94	4.26	lnc-RP11-38	LNCipedia l	lnc-RP11-38	NONHSAT12	chr8	71753355	71756706	+
TC0800000700.oe.1	2.43	1.28	2.43	up	11.51	10.23	lnc-TERF1-4	LNCipedia l	lnc-TERF1-4	NONHSAT12	chr8	73321840	73323308	+
TC0800000742.oe.1	-2.07	-1.05	2.07	down	9.03	10.08	lnc-FAM164	LNCipedia l	lnc-FAM164	NONHSAT12	chr8	78718080	78719760	+
TC0800000803.oe.1	2.04	1.03	2.04	up	14.49	13.46	lnc-OSGIN2	LNCipedia l	lnc-OSGIN2-	NONHSAT12	chr8	89758142	89769824	+

TC0800000847.oe.1	-2.16	-1.11	2.16	down	5.96	7.07	lnc-DPY19L4	LNCipedia lc	lnc-DPY19L4	NONHSAT12	chr8	94793492	94793843	+
TC0800000864.oe.1	2.26	1.18	2.26	up	7.72	6.55	lnc-SDC2-2	LNCipedia lc	lnc-SDC2-2:	NONHSAT12	chr8	96330317	96333892	+
TC0800001059.oe.1	2.55	1.35	2.55	up	6.75	5.40	lnc-FER1L6-	N/A//NONC	lnc-FER1L6-	NONHSAT12	chr8	124271683	124277584	+
TC0800001065.oe.1	-2.32	-1.22	2.32	down	3.38	4.60	lnc-ZNF572-	LNCipedia lc	lnc-ZNF572-	---	chr8	125012451	125020276	+
TC0800001073.oe.1	2.16	1.11	2.16	up	8.03	6.92	lnc-NSMCE2	LNCipedia lc	lnc-NSMCE2	NONHSAT12	chr8	125432859	125433581	+
TC0800001134.oe.1	5.37	2.42	5.37	up	9.67	7.24	lnc-WISP1-5	LNCipedia lc	lnc-WISP1-5	NONHSAT12	chr8	133229056	133229697	+
TC0800001297.oe.1	-2.19	-1.13	2.19	down	3.79	4.93	lnc-C8orf42-	LNCipedia lc	lnc-C8orf42-	NONHSAT12	chr8	664622	670092	-
TC0800001446.oe.1	2.19	1.13	2.19	up	4.43	3.30	lnc-LONRF1	LNCipedia lc	lnc-LONRF1	NONHSAT12	chr8	12696307	12697273	-
TC0800001488.oe.1	-2.81	-1.49	2.81	down	3.19	4.68	RP11-108A1	N/A	---	---	chr8	18864681	18865247	-
TC0800001537.oe.1	2.55	1.35	2.55	up	6.37	5.02	lnc-NKX3-1-	LNCipedia lc	lnc-NKX3-1-	NONHSAT12	chr8	23678694	23682937	-
TC0800001576.oe.1	2.09	1.07	2.09	up	5.12	4.05	lnc-ZNF395-	NONCODE c	lnc-ZNF395-	NONHSAT12	chr8	28446963	28490226	-
TC0800001732.oe.1	4.85	2.28	4.85	up	5.48	3.20	lnc-RNF170-	NONCODE c	lnc-RNF170-	NONHSAT12	chr8	43239511	43240930	-
TC0800001889.oe.1	-2.46	-1.30	2.46	down	4.82	6.11	lnc-ARMC1-	LNCipedia lc	lnc-ARMC1-	NONHSAT12	chr8	65591850	65597752	-
TC0800001902.oe.1	-2.01	-1.01	2.01	down	3.20	4.21	lnc-MYBL1-1	LNCipedia lc	lnc-MYBL1-1	NONHSAT12	chr8	66419587	66428977	-
TC0800001922.oe.1	-2.09	-1.06	2.09	down	4.35	5.41	lnc-RP11-66	NONCODE c	lnc-RP11-66	NONHSAT12	chr8	68974883	68976198	-
TC0800001949.oe.1	2.30	1.20	2.30	up	9.54	8.33	lnc-TRPA1-2	LNCipedia lc	lnc-TRPA1-2	NONHSAT12	chr8	71842349	71843413	-
TC0800001951.oe.1	2.46	1.30	2.46	up	6.04	4.75	lnc-MSC-3	NONCODE c	lnc-MSC-3:1	NONHSAT12	chr8	72034310	72046545	-
TC0800001997.oe.1	2.99	1.58	2.99	up	6.04	4.46	lnc-MRPS28	LNCipedia lc	lnc-MRPS28	NONHSAT12	chr8	79764494	79766493	-
TC0800002013.oe.1	-2.08	-1.06	2.08	down	4.33	5.38	lnc-PAG1-1:	LNCipedia lc	lnc-PAG1-1:	NONHSAT12	chr8	80628457	80631612	-
TC0800002118.oe.1	-2.21	-1.14	2.21	down	4.11	5.25	CTD-2006H1	LNCipedia lc	lnc-RBM12B	---	chr8	93715378	93716113	-
TC0800002242.oe.1	2.60	1.38	2.60	up	10.86	9.49	lnc-KLF10-2	NONCODE l	lnc-KLF10-2	NONHSAT12	chr8	102648775	102653964	-
TC0800002315.oe.1	-3.06	-1.61	3.06	down	4.37	5.99	lnc-EIF3H-3	LNCipedia lc	lnc-EIF3H-3:	NONHSAT12	chr8	115568457	115569020	-
TC0800002317.oe.1	3.30	1.72	3.30	up	10.16	8.44	NONHSAG0	NONCODE c	lnc-EIF3H-2:	NONHSAT12	chr8	116402543	116403757	-
TC0800002342.oe.1	-2.17	-1.12	2.17	down	3.24	4.36	lnc-TAF2-3	NONCODE c	lnc-TAF2-3:1	NONHSAT12	chr8	119572620	119578485	-
TC0800002437.oe.1	-2.26	-1.18	2.26	down	3.67	4.85	lnc-GSDMC-	LNCipedia lc	lnc-GSDMC-	---	chr8	129285647	129310432	-
TC0800002444.oe.1	3.69	1.88	3.69	up	8.33	6.45	lnc-GSDMC-	LNCipedia lc	lnc-GSDMC-	NONHSAT12	chr8	129706110	129707182	-
TC0800002481.oe.1	2.39	1.26	2.39	up	11.93	10.67	lnc-NDRG1-	LNCipedia lc	lnc-NDRG1-	NONHSAT12	chr8	133454853	133458864	-
TC0800002482.oe.1	2.12	1.08	2.12	up	8.12	7.04	lnc-NDRG1-	NONCODE c	lnc-NDRG1-	NONHSAT12	chr8	133476029	133571926	-
TC0800002621.oe.1	2.14	1.10	2.14	up	8.56	7.46	lnc-SLC39A4	NONCODE c	lnc-SLC39A4	NONHSAT12	chr8	144393565	144394761	-
TC0800002624.oe.1	2.22	1.15	2.22	up	7.73	6.58	lnc-CPSF1-1	LNCipedia lc	lnc-CPSF1-1	NONHSAT12	chr8	144412415	144413586	-
TC08000033.hg.4	-2.18	-1.13	2.18	down	3.68	4.81	DEFB109P1E	defensin, be	---	---	chr8	7312844	7319951	+
TC08000568.hg.4	-2.51	-1.33	2.51	down	2.43	3.75	RBM12B-AS	RBM12B ant	---	---	chr8	93740111	93740819	+
TC08001043.hg.4	2.26	1.18	2.26	up	4.25	3.07	BIN3-IT1	BIN3 intronic	---	---	chr8	22640369	22642209	-
TC08001136.hg.4	3.26	1.71	3.26	up	8.92	7.21	LOC728024	chromosome	---	---	chr8	37746556	37748046	-
TC0900000062.oe.1	2.71	1.44	2.71	up	10.33	8.89	lnc-CDC37L1	NONCODE c	lnc-CDC37L1	NONHSAT13	chr9	4793087	4841357	+
TC0900000316.oe.1	-2.10	-1.07	2.10	down	3.53	4.60	lnc-UNC13B	NONCODE c	lnc-UNC13B	NONHSAT14	chr9	35406788	35437833	+
TC0900000319.oe.1	2.46	1.30	2.46	up	8.63	7.33	lnc-RUSC2-3	LNCipedia lc	lnc-RUSC2-3	NONHSAT13	chr9	35607225	35608435	+
TC0900000332.oe.1	-2.56	-1.36	2.56	down	13.88	15.24	lnc-HRCT1-2	NONCODE c	lnc-HRCT1-2	NONHSAT13	chr9	35912942	35914642	+
TC0900000646.oe.1	-2.52	-1.34	2.52	down	4.10	5.43	lnc-NAA35-	NONCODE c	lnc-NAA35-	NONHSAT13	chr9	86570888	86588143	+
TC0900000657.oe.1	-2.76	-1.46	2.76	down	11.31	12.78	lnc-CTSL1-8	LNCipedia lc	lnc-CTSL1-8	NONHSAT13	chr9	87639438	87640412	+
TC0900000740.oe.1	2.14	1.10	2.14	up	3.91	2.81	lnc-PTPDC1	LNCipedia lc	lnc-PTPDC1-	---	chr9	94122974	94123577	+
TC0900000938.oe.1	-2.52	-1.33	2.52	down	9.43	10.76	lnc-DNAJC2	NONCODE c	lnc-DNAJC2	NONHSAT13	chr9	111929249	111931308	+
TC0900001298.oe.1	2.16	1.11	2.16	up	4.15	3.04	lnc-PPP1R26	LNCipedia lc	lnc-PPP1R26	NONHSAT13	chr9	135469113	135469802	+
TC0900001534.oe.1	2.48	1.31	2.48	up	4.58	3.27	RP11-321L2	LNCipedia lc	lnc-ELAVL2-	NONHSAT13	chr9	23894990	23898056	-
TC0900001616.oe.1	2.02	1.01	2.02	up	6.10	5.09	lnc-VCP-2	NONCODE c	lnc-VCP-2:1	NONHSAT13	chr9	35074779	35075509	-
TC0900001683.oe.1	-2.14	-1.09	2.14	down	6.04	7.13	lnc-ANKRD1	LNCipedia lc	lnc-ANKRD1	NONHSAT13	chr9	39099815	39109287	-
TC0900001685.oe.1	-2.19	-1.13	2.19	down	4.29	5.43	lnc-ANKRD1	LNCipedia lc	lnc-ANKRD1	NONHSAT13	chr9	39173794	39174513	-
TC0900001811.oe.1	-2.37	-1.24	2.37	down	6.11	7.36	lnc-APBA1-4	LNCipedia lc	lnc-APBA1-4	NONHSAT13	chr9	69731932	69734258	-
TC0900001864.oe.1	-2.73	-1.45	2.73	down	11.73	13.18	lnc-GNA14-	LNCipedia lc	lnc-GNA14-	NONHSAT13	chr9	77716723	77720497	-

TC0900001865.oe.1	-2.00	-1.00	2.00	down	2.91	3.92	lnc-GNAQ-4	LNCipedia lo	lnc-GNAQ-4	---	chr9	78047328	78047558	-
TC0900002102.oe.1	2.25	1.17	2.25	up	4.29	3.12	lnc-OR13C4	NONCODE (	lnc-OR13C4	NONHSAT13	chr9	103960790	103963783	-
TC0900002103.oe.1	-2.50	-1.32	2.50	down	3.79	5.11	lnc-OR13C4	LNCipedia lo	lnc-OR13C4	---	chr9	104013156	104031630	-
TC0900002232.oe.1	3.92	1.97	3.92	up	12.40	10.43	lnc-MEGF9-	NONCODE (	lnc-MEGF9-	NONHSAT13	chr9	120389195	120401019	-
TC0900002260.oe.1	-2.77	-1.47	2.77	down	5.40	6.87	lnc-RC3H2-1	LNCipedia lo	lnc-RC3H2-1	NONHSAT13	chr9	122844556	122848494	-
TC0900002297.oe.1	-2.28	-1.19	2.28	down	3.51	4.70	lnc-SCAI-2	LNCipedia lo	lnc-SCAI-2:1	NONHSAT13	chr9	124942608	124943495	-
TC0900002298.oe.1	-2.05	-1.03	2.05	down	4.50	5.53	lnc-GOLGA1	NONCODE (	lnc-GOLGA1	NONHSAT13	chr9	124947607	124948492	-
TC0900002315.oe.1	2.32	1.22	2.32	up	5.08	3.87	lnc-RPL12-4	LNCipedia lo	lnc-RPL12-4	NONHSAT13	chr9	127106925	127108047	-
TC0900002316.oe.1	-2.13	-1.09	2.13	down	3.21	4.29	lnc-ANGPTL	LNCipedia lo	lnc-ANGPTL	---	chr9	127150992	127158555	-
TC0900002335.oe.1	2.20	1.14	2.20	up	8.02	6.89	lnc-ST6GALI	NONCODE (	lnc-ST6GALI	NONHSAT13	chr9	127908325	127912394	-
TC0900002339.oe.1	2.11	1.07	2.11	up	8.91	7.83	lnc-DPM2-3	LNCipedia lo	lnc-DPM2-3	NONHSAT13	chr9	127953269	127980461	-
TC0900002347.oe.1	2.10	1.07	2.10	up	7.42	6.35	lnc-PTGES2-	LNCipedia lo	lnc-PTGES2-	NONHSAT13	chr9	128177569	128179009	-
TC0900002378.oe.1	2.51	1.33	2.51	up	6.49	5.17	lnc-TOR1A-	LNCipedia lo	lnc-TOR1A-	NONHSAT13	chr9	129738350	129739623	-
TC0900002384.oe.1	-2.06	-1.05	2.06	down	4.93	5.97	lnc-FNBP1-1	LNCipedia lo	lnc-FNBP1-1	NONHSAT13	chr9	130044951	130045620	-
TC09001698.hg.4	-2.66	-1.41	2.66	down	5.11	6.53	RNU6ATAC	RNA, U6atac	---	---	chr9	134164439	134164564	-
TC0X00000088.oe.1	-2.09	-1.06	2.09	down	3.53	4.59	lnc-RAB9A-(	LNCipedia lo	lnc-RAB9A-(	NONHSAT13	chrX	13767905	13769173	+
TC0X00000148.oe.1	-2.00	-1.00	2.00	down	3.51	4.51	GS1-358P8.4	LNCipedia lo	lnc-PDK3-1:	NONHSAT13	chrX	24545516	24550466	+
TC0X00000161.oe.1	-2.30	-1.20	2.30	down	12.45	13.65	lnc-PDK3-12	LNCipedia lo	lnc-PDK3-12	---	chrX	24909857	24910078	+
TC0X00000190.oe.1	-2.12	-1.08	2.12	down	2.70	3.79	lnc-GK-5//N	LNCipedia lo	lnc-GK-5:1	NONHSAT13	chrX	32754940	32756400	+
TC0X00000260.oe.1	2.10	1.07	2.10	up	9.27	8.20	lnc-DUSP21	LNCipedia lo	lnc-DUSP21	NONHSAT13	chrX	43743704	43744658	+
TC0X00000299.oe.1	2.21	1.14	2.21	up	8.90	7.76	lnc-CDK16-2	LNCipedia lo	lnc-CDK16-2	NONHSAT13	chrX	47241454	47242175	+
TC0X00000300.oe.1	2.33	1.22	2.33	up	8.98	7.76	lnc-CDK16-4	LNCipedia lo	lnc-CDK16-4	NONHSAT13	chrX	47247082	47247952	+
TC0X00000336.oe.1	2.21	1.14	2.21	up	9.83	8.69	lnc-WAS-2	NONCODE (	lnc-WAS-2:1	NONHSAT13	chrX	48706303	48707857	+
TC0X00000342.oe.1	2.93	1.55	2.93	up	10.96	9.41	lnc-ERAS-4	LNCipedia lo	lnc-ERAS-4:	NONHSAT13	chrX	48898510	48903084	+
TC0X00000348.oe.1	-2.75	-1.46	2.75	down	3.00	4.47	lnc-MAGIX-4	LNCipedia lo	lnc-MAGIX-4	NONHSAT13	chrX	49133836	49134083	+
TC0X00000355.oe.1	-4.04	-2.02	4.04	down	5.36	7.38	lnc-GAGE12	LNCipedia lo	lnc-GAGE12	NONHSAT13	chrX	49560842	49603790	+
TC0X00000394.oe.1	2.76	1.46	2.76	up	5.35	3.89	lnc-TSPYL2-	LNCipedia lo	lnc-TSPYL2-	NONHSAT13	chrX	53169097	53170914	+
TC0X00000444.oe.1	2.03	1.02	2.03	up	5.17	4.14	NONHSAG0	SPIN4 antise	lnc-ZC3H12I	NONHSAT13	chrX	63349646	63352178	+
TC0X00000468.oe.1	-2.73	-1.45	2.73	down	8.47	9.92	lnc-STARD8	NONCODE I	lnc-STARD8-	NONHSAT13	chrX	68533180	68537282	+
TC0X00000573.oe.1	-2.10	-1.07	2.10	down	3.59	4.66	lnc-PGK1-3	LNCipedia lo	lnc-PGK1-3:	---	chrX	78006117	78006439	+
TC0X00000669.oe.1	-2.07	-1.05	2.07	down	3.08	4.13	lnc-BHLHB9	LNCipedia lo	lnc-BHLHB9	NONHSAT13	chrX	102785667	102786604	+
TC0X00000672.oe.1	-2.02	-1.01	2.02	down	4.24	5.25	lnc-BHLHB9	LNCipedia lo	lnc-BHLHB9	NONHSAT13	chrX	102798396	102799560	+
TC0X00000676.oe.1	-2.11	-1.07	2.11	down	3.74	4.81	lnc-BHLHB9	LNCipedia lo	lnc-BHLHB9	NONHSAT13	chrX	102806741	102807824	+
TC0X00000700.oe.1	-3.47	-1.80	3.47	down	3.09	4.89	lnc-CLDN2-	NONCODE (	lnc-CLDN2-	NONHSAT13	chrX	106865546	106866862	+
TC0X00000719.oe.1	2.95	1.56	2.95	up	9.60	8.04	lnc-RGAG1-	LNCipedia lo	lnc-RGAG1-	NONHSAT13	chrX	110175747	110177786	+
TC0X00000762.oe.1	-3.24	-1.70	3.24	down	7.94	9.64	lnc-WDR44-	NONCODE (	lnc-WDR44-	NONHSAT13	chrX	118578568	118588310	+
TC0X00000874.oe.1	2.02	1.02	2.02	up	5.29	4.28	lnc-PHF6-1	LNCipedia lo	lnc-PHF6-1:	NONHSAT13	chrX	134493582	134493800	+
TC0X00000903.oe.1	3.08	1.62	3.08	up	6.94	5.32	lnc-GPR112-	LNCipedia lo	lnc-GPR112-	NONHSAT13	chrX	136207776	136209947	+
TC0X00000982.oe.1	-2.69	-1.43	2.69	down	4.17	5.60	lnc-CSAG1-	LNCipedia lo	lnc-CSAG1-	NONHSAT13	chrX	152753921	152760222	+
TC0X00001185.oe.1	2.03	1.02	2.03	up	9.83	8.81	lnc-APOO-1	LNCipedia lo	lnc-APOO-1	NONHSAT13	chrX	23707717	23722742	-
TC0X00001198.oe.1	-2.11	-1.08	2.11	down	13.44	14.51	lnc-ARX-2	LNCipedia lo	lnc-ARX-2:1	---	chrX	24909857	24910078	-
TC0X00001274.oe.1	2.06	1.04	2.06	up	4.17	3.13	lnc-CXorf36	LNCipedia lo	lnc-CXorf36-	---	chrX	45631168	45631643	-
TC0X00001326.oe.1	2.13	1.09	2.13	up	5.99	4.89	lnc-SLC35A2	LNCipedia lo	lnc-SLC35A2	NONHSAT13	chrX	48914104	48914914	-
TC0X00001386.oe.1	2.00	1.00	2.00	up	11.18	10.18	lnc-HSD17B	LNCipedia lo	lnc-HSD17B	NONHSAT13	chrX	53431258	53432322	-
TC0X00001388.oe.1	2.05	1.03	2.05	up	4.07	3.04	lnc-HSD17B	LNCipedia lo	lnc-HSD17B	NONHSAT13	chrX	53456273	53457029	-
TC0X00001460.oe.1	-2.09	-1.07	2.09	down	4.14	5.21	DLG3-AS1//	NONCODE (	lnc-TEX11-1	NONHSAT13	chrX	70452956	70455994	-
TC0X00001555.oe.1	2.13	1.09	2.13	up	4.18	3.09	lnc-CHM-2	LNCipedia lo	lnc-CHM-2:	NONHSAT13	chrX	85849410	85850139	-
TC0X00001557.oe.1	-2.12	-1.08	2.12	down	8.67	9.75	lnc-POF1B-1	LNCipedia lo	lnc-POF1B-1	NONHSAT13	chrX	85978656	85981809	-
TC0X00001602.oe.1	2.18	1.13	2.18	up	5.33	4.20	lnc-ARMCX6	LNCipedia lo	lnc-ARMCX6	NONHSAT13	chrX	101537485	101539276	-

TC0X00001657.oe.1	-3.26	-1.70	3.26	down	12.59	14.30	lnc-IRS4-2	LNCipedia lc	lnc-IRS4-2:1	NONHSAT13	chrX	109054131	109054562	-
TC0X00001693.oe.1	-2.08	-1.06	2.08	down	7.17	8.23	lnc-KLHL13-	NONCODE (	lnc-KLHL13-	NONHSAT13	chrX	117282456	117284368	-
TC0X00001737.oe.1	-2.16	-1.11	2.16	down	6.42	7.53	lnc-MRRFP1	NONCODE (	lnc-MRRFP1	NONHSAT13	chrX	123621475	123623042	-
TC0X00001738.oe.1	-3.40	-1.77	3.40	down	10.91	12.67	lnc-MRRFP1	LNCipedia lc	lnc-MRRFP1	NONHSAT13	chrX	123623513	123624609	-
TC0X00001739.oe.1	-2.47	-1.30	2.47	down	11.63	12.94	lnc-MRRFP1	LNCipedia lc	lnc-MRRFP1	NONHSAT13	chrX	123697726	123712904	-
TC0X00001744.oe.1	-2.33	-1.22	2.33	down	5.56	6.78	lnc-ODZ1-6	LNCipedia lc	lnc-ODZ1-6	NONHSAT13	chrX	124101764	124102553	-
TC0X00001773.oe.1	-2.11	-1.08	2.11	down	6.56	7.64	lnc-IGSF1-4	LNCipedia lc	lnc-IGSF1-4:	---	chrX	131577001	131583478	-
TC0X00001810.oe.1	3.25	1.70	3.25	up	6.51	4.82	lnc-ARHGEF	NONCODE (	lnc-ARHGEF	NONHSAT13	chrX	136807223	136807877	-
TC0X00001814.oe.1	2.23	1.16	2.23	up	10.37	9.21	lnc-ARHGEF	LNCipedia lc	lnc-ARHGEF	NONHSAT13	chrX	136879017	136880722	-
TC0X00001834.oe.1	-2.09	-1.06	2.09	down	2.82	3.88	lnc-CDR1-3	LNCipedia lc	lnc-CDR1-3:	NONHSAT13	chrX	140713836	140727872	-
TC0X00001843.oe.1	-2.23	-1.16	2.23	down	5.21	6.37	lnc-IDS-17	LNCipedia lc	lnc-IDS-17:1	---	chrX	146621798	146622418	-
TC0X00001858.oe.1	3.03	1.60	3.03	up	14.20	12.60	lnc-MAGEA9	LNCipedia lc	lnc-MAGEA9	NONHSAT13	chrX	149478751	149478981	-
TC0X00001883.oe.1	-2.54	-1.35	2.54	down	4.04	5.39	lnc-CSAG1-	LNCipedia lc	lnc-CSAG1-	NONHSAT13	chrX	152708261	152714549	-
TC0X00001930.oe.1	2.90	1.54	2.90	up	5.55	4.01	lnc-GAB3-1	LNCipedia lc	lnc-GAB3-1:	NONHSAT13	chrX	154778723	154781596	-
TC0X000246.hg.4	2.27	1.18	2.27	up	4.31	3.13	SSX6	synovial sarc	---	---	chrX	48107980	48120691	+
TC0X000404.hg.4	2.22	1.15	2.22	up	3.52	2.37	TSIX	TSIX transcri	---	---	chrX	73792205	73829231	+
TC0Y00000123.oe.1	-2.31	-1.21	2.31	down	3.10	4.30	lnc-CYorf15/	LNCipedia lc	lnc-CYorf15/	NONHSAT13	chrY	19598188	19598757	+
TC0Y00000162.oe.1	-2.01	-1.01	2.01	down	2.74	3.75	lnc-DAZ2-7	LNCipedia lc	lnc-DAZ2-7:	NONHSAT13	chrY	23734193	23736249	+
TC0Y00000178.oe.1	-2.22	-1.15	2.22	down	2.86	4.01	lnc-DAZ4-1	LNCipedia lc	lnc-DAZ4-1:	NONHSAT13	chrY	24618028	24639148	+
TC0Y00000205.oe.1	-2.10	-1.07	2.10	down	3.49	4.56	lnc-PRYP4-1	LNCipedia lc	lnc-PRYP4-1	NONHSAT13	chrY	56855538	56856204	+
TC0Y00000266.oe.1	-2.62	-1.39	2.62	down	7.53	8.92	lnc-AC00615	LNCipedia lc	lnc-AC00615	NONHSAT13	chrY	10198504	10199102	-
TC0Y00000271.oe.1	2.86	1.52	2.86	up	4.69	3.18	lnc-AC13488	NONCODE (	lnc-AC13488	NONHSAT13	chrY	11692704	11692929	-
TC0Y00000312.oe.1	-3.98	-1.99	3.98	down	7.15	9.14	lnc-HSFY2-1	LNCipedia lc	lnc-HSFY2-1	NONHSAT13	chrY	18992467	18992709	-
TC0Y00000393.oe.1	2.02	1.02	2.02	up	4.52	3.51	lnc-PRYP4-3	LNCipedia lc	lnc-PRYP4-3	NONHSAT13	chrY	56703707	56707491	-
TC1000000127.oe.1	3.95	1.98	3.95	up	13.80	11.81	lnc-ATP5C1-	NONCODE (	lnc-ATP5C1-	NONHSAT01	chr10	7703368	7720901	+
TC1000000238.oe.1	2.73	1.45	2.73	up	4.39	2.94	lnc-C10orf1	LNCipedia lc	lnc-C10orf1	NONHSAT01	chr10	19692310	19790401	+
TC1000000269.oe.1	2.19	1.13	2.19	up	3.71	2.58	lnc-PTF1A-1	LNCipedia lc	lnc-PTF1A-1	NONHSAT01	chr10	23258211	23258545	+
TC1000000390.oe.1	2.03	1.02	2.03	up	6.39	5.37	lnc-CREM-3	NONCODE (	lnc-CREM-3	NONHSAT01	chr10	34875210	34875422	+
TC1000000438.oe.1	4.20	2.07	4.20	up	6.41	4.34	lnc-CCNYL2	LNCipedia lc	lnc-CCNYL2	NONHSAT01	chr10	41843735	41851732	+
TC1000000439.oe.1	2.27	1.18	2.27	up	5.51	4.32	lnc-CCNYL2	gene_id XLC	lnc-CCNYL2	NONHSAT01	chr10	41848343	41856930	+
TC1000000443.oe.1	3.01	1.59	3.01	up	5.36	3.76	lnc-BMS1-6	LNCipedia lc	lnc-BMS1-6	NONHSAT01	chr10	42295630	42295874	+
TC1000000507.oe.1	-2.06	-1.05	2.06	down	4.82	5.86	lnc-PPYR1-2	LNCipedia lc	lnc-PPYR1-2	NONHSAT01	chr10	46525314	46526388	+
TC1000000538.oe.1	-2.37	-1.25	2.37	down	3.95	5.20	lnc-WDFY4-	LNCipedia lc	lnc-WDFY4-	---	chr10	49066718	49066994	+
TC1000000650.oe.1	-2.20	-1.14	2.20	down	2.70	3.83	lnc-HNRNPH	NONCODE (	lnc-HNRNPH	NONHSAT01	chr10	68412497	68413051	+
TC1000000666.oe.1	5.58	2.48	5.58	up	11.08	8.60	lnc-SRGN-2	NONCODE (	lnc-SRGN-2	NONHSAT01	chr10	69088072	69104811	+
TC1000000734.oe.1	2.08	1.05	2.08	up	6.49	5.44	lnc-FUT11-1	NONCODE (	lnc-FUT11-1	NONHSAT01	chr10	73746867	73752102	+
TC1000000834.oe.1	2.03	1.02	2.03	up	6.74	5.72	LINC00857//	NONCODE (	lnc-AL35919	NONHSAT01	chr10	80207710	80219657	+
TC1000000889.oe.1	-2.85	-1.51	2.85	down	6.72	8.23	lnc-PAPSS2-	LNCipedia lc	lnc-PAPSS2-	---	chr10	87868872	87869144	+
TC1000001165.oe.1	-3.03	-1.60	3.03	down	9.59	11.20	lnc-MXI1-2	LNCipedia lc	lnc-MXI1-2:	NONHSAT01	chr10	110119315	110119690	+
TC1000001166.oe.1	-2.09	-1.07	2.09	down	11.32	12.39	lnc-MXI1-1	NONCODE (	lnc-MXI1-1:	NONHSAT01	chr10	110124240	110130393	+
TC1000001176.oe.1	-2.18	-1.12	2.18	down	9.67	10.79	lnc-SHOC2-	LNCipedia lc	lnc-SHOC2-	NONHSAT01	chr10	110885253	110898718	+
TC1000001180.oe.1	-2.66	-1.41	2.66	down	9.86	11.27	lnc-PDCD4-	LNCipedia lc	lnc-PDCD4-	NONHSAT01	chr10	110965032	110985922	+
TC1000001182.oe.1	-2.07	-1.05	2.07	down	3.36	4.41	lnc-SHOC2-	NONCODE (	lnc-SHOC2-	NONHSAT01	chr10	111016820	111022987	+
TC1000001269.oe.1	2.04	1.03	2.04	up	4.31	3.28	lnc-BAG3-2	NONCODE (	lnc-BAG3-2:	NONHSAT01	chr10	119621068	119621880	+
TC1000001379.oe.1	2.07	1.05	2.07	up	10.38	9.33	lnc-MGMT-1	LNCipedia lc	lnc-MGMT-1	NONHSAT01	chr10	130179374	130180382	+
TC1000001431.oe.1	-3.32	-1.73	3.32	down	3.92	5.65	lnc-CYP2E1-	NONCODE (	lnc-CYP2E1-	NONHSAT01	chr10	133779170	133785043	+
TC1000001436.oe.1	-2.10	-1.07	2.10	down	7.63	8.70	lnc-LARP4B-	LNCipedia lc	lnc-LARP4B-	NONHSAT01	chr10	678455	678796	-
TC1000001596.oe.1	2.21	1.15	2.21	up	4.36	3.21	lnc-BEND7-	LNCipedia lc	lnc-BEND7-	NONHSAT01	chr10	13646272	13654526	-
TC1000001646.oe.1	-2.54	-1.34	2.54	down	4.04	5.39	lnc-C10orf1	LNCipedia lc	lnc-C10orf1	NONHSAT01	chr10	21170262	21173838	-

TC1000001650.oe.1	-2.50	-1.32	2.50	down	3.45	4.77	lnc-C10orf1:	LNCipedia lc	lnc-C10orf1:	NONHSAT0:	chr10	21487525	21490472	-
TC1000001663.oe.1	-2.06	-1.04	2.06	down	3.35	4.39	lnc-C10orf1:	LNCipedia lc	lnc-C10orf1:	NONHSAT0:	chr10	22642022	22691830	-
TC1000001803.oe.1	2.19	1.13	2.19	up	5.66	4.53	lnc-BMS1-1	LNCipedia lc	lnc-BMS1-1	NONHSAT0:	chr10	41854927	41855208	-
TC1000001818.oe.1	-2.10	-1.07	2.10	down	3.36	4.43	lnc-HNRNPF	LNCipedia lc	lnc-HNRNPF	---	chr10	43221371	43232349	-
TC1000001916.oe.1	2.01	1.00	2.01	up	8.28	7.27	lnc-SGMS1-	LNCipedia lc	lnc-SGMS1-	NONHSAT0:	chr10	50187211	50199146	-
TC1000001926.oe.1	-3.13	-1.64	3.13	down	5.24	6.89	lnc-A1CF-4	LNCipedia lc	lnc-A1CF-4:	NONHSAT0:	chr10	50800033	50805968	-
TC1000001937.oe.1	-2.59	-1.37	2.59	down	3.29	4.66	lnc-MBL2-3/	N/A//NONC	lnc-MBL2-3:	NONHSAT0:	chr10	52556680	52755544	-
TC1000001966.oe.1	-2.85	-1.51	2.85	down	5.15	6.66	lnc-C10orf4(	NONCODE (	lnc-C10orf4(	NONHSAT0:	chr10	60029691	60044803	-
TC1000001968.oe.1	-4.36	-2.12	4.36	down	6.67	8.80	lnc-C10orf4(	LNCipedia lc	lnc-C10orf4(	NONHSAT0:	chr10	60061921	60064292	-
TC1000001969.oe.1	-3.71	-1.89	3.71	down	7.76	9.65	lnc-C10orf4(	LNCipedia lc	lnc-C10orf4(	NONHSAT0:	chr10	60082508	60084715	-
TC1000001970.oe.1	-4.29	-2.10	4.29	down	5.52	7.63	lnc-C10orf4(	LNCipedia lc	lnc-C10orf4(	NONHSAT0:	chr10	60138517	60140515	-
TC1000001986.oe.1	-2.17	-1.12	2.17	down	5.88	7.00	lnc-JMJD1C-	NONCODE (	lnc-JMJD1C-	NONHSAT0:	chr10	63167221	63176555	-
TC1000001988.oe.1	-2.23	-1.16	2.23	down	7.64	8.79	lnc-EGR2-6	LNCipedia lc	lnc-EGR2-6:	NONHSAT0:	chr10	63214896	63269253	-
TC1000002028.oe.1	-2.18	-1.12	2.18	down	3.33	4.45	lnc-SLC25A1	NONCODE (	lnc-SLC25A1	NONHSAT0:	chr10	68691664	68692941	-
TC1000002052.oe.1	2.15	1.11	2.15	up	10.90	9.79	lnc-PPA1-1	LNCipedia lc	lnc-PPA1-1:	NONHSAT0:	chr10	70218779	70233911	-
TC1000002054.oe.1	-2.06	-1.04	2.06	down	3.25	4.29	lnc-NODAL-	NONCODE (	lnc-NODAL-	NONHSAT0:	chr10	70485336	70486325	-
TC1000002068.oe.1	-3.22	-1.69	3.22	down	4.12	5.80	lnc-C10orf1(	NONCODE (	lnc-C10orf1(	NONHSAT0:	chr10	71706223	71709214	-
TC1000002176.oe.1	2.03	1.02	2.03	up	6.16	5.14	lnc-SFTPD-7	LNCipedia lc	lnc-SFTPD-7	NONHSAT0:	chr10	80031844	80032393	-
TC1000002350.oe.1	2.11	1.08	2.11	up	8.90	7.83	lnc-CHUK-1	LNCipedia lc	lnc-CHUK-1	NONHSAT0:	chr10	100238183	100246116	-
TC1000002413.oe.1	-4.41	-2.14	4.41	down	4.67	6.81	lnc-ARL3-2	NONCODE (	lnc-ARL3-2:	NONHSAT0:	chr10	102830948	102831703	-
TC1000002537.oe.1	2.21	1.15	2.21	up	9.76	8.61	lnc-PRDX3-4	NONCODE (	lnc-PRDX3-4	NONHSAT0:	chr10	119158007	119164196	-
TC1000002561.oe.1	-2.01	-1.01	2.01	down	3.88	4.89	RP11-95I16.	LNCipedia lc	lnc-FGFR2-3	NONHSAT0:	chr10	120925547	120980700	-
TC1000002605.oe.1	-2.55	-1.35	2.55	down	5.63	6.98	lnc-FAM53B	LNCipedia lc	lnc-FAM53B	NONHSAT0:	chr10	124778791	124779097	-
TC1100000163.oe.1	2.02	1.02	2.02	up	8.84	7.82	lnc-OR10AB	NONCODE (	lnc-OR10AB	NONHSAT0:	chr11	7649210	7650998	+
TC1100000178.oe.1	2.15	1.10	2.15	up	9.12	8.02	lnc-AKIP1-9	NONCODE (	lnc-AKIP1-9	NONHSAT0:	chr11	8682788	8684489	+
TC1100000191.oe.1	2.18	1.12	2.18	up	8.17	7.05	lnc-IPO7-1	LNCipedia lc	lnc-IPO7-1::	NONHSAT0:	chr11	9471107	9472716	+
TC1100000225.oe.1	2.11	1.07	2.11	up	9.38	8.31	lnc-MICALCI	LNCipedia lc	lnc-MICALCI	NONHSAT0:	chr11	12225585	12227098	+
TC1100000274.oe.1	2.06	1.04	2.06	up	5.73	4.69	lnc-NUCB2-	NONCODE (	lnc-NUCB2-	NONHSAT0:	chr11	17013998	17052658	+
TC1100000343.oe.1	-2.06	-1.04	2.06	down	2.40	3.44	lnc-LUZP2-4	LNCipedia lc	lnc-LUZP2-4	NONHSAT0:	chr11	23673812	23675768	+
TC1100000391.oe.1	-2.09	-1.06	2.09	down	5.58	6.64	lnc-C11orf4(	LNCipedia lc	lnc-C11orf4(	NONHSAT0:	chr11	30337010	30338431	+
TC1100000601.oe.1	-2.23	-1.15	2.23	down	3.81	4.97	lnc-OR4C13	LNCipedia lc	lnc-OR4C13	NONHSAT0:	chr11	49882999	49884093	+
TC1100000702.oe.1	2.87	1.52	2.87	up	4.88	3.36	NONHSAG0	LNCipedia lc	lnc-PGA5-1:	NONHSAT0:	chr11	61227168	61248533	+
TC1100000724.oe.1	2.70	1.44	2.70	up	7.93	6.49	lnc-FEN1-4	NONCODE (	lnc-FEN1-4:	NONHSAT0:	chr11	61861973	61865319	+
TC1100000848.oe.1	2.03	1.02	2.03	up	7.36	6.34	lnc-RAB1B-1	NONCODE (	lnc-RAB1B-1	NONHSAT0:	chr11	66261930	66263062	+
TC1100000940.oe.1	-2.17	-1.12	2.17	down	8.73	9.85	lnc-LRP5-4	LNCipedia lc	lnc-LRP5-4::	NONHSAT0:	chr11	68558196	68564407	+
TC1100001044.oe.1	2.19	1.13	2.19	up	3.85	2.72	lnc-ATG16L2	LNCipedia lc	lnc-ATG16L2	---	chr11	72931403	72936167	+
TC1100001048.oe.1	-3.21	-1.68	3.21	down	3.52	5.20	lnc-P2RY2-5	LNCipedia lc	lnc-P2RY2-5	---	chr11	73065032	73065388	+
TC1100001073.oe.1	2.05	1.03	2.05	up	10.21	9.18	lnc-POLD3-9	LNCipedia lc	lnc-POLD3-9	NONHSAT0:	chr11	74248243	74254702	+
TC1100001092.oe.1	10.85	3.44	10.85	up	10.06	6.62	lnc-SLCO2B1	LNCipedia lc	lnc-SLCO2B1	NONHSAT0:	chr11	75100563	75188233	+
TC1100001094.oe.1	2.20	1.14	2.20	up	5.59	4.45	lnc-NEU3-3	NONCODE (	lnc-NEU3-3:	NONHSAT0:	chr11	75196275	75206400	+
TC1100001170.oe.1	-2.14	-1.10	2.14	down	6.35	7.45	lnc-ANKRD4	LNCipedia lc	lnc-ANKRD4	NONHSAT0:	chr11	83180144	83184520	+
TC1100001313.oe.1	-2.17	-1.11	2.17	down	6.06	7.17	lnc-YAP1-2	LNCipedia lc	lnc-YAP1-2:	NONHSAT0:	chr11	102047468	102083419	+
TC1100001351.oe.1	-2.73	-1.45	2.73	down	6.66	8.11	lnc-ACAT1-5	LNCipedia lc	lnc-ACAT1-5	NONHSAT0:	chr11	108267340	108271352	+
TC1100001352.oe.1	-2.08	-1.06	2.08	down	6.86	7.91	lnc-ACAT1-(	LNCipedia lc	lnc-ACAT1-(	NONHSAT0:	chr11	108287178	108290667	+
TC1100001353.oe.1	-3.96	-1.99	3.96	down	5.55	7.54	lnc-ACAT1-7	LNCipedia lc	lnc-ACAT1-7	NONHSAT0:	chr11	108293385	108295370	+
TC1100001464.oe.1	2.24	1.16	2.24	up	4.09	2.93	lnc-CD3G-1	LNCipedia lc	lnc-CD3G-1	NONHSAT0:	chr11	118304730	118313744	+
TC1100001537.oe.1	2.10	1.07	2.10	up	8.16	7.09	lnc-CRTAM-	NONCODE (	lnc-CRTAM-	NONHSAT0:	chr11	122709262	122779595	+
TC1100001587.oe.1	2.17	1.12	2.17	up	4.23	3.11	lnc-PATE3-2	LNCipedia lc	lnc-PATE3-2	NONHSAT0:	chr11	125811751	125811959	+
TC1100001689.oe.1	2.03	1.02	2.03	up	5.57	4.55	lnc-SIRT3-2	NONCODE (	lnc-SIRT3-2:	NONHSAT0:	chr11	203485	204363	-

TC1100001729.oe.1	2.85	1.51	2.85	up	9.46	7.95	lnc-POLR2L-	LNCipedia lc	lnc-POLR2L-	NONHSAT01	chr11	881476	882176	-
TC1100001794.oe.1	2.67	1.41	2.67	up	5.47	4.06	lnc-C11orf4(	LNCipedia lc	lnc-C11orf4(	NONHSAT01	chr11	4600800	4601565	-
TC1100002079.oe.1	5.35	2.42	5.35	up	8.93	6.51	lnc-CCDC73	LNCipedia lc	lnc-CCDC73	NONHSAT01	chr11	32389058	32396403	-
TC1100002125.oe.1	2.43	1.28	2.43	up	9.66	8.38	lnc-RAG2-3	LNCipedia lc	lnc-RAG2-3:	NONHSAT01	chr11	36490377	36496510	-
TC1100002126.oe.1	-2.00	-1.00	2.00	down	9.23	10.23	lnc-RAG2-2	LNCipedia lc	lnc-RAG2-2:	---	chr11	36551310	36554855	-
TC1100002186.oe.1	2.28	1.19	2.28	up	7.38	6.19	lnc-PEX16-4	NONCODE (	lnc-PEX16-4	NONHSAT02	chr11	46090507	46119827	-
TC1100002248.oe.1	-2.02	-1.01	2.02	down	3.91	4.92	lnc-OR4C46	LNCipedia lc	lnc-OR4C46	NONHSAT02	chr11	54543571	54559115	-
TC1100002267.oe.1	3.23	1.69	3.23	up	6.26	4.56	lnc-SLC43A3	NONCODE (	lnc-SLC43A3	NONHSAT02	chr11	57406954	57408749	-
TC1100002392.oe.1	-2.23	-1.16	2.23	down	9.46	10.62	lnc-ATL3-2	LNCipedia lc	lnc-ATL3-2::	NONHSAT02	chr11	63624084	63627853	-
TC1100002451.oe.1	-2.17	-1.12	2.17	down	10.72	11.84	lnc-LTBP3-2	NONCODE l	lnc-LTBP3-2	NONHSAT02	chr11	65422322	65511027	-
TC1100002525.oe.1	2.40	1.26	2.40	up	6.07	4.81	lnc-ALDH3B	NONCODE (	lnc-ALDH3B	NONHSAT02	chr11	67749043	67756968	-
TC1100002651.oe.1	-2.13	-1.09	2.13	down	3.04	4.12	lnc-OR2AT4	LNCipedia lc	lnc-OR2AT4	---	chr11	74938634	74939132	-
TC1100002694.oe.1	-2.84	-1.50	2.84	down	2.96	4.46	lnc-PAK1-3	LNCipedia lc	lnc-PAK1-3:	---	chr11	77294538	77294798	-
TC1100002760.oe.1	2.32	1.21	2.32	up	4.56	3.35	lnc-CCDC89	LNCipedia lc	lnc-CCDC89	---	chr11	85694324	85694600	-
TC1100002812.oe.1	-2.46	-1.30	2.46	down	3.84	5.14	lnc-CHORD(	LNCipedia lc	lnc-CHORD(	NONHSAT02	chr11	90194506	90198120	-
TC1100002859.oe.1	-2.11	-1.08	2.11	down	5.97	7.04	lnc-MTMR2-	NONCODE (	lnc-MTMR2-	NONHSAT02	chr11	95775854	95789782	-
TC1100002894.oe.1	3.38	1.76	3.38	up	7.31	5.55	lnc-MMP10-	LNCipedia lc	lnc-MMP10-	NONHSAT02	chr11	102797044	102797339	-
TC1100002895.oe.1	5.16	2.37	5.16	up	8.98	6.61	lnc-MMP10-	LNCipedia lc	lnc-MMP10-	NONHSAT02	chr11	102797397	102798146	-
TC1100002899.oe.1	2.09	1.06	2.09	up	7.81	6.75	lnc-MMP13-	NONCODE (	lnc-MMP13-	NONHSAT02	chr11	103089271	103092126	-
TC1100002901.oe.1	2.08	1.06	2.08	up	4.70	3.64	lnc-DCUN1D	NONCODE (	lnc-DCUN1D	NONHSAT02	chr11	103402588	103403422	-
TC1100002938.oe.1	-2.10	-1.07	2.10	down	5.73	6.80	lnc-C11orf6!	LNCipedia lc	lnc-C11orf6!	NONHSAT02	chr11	108158638	108161683	-
TC1100002954.oe.1	-2.20	-1.14	2.20	down	12.51	13.65	lnc-ARHGAF	LNCipedia lc	lnc-ARHGAF	NONHSAT02	chr11	110230175	110230558	-
TC1100002985.oe.1	-2.04	-1.03	2.04	down	2.51	3.54	NONHSAG0	gene_id XLC	lnc-IL18-1:2	NONHSAT02	chr11	112165197	112172606	-
TC1100003047.oe.1	2.05	1.03	2.05	up	7.81	6.78	lnc-AP00089	LNCipedia lc	lnc-AP00089	NONHSAT02	chr11	117290487	117291765	-
TC1100003133.oe.1	-2.13	-1.09	2.13	down	3.34	4.43	lnc-BLID-5/	LNCipedia lc	lnc-BLID-5:1	NONHSAT02	chr11	122095900	122098353	-
TC1100003141.oe.1	2.07	1.05	2.07	up	18.85	17.80	lnc-CLMP-3	LNCipedia lc	lnc-CLMP-3	NONHSAT02	chr11	123057493	123062136	-
TC11000245.hg.4	-2.11	-1.08	2.11	down	2.89	3.98	SPTY2D1-AS	SPTY2D1 an	---	---	chr11	18599787	18610255	+
TC11000631.hg.4	-3.35	-1.74	3.35	down	14.10	15.84	MALAT1	metastasis a:	---	---	chr11	65497762	65506469	+
TC11002277.hg.4	-4.27	-2.09	4.27	down	5.71	7.80	COLCA1	colorectal ca	---	---	chr11	111293389	111305045	-
TC1200000029.oe.1	2.15	1.11	2.15	up	12.35	11.24	lnc-ERC1-1	NONCODE (	lnc-ERC1-1::	NONHSAT02	chr12	1500491	1507318	+
TC1200000147.oe.1	2.04	1.03	2.04	up	7.29	6.27	lnc-U47924.	NONCODE (	lnc-U47924.	NONHSAT02	chr12	7060717	7062808	+
TC1200000148.oe.1	3.19	1.67	3.19	up	11.98	10.31	lnc-U47924.	LNCipedia lc	lnc-U47924.	NONHSAT02	chr12	7066192	7068530	+
TC1200000243.oe.1	-2.09	-1.06	2.09	down	5.15	6.21	RP11-180M1	N/A	---	---	chr12	12718973	12719521	+
TC1200000277.oe.1	2.44	1.29	2.44	up	5.60	4.32	lnc-H2AFJ-4	NONCODE (	lnc-H2AFJ-4	NONHSAT02	chr12	14786070	14787500	+
TC1200000314.oe.1	-2.02	-1.01	2.02	down	3.97	4.98	NONHSAG0	LNCipedia lc	lnc-AEBP2-3	NONHSAT02	chr12	19147074	19154659	+
TC1200000332.oe.1	-2.06	-1.04	2.06	down	7.91	8.95	lnc-GOLT1B	LNCipedia lc	lnc-GOLT1B	NONHSAT02	chr12	21454782	21456062	+
TC1200000390.oe.1	2.03	1.02	2.03	up	5.97	4.95	lnc-STK38L-	NONCODE (	lnc-STK38L-	NONHSAT02	chr12	27368317	27377503	+
TC1200000406.oe.1	-2.09	-1.07	2.09	down	3.99	5.06	lnc-KLHDC5	NONCODE (	lnc-KLHDC5	NONHSAT02	chr12	28161256	28163249	+
TC1200000409.oe.1	-2.29	-1.19	2.29	down	3.90	5.10	RP11-425D1	N/A	---	---	chr12	28236227	28236828	+
TC1200000496.oe.1	-2.02	-1.01	2.02	down	2.84	3.85	lnc-C12orf4(	LNCipedia lc	lnc-C12orf4(	---	chr12	40050421	40051840	+
TC1200000545.oe.1	-2.03	-1.02	2.03	down	3.57	4.59	lnc-FAM113	gene_id XLC	lnc-FAM113	NONHSAT02	chr12	47248124	47258595	+
TC1200000660.oe.1	2.22	1.15	2.22	up	5.96	4.81	lnc-AC0210(	NONCODE (	lnc-AC0210(	NONHSAT02	chr12	52241545	52252186	+
TC1200000693.oe.1	-2.25	-1.17	2.25	down	4.59	5.76	lnc-TARBP2-	LNCipedia lc	lnc-TARBP2-	NONHSAT02	chr12	53639006	53639268	+
TC1200000752.oe.1	-2.83	-1.50	2.83	down	8.10	9.60	lnc-RP11-6(	LNCipedia lc	lnc-RP11-6(	NONHSAT02	chr12	56086519	56088064	+
TC1200000754.oe.1	-2.66	-1.41	2.66	down	3.75	5.16	lnc-RP11-6(	LNCipedia lc	lnc-RP11-6(	NONHSAT02	chr12	56093514	56093992	+
TC1200000756.oe.1	-2.13	-1.09	2.13	down	5.43	6.52	lnc-RP11-6(	NONCODE (	lnc-RP11-6(	NONHSAT02	chr12	56099866	56100300	+
TC1200000758.oe.1	2.05	1.03	2.05	up	9.34	8.30	lnc-ZC3H10	NONCODE (	lnc-ZC3H10	NONHSAT02	chr12	56116590	56117831	+
TC1200000848.oe.1	2.54	1.34	2.54	up	4.23	2.88	lnc-TMEM5-	LNCipedia lc	lnc-TMEM5-	---	chr12	63892328	63900553	+
TC1200000881.oe.1	-3.02	-1.59	3.02	down	3.06	4.66	lnc-IRAK3-4	LNCipedia lc	lnc-IRAK3-4	---	chr12	65949374	65949721	+

TC1200000930.oe.1	2.62	1.39	2.62	up	13.32	11.93	lnc-LYZ-2	NONCODE (	lnc-LYZ-2:1	NONHSAT02	chr12	69239620	69258189	+
TC1200001005.oe.1	3.94	1.98	3.94	up	6.92	4.94	lnc-SYT1-10	LNCipedia lc	lnc-SYT1-10	NONHSAT02	chr12	77940267	77950895	+
TC1200001006.oe.1	5.86	2.55	5.86	up	11.03	8.47	lnc-SYT1-9	NONCODE (	lnc-SYT1-9:	NONHSAT02	chr12	77966276	78006594	+
TC1200001007.oe.1	3.98	1.99	3.98	up	7.53	5.54	lnc-SYT1-8	NONCODE (	lnc-SYT1-8:	NONHSAT02	chr12	78007178	78010897	+
TC1200001008.oe.1	3.77	1.91	3.77	up	9.20	7.29	lnc-SYT1-7	LNCipedia lc	lnc-SYT1-7:	NONHSAT02	chr12	78199159	78200562	+
TC1200001134.oe.1	2.28	1.19	2.28	up	6.00	4.81	lnc-RP11-53	LNCipedia lc	lnc-RP11-53	NONHSAT03	chr12	95865079	95866451	+
TC1200001176.oe.1	-2.19	-1.13	2.19	down	10.29	11.42	lnc-NR1H4-	NONCODE (	lnc-NR1H4-	NONHSAT03	chr12	100601642	100618687	+
TC1200001229.oe.1	-2.11	-1.08	2.11	down	9.68	10.76	lnc-C12orf75	NONCODE (	lnc-C12orf75	NONHSAT03	chr12	105156615	105162790	+
TC1200001315.oe.1	2.07	1.05	2.07	up	5.96	4.91	lnc-ALDH2-	LNCipedia lc	lnc-ALDH2-	NONHSAT03	chr12	111843032	111843423	+
TC1200001334.oe.1	3.57	1.84	3.57	up	8.70	6.86	lnc-OAS2-2	LNCipedia lc	lnc-OAS2-2:	NONHSAT03	chr12	112969332	112970705	+
TC1200001416.oe.1	3.83	1.94	3.83	up	7.54	5.60	lnc-SRRM4-	NONCODE (	lnc-SRRM4-	NONHSAT03	chr12	119186800	119193828	+
TC1200001428.oe.1	2.62	1.39	2.62	up	7.45	6.06	lnc-SIRT4-7	LNCipedia lc	lnc-SIRT4-7:	NONHSAT03	chr12	120080681	120090027	+
TC1200001468.oe.1	2.16	1.11	2.16	up	6.17	5.06	lnc-PSMD9-	NONCODE (	lnc-PSMD9-	NONHSAT03	chr12	121902048	121921391	+
TC1200001484.oe.1	-2.43	-1.28	2.43	down	8.92	10.21	lnc-DENR-5	LNCipedia lc	lnc-DENR-5	NONHSAT03	chr12	122592818	122602644	+
TC1200001485.oe.1	-2.69	-1.43	2.69	down	9.78	11.20	lnc-DENR-4	LNCipedia lc	lnc-DENR-4	NONHSAT03	chr12	122609197	122613669	+
TC1200001570.oe.1	-2.26	-1.17	2.26	down	14.20	15.37	lnc-TMEM13	LNCipedia lc	lnc-TMEM13	NONHSAT03	chr12	127165923	127166390	+
TC1200001653.oe.1	2.10	1.07	2.10	up	10.82	9.74	lnc-PXMP2-	LNCipedia lc	lnc-PXMP2-	NONHSAT03	chr12	132717966	132721084	+
TC1200001672.oe.1	2.00	1.00	2.00	up	7.40	6.39	lnc-IQSEC3-	LNCipedia lc	lnc-IQSEC3-	NONHSAT02	chr12	18117	26957	-
TC1200001677.oe.1	2.67	1.42	2.67	up	5.40	3.98	OTTHUMG0	LNCipedia lc	lnc-SLC6A12	NONHSAT02	chr12	164664	166321	-
TC1200001682.oe.1	-2.03	-1.02	2.03	down	4.19	5.21	NONHSAG0	LNCipedia lc	lnc-KDM5A-	NONHSAT02	chr12	273954	277123	-
TC1200001697.oe.1	2.34	1.22	2.34	up	5.47	4.25	lnc-CACNA2	LNCipedia lc	lnc-CACNA2	NONHSAT02	chr12	1587361	1593038	-
TC1200001784.oe.1	2.17	1.12	2.17	up	7.25	6.13	lnc-CHD4-1	NONCODE (	lnc-CHD4-1	NONHSAT02	chr12	6566576	6568296	-
TC1200001809.oe.1	3.08	1.62	3.08	up	5.66	4.04	lnc-RBP5-5	NONCODE (	lnc-RBP5-5:	NONHSAT02	chr12	7089648	7090358	-
TC1200001810.oe.1	2.58	1.37	2.58	up	4.80	3.43	lnc-RBP5-4	NONCODE (	lnc-RBP5-4:	NONHSAT02	chr12	7091156	7092493	-
TC1200001826.oe.1	2.57	1.36	2.57	up	6.86	5.50	lnc-SLC2A14	LNCipedia lc	lnc-SLC2A14	NONHSAT02	chr12	7921547	7925908	-
TC1200001891.oe.1	-2.02	-1.01	2.02	down	4.46	5.47	lnc-TAS2R50	NONCODE (	lnc-TAS2R50	NONHSAT02	chr12	10964425	10965352	-
TC1200001900.oe.1	-2.18	-1.13	2.18	down	7.98	9.11	lnc-MANSC1	LNCipedia lc	lnc-MANSC1	NONHSAT02	chr12	12116029	12119873	-
TC1200001977.oe.1	2.97	1.57	2.97	up	4.65	3.07	lnc-C12orf77	NONCODE (	lnc-C12orf77	NONHSAT02	chr12	24817656	24830328	-
TC1200001988.oe.1	-2.07	-1.05	2.07	down	3.13	4.18	lnc-KRAS-3	NONCODE (	lnc-KRAS-3:	NONHSAT02	chr12	25435581	25436238	-
TC1200002097.oe.1	-2.20	-1.14	2.20	down	6.59	7.72	lnc-ABCD2-	NONCODE (	lnc-ABCD2-	NONHSAT02	chr12	39309752	39321648	-
TC1200002127.oe.1	-2.88	-1.52	2.88	down	5.26	6.79	lnc-PUS7L-1	LNCipedia lc	lnc-PUS7L-1	NONHSAT02	chr12	43718993	43724000	-
TC1200002143.oe.1	-2.07	-1.05	2.07	down	13.31	14.36	lnc-SLC38A1	LNCipedia lc	lnc-SLC38A1	NONHSAT02	chr12	45924947	45926710	-
TC1200002179.oe.1	3.16	1.66	3.16	up	7.92	6.25	lnc-HDAC7-	LNCipedia lc	lnc-HDAC7-	NONHSAT02	chr12	47865053	47879044	-
TC1200002220.oe.1	2.44	1.29	2.44	up	10.33	9.04	lnc-LMBR1L	LNCipedia lc	lnc-LMBR1L	NONHSAT02	chr12	49128981	49131395	-
TC1200002222.oe.1	2.26	1.17	2.26	up	7.95	6.78	OTTHUMG0	LNCipedia lc	lnc-C1QL4-	NONHSAT02	chr12	49265156	49273306	-
TC1200002306.oe.1	2.01	1.01	2.01	up	5.31	4.30	NONHSAG0	LNCipedia lc	lnc-AAAS-1:	NONHSAT02	chr12	53298655	53300314	-
TC1200002307.oe.1	2.05	1.04	2.05	up	7.56	6.52	lnc-SP7-2	NONCODE (	lnc-SP7-2:1	NONHSAT02	chr12	53307738	53315127	-
TC1200002336.oe.1	2.18	1.12	2.18	up	5.69	4.57	lnc-ZNF385	LNCipedia lc	lnc-ZNF385	NONHSAT02	chr12	54403632	54404629	-
TC1200002349.oe.1	2.26	1.18	2.26	up	9.09	7.91	lnc-OR6C70	LNCipedia lc	lnc-OR6C70	NONHSAT02	chr12	55412625	55413431	-
TC1200002375.oe.1	-2.28	-1.19	2.28	down	4.88	6.07	lnc-SMARCC	LNCipedia lc	lnc-SMARCC	NONHSAT02	chr12	56103124	56103508	-
TC1200002376.oe.1	2.10	1.07	2.10	up	7.17	6.10	lnc-SMARCC	LNCipedia lc	lnc-SMARCC	NONHSAT02	chr12	56104614	56113905	-
TC1200002403.oe.1	2.76	1.46	2.76	up	8.67	7.21	lnc-NACA-2	LNCipedia lc	lnc-NACA-2	NONHSAT02	chr12	56688026	56688226	-
TC1200002526.oe.1	-2.06	-1.04	2.06	down	3.06	4.11	RP11-1143G	N/A	---	---	chr12	69326574	69331882	-
TC1200002530.oe.1	-2.18	-1.13	2.18	down	2.41	3.54	LOC101928	LNCipedia lc	lnc-BEST3-1	NONHSAT02	chr12	69705973	69738568	-
TC1200002544.oe.1	2.16	1.11	2.16	up	5.92	4.81	NONHSAG0	LNCipedia lc	lnc-TSPAN8	NONHSAT02	chr12	71047402	71118247	-
TC1200002589.oe.1	2.16	1.11	2.16	up	8.19	7.07	lnc-E2F7-7	LNCipedia lc	lnc-E2F7-7:1	NONHSAT02	chr12	76858716	76864954	-
TC1200002617.oe.1	-2.03	-1.02	2.03	down	4.63	5.65	lnc-LIN7A-3	LNCipedia lc	lnc-LIN7A-3	NONHSAT02	chr12	80792520	80795906	-
TC1200002815.oe.1	6.71	2.75	6.71	up	7.73	4.98	OTTHUMG0	NONCODE (	lnc-NFYB-1:	NONHSAT03	chr12	104262314	104280722	-
TC1200002923.oe.1	2.37	1.25	2.37	up	5.65	4.41	lnc-NAA25-	NONCODE (	lnc-NAA25-	NONHSAT03	chr12	112160263	112162262	-

TC1200002932.oe.1	2.30	1.20	2.30	up	6.76	5.55	lnc-IQCD-1	LNCipedia l	lnc-IQCD-1:	NONHSAT0	chr12	113176828	113177363	-
TC1200002946.oe.1	20.83	4.38	20.83	up	9.66	5.28	lnc-TBX5-4	NONCODE l	lnc-TBX5-4:	NONHSAT0	chr12	114677581	114680987	-
TC1200003041.oe.1	2.19	1.13	2.19	up	7.44	6.31	OTTHUMG0	NONCODE (	lnc-SPPL3.1-	NONHSAT0	chr12	120696314	120703825	-
TC1200003059.oe.1	2.63	1.40	2.63	up	6.21	4.81	lnc-RHOF-4	LNCipedia l	lnc-RHOF-4	NONHSAT0	chr12	121856598	121858855	-
TC1200003076.oe.1	-2.47	-1.31	2.47	down	10.69	11.99	lnc-CDK2AP	NONCODE (	lnc-CDK2AP	NONHSAT0	chr12	123230037	123233101	-
TC1200003096.oe.1	-7.53	-2.91	7.53	down	2.87	5.78	RP11-214K3	LNCipedia l	lnc-DNAH1(	NONHSAT0	chr12	123966077	123966629	-
TC1200003099.oe.1	-2.67	-1.42	2.67	down	4.64	6.06	RP11-214K3	LNCipedia l	lnc-DNAH1(	NONHSAT0	chr12	123971457	123971714	-
TC1200003229.oe.1	2.04	1.03	2.04	up	4.89	3.87	lnc-GALNT9	NONCODE (	lnc-GALNT9	NONHSAT0	chr12	132141006	132142373	-
TC12001201.hg.4	2.49	1.31	2.49	up	7.13	5.82	LOC642846/	DEAD/H (As	---	---	chr12	9417691	9448228	-
TC12001420.hg.4	-4.24	-2.08	4.24	down	2.73	4.81	RNY5	RNA, Ro-ass	---	---	chr12	45187091	45187469	-
TC12002057.hg.4	-3.85	-1.95	3.85	down	7.58	9.52	HNF1A-AS1	HNF1A antis	---	---	chr12	120969838	120972292	-
TC1300000197.oe.1	2.55	1.35	2.55	up	4.22	2.87	lnc-FRY-1	furry homolo	lnc-FRY-1:1	NONHSAT0	chr13	32297335	32299122	+
TC1300000211.oe.1	-2.28	-1.19	2.28	down	3.33	4.52	lnc-RFC3-4	LNCipedia l	lnc-RFC3-4:	NONHSAT0	chr13	33772098	33787164	+
TC1300000447.oe.1	-2.13	-1.09	2.13	down	3.09	4.18	lnc-OLFM4-	NONCODE (	lnc-OLFM4-	NONHSAT0	chr13	53760721	53774984	+
TC1300000680.oe.1	2.17	1.12	2.17	up	4.03	2.91	lnc-GPR180-	LNCipedia l	lnc-GPR180-	NONHSAT0	chr13	94649048	94649586	+
TC1300000783.oe.1	2.65	1.41	2.65	up	5.47	4.06	XLOC_0104	long interge	lnc-DAOA-4	NONHSAT0	chr13	106376563	106378595	+
TC1300001078.oe.1	2.21	1.14	2.21	up	3.65	2.51	LINC00423/	LNCipedia l	lnc-STARD1	NONHSAT1	chr13	32809190	32911652	-
TC1300001179.oe.1	-3.08	-1.62	3.08	down	9.14	10.77	RP11-5G9.6	N/A	---	---	chr13	43894725	43895182	-
TC1300001250.oe.1	-2.19	-1.13	2.19	down	2.88	4.01	lnc-AL13621	LNCipedia l	lnc-AL13621	NONHSAT0	chr13	49533918	49539503	-
TC1300001322.oe.1	-3.02	-1.59	3.02	down	2.72	4.31	lnc-DIAPH3-	LNCipedia l	lnc-DIAPH3-	---	chr13	58559074	58563053	-
TC1300001410.oe.1	-2.03	-1.02	2.03	down	3.41	4.43	LINC01034/	NONCODE (	lnc-COMMD	NONHSAT0	chr13	76013023	76014501	-
TC1300001436.oe.1	-2.16	-1.11	2.16	down	3.08	4.19	lnc-POU4F1	LNCipedia l	lnc-POU4F1	NONHSAT0	chr13	78631381	78632921	-
TC1300001441.oe.1	-2.15	-1.11	2.15	down	3.62	4.73	lnc-RNF219-	LNCipedia l	lnc-RNF219-	---	chr13	79316220	79350237	-
TC1300001471.oe.1	-2.64	-1.40	2.64	down	2.97	4.37	lnc-SLITRK6-	LNCipedia l	lnc-SLITRK6-	---	chr13	88012847	88034890	-
TC1300001503.oe.1	2.24	1.17	2.24	up	4.02	2.85	lnc-DCT-1	LNCipedia l	lnc-DCT-1:1	NONHSAT0	chr13	94488893	94491018	-
TC13000107.hg.4	2.65	1.40	2.65	up	7.26	5.85	EEF1DP3	eukaryotic tr	---	---	chr13	31846783	31959584	+
TC1400000032.oe.1	2.07	1.05	2.07	up	10.41	9.36	lnc-APEX1-3	LNCipedia l	lnc-APEX1-3	NONHSAT0	chr14	20476489	20477087	+
TC1400000036.oe.1	2.20	1.14	2.20	up	4.79	3.65	lnc-EDDM3A	LNCipedia l	lnc-EDDM3A	NONHSAT0	chr14	20721196	20735853	+
TC1400000081.oe.1	2.08	1.05	2.08	up	7.65	6.59	lnc-AE0006	LNCipedia l	lnc-AE0006	NONHSAT0	chr14	22611541	22612358	+
TC1400000157.oe.1	-2.53	-1.34	2.53	down	3.67	5.01	lnc-FOXG1-	LNCipedia l	lnc-FOXG1-	---	chr14	27833680	27833946	+
TC1400000203.oe.1	-3.13	-1.65	3.13	down	9.52	11.17	lnc-RP11-18	LNCipedia l	lnc-RP11-18	NONHSAT0	chr14	32154010	32157216	+
TC1400000247.oe.1	-2.00	-1.00	2.00	down	3.56	4.56	lnc-BRMS1L	LNCipedia l	lnc-BRMS1L	NONHSAT0	chr14	35948352	35949143	+
TC1400000324.oe.1	2.11	1.08	2.11	up	5.09	4.01	lnc-PRPF39-	LNCipedia l	lnc-PRPF39-	NONHSAT0	chr14	45136514	45137959	+
TC1400000353.oe.1	-2.53	-1.34	2.53	down	9.29	10.63	lnc-ARF6-3	LNCipedia l	lnc-ARF6-3:	NONHSAT0	chr14	49894417	49897054	+
TC1400000482.oe.1	-3.94	-1.98	3.94	down	3.86	5.84	lnc-TOMM2	LNCipedia l	lnc-TOMM2	NONHSAT0	chr14	58351297	58359172	+
TC1400000483.oe.1	-2.16	-1.11	2.16	down	4.85	5.96	lnc-TOMM2	LNCipedia l	lnc-TOMM2	NONHSAT0	chr14	58365475	58371960	+
TC1400000540.oe.1	2.35	1.23	2.35	up	4.38	3.14	lnc-SYNE2-5	LNCipedia l	lnc-SYNE2-5	---	chr14	63580381	63580719	+
TC1400000558.oe.1	-2.17	-1.12	2.17	down	5.70	6.81	lnc-FNTB-2	LNCipedia l	lnc-FNTB-2:	NONHSAT0	chr14	64933813	64935368	+
TC1400000576.oe.1	-2.33	-1.22	2.33	down	3.21	4.43	lnc-MPP5-1	NONCODE (	lnc-MPP5-1	NONHSAT0	chr14	67227471	67228544	+
TC1400000664.oe.1	2.01	1.01	2.01	up	6.64	5.63	lnc-ACOT1-	LNCipedia l	lnc-ACOT1-	---	chr14	73490964	73493392	+
TC1400000717.oe.1	-2.07	-1.05	2.07	down	5.38	6.43	lnc-ESRRB-3	LNCipedia l	lnc-ESRRB-3	NONHSAT0	chr14	76196438	76199542	+
TC1400000765.oe.1	-2.27	-1.18	2.27	down	2.57	3.75	lnc-RP11-49	LNCipedia l	lnc-RP11-49	---	chr14	85140628	85197861	+
TC1400000771.oe.1	-2.61	-1.38	2.61	down	4.73	6.12	lnc-RP11-49	NONCODE (	lnc-RP11-49	NONHSAT0	chr14	85623690	85626228	+
TC1400000831.oe.1	2.03	1.02	2.03	up	4.92	3.90	lnc-CTD-25	NONCODE (	lnc-CTD-25	NONHSAT0	chr14	91419449	91419751	+
TC1400000835.oe.1	-2.06	-1.04	2.06	down	2.68	3.72	lnc-SLC24A	LNCipedia l	lnc-SLC24A	---	chr14	92137206	92141887	+
TC1400000895.oe.1	-2.18	-1.12	2.18	down	8.15	9.27	lnc-AK7-3	LNCipedia l	lnc-AK7-3:1	NONHSAT0	chr14	96534223	96537059	+
TC1400001066.oe.1	2.68	1.42	2.68	up	7.31	5.89	lnc-BTBD6-2	NONCODE (	lnc-BTBD6-2	NONHSAT0	chr14	105250677	105251099	+
TC1400001069.oe.1	-2.16	-1.11	2.16	down	3.03	4.15	lnc-TEX22-3	LNCipedia l	lnc-TEX22-3	NONHSAT0	chr14	105374618	105374971	+
TC1400001166.oe.1	2.03	1.02	2.03	up	6.11	5.09	lnc-CDH24-	NONCODE (	lnc-CDH24-	NONHSAT0	chr14	23061572	23062770	-

TC1400001308.oe.1	-3.49	-1.80	3.49	down	4.30	6.10	lnc-NFKBIA-	LNCipedia l	lnc-NFKBIA-	NONHSAT0	chr14	35549174	35572860	-
TC1400001368.oe.1	2.07	1.05	2.07	up	9.61	8.56	lnc-KLHL28-	LNCipedia l	lnc-KLHL28-	NONHSAT0	chr14	44887434	44887741	-
TC1400001442.oe.1	-2.21	-1.15	2.21	down	3.71	4.85	lnc-NID2-5	LNCipedia l	lnc-NID2-5:	NONHSAT0	chr14	51998175	51999916	-
TC1400001623.oe.1	2.29	1.20	2.29	up	6.70	5.51	lnc-VTI1B-2	LNCipedia l	lnc-VTI1B-2	NONHSAT0	chr14	67589755	67592776	-
TC1400001702.oe.1	2.16	1.11	2.16	up	5.24	4.12	lnc-HEATR4	LNCipedia l	lnc-HEATR4	NONHSAT0	chr14	73568238	73569833	-
TC1400001897.oe.1	-2.25	-1.17	2.25	down	8.50	9.67	lnc-CATSPE	LNCipedia l	lnc-CATSPE	NONHSAT0	chr14	91812016	91813828	-
TC1400002000.oe.1	2.07	1.05	2.07	up	7.82	6.77	lnc-SLC25A	NONCODE	lnc-SLC25A	NONHSAT0	chr14	100361703	100375547	-
TC14001019.hg.4	2.16	1.11	2.16	up	8.72	7.61	ARHGAP5-A	ARHGAP5 a	---	---	chr14	32075419	32076793	-
TC14002287.hg.4	-2.05	-1.03	2.05	down	4.64	5.68	ECRP	ribonuclease	---	---	chr14	20919341	20920299	+
TC1500000102.oe.1	-2.26	-1.18	2.26	down	4.38	5.56	lnc-GOLGA	LNCipedia l	lnc-GOLGA	---	chr15	28604592	28607342	+
TC1500000176.oe.1	-2.04	-1.03	2.04	down	3.77	4.80	lnc-C15orf5	LNCipedia l	lnc-C15orf5	NONHSAT0	chr15	34336408	34340368	+
TC1500000232.oe.1	2.32	1.22	2.32	up	6.89	5.67	lnc-C15orf5	NONCODE	lnc-C15orf5	NONHSAT0	chr15	39585635	39588616	+
TC1500000233.oe.1	4.03	2.01	4.03	up	7.37	5.36	lnc-C15orf5	NONCODE	lnc-C15orf5	NONHSAT0	chr15	39588848	39589843	+
TC1500000234.oe.1	2.97	1.57	2.97	up	10.24	8.67	lnc-C15orf5	NONCODE	lnc-C15orf5	NONHSAT0	chr15	39590928	39591618	+
TC1500000235.oe.1	2.98	1.57	2.98	up	13.87	12.30	lnc-EIF2AK4	LNCipedia l	lnc-EIF2AK4	NONHSAT0	chr15	39594068	39595551	+
TC1500000236.oe.1	3.91	1.97	3.91	up	10.86	8.89	lnc-EIF2AK4	LNCipedia l	lnc-EIF2AK4	---	chr15	39595680	39597832	+
TC1500000273.oe.1	-2.34	-1.23	2.34	down	3.28	4.51	lnc-RAD51-	NONCODE	lnc-RAD51-	NONHSAT0	chr15	40662573	40663904	+
TC1500000279.oe.1	2.70	1.43	2.70	up	5.84	4.41	lnc-VPS18-4	NONCODE	lnc-VPS18-4	NONHSAT0	chr15	40854378	40856899	+
TC1500000347.oe.1	-2.03	-1.02	2.03	down	9.44	10.46	lnc-EIF3J-1	LNCipedia l	lnc-EIF3J-1:	NONHSAT0	chr15	44519224	44525947	+
TC1500000379.oe.1	-2.08	-1.06	2.08	down	3.15	4.21	OTTHUMG0	LNCipedia l	lnc-SLC24A	NONHSAT0	chr15	47274183	47275164	+
TC1500000436.oe.1	-2.96	-1.57	2.96	down	4.95	6.52	RP11-56B16	N/A	---	---	chr15	51833134	51833426	+
TC1500000451.oe.1	-2.01	-1.01	2.01	down	3.74	4.75	lnc-MAPK6-	LNCipedia l	lnc-MAPK6-	NONHSAT0	chr15	52577842	52598709	+
TC1500000472.oe.1	-2.06	-1.04	2.06	down	2.88	3.92	RP11-420M	LNCipedia l	lnc-RP11-17	NONHSAT0	chr15	55680385	55681463	+
TC1500000494.oe.1	2.01	1.00	2.01	up	3.69	2.69	OTTHUMG0	NONCODE	lnc-GCOM1	NONHSAT0	chr15	57720295	57720928	+
TC1500000514.oe.1	-2.36	-1.24	2.36	down	5.80	7.04	lnc-RNF111	LNCipedia l	lnc-RNF111	NONHSAT0	chr15	58855827	58857551	+
TC1500000576.oe.1	-2.30	-1.20	2.30	down	3.30	4.50	lnc-FBXL22-	LNCipedia l	lnc-FBXL22-	---	chr15	63676880	63677241	+
TC1500000606.oe.1	-2.51	-1.33	2.51	down	4.63	5.95	lnc-RAB11A	LNCipedia l	lnc-RAB11A	NONHSAT0	chr15	65890771	65891991	+
TC1500000705.oe.1	2.13	1.09	2.13	up	10.84	9.75	lnc-C15orf6	LNCipedia l	lnc-C15orf6	NONHSAT0	chr15	73709565	73714514	+
TC1500000841.oe.1	3.25	1.70	3.25	up	6.79	5.09	lnc-MESDC1	LNCipedia l	lnc-MESDC1	NONHSAT0	chr15	80946944	80949162	+
TC1500000894.oe.1	-2.48	-1.31	2.48	down	2.80	4.10	lnc-ZSCAN2	NONCODE	lnc-ZSCAN2	NONHSAT0	chr15	84661754	84662191	+
TC1500001415.oe.1	3.37	1.75	3.37	up	10.15	8.40	lnc-SPTBN5	LNCipedia l	lnc-SPTBN5	NONHSAT0	chr15	41899432	41900010	-
TC1500001431.oe.1	-2.43	-1.28	2.43	down	10.62	11.90	lnc-EPB42-4	LNCipedia l	lnc-EPB42-4	NONHSAT0	chr15	43015796	43036264	-
TC1500001474.oe.1	2.14	1.10	2.14	up	5.15	4.05	RP11-109D2	LNCipedia l	lnc-DUOX2-	NONHSAT0	chr15	45073492	45074048	-
TC1500001539.oe.1	-2.34	-1.23	2.34	down	7.49	8.71	lnc-USP50-	NONCODE	lnc-USP50-	NONHSAT0	chr15	50574959	50586582	-
TC1500001553.oe.1	-2.90	-1.54	2.90	down	6.28	7.82	lnc-CYP19A	NONCODE	lnc-CYP19A	NONHSAT0	chr15	51453248	51458878	-
TC1500001573.oe.1	-2.09	-1.06	2.09	down	3.18	4.24	RP11-519C1	LNCipedia l	lnc-ARPP19	NONHSAT0	chr15	52648634	52649866	-
TC1500001575.oe.1	-2.17	-1.11	2.17	down	9.42	10.53	lnc-ONECUT	LNCipedia l	lnc-ONECUT	NONHSAT0	chr15	52757315	52791076	-
TC1500001682.oe.1	-3.23	-1.69	3.23	down	5.35	7.04	lnc-C2CD4B	NONCODE	lnc-C2CD4B	NONHSAT0	chr15	61951860	61961671	-
TC1500001813.oe.1	2.11	1.08	2.11	up	5.80	4.72	lnc-UACA-6	NONCODE	lnc-UACA-6	NONHSAT0	chr15	70048977	70058815	-
TC1500001823.oe.1	-3.06	-1.61	3.06	down	6.41	8.02	lnc-LARP6-	NONCODE	lnc-LARP6-	NONHSAT0	chr15	70678099	70680034	-
TC1500001839.oe.1	-3.49	-1.80	3.49	down	3.60	5.40	lnc-GRAMD	LNCipedia l	lnc-GRAMD	NONHSAT0	chr15	71883701	71888607	-
TC1500001841.oe.1	-2.21	-1.14	2.21	down	4.87	6.02	lnc-GRAMD	NONCODE	lnc-GRAMD	NONHSAT0	chr15	71935140	71960403	-
TC1500001879.oe.1	2.61	1.38	2.61	up	8.86	7.48	lnc-RP11-24	LNCipedia l	lnc-RP11-24	NONHSAT0	chr15	74182227	74182878	-
TC1500001969.oe.1	-2.05	-1.03	2.05	down	3.99	5.02	lnc-ACSBG1	LNCipedia l	lnc-ACSBG1	NONHSAT0	chr15	78290527	78291221	-
TC1500002060.oe.1	2.32	1.21	2.32	up	8.80	7.59	lnc-SEC11A	LNCipedia l	lnc-SEC11A	NONHSAT0	chr15	85204032	85204330	-
TC1500002104.oe.1	2.28	1.19	2.28	up	13.60	12.40	lnc-HAPLN3	NONCODE	lnc-HAPLN3	NONHSAT0	chr15	88905382	88906716	-
TC1600000149.oe.1	3.78	1.92	3.78	up	10.75	8.83	lnc-TNFRSF1	NONCODE	lnc-TNFRSF1	NONHSAT1	chr16	3020390	3022383	+
TC1600000197.oe.1	2.34	1.23	2.34	up	7.05	5.83	lnc-ALG1-2	NONCODE	lnc-ALG1-2:	NONHSAT1	chr16	5085071	5087379	+
TC1600000400.oe.1	-2.72	-1.44	2.72	down	4.18	5.62	lnc-AC0043	LNCipedia l	lnc-AC0043	NONHSAT1	chr16	20783264	20786152	+

TC1600000401.oe.1	-2.11	-1.08	2.11	down	4.08	5.15	lnc-AC00438	NONCODE c	lnc-AC00438	NONHSAT14	chr16	20796078	20797156	+
TC1600000408.oe.1	-2.69	-1.43	2.69	down	3.97	5.40	lnc-ANKS4B	NONCODE c	lnc-ANKS4B	NONHSAT14	chr16	21304270	21306591	+
TC1600000491.oe.1	2.08	1.06	2.08	up	5.15	4.09	lnc-KIAA055	LNCipedia lc	lnc-KIAA055	NONHSAT14	chr16	27550133	27574295	+
TC1600000609.oe.1	2.70	1.43	2.70	up	8.44	7.00	lnc-BCKDK-2	LNCipedia lc	lnc-BCKDK-2	---	chr16	31103629	31103977	+
TC1600000622.oe.1	2.82	1.49	2.82	up	4.82	3.32	lnc-ITGAD-2	LNCipedia lc	lnc-ITGAD-2	NONHSAT14	chr16	31371474	31372654	+
TC1600000831.oe.1	-2.76	-1.46	2.76	down	6.31	7.77	lnc-RBL2-4	NONCODE c	lnc-RBL2-4:1	NONHSAT14	chr16	53208032	53226403	+
TC1600000875.oe.1	2.16	1.11	2.16	up	4.33	3.22	RP11-413H2	NONCODE c	lnc-OGFOD1	NONHSAT14	chr16	56409320	56411683	+
TC1600000886.oe.1	2.21	1.14	2.21	up	5.91	4.76	lnc-NUP93-1	LNCipedia lc	lnc-NUP93-1	NONHSAT14	chr16	56841641	56846880	+
TC1600001006.oe.1	-2.25	-1.17	2.25	down	2.89	4.06	lnc-PLEKHG4	NONCODE c	lnc-PLEKHG4	NONHSAT14	chr16	67326980	67347591	+
TC1600001209.oe.1	-2.18	-1.13	2.18	down	4.65	5.78	RP11-358L2	N/A	---	---	chr16	78123243	78124332	+
TC1600001236.oe.1	-2.03	-1.02	2.03	down	3.35	4.37	CTD-2055G2	NONCODE c	lnc-DYNLRB	NONHSAT14	chr16	80736360	80742305	+
TC1600001350.oe.1	2.02	1.02	2.02	up	7.90	6.89	lnc-JPH3-7	LNCipedia lc	lnc-JPH3-7:1	NONHSAT14	chr16	87392423	87399504	+
TC1600001410.oe.1	2.21	1.14	2.21	up	8.36	7.21	lnc-RPL13-4	NONCODE c	lnc-RPL13-4	NONHSAT14	chr16	89579346	89581530	+
TC1600001738.oe.1	2.02	1.02	2.02	up	3.69	2.68	lnc-PARN-1	LNCipedia lc	lnc-PARN-1	---	chr16	14270610	14277767	-
TC1600001797.oe.1	2.03	1.02	2.03	up	8.48	7.46	lnc-ARL6IP1	LNCipedia lc	lnc-ARL6IP1	NONHSAT14	chr16	18788544	18790340	-
TC1600001801.oe.1	-3.13	-1.65	3.13	down	4.60	6.25	lnc-ARL6IP1	LNCipedia lc	lnc-ARL6IP1	NONHSAT14	chr16	18856338	18861487	-
TC1600001836.oe.1	-2.11	-1.08	2.11	down	9.05	10.13	lnc-CRYM-3	LNCipedia lc	lnc-CRYM-3	NONHSAT14	chr16	21432024	21434455	-
TC1600001856.oe.1	-2.67	-1.42	2.67	down	4.59	6.00	lnc-COG7-2	NONCODE c	lnc-COG7-2	NONHSAT14	chr16	23061406	23064173	-
TC1600002117.oe.1	5.20	2.38	5.20	up	6.62	4.24	lnc-ABCC11	LNCipedia lc	lnc-ABCC11	NONHSAT14	chr16	48084055	48086640	-
TC1600002118.oe.1	2.79	1.48	2.79	up	5.56	4.08	lnc-ABCC11	NONCODE c	lnc-ABCC11	NONHSAT14	chr16	48087166	48091238	-
TC1600002409.oe.1	-2.10	-1.07	2.10	down	3.16	4.24	lnc-ZNF19-2	LNCipedia lc	lnc-ZNF19-2	NONHSAT14	chr16	71456782	71462392	-
TC1600002417.oe.1	2.32	1.21	2.32	up	6.59	5.38	lnc-ZNF821-	NONCODE c	lnc-ZNF821-	NONHSAT14	chr16	71789323	71809084	-
TC1600002451.oe.1	-2.40	-1.27	2.40	down	6.45	7.72	lnc-CLEC18E	NONCODE c	lnc-CLEC18E	NONHSAT14	chr16	74377490	74377819	-
TC1600002488.oe.1	-3.13	-1.65	3.13	down	2.58	4.23	lnc-ADAMTS	LNCipedia lc	lnc-ADAMTS	NONHSAT14	chr16	76994308	77008307	-
TC1600002565.oe.1	2.05	1.03	2.05	up	4.14	3.10	lnc-COTL1-2	LNCipedia lc	lnc-COTL1-2	---	chr16	84778324	84784278	-
TC1600002582.oe.1	2.05	1.03	2.05	up	5.69	4.65	lnc-C16orf74	NONCODE c	lnc-C16orf74	NONHSAT14	chr16	85779398	85781223	-
TC16000229.hg.4	-2.15	-1.10	2.15	down	7.96	9.07	CRYM-AS1	CRYM antise	---	---	chr16	21300849	21318591	+
TC1700000002.oe.1	2.22	1.15	2.22	up	6.03	4.88	lnc-C17orf97	NONCODE c	lnc-C17orf97	NONHSAT14	chr17	206418	206810	+
TC1700000063.oe.1	-2.44	-1.29	2.44	down	5.80	7.09	RP11-135N5	N/A	---	---	chr17	2639297	2642418	+
TC1700000104.oe.1	2.38	1.25	2.38	up	10.26	9.01	lnc-RNF167-	NONCODE c	lnc-RNF167-	NONHSAT14	chr17	4940046	4940836	+
TC1700000162.oe.1	2.29	1.20	2.29	up	8.78	7.58	lnc-SLC35G6	NONCODE c	lnc-SLC35G6	NONHSAT14	chr17	7502085	7502724	+
TC1700000166.oe.1	2.07	1.05	2.07	up	10.13	9.08	OTTHUMG00	small nucleo	lnc-SENP3-1	NONHSAT14	chr17	7572706	7582024	+
TC1700000331.oe.1	2.15	1.10	2.15	up	7.40	6.30	lnc-MYO15A	NONCODE c	lnc-MYO15A	NONHSAT14	chr17	18087961	18099221	+
TC1700000395.oe.1	-2.34	-1.23	2.34	down	7.15	8.37	lnc-AC11598	LNCipedia lc	lnc-AC11598	NONHSAT14	chr17	19672352	19677595	+
TC1700000428.oe.1	2.14	1.09	2.14	up	6.86	5.76	lnc-KCNJ12-	NONCODE c	lnc-KCNJ12-	NONHSAT05	chr17	21284710	21314227	+
TC1700000470.oe.1	2.71	1.44	2.71	up	5.39	3.95	lnc-KSR1-1	NONCODE c	lnc-KSR1-1:1	NONHSAT05	chr17	27623535	27626435	+
TC1700000525.oe.1	-3.39	-1.76	3.39	down	8.07	9.83	lnc-TAOK1-3	LNCipedia lc	lnc-TAOK1-3	---	chr17	29544715	29545717	+
TC1700000526.oe.1	-2.98	-1.58	2.98	down	8.46	10.04	lnc-TAOK1-4	NONCODE l	lnc-TAOK1-4	NONHSAT05	chr17	29546887	29552359	+
TC1700000542.oe.1	-2.24	-1.16	2.24	down	11.52	12.68	lnc-GOSR1-	NONCODE c	lnc-GOSR1-	NONHSAT05	chr17	30438985	30444144	+
TC1700000630.oe.1	-2.10	-1.07	2.10	down	11.37	12.44	lnc-CCL2-9	LNCipedia lc	lnc-CCL2-9:1	---	chr17	34083580	34083847	+
TC1700000822.oe.1	2.21	1.14	2.21	up	14.98	13.84	lnc-AOC2-1	LNCipedia lc	lnc-AOC2-1	NONHSAT05	chr17	42833418	42834804	+
TC1700000937.oe.1	2.12	1.08	2.12	up	6.45	5.36	lnc-RP11-29	LNCipedia lc	lnc-RP11-29	NONHSAT05	chr17	47369616	47374638	+
TC1700000956.oe.1	-2.36	-1.24	2.36	down	3.35	4.59	lnc-SNX11-9	LNCipedia lc	lnc-SNX11-9	---	chr17	48148329	48149933	+
TC1700000981.oe.1	2.24	1.16	2.24	up	8.11	6.95	lnc-B4GALN	LNCipedia lc	lnc-B4GALN	NONHSAT05	chr17	49051815	49055155	+
TC1700001005.oe.1	2.41	1.27	2.41	up	10.01	8.74	lnc-PDK2-2	NONCODE c	lnc-PDK2-2:	NONHSAT05	chr17	50077400	50079016	+
TC1700001034.oe.1	-2.23	-1.16	2.23	down	11.32	12.48	lnc-ABCC3-2	NONCODE c	lnc-ABCC3-2	NONHSAT05	chr17	50736635	50741247	+
TC1700001035.oe.1	-2.43	-1.28	2.43	down	12.41	13.69	lnc-ABCC3-3	NONCODE c	lnc-ABCC3-3	NONHSAT05	chr17	50743672	50745858	+
TC1700001038.oe.1	-2.04	-1.03	2.04	down	4.07	5.10	lnc-ABCC3-6	LNCipedia lc	lnc-ABCC3-6	NONHSAT05	chr17	50753056	50756213	+
TC1700001061.oe.1	-2.22	-1.15	2.22	down	5.35	6.51	lnc-TOM1L1	LNCipedia lc	lnc-TOM1L1	---	chr17	54763265	54763520	+

TC1700001170.oe.1	3.77	1.92	3.77	up	10.95	9.04	lnc-C17orf82	LNCipedia lo	lnc-C17orf82	NONHSAT05	chr17	61401773	61403163	+
TC1700001227.oe.1	2.45	1.29	2.45	up	6.31	5.02	lnc-AC03748	LNCipedia lo	lnc-AC03748	NONHSAT05	chr17	64814985	64822052	+
TC1700001258.oe.1	2.01	1.01	2.01	up	10.93	9.92	lnc-NOL11-4	NONCODE (	lnc-NOL11-4	NONHSAT05	chr17	67532905	67632421	+
TC1700001282.oe.1	-2.17	-1.12	2.17	down	5.49	6.60	lnc-AMZ2-2	LNCipedia lo	lnc-AMZ2-2	NONHSAT05	chr17	68267029	68267396	+
TC1700001334.oe.1	2.73	1.45	2.73	up	7.85	6.40	lnc-DNAI2-2	LNCipedia lo	lnc-DNAI2-2	NONHSAT05	chr17	74249365	74250730	+
TC1700001448.oe.1	2.06	1.04	2.06	up	7.41	6.37	lnc-C1QTNF	NONCODE (	lnc-C1QTNF	NONHSAT05	chr17	78855478	78855844	+
TC1700001515.oe.1	2.01	1.00	2.01	up	7.95	6.94	lnc-HGS-1	LNCipedia lo	lnc-HGS-1:1	NONHSAT05	chr17	81703501	81704420	+
TC1700001520.oe.1	2.09	1.06	2.09	up	9.84	8.78	NONHSAG0	NONCODE (	lnc-GCGR-1	NONHSAT05	chr17	81843165	81843958	+
TC1700001546.oe.1	2.44	1.29	2.44	up	9.45	8.16	lnc-TEX19-1	LNCipedia lo	lnc-TEX19-1	NONHSAT05	chr17	82236474	82237416	+
TC1700001562.oe.1	2.49	1.31	2.49	up	11.79	10.48	lnc-NARF-5	LNCipedia lo	lnc-NARF-5:	NONHSAT05	chr17	82582775	82586043	+
TC1700001692.oe.1	2.12	1.09	2.12	up	10.46	9.37	lnc-ANKFY1	LNCipedia lo	lnc-ANKFY1	NONHSAT14	chr17	4064799	4072827	-
TC1700001703.oe.1	2.51	1.33	2.51	up	7.72	6.39	lnc-GGT6-2	NONCODE (	lnc-GGT6-2:	NONHSAT14	chr17	4539439	4542100	-
TC1700001704.oe.1	2.58	1.37	2.58	up	7.57	6.21	lnc-GGT6-1	LNCipedia lo	lnc-GGT6-1:	NONHSAT14	chr17	4552183	4555358	-
TC1700001757.oe.1	-2.19	-1.13	2.19	down	3.34	4.47	lnc-AC02776	LNCipedia lo	lnc-AC02776	NONHSAT14	chr17	6980856	6981165	-
TC1700001782.oe.1	3.46	1.79	3.46	up	6.13	4.34	lnc-SOX15-2	NONCODE (	lnc-SOX15-2	NONHSAT14	chr17	7591442	7592357	-
TC1700001808.oe.1	2.15	1.11	2.15	up	6.44	5.33	lnc-AURKB-1	NONCODE (	lnc-AURKB-1	NONHSAT14	chr17	8230268	8230933	-
TC1700002011.oe.1	-2.38	-1.25	2.38	down	3.89	5.15	lnc-SLC47A2	LNCipedia lo	lnc-SLC47A2	NONHSAT14	chr17	19639128	19640550	-
TC1700002215.oe.1	-2.07	-1.05	2.07	down	4.16	5.21	lnc-ACCN1-	LNCipedia lo	lnc-ACCN1-	NONHSAT05	chr17	34219118	34251909	-
TC1700002225.oe.1	2.04	1.03	2.04	up	4.30	3.27	RP5-837J1.4	LNCipedia lo	lnc-RAD51D	NONHSAT05	chr17	35073831	35074374	-
TC1700002303.oe.1	-2.16	-1.11	2.16	down	3.65	4.77	lnc-SRCIN1-	LNCipedia lo	lnc-SRCIN1-	NONHSAT05	chr17	38636682	38637016	-
TC1700002464.oe.1	2.04	1.03	2.04	up	8.99	7.96	lnc-SLC25A3	LNCipedia lo	lnc-SLC25A3	NONHSAT05	chr17	44319614	44319996	-
TC1700002483.oe.1	-2.17	-1.12	2.17	down	2.94	4.06	lnc-DCAKD-	LNCipedia lo	lnc-DCAKD-	---	chr17	45004464	45004665	-
TC1700002515.oe.1	-2.53	-1.34	2.53	down	6.97	8.31	lnc-KIAA126	LNCipedia lo	lnc-KIAA126	---	chr17	45941539	45941745	-
TC1700002622.oe.1	-2.64	-1.40	2.64	down	7.32	8.72	lnc-TOB1-4	LNCipedia lo	lnc-TOB1-4:	NONHSAT05	chr17	50964149	50965494	-
TC1700002628.oe.1	-2.07	-1.05	2.07	down	3.97	5.01	RP11-421E1	LNCipedia lo	lnc-SPAG9-2	NONHSAT05	chr17	51249577	51251748	-
TC1700002663.oe.1	2.17	1.12	2.17	up	8.08	6.96	lnc-COIL-7	LNCipedia lo	lnc-COIL-7:1	NONHSAT05	chr17	56890521	56891526	-
TC1700002709.oe.1	-2.38	-1.25	2.38	down	3.46	4.71	RP11-567L7	NONCODE (	lnc-SKA2-2:1	NONHSAT05	chr17	59400488	59403303	-
TC1700002777.oe.1	2.05	1.04	2.05	up	6.82	5.79	lnc-ERN1-1	NONCODE (	lnc-ERN1-1:	NONHSAT05	chr17	64039150	64041843	-
TC1700002843.oe.1	-2.16	-1.11	2.16	down	3.42	4.53	lnc-ABCA10	LNCipedia lo	lnc-ABCA10	NONHSAT05	chr17	69247376	69249141	-
TC1700002921.oe.1	2.80	1.48	2.80	up	6.72	5.24	lnc-GRB2-2	NONCODE (	lnc-GRB2-2:	NONHSAT05	chr17	75506743	75507635	-
TC1700002935.oe.1	2.05	1.03	2.05	up	5.83	4.80	lnc-WBP2-2	NONCODE (	lnc-WBP2-2	NONHSAT05	chr17	75840755	75841156	-
TC1700002969.oe.1	2.01	1.00	2.01	up	11.15	10.14	lnc-JMJD6-2	NONCODE (	lnc-JMJD6-2	NONHSAT05	chr17	76679571	76710974	-
TC1700002996.oe.1	2.56	1.36	2.56	up	6.35	5.00	lnc-TK1-2	LNCipedia lo	lnc-TK1-2:1	NONHSAT05	chr17	78223982	78224432	-
TC1700003006.oe.1	3.00	1.58	3.00	up	8.21	6.63	lnc-TIMP2-1	LNCipedia lo	lnc-TIMP2-1	NONHSAT05	chr17	78835279	78840786	-
TC1700003013.oe.1	2.02	1.02	2.02	up	10.77	9.76	lnc-LGALS3B	LNCipedia lo	lnc-LGALS3B	NONHSAT05	chr17	78993784	78995249	-
TC1700003087.oe.1	2.16	1.11	2.16	up	9.97	8.85	lnc-P4HB-2	LNCipedia lo	lnc-P4HB-2:	NONHSAT05	chr17	81869523	81870265	-
TC1700003101.oe.1	2.58	1.37	2.58	up	6.78	5.41	lnc-RFNG-2	LNCipedia lo	lnc-RFNG-2:	NONHSAT05	chr17	82057880	82063477	-
TC17000121.hg.4	2.11	1.08	2.11	up	4.23	3.16	SCARNA21	small Cajal b	---	---	chr17	7906122	7906260	+
TC17000179.hg.4	-2.19	-1.13	2.19	down	2.76	3.89	MEIS3P1	Meis homeo	---	---	chr17	15786850	15789705	+
TC17000940.hg.4	2.17	1.12	2.17	up	7.79	6.67	MAFG-AS1	MAFG antise	---	---	chr17	81927829	81930753	+
TC1800000014.oe.1	-2.21	-1.15	2.21	down	3.66	4.80	RP11-806L2	NONCODE (	lnc-TYMS-2:	NONHSAT05	chr18	706523	707648	+
TC1800000029.oe.1	-2.12	-1.09	2.12	down	2.63	3.72	lnc-NDC80-	NONCODE (	lnc-NDC80-	NONHSAT05	chr18	1963907	1972875	+
TC1800000055.oe.1	-2.32	-1.22	2.32	down	4.00	5.22	lnc-AP00247	LNCipedia lo	lnc-AP00247	---	chr18	3441009	3441248	+
TC1800000134.oe.1	-2.49	-1.32	2.49	down	5.12	6.43	lnc-TXNDC2	LNCipedia lo	lnc-TXNDC2	---	chr18	9939991	9940343	+
TC1800000207.oe.1	2.36	1.24	2.36	up	6.70	5.46	lnc-MC5R-1	NONCODE l	lnc-MC5R-1	NONHSAT05	chr18	14201138	14221409	+
TC1800000222.oe.1	2.20	1.14	2.20	up	6.60	5.46	lnc-SNRPD1	LNCipedia lo	lnc-SNRPD1	NONHSAT05	chr18	21449741	21451370	+
TC1800000352.oe.1	-2.06	-1.04	2.06	down	3.18	4.22	lnc-GALNT1	LNCipedia lo	lnc-GALNT1	---	chr18	35737684	35737896	+
TC1800000497.oe.1	-6.56	-2.71	6.56	down	6.49	9.21	lnc-C18orf26	LNCipedia lo	lnc-C18orf26	NONHSAT05	chr18	54892644	54895106	+
TC1800000611.oe.1	2.06	1.04	2.06	up	4.63	3.58	lnc-CCDC10	LNCipedia lo	lnc-CCDC10	NONHSAT05	chr18	68455951	68456877	+

TC1800000615.oe.1	2.37	1.24	2.37	up	4.51	3.26	lnc-DOK6-6	LNCipedia l	lnc-DOK6-6	NONHSAT0	chr18	68846395	68874965	+
TC1800000627.oe.1	-2.37	-1.25	2.37	down	7.59	8.83	lnc-DOK6-3	LNCipedia l	lnc-DOK6-3	NONHSAT0	chr18	70327517	70330195	+
TC1800000675.oe.1	2.37	1.25	2.37	up	5.77	4.53	lnc-TSHZ1-6	NONCODE (	lnc-TSHZ1-6	NONHSAT0	chr18	74877198	74905938	+
TC1800000787.oe.1	-2.06	-1.04	2.06	down	2.98	4.02	lnc-DLGAP1	LNCipedia l	lnc-DLGAP1	NONHSAT0	chr18	4161046	4162646	-
TC1800000825.oe.1	-2.04	-1.03	2.04	down	3.01	4.04	lnc-PPP4R1-	LNCipedia l	lnc-PPP4R1-	---	chr18	9281111	9324470	-
TC1800000849.oe.1	2.21	1.14	2.21	up	6.13	4.99	lnc-RP11-67	LNCipedia l	lnc-RP11-67	NONHSAT0	chr18	10794070	10795004	-
TC1800000862.oe.1	2.42	1.27	2.42	up	10.70	9.43	lnc-MPPE1-	LNCipedia l	lnc-MPPE1-	NONHSAT0	chr18	11851414	11852751	-
TC1800000960.oe.1	2.03	1.02	2.03	up	4.93	3.90	lnc-NPC1-3	LNCipedia l	lnc-NPC1-3:	---	chr18	23430389	23430611	-
TC1800000961.oe.1	2.31	1.21	2.31	up	11.97	10.76	lnc-ANKRD2	NONCODE (	lnc-ANKRD2	NONHSAT0	chr18	23533202	23534560	-
TC1800000962.oe.1	2.68	1.42	2.68	up	8.56	7.13	lnc-ANKRD2	LNCipedia l	lnc-ANKRD2	---	chr18	23539241	23539565	-
TC1800001020.oe.1	-2.17	-1.12	2.17	down	4.75	5.87	lnc-MCART2	NONCODE (	lnc-MCART2	NONHSAT0	chr18	31830498	31833406	-
TC1800001063.oe.1	-2.15	-1.10	2.15	down	15.33	16.44	lnc-SLC39A6	LNCipedia l	lnc-SLC39A6	---	chr18	36238967	36239177	-
TC1800001118.oe.1	2.29	1.20	2.29	up	4.56	3.36	lnc-EPG5-4	LNCipedia l	lnc-EPG5-4:	NONHSAT0	chr18	45991936	45993974	-
TC1800001203.oe.1	-2.80	-1.49	2.80	down	3.88	5.37	lnc-AC0063(	LNCipedia l	lnc-AC0063(	NONHSAT0	chr18	56599068	56600986	-
TC1800001211.oe.1	2.36	1.24	2.36	up	4.86	3.62	RP11-706P1	N/A	---	---	chr18	57588611	57589091	-
TC1800001294.oe.1	-2.15	-1.10	2.15	down	3.05	4.16	lnc-CD226-(	LNCipedia l	lnc-CD226-(	NONHSAT0	chr18	68697376	68700423	-
TC1800001376.oe.1	-2.09	-1.07	2.09	down	3.70	4.77	RP11-4B16.1	N/A	---	---	chr18	77108284	77110537	-
TC1800001377.oe.1	-2.98	-1.57	2.98	down	5.98	7.56	lnc-ZNF516-	LNCipedia l	lnc-ZNF516-	---	chr18	77125605	77127925	-
TC18000020.hg.4	2.81	1.49	2.81	up	8.32	6.83	DLGAP1-AS	DLGAP1 anti	---	---	chr18	3594114	3598352	+
TC1900000113.oe.1	2.34	1.23	2.34	up	9.48	8.25	lnc-GNA15-	NONCODE (	lnc-GNA15-	NONHSAT0	chr19	3094649	3115428	+
TC1900000116.oe.1	2.33	1.22	2.33	up	6.92	5.70	lnc-CELF5-1	LNCipedia l	lnc-CELF5-1	NONHSAT0	chr19	3207466	3209571	+
TC1900000202.oe.1	-2.21	-1.15	2.21	down	7.77	8.91	CTD-2396E7	N/A	---	---	chr19	6469465	6470152	+
TC1900000299.oe.1	3.38	1.76	3.38	up	13.75	11.99	lnc-ICAM4-:	LNCipedia l	lnc-ICAM4-:	NONHSAT0	chr19	10285485	10285698	+
TC1900000322.oe.1	4.38	2.13	4.38	up	9.90	7.77	lnc-SMARCA	NONCODE (	lnc-SMARCA	NONHSAT0	chr19	11106356	11107515	+
TC1900000323.oe.1	2.54	1.34	2.54	up	6.48	5.14	lnc-SMARCA	LNCipedia l	lnc-SMARCA	NONHSAT0	chr19	11110713	11113481	+
TC1900000465.oe.1	2.13	1.09	2.13	up	6.54	5.45	lnc-MYO9B-	LNCipedia l	lnc-MYO9B-	NONHSAT0	chr19	17215392	17217489	+
TC1900000471.oe.1	2.07	1.05	2.07	up	8.86	7.80	lnc-MRPL34	LNCipedia l	lnc-MRPL34	NONHSAT0	chr19	17315772	17319719	+
TC1900000574.oe.1	-2.01	-1.01	2.01	down	5.43	6.43	lnc-ZNF714-	NONCODE (	lnc-ZNF714-	NONHSAT0	chr19	21123817	21124188	+
TC1900000649.oe.1	-2.08	-1.06	2.08	down	12.90	13.95	NONHSAG0	NONCODE (	lnc-VSTM2B	NONHSAT0	chr19	28606688	28615229	+
TC1900000726.oe.1	-2.11	-1.08	2.11	down	4.31	5.39	lnc-PDCD2L	LNCipedia l	lnc-PDCD2L	NONHSAT0	chr19	34389107	34390806	+
TC1900000728.oe.1	2.14	1.10	2.14	up	6.91	5.82	lnc-PDCD2L	NONCODE (	lnc-PDCD2L	NONHSAT0	chr19	34404827	34426167	+
TC1900000730.oe.1	-2.63	-1.40	2.63	down	3.54	4.94	lnc-WTIP-1	NONCODE (	lnc-WTIP-1:	NONHSAT0	chr19	34542666	34543390	+
TC1900000809.oe.1	-2.37	-1.25	2.37	down	10.46	11.70	lnc-CAPNS1	LNCipedia l	lnc-CAPNS1	NONHSAT0	chr19	36228459	36235868	+
TC1900001154.oe.1	-2.05	-1.04	2.05	down	11.37	12.41	lnc-CD37-2	LNCipedia l	lnc-CD37-2:	NONHSAT0	chr19	49275119	49275420	+
TC1900001351.oe.1	2.01	1.01	2.01	up	6.57	5.56	lnc-CCDC10	LNCipedia l	lnc-CCDC10	NONHSAT0	chr19	55654132	55655019	+
TC1900001358.oe.1	-2.09	-1.07	2.09	down	3.20	4.27	lnc-EPN1-4	NONCODE (	lnc-EPN1-4:	NONHSAT0	chr19	55700049	55701219	+
TC1900001394.oe.1	-2.92	-1.54	2.92	down	6.68	8.22	lnc-ZNF460-	LNCipedia l	lnc-ZNF460-	NONHSAT0	chr19	57304305	57308562	+
TC1900001652.oe.1	3.12	1.64	3.12	up	5.33	3.69	lnc-GPR108-	LNCipedia l	lnc-GPR108-	NONHSAT0	chr19	6712280	6713315	-
TC1900001818.oe.1	2.09	1.06	2.09	up	8.11	7.05	lnc-LYL1-2	NONCODE (	lnc-LYL1-2:1	NONHSAT0	chr19	13112955	13116733	-
TC1900001900.oe.1	2.10	1.07	2.10	up	7.12	6.04	lnc-MED26-	NONCODE (	lnc-MED26-	NONHSAT0	chr19	16555349	16566591	-
TC1900001974.oe.1	2.05	1.03	2.05	up	4.90	3.86	lnc-LPAR2-2	NONCODE (	lnc-LPAR2-2	NONHSAT0	chr19	19588092	19588416	-
TC1900001977.oe.1	2.02	1.02	2.02	up	7.34	6.33	lnc-GMIP-2	LNCipedia l	lnc-GMIP-2:	NONHSAT0	chr19	19653777	19655451	-
TC1900002057.oe.1	-2.38	-1.25	2.38	down	5.55	6.80	lnc-ZNF91-2	LNCipedia l	lnc-ZNF91-2	NONHSAT0	chr19	23322938	23324418	-
TC1900002169.oe.1	-2.08	-1.06	2.08	down	5.30	6.36	lnc-AC0209(	LNCipedia l	lnc-AC0209(	---	chr19	35042466	35042697	-
TC1900002264.oe.1	-3.03	-1.60	3.03	down	10.58	12.18	lnc-ECH1-4	NONCODE (	lnc-ECH1-4:	NONHSAT0	chr19	38801883	38803651	-
TC1900002265.oe.1	-3.47	-1.79	3.47	down	8.19	9.99	lnc-ECH1-3	NONCODE (	lnc-ECH1-3:	NONHSAT0	chr19	38806461	38812945	-
TC1900002328.oe.1	2.05	1.04	2.05	up	8.80	7.76	lnc-B9D2-2	NONCODE (	lnc-B9D2-2:	NONHSAT0	chr19	41332055	41344883	-
TC1900002421.oe.1	-2.06	-1.05	2.06	down	3.76	4.81	AC006126.4	LNCipedia l	lnc-EXOC3L	---	chr19	45238632	45245370	-
TC1900002476.oe.1	2.16	1.11	2.16	up	8.01	6.89	lnc-TMEM16	LNCipedia l	lnc-TMEM16	NONHSAT0	chr19	47093749	47094487	-

TC1900002597.oe.1	-2.17	-1.12	2.17	down	3.53	4.65	CTB-147C22	N/A	---	---	chr19	50962197	50962781	-
TC1900002696.oe.1	-3.80	-1.92	3.80	down	4.75	6.68	NONHSAG0	LNCipedia lo	lnc-LENG9-1	NONHSAT14	chr19	54438010	54438346	-
TC1900002698.oe.1	-2.20	-1.14	2.20	down	5.61	6.74	NONHSAG0	LNCipedia lo	lnc-LENG9-1	NONHSAT14	chr19	54438665	54439544	-
TC1900002749.oe.1	-2.38	-1.25	2.38	down	5.64	6.90	AC006116.19	NONCODE c	lnc-ZNF582-	NONHSAT0	chr19	56311928	56312486	-
TC19000570.hg.4	-11.21	-3.49	11.21	down	5.98	9.47	CYP2B7P	cytochrome	---	---	chr19	40924265	40950660	+
TC19000679.hg.4	2.04	1.03	2.04	up	5.48	4.45	NAPA-AS1	NAPA antise	---	---	chr19	47484282	47501597	+
TC19000694.hg.4	-2.08	-1.06	2.08	down	3.68	4.74	SNAR-C3	small ILF3/N	---	---	chr19	47950296	47950414	+
TC19000708.hg.4	-2.48	-1.31	2.48	down	4.14	5.45	SEC1P	secretory blo	---	---	chr19	48638039	48682245	+
TC19001130.hg.4	-2.80	-1.49	2.80	down	5.67	7.16	RAB11B-AS1	RAB11B anti	---	---	chr19	8374376	8390691	-
TC2000000038.oe.1	2.24	1.16	2.24	up	7.74	6.57	lnc-TMC2-3	NONCODE c	lnc-TMC2-3	NONHSAT0	chr20	2652633	2653820	+
TC2000000177.oe.1	2.06	1.04	2.06	up	13.06	12.02	lnc-POLR3F-	LNCipedia lo	lnc-POLR3F-	NONHSAT0	chr20	18510977	18515984	+
TC2000000191.oe.1	-2.16	-1.11	2.16	down	3.55	4.66	lnc-INSM1-2	LNCipedia lo	lnc-INSM1-2	NONHSAT0	chr20	20354545	20355962	+
TC2000000285.oe.1	2.43	1.28	2.43	up	11.36	10.08	lnc-REM1-2	NONCODE c	lnc-REM1-2	NONHSAT0	chr20	31514655	31538228	+
TC2000000323.oe.1	-2.26	-1.18	2.26	down	3.84	5.02	lnc-C20orf14	LNCipedia lo	lnc-C20orf14	---	chr20	33539090	33539334	+
TC2000000364.oe.1	2.12	1.08	2.12	up	6.96	5.87	lnc-C20orf24	NONCODE c	lnc-C20orf24	NONHSAT0	chr20	36578807	36592298	+
TC2000000439.oe.1	-2.42	-1.27	2.42	down	7.46	8.74	lnc-SGK2-1	LNCipedia lo	lnc-SGK2-1:	NONHSAT0	chr20	43564718	43588237	+
TC2000000552.oe.1	3.11	1.64	3.11	up	7.78	6.15	lnc-SNAI1-4	LNCipedia lo	lnc-SNAI1-4	NONHSAT0	chr20	50191750	50192009	+
TC2000000682.oe.1	2.59	1.37	2.59	up	8.85	7.47	lnc-OSBPL2-	LNCipedia lo	lnc-OSBPL2-	NONHSAT0	chr20	62304159	62306734	+
TC2000000856.oe.1	2.01	1.01	2.01	up	10.53	9.52	lnc-JAG1-7	LNCipedia lo	lnc-JAG1-7:	NONHSAT0	chr20	10673629	10673976	-
TC2000000888.oe.1	-3.28	-1.71	3.28	down	6.27	7.99	lnc-SEL1L2-	LNCipedia lo	lnc-SEL1L2-	NONHSAT0	chr20	14328922	14337614	-
TC2000001019.oe.1	-2.06	-1.04	2.06	down	2.71	3.75	lnc-FAM182	LNCipedia lo	lnc-FAM182	---	chr20	25722019	25811201	-
TC2000001072.oe.1	2.04	1.03	2.04	up	7.52	6.50	lnc-E2F1-1	LNCipedia lo	lnc-E2F1-1:1	NONHSAT0	chr20	33669143	33674420	-
TC2000001107.oe.1	2.03	1.02	2.03	up	12.36	11.34	lnc-FAM83C	NONCODE c	lnc-FAM83C	NONHSAT0	chr20	35280074	35284702	-
TC2000001284.oe.1	2.07	1.05	2.07	up	3.72	2.66	lnc-PTGIS-2	LNCipedia lo	lnc-PTGIS-2	---	chr20	49474123	49474911	-
TC20000751.hg.4	-2.24	-1.16	2.24	down	2.67	3.83	DEFB122	defensin, be	---	---	chr20	31421436	31429180	-
TC2100000339.oe.1	-2.01	-1.01	2.01	down	2.98	3.98	lnc-BACE2-2	LNCipedia lo	lnc-BACE2-2	---	chr21	40943256	40946203	+
TC2100000343.oe.1	3.44	1.78	3.44	up	6.40	4.61	lnc-FAM3B-	NONCODE c	lnc-FAM3B-	NONHSAT0	chr21	41225476	41241903	+
TC2100000344.oe.1	4.42	2.15	4.42	up	9.38	7.24	lnc-FAM3B-	LNCipedia lo	lnc-FAM3B-	NONHSAT0	chr21	41245959	41275538	+
TC2100000345.oe.1	2.23	1.16	2.23	up	5.84	4.68	lnc-FAM3B-	NONCODE c	lnc-FAM3B-	NONHSAT0	chr21	41252465	41257326	+
TC2100000367.oe.1	-2.33	-1.22	2.33	down	6.08	7.30	lnc-UBASH3	NONCODE c	lnc-UBASH3	NONHSAT0	chr21	42292533	42293943	+
TC2100000463.oe.1	2.39	1.26	2.39	up	5.54	4.28	lnc-COL18A	gene_id XLC	lnc-COL18A	NONHSAT0	chr21	45370948	45371927	+
TC2100000513.oe.1	-3.29	-1.72	3.29	down	15.34	17.06	CH507-513H	N/A	---	---	chr21	8210384	8211306	-
TC2100000514.oe.1	-3.71	-1.89	3.71	down	15.28	17.17	CH507-513H	N/A	---	---	chr21	8254592	8255514	-
TC2100000515.oe.1	-3.82	-1.94	3.82	down	15.08	17.02	CH507-513H	N/A	---	---	chr21	8393419	8394341	-
TC2100000516.oe.1	-3.07	-1.62	3.07	down	15.45	17.07	CH507-513H	N/A	---	---	chr21	8437629	8438551	-
TC2100000518.oe.1	-2.34	-1.23	2.34	down	13.69	14.92	lnc-TPTE-3	NONCODE c	lnc-TPTE-3:1	NONHSAT0	chr21	8987561	8988887	-
TC2100000550.oe.1	-2.66	-1.41	2.66	down	5.21	6.62	lnc-LIPI-5	LNCipedia lo	lnc-LIPI-5:1	NONHSAT14	chr21	13916917	13917802	-
TC2100000601.oe.1	-2.58	-1.37	2.58	down	2.65	4.01	lnc-TMPRSS	LNCipedia lo	lnc-TMPRSS	NONHSAT0	chr21	18574969	18578511	-
TC2100000634.oe.1	2.53	1.34	2.53	up	4.47	3.13	lnc-MRPL39	LNCipedia lo	lnc-MRPL39	---	chr21	23226412	23243239	-
TC2100000653.oe.1	-2.37	-1.24	2.37	down	2.87	4.11	lnc-MRPL39	LNCipedia lo	lnc-MRPL39	NONHSAT0	chr21	25430145	25430357	-
TC2100000852.oe.1	-2.38	-1.25	2.38	down	4.30	5.55	lnc-RIPK4-6	NONCODE c	lnc-RIPK4-6:	NONHSAT0	chr21	41465890	41468837	-
TC2100000872.oe.1	11.58	3.53	11.58	up	9.01	5.48	lnc-TFF1-1	LNCipedia lo	lnc-TFF1-1:1	NONHSAT0	chr21	42362282	42366535	-
TC2100000950.oe.1	2.04	1.03	2.04	up	10.54	9.51	lnc-SLC19A1	NONCODE c	lnc-SLC19A1	NONHSAT0	chr21	45483805	45484746	-
TC21000116.hg.4	2.88	1.52	2.88	up	5.26	3.73	C21orf119	chromosome	---	---	chr21	32393130	32393960	+
TC2200000011.oe.1	-2.21	-1.15	2.21	down	6.38	7.53	LL22NC03-N	LNCipedia lo	lnc-AP00052	---	chr22	15823197	15823890	+
TC2200000270.oe.1	-2.03	-1.02	2.03	down	3.35	4.37	lnc-MYO18E	LNCipedia lo	lnc-MYO18E	---	chr22	25606234	25616343	+
TC2200000455.oe.1	2.01	1.01	2.01	up	4.98	3.98	lnc-KCTD17-	LNCipedia lo	lnc-KCTD17-	NONHSAT0	chr22	37300882	37303295	+
TC2200000466.oe.1	2.12	1.08	2.12	up	11.59	10.51	lnc-NOL12.1	LNCipedia lo	lnc-NOL12.1	NONHSAT0	chr22	37675652	37677228	+
TC2200000580.oe.1	3.57	1.84	3.57	up	6.75	4.91	lnc-SERHL2-	NONCODE c	lnc-SERHL2-	NONHSAT0	chr22	42614914	42616588	+

TC2200000632.oe.1	-2.01	-1.01	2.01	down	4.85	5.85	lnc-FBLN1-3	LNCipedia lc	lnc-FBLN1-3	---	chr22	45724103	45752075	+
TC2200000749.oe.1	2.08	1.06	2.08	up	9.29	8.24	lnc-ATP6V1	NONCODE	lnc-ATP6V1	NONHSAT0	chr22	17592137	17628822	-
TC2200000855.oe.1	-2.02	-1.01	2.02	down	4.57	5.58	LL22NC03-2	N/A	---	---	chr22	22283928	22287220	-
TC2200000987.oe.1	2.17	1.12	2.17	up	12.55	11.44	lnc-TBC1D1	LNCipedia lc	lnc-TBC1D1	NONHSAT0	chr22	30342547	30346489	-
TC2200001003.oe.1	-2.37	-1.24	2.37	down	5.66	6.90	RP3-430N8.	LNCipedia lc	lnc-MORC2-	NONHSAT0	chr22	30977516	30977858	-
TC2200001074.oe.1	2.17	1.12	2.17	up	8.21	7.10	lnc-FOXRED	LNCipedia lc	lnc-FOXRED	NONHSAT0	chr22	36510856	36511641	-
TC2200001091.oe.1	3.53	1.82	3.53	up	7.66	5.84	lnc-SSTR3-2	LNCipedia lc	lnc-SSTR3-2	NONHSAT0	chr22	37225264	37225995	-
TC2200001106.oe.1	2.28	1.19	2.28	up	6.39	5.20	lnc-C22orf2	NONCODE	lnc-C22orf2	NONHSAT1	chr22	37832676	37840234	-
TC2200001165.oe.1	2.66	1.41	2.66	up	7.23	5.82	lnc-PMM1-3	NONCODE	lnc-PMM1-3	NONHSAT0	chr22	41532625	41544386	-
TC2200001219.oe.1	2.18	1.13	2.18	up	4.69	3.57	lnc-LDOC1L	LNCipedia lc	lnc-LDOC1L	NONHSAT0	chr22	44514717	44520948	-
TC22000220.hg.4	-2.15	-1.11	2.15	down	11.32	12.43	TUG1	taurine upre	---	---	chr22	30969648	30979394	+
TC22000515.hg.4	2.04	1.03	2.04	up	8.71	7.68	PI4KAP1//PI	phosphatidy	---	---	chr22	18533671	18577948	-
TC22000847.hg.4	2.17	1.11	2.17	up	6.42	5.31	RRP7B//RNU	RNA, U6 sm	---	---	chr22	42555223	42582038	-
