# Supplementary material for: Differentially Expressed Long Noncoding RNAs Involved in FUBP1 Promoting Hepatocellular Carcinoma Cells Proliferation
Source: Biomed Res Int. 2021 Apr 14;2021:6664519. doi: 10.1155/2021/6664519 (PMC8063849; doi:10.1155/2021/6664519)
Supplement: Supplementary 3 — Differentially expressed long noncoding RNAs in Huh-7 cells with or without FUBP1 overexpression. [file 6664519.f3.pdf]

Probe Set ID	FC ([Hul	Log FC (	FC (abs)	Regulati	Huh7-FL	Huh7-n	GeneSymbol	Description	lncipediaID	NonCodeID	Chr	start	stop	strand
TC0100000260.oe.1	2.05	1.04	2.05	up	4.39	3.35	lnc-CELA2B-	LNCipedia lc	lnc-CELA2B-	---	chr1	15538858	15541629	+
TC0100000281.oe.1	2.07	1.05	2.07	up	6.58	5.53	lnc-C1orf14	LNCipedia lc	lnc-C1orf14	NONHSAT0	chr1	16440736	16441619	+
TC0100000284.oe.1	3.15	1.66	3.15	up	5.93	4.27	RP4-798A10	LNCipedia lc	lnc-AL35514	NONHSAT0	chr1	16514643	16515754	+
TC0100000412.oe.1	2.01	1.00	2.01	up	6.99	5.98	lnc-C1orf13	NONCODE	lnc-C1orf13	NONHSAT0	chr1	24666555	24669264	+
TC0100000413.oe.1	2.14	1.10	2.14	up	9.27	8.17	lnc-C1orf13	LNCipedia lc	lnc-C1orf13	NONHSAT0	chr1	24669583	24671469	+
TC0100000619.oe.1	-2.32	-1.22	2.32	down	4.42	5.64	lnc-EIF2C3-	LNCipedia lc	lnc-EIF2C3-	---	chr1	36060096	36060737	+
TC0100000673.oe.1	2.10	1.07	2.10	up	4.24	3.18	NONHSAG0	LNCipedia lc	lnc-NDUFS5	NONHSAT0	chr1	39206512	39206957	+
TC0100000709.oe.1	-2.55	-1.35	2.55	down	10.46	11.81	OTTHUMG0	LNCipedia lc	lnc-ZNF643-	NONHSAT0	chr1	40464319	40466767	+
TC0100000713.oe.1	2.05	1.03	2.05	up	8.03	6.99	lnc-DEM1-3	NONCODE	lnc-DEM1-3	NONHSAT0	chr1	40531722	40547785	+
TC0100000714.oe.1	2.62	1.39	2.62	up	7.92	6.53	lnc-DEM1-2	LNCipedia lc	lnc-DEM1-2	---	chr1	40542029	40542308	+
TC0100000757.oe.1	3.20	1.68	3.20	up	8.84	7.16	lnc-MPL-1	NONCODE	lnc-MPL-1:1	NONHSAT0	chr1	43361058	43363066	+
TC0100000762.oe.1	-2.01	-1.01	2.01	down	4.34	5.35	lnc-PTPRF-1	LNCipedia lc	lnc-PTPRF-1	NONHSAT0	chr1	43525187	43553539	+
TC0100001031.oe.1	-2.13	-1.09	2.13	down	4.45	5.54	lnc-FOXD3-	LNCipedia lc	lnc-FOXD3-	NONHSAT0	chr1	63324699	63325126	+
TC0100001034.oe.1	2.05	1.04	2.05	up	8.11	7.07	lnc-EFCAB7-	LNCipedia lc	lnc-EFCAB7-	NONHSAT0	chr1	63523372	63532696	+
TC0100001171.oe.1	2.34	1.23	2.34	up	5.50	4.27	lnc-LPHN2-	LNCipedia lc	lnc-LPHN2-	NONHSAT0	chr1	81992876	81996530	+
TC0100001257.oe.1	2.59	1.37	2.59	up	10.98	9.61	lnc-MTF2-3	LNCipedia lc	lnc-MTF2-3:	NONHSAT0	chr1	93205556	93246901	+
TC0100001258.oe.1	2.16	1.11	2.16	up	8.75	7.64	lnc-DR1-4	LNCipedia lc	lnc-DR1-4:1	NONHSAT0	chr1	93254511	93264949	+
TC0100001301.oe.1	2.40	1.27	2.40	up	10.42	9.16	lnc-RWDD3-	NONCODE	lnc-RWDD3-	NONHSAT0	chr1	96785194	96813447	+
TC0100001326.oe.1	2.32	1.21	2.32	up	4.30	3.09	lnc-SLC35A3	NONCODE	lnc-SLC35A3	NONHSAT0	chr1	99966041	99966379	+
TC0100001344.oe.1	2.22	1.15	2.22	up	5.83	4.68	RP4-549L20	LNCipedia lc	lnc-SLC30A7	---	chr1	1.01E+08	100895356	+
TC0100001372.oe.1	2.54	1.35	2.54	up	6.71	5.37	lnc-AMY2B-	LNCipedia lc	lnc-AMY2B-	NONHSAT0	chr1	1.04E+08	103551263	+
TC0100001419.oe.1	2.14	1.10	2.14	up	4.48	3.38	lnc-SYPL2-1	LNCipedia lc	lnc-SYPL2-1	NONHSAT0	chr1	1.09E+08	109425540	+
TC0100001503.oe.1	-2.06	-1.04	2.06	down	4.31	5.36	lnc-LRIG2-1	gene_id XLC	lnc-LRIG2-1	NONHSAT0	chr1	1.13E+08	113132124	+
TC0100001550.oe.1	-2.70	-1.44	2.70	down	9.14	10.58	lnc-FAM46C	LNCipedia lc	lnc-FAM46C	NONHSAT0	chr1	1.18E+08	117528869	+
TC0100001609.oe.1	2.18	1.12	2.18	up	11.45	10.33	RNVU1-14	RNA, variant	---	---	chr1	1.45E+08	145281462	+
TC0100001683.oe.1	2.15	1.11	2.15	up	8.95	7.84	lnc-RPRD2-	NONCODE	lnc-RPRD2-	NONHSAT0	chr1	1.5E+08	150344375	+
TC0100001711.oe.1	2.07	1.05	2.07	up	5.41	4.37	lnc-TNFAIP8	LNCipedia lc	lnc-TNFAIP8	NONHSAT0	chr1	1.51E+08	151166950	+
TC0100001743.oe.1	-2.08	-1.06	2.08	down	3.10	4.16	NONHSAG0	LNCipedia lc	lnc-OAZ3-4	NONHSAT0	chr1	1.52E+08	152125065	+
TC0100001776.oe.1	2.07	1.05	2.07	up	6.78	5.73	RP11-422P2	LNCipedia lc	lnc-CREB3L4	---	chr1	1.54E+08	153966930	+
TC0100001799.oe.1	2.64	1.40	2.64	up	8.56	7.16	lnc-CKS1B-1	NONCODE	lnc-CKS1B-1	NONHSAT0	chr1	1.55E+08	154979248	+
TC0100001874.oe.1	-2.12	-1.08	2.12	down	3.53	4.62	lnc-APCS-3	LNCipedia lc	lnc-APCS-3:	NONHSAT0	chr1	1.6E+08	159621990	+
TC0100001928.oe.1	-2.93	-1.55	2.93	down	6.90	8.45	lnc-NOS1AP	LNCipedia lc	lnc-NOS1AP	NONHSAT0	chr1	1.62E+08	161960124	+
TC0100001951.oe.1	2.05	1.03	2.05	up	9.61	8.58	lnc-RGS4-4	NONCODE	lnc-RGS4-4:	NONHSAT0	chr1	1.63E+08	163343815	+
TC0100001963.oe.1	2.38	1.25	2.38	up	4.89	3.64	NONHSAG0	LNCipedia lc	lnc-LRRC52-	NONHSAT0	chr1	1.65E+08	164829952	+
TC0100002137.oe.1	-2.59	-1.37	2.59	down	3.07	4.44	lnc-LHX4-3	LNCipedia lc	lnc-LHX4-3:	---	chr1	1.8E+08	180384451	+
TC0100002344.oe.1	2.20	1.14	2.20	up	11.20	10.06	lnc-RNPEP-	NONCODE	lnc-RNPEP-	NONHSAT0	chr1	2.02E+08	201970351	+
TC0100002396.oe.1	-2.49	-1.31	2.49	down	4.98	6.29	lnc-MDM4-	LNCipedia lc	lnc-MDM4-	NONHSAT0	chr1	2.04E+08	204404742	+
TC0100002534.oe.1	3.24	1.70	3.24	up	10.62	8.93	lnc-TATDN3	LNCipedia lc	lnc-TATDN3	NONHSAT0	chr1	2.13E+08	212885565	+
TC0100002545.oe.1	2.81	1.49	2.81	up	5.57	4.08	lnc-CENPF-	NONCODE	lnc-CENPF-	NONHSAT0	chr1	2.15E+08	214615190	+
TC0100002674.oe.1	2.19	1.13	2.19	up	3.89	2.76	OTTHUMG0	LNCipedia lc	lnc-ADCK3-	NONHSAT0	chr1	2.27E+08	227183444	+
TC0100002686.oe.1	2.63	1.39	2.63	up	5.67	4.28	lnc-ARF1-1	NONCODE	lnc-ARF1-1:	NONHSAT0	chr1	2.28E+08	228104946	+
TC0100002863.oe.1	2.12	1.08	2.12	up	5.70	4.62	lnc-FAM36A	LNCipedia lc	lnc-FAM36A	---	chr1	2.45E+08	244865272	+
TC0100002878.oe.1	-2.57	-1.36	2.57	down	7.89	9.25	lnc-SCCPDH	LNCipedia lc	lnc-SCCPDH	NONHSAT0	chr1	2.47E+08	246668356	+
TC0100003415.oe.1	-2.53	-1.34	2.53	down	14.46	15.80	lnc-PTP4A2-	NONCODE	lnc-PTP4A2-	NONHSAT0	chr1	31906421	31908111	-
TC0100003509.oe.1	2.08	1.06	2.08	up	9.36	8.30	lnc-SNIP1-1	LNCipedia lc	lnc-SNIP1-1	NONHSAT0	chr1	37576170	37582809	-
TC0100003545.oe.1	2.28	1.19	2.28	up	4.56	3.37	lnc-PPT1-2	LNCipedia lc	lnc-PPT1-2:	---	chr1	40029014	40030573	-
TC0100003723.oe.1	-2.22	-1.15	2.22	down	3.08	4.23	lnc-RAB3B-	LNCipedia lc	lnc-RAB3B-	NONHSAT0	chr1	51856526	51860389	-
TC0100003755.oe.1	2.96	1.56	2.96	up	14.36	12.79	lnc-YIPF1-1	NONCODE	lnc-YIPF1-1:	NONHSAT0	chr1	53818970	53838463	-

TC0100003797.oe.1	-2.90	-1.54	2.90	down	3.78	5.31	lnc-C8B-1	NONCODE	lnc-C8B-1:2	NONHSAT0	chr1	56956855	56966140	-
TC0100003810.oe.1	2.90	1.53	2.90	up	5.22	3.68	lnc-TACSTD	LNCipedia l	lnc-TACSTD	NONHSAT0	chr1	58654740	58669291	-
TC0100003945.oe.1	4.28	2.10	4.28	up	10.90	8.80	lnc-FUBP1-2	NONCODE	lnc-FUBP1-2	NONHSAT0	chr1	77944055	77960785	-
TC0100003984.oe.1	-2.71	-1.44	2.71	down	12.28	13.72	lnc-CTBS-1	NONCODE	lnc-CTBS-1:	NONHSAT0	chr1	84553121	84574480	-
TC0100003997.oe.1	-3.48	-1.80	3.48	down	5.11	6.91	lnc-C1orf52	LNCipedia l	lnc-C1orf52	NONHSAT0	chr1	85174312	85176880	-
TC0100004080.oe.1	2.04	1.03	2.04	up	10.40	9.37	lnc-GCLM-4	LNCipedia l	lnc-GCLM-4	NONHSAT0	chr1	93869780	93870997	-
TC0100004092.oe.1	19.84	4.31	19.84	up	10.20	5.89	lnc-ARHGAF	NONCODE	lnc-ARHGAF	NONHSAT0	chr1	94148988	94154829	-
TC0100004122.oe.1	-3.01	-1.59	3.01	down	5.06	6.65	lnc-LPPR5.1	LNCipedia l	lnc-LPPR5.1	NONHSAT0	chr1	97691207	97721654	-
TC0100004124.oe.1	-2.36	-1.24	2.36	down	5.41	6.64	lnc-LPPR5.1	LNCipedia l	lnc-LPPR5.1	NONHSAT0	chr1	97855030	97920997	-
TC0100004205.oe.1	2.22	1.15	2.22	up	7.01	5.86	lnc-GSTM3-	LNCipedia l	lnc-GSTM3-	NONHSAT0	chr1	1.1E+08	109751940	-
TC0100004243.oe.1	2.26	1.18	2.26	up	3.84	2.66	lnc-FAM212	LNCipedia l	lnc-FAM212	NONHSAT0	chr1	1.12E+08	111772569	-
TC0100004270.oe.1	-2.39	-1.26	2.39	down	6.28	7.54	lnc-RSBN1-1	LNCipedia l	lnc-RSBN1-1	NONHSAT0	chr1	1.14E+08	113764810	-
TC0100004282.oe.1	2.17	1.12	2.17	up	4.32	3.20	lnc-BCAS2-1	LNCipedia l	lnc-BCAS2-1	---	chr1	1.15E+08	114558681	-
TC0100004366.oe.1	2.08	1.06	2.08	up	13.69	12.64	lnc-PPIAL4G	LNCipedia l	lnc-PPIAL4G	NONHSAT0	chr1	1.44E+08	143971749	-
TC0100004378.oe.1	-2.29	-1.19	2.29	down	8.70	9.89	lnc-PDZK1-1	LNCipedia l	lnc-PDZK1-1	NONHSAT0	chr1	1.46E+08	145672848	-
TC0100004444.oe.1	-2.03	-1.02	2.03	down	9.53	10.55	lnc-C1orf13	LNCipedia l	lnc-C1orf13	NONHSAT0	chr1	1.51E+08	150575125	-
TC0100004449.oe.1	2.86	1.51	2.86	up	5.81	4.30	lnc-HORMA	LNCipedia l	lnc-HORMA	NONHSAT0	chr1	1.51E+08	150720888	-
TC0100004580.oe.1	2.40	1.26	2.40	up	7.63	6.36	lnc-RIT1-1	NONCODE	lnc-RIT1-1:1	NONHSAT0	chr1	1.56E+08	155917574	-
TC0100004592.oe.1	2.23	1.15	2.23	up	11.64	10.49	lnc-C1orf85	LNCipedia l	lnc-C1orf85	NONHSAT0	chr1	1.56E+08	156338206	-
TC0100004676.oe.1	-2.64	-1.40	2.64	down	13.30	14.69	lnc-APOA2-	NONCODE	lnc-APOA2-	NONHSAT0	chr1	1.61E+08	161223631	-
TC0100004822.oe.1	-2.20	-1.14	2.20	down	3.75	4.88	lnc-RFWD2-	LNCipedia l	lnc-RFWD2-	NONHSAT0	chr1	1.76E+08	175922179	-
TC0100004826.oe.1	-2.08	-1.06	2.08	down	7.73	8.79	lnc-ASTN1-	NONCODE	lnc-ASTN1-	NONHSAT0	chr1	1.77E+08	176616789	-
TC0100004859.oe.1	2.00	1.00	2.00	up	4.02	3.02	OTTHUMG0	NONCODE	lnc-TOR1AIF	NONHSAT0	chr1	1.8E+08	179836124	-
TC0100004916.oe.1	2.06	1.05	2.06	up	7.20	6.16	lnc-FAM129	LNCipedia l	lnc-FAM129	NONHSAT0	chr1	1.85E+08	185120869	-
TC0100004961.oe.1	-2.12	-1.08	2.12	down	4.10	5.18	lnc-GLRX2-1	LNCipedia l	lnc-GLRX2-1	---	chr1	1.93E+08	193091556	-
TC0100004976.oe.1	3.03	1.60	3.03	up	9.76	8.16	lnc-ZBTB41-	LNCipedia l	lnc-ZBTB41-	NONHSAT0	chr1	1.97E+08	197146535	-
TC0100005156.oe.1	2.37	1.25	2.37	up	10.68	9.43	lnc-LPGAT1	LNCipedia l	lnc-LPGAT1	NONHSAT0	chr1	2.12E+08	211674363	-
TC0100005450.oe.1	-2.84	-1.50	2.84	down	6.21	7.72	lnc-GNG4-1	LNCipedia l	lnc-GNG4-1	NONHSAT0	chr1	2.36E+08	235549698	-
TC01000521.hg.4	2.81	1.49	2.81	up	5.35	3.86	SLFNL1-AS1	SLFNL1 antis	---	---	chr1	41014590	41043890	+
TC01000721.hg.4	2.27	1.18	2.27	up	5.44	4.26	DLEU2L	deleted in ly	---	---	chr1	63548964	63550636	+
TC01002278.hg.4	-2.46	-1.30	2.46	down	3.84	5.14	MST1//MST1	macrophage	---	---	chr1	16754906	16764480	-
TC01002632.hg.4	2.23	1.16	2.23	up	5.76	4.60	FOXD2-AS1	FOXD2 antis	---	---	chr1	47432133	47434641	-
TC01003327.hg.4	2.22	1.15	2.22	up	4.70	3.55	SCARNA4	small Cajal b	---	---	chr1	1.56E+08	155926086	-
TC01003969.hg.4	-2.34	-1.23	2.34	down	3.56	4.79	SNRPD2P2	small nuclea	---	---	chr1	2.31E+08	231476525	-
TC01006390.hg.4	2.63	1.39	2.63	up	9.64	8.25	HNRNPU-AS	non-protein	---	---	chr1	2.45E+08	244846941	-
TC01006415.hg.4	2.78	1.47	2.78	up	5.05	3.58	RP11-267N1	regulator of	---	---	chr1	1.63E+08	163321894	-
TC0200000115.oe.1	2.04	1.03	2.04	up	5.70	4.67	lnc-RRM2-1	LNCipedia l	lnc-RRM2-1	---	chr2	10120734	10121065	+
TC0200000226.oe.1	2.04	1.03	2.04	up	5.41	4.38	RP11-79O8.	LNCipedia l	lnc-RHOB-1	---	chr2	19902025	19902569	+
TC0200000245.oe.1	2.63	1.39	2.63	up	6.06	4.67	AC010872.1	N/A	---	---	chr2	21023496	21024170	+
TC0200000468.oe.1	2.19	1.13	2.19	up	6.18	5.04	lnc-MTA3-7	NONCODE	lnc-MTA3-7	NONHSAT0	chr2	42317474	42320341	+
TC0200000809.oe.1	-2.04	-1.03	2.04	down	3.41	4.44	lnc-ALMS1-	LNCipedia l	lnc-ALMS1-	NONHSAT0	chr2	73640949	73641628	+
TC0200000860.oe.1	-2.16	-1.11	2.16	down	6.35	7.46	NONHSAG0	LNCipedia l	lnc-POLE4-6	NONHSAT0	chr2	75669989	75670454	+
TC0200000971.oe.1	-2.23	-1.16	2.23	down	3.02	4.17	lnc-AC2332	LNCipedia l	lnc-AC2332	NONHSAT0	chr2	89862722	89862986	+
TC0200001002.oe.1	2.67	1.42	2.67	up	7.96	6.54	lnc-AC0734	gene_id XLC	lnc-AC0734	NONHSAT0	chr2	92085371	92106501	+
TC0200001004.oe.1	2.31	1.21	2.31	up	12.79	11.58	lnc-AC0734	NONCODE	lnc-AC0734	NONHSAT0	chr2	92117808	92128237	+
TC0200001005.oe.1	2.41	1.27	2.41	up	6.43	5.16	lnc-AC0734	LNCipedia l	lnc-AC0734	NONHSAT0	chr2	92130438	92136243	+
TC0200001085.oe.1	2.05	1.03	2.05	up	5.07	4.04	lnc-LIPT1-1	NONCODE	lnc-LIPT1-1:	NONHSAT0	chr2	99141950	99147490	+
TC0200001130.oe.1	-2.01	-1.01	2.01	down	4.76	5.77	lnc-TMEM18	NONCODE	lnc-TMEM18	NONHSAT0	chr2	1.03E+08	102686768	+
TC0200001131.oe.1	-2.29	-1.20	2.29	down	2.84	4.04	lnc-TMEM18	LNCipedia l	lnc-TMEM18	NONHSAT0	chr2	1.03E+08	102702451	+

TC0200001322.oe.1	-2.04	-1.03	2.04	down	8.80	9.83	lnc-INSIG2-4	NONCODE (	lnc-INSIG2-4	NONHSAT0	chr2	1.18E+08	118110997	+
TC0200001450.oe.1	-2.02	-1.01	2.02	down	3.53	4.54	lnc-PLEKHB2	LNCipedia lc	lnc-PLEKHB2	---	chr2	1.31E+08	131362128	+
TC0200001598.oe.1	2.05	1.03	2.05	up	3.98	2.95	lnc-LYPD6-8	LNCipedia lc	lnc-LYPD6-8	NONHSAT0	chr2	1.5E+08	149636840	+
TC0200001740.oe.1	-2.40	-1.26	2.40	down	5.51	6.77	lnc-PHOSPH	LNCipedia lc	lnc-PHOSPH	NONHSAT0	chr2	1.7E+08	169641403	+
TC0200001776.oe.1	2.22	1.15	2.22	up	7.97	6.82	lnc-METAP1	NONCODE (	lnc-METAP1	NONHSAT0	chr2	1.72E+08	171923475	+
TC0200001834.oe.1	2.04	1.03	2.04	up	5.53	4.50	lnc-HNRNPA	LNCipedia lc	lnc-HNRNPA	NONHSAT0	chr2	1.77E+08	177198547	+
TC0200001865.oe.1	2.35	1.23	2.35	up	5.29	4.06	lnc-PLEKHA3	LNCipedia lc	lnc-PLEKHA3	NONHSAT0	chr2	1.79E+08	178550681	+
TC0200002088.oe.1	-2.32	-1.22	2.32	down	3.78	5.00	lnc-NRP2-2	LNCipedia lc	lnc-NRP2-2:	NONHSAT0	chr2	2.06E+08	205618192	+
TC0200002150.oe.1	-2.58	-1.37	2.58	down	13.05	14.42	lnc-RPE-11	LNCipedia lc	lnc-RPE-11:	NONHSAT0	chr2	2.11E+08	210606771	+
TC0200002152.oe.1	-3.39	-1.76	3.39	down	15.93	17.70	lnc-RPE-13	NONCODE (	lnc-RPE-13:	NONHSAT0	chr2	2.11E+08	210663146	+
TC0200002307.oe.1	-2.39	-1.26	2.39	down	6.38	7.64	lnc-KCNE4-7	LNCipedia lc	lnc-KCNE4-7	NONHSAT0	chr2	2.23E+08	222944636	+
TC0200002339.oe.1	8.94	3.16	8.94	up	11.81	8.65	lnc-WDR69-	NONCODE (	lnc-WDR69-	NONHSAT0	chr2	2.28E+08	227817463	+
TC0200002479.oe.1	2.07	1.05	2.07	up	6.17	5.12	lnc-PRLH-1	LNCipedia lc	lnc-PRLH-1:	NONHSAT0	chr2	2.38E+08	237517509	+
TC0200002687.oe.1	-2.14	-1.10	2.14	down	3.16	4.26	lnc-MBOAT2	LNCipedia lc	lnc-MBOAT2	NONHSAT0	chr2	8852690	8854246	-
TC0200002711.oe.1	2.11	1.08	2.11	up	12.54	11.46	lnc-NOL10-	LNCipedia lc	lnc-NOL10-	NONHSAT0	chr2	10444095	10448309	-
TC0200002770.oe.1	-2.32	-1.21	2.32	down	3.11	4.32	lnc-RDH14-	LNCipedia lc	lnc-RDH14-	NONHSAT0	chr2	18423534	18425304	-
TC0200003220.oe.1	2.41	1.27	2.41	up	4.37	3.11	lnc-USP34-8	LNCipedia lc	lnc-USP34-8	---	chr2	60993208	60998496	-
TC0200003241.oe.1	2.56	1.35	2.56	up	10.83	9.48	lnc-FAM161	NONCODE (	lnc-FAM161	NONHSAT0	chr2	61887948	61888458	-
TC0200003555.oe.1	2.32	1.22	2.32	up	6.14	4.92	lnc-KRCC1-2	NONCODE (	lnc-KRCC1-2	NONHSAT0	chr2	88123029	88128059	-
TC0200003632.oe.1	2.27	1.18	2.27	up	7.96	6.77	lnc-GPAT2-5	LNCipedia lc	lnc-GPAT2-5	---	chr2	95891657	95896022	-
TC0200003671.oe.1	2.40	1.26	2.40	up	5.00	3.74	lnc-ACTR1B	NONCODE (	lnc-ACTR1B	NONHSAT0	chr2	97523949	97524976	-
TC0200003672.oe.1	3.91	1.97	3.91	up	7.07	5.10	lnc-ACTR1B	NONCODE (	lnc-ACTR1B	NONHSAT0	chr2	97548007	97550242	-
TC0200003708.oe.1	2.02	1.01	2.02	up	5.39	4.37	lnc-TBC1D8	LNCipedia lc	lnc-TBC1D8	NONHSAT0	chr2	1.01E+08	101294662	-
TC0200003727.oe.1	-2.21	-1.15	2.21	down	3.11	4.26	lnc-MFSD9-	LNCipedia lc	lnc-MFSD9-	---	chr2	1.03E+08	103486916	-
TC0200003810.oe.1	2.20	1.13	2.20	up	8.81	7.68	lnc-RGPD6-	LNCipedia lc	lnc-RGPD6-	NONHSAT0	chr2	1.11E+08	110650739	-
TC0200003811.oe.1	2.92	1.54	2.92	up	12.82	11.27	lnc-RGPD6-	LNCipedia lc	lnc-RGPD6-	NONHSAT0	chr2	1.11E+08	110667565	-
TC0200003860.oe.1	-3.25	-1.70	3.25	down	7.46	9.16	lnc-AC0167	NONCODE (	lnc-AC0167	NONHSAT0	chr2	1.14E+08	113710239	-
TC0200004166.oe.1	-2.00	-1.00	2.00	down	2.67	3.67	lnc-RPRM-6	LNCipedia lc	lnc-RPRM-6	NONHSAT0	chr2	1.54E+08	153762646	-
TC0200004364.oe.1	-2.12	-1.08	2.12	down	8.58	9.66	lnc-CHN1-5	NONCODE (	lnc-CHN1-5	NONHSAT0	chr2	1.75E+08	175074044	-
TC0200004365.oe.1	2.21	1.15	2.21	up	8.50	7.36	lnc-CHN1-6	LNCipedia lc	lnc-CHN1-6	NONHSAT0	chr2	1.75E+08	175093254	-
TC0200004398.oe.1	2.40	1.26	2.40	up	10.43	9.17	lnc-TTC30B-	LNCipedia lc	lnc-TTC30B-	NONHSAT0	chr2	1.77E+08	177229506	-
TC0200004462.oe.1	-3.58	-1.84	3.58	down	8.57	10.41	lnc-CALCRL	LNCipedia lc	lnc-CALCRL	NONHSAT0	chr2	1.87E+08	187466042	-
TC0200004490.oe.1	2.16	1.11	2.16	up	4.69	3.58	lnc-STAT1-1	LNCipedia lc	lnc-STAT1-1	---	chr2	1.91E+08	190935288	-
TC0200004662.oe.1	-2.26	-1.17	2.26	down	8.68	9.86	lnc-PECR-1	LNCipedia lc	lnc-PECR-1:	NONHSAT0	chr2	2.16E+08	216082955	-
TC0200004680.oe.1	-2.92	-1.55	2.92	down	4.98	6.52	lnc-DIRC3-4	NONCODE (	lnc-DIRC3-4	NONHSAT0	chr2	2.18E+08	217810304	-
TC0200004681.oe.1	-2.24	-1.16	2.24	down	4.97	6.13	lnc-DIRC3-5	LNCipedia lc	lnc-DIRC3-5	NONHSAT0	chr2	2.18E+08	217815368	-
TC0300000084.oe.1	2.70	1.43	2.70	up	4.86	3.43	RP11-1020A	LNCipedia lc	lnc-IL17RC-1	NONHSAT0	chr3	9935706	9936258	+
TC0300000086.oe.1	2.98	1.57	2.98	up	8.46	6.89	lnc-CRELD1	NONCODE (	lnc-CRELD1	NONHSAT0	chr3	9947404	9954787	+
TC0300000119.oe.1	2.25	1.17	2.25	up	5.15	3.98	lnc-TSEN2-3	LNCipedia lc	lnc-TSEN2-3	---	chr3	12558775	12560797	+
TC0300000192.oe.1	-2.82	-1.50	2.82	down	5.99	7.48	lnc-RAB5A-1	NONCODE (	lnc-RAB5A-1	NONHSAT0	chr3	20111594	20122795	+
TC0300000510.oe.1	-2.04	-1.03	2.04	down	3.03	4.05	lnc-RBM5-1	LNCipedia lc	lnc-RBM5-1	---	chr3	50086735	50086989	+
TC0300000517.oe.1	-3.50	-1.81	3.50	down	6.65	8.46	lnc-GNAI2-2	LNCipedia lc	lnc-GNAI2-2	NONHSAT0	chr3	50205246	50220979	+
TC0300000839.oe.1	-2.76	-1.46	2.76	down	4.81	6.27	lnc-GPR128	NONCODE (	lnc-GPR128	NONHSAT0	chr3	1.01E+08	100577065	+
TC0300000840.oe.1	2.60	1.38	2.60	up	4.48	3.10	lnc-TMEM45	LNCipedia lc	lnc-TMEM45	NONHSAT0	chr3	1.01E+08	100637575	+
TC0300000943.oe.1	-2.02	-1.01	2.02	down	3.34	4.35	lnc-BOC-2	LNCipedia lc	lnc-BOC-2:1	NONHSAT0	chr3	1.13E+08	113361319	+
TC0300001167.oe.1	-2.63	-1.39	2.63	down	5.13	6.53	lnc-NEK11-5	LNCipedia lc	lnc-NEK11-5	NONHSAT0	chr3	1.31E+08	131388824	+
TC0300001292.oe.1	4.67	2.22	4.67	up	10.68	8.46	lnc-AC1070	NONCODE (	lnc-AC1070	NONHSAT0	chr3	1.45E+08	145380795	+
TC0300001547.oe.1	2.31	1.21	2.31	up	11.19	9.98	lnc-FNDC3B	NONCODE (	lnc-FNDC3B	NONHSAT0	chr3	1.73E+08	172806107	+
TC0300001548.oe.1	2.73	1.45	2.73	up	9.33	7.88	lnc-FNDC3B	LNCipedia lc	lnc-FNDC3B	NONHSAT0	chr3	1.73E+08	172820311	+

TC0300001586.oe.1	-2.55	-1.35	2.55	down	4.98	6.33	lnc-ZNF639-	LNCipedia lc	lnc-ZNF639-	NONHSAT09	chr3	1.79E+08	179237501	+
TC0300001614.oe.1	2.26	1.18	2.26	up	7.44	6.27	lnc-TTC14-9	NONCODE c	lnc-TTC14-9	NONHSAT09	chr3	1.81E+08	180971816	+
TC0300001656.oe.1	2.03	1.02	2.03	up	13.80	12.78	lnc-ECE2-3	LNCipedia lc	lnc-ECE2-3:1	NONHSAT09	chr3	1.84E+08	184304011	+
TC0300001725.oe.1	-2.82	-1.50	2.82	down	7.30	8.80	lnc-LPP-2	NONCODE c	lnc-LPP-2:1	NONHSAT09	chr3	1.89E+08	188890671	+
TC0300001791.oe.1	2.08	1.06	2.08	up	6.33	5.27	lnc-MUC20-	LNCipedia lc	lnc-MUC20-	---	chr3	1.96E+08	195610974	+
TC0300001889.oe.1	4.59	2.20	4.59	up	6.71	4.52	NONHSAG0	NONCODE c	lnc-AC01881	NONHSAT08	chr3	5156905	5188298	-
TC0300002070.oe.1	-2.13	-1.09	2.13	down	4.12	5.21	lnc-AC10358	NONCODE c	lnc-AC10358	NONHSAT08	chr3	25764129	25784156	-
TC0300002078.oe.1	2.11	1.08	2.11	up	6.89	5.81	lnc-NEK10-2	LNCipedia lc	lnc-NEK10-2	NONHSAT08	chr3	27397865	27401044	-
TC0300002080.oe.1	2.27	1.18	2.27	up	8.17	6.99	lnc-NEK10-4	LNCipedia lc	lnc-NEK10-4	NONHSAT08	chr3	27421672	27424502	-
TC0300002219.oe.1	2.01	1.00	2.01	up	5.01	4.00	lnc-ZNF445-	LNCipedia lc	lnc-ZNF445-	---	chr3	44396137	44399039	-
TC0300002376.oe.1	2.28	1.19	2.28	up	10.24	9.05	lnc-ABHD14	NONCODE c	lnc-ABHD14	NONHSAT08	chr3	51994935	51995895	-
TC0300002559.oe.1	-2.45	-1.29	2.45	down	11.98	13.27	lnc-ZNF717-	LNCipedia lc	lnc-ZNF717-	NONHSAT09	chr3	78600113	78607289	-
TC0300002569.oe.1	-2.93	-1.55	2.93	down	10.41	11.95	lnc-ROBO1-	LNCipedia lc	lnc-ROBO1-	NONHSAT09	chr3	81654579	81670982	-
TC0300002663.oe.1	2.51	1.33	2.51	up	9.06	7.73	lnc-KIAA152	LNCipedia lc	lnc-KIAA152	NONHSAT09	chr3	1.09E+08	108589644	-
TC0300002763.oe.1	2.23	1.16	2.23	up	6.77	5.61	lnc-HCLS1-1	LNCipedia lc	lnc-HCLS1-1	NONHSAT09	chr3	1.22E+08	121533210	-
TC0300002790.oe.1	3.99	2.00	3.99	up	10.95	8.95	lnc-ITGB5-2	LNCipedia lc	lnc-ITGB5-2	NONHSAT09	chr3	1.25E+08	124927771	-
TC0300002892.oe.1	2.46	1.30	2.46	up	9.14	7.84	lnc-RAB6B-2	NONCODE c	lnc-RAB6B-2	NONHSAT09	chr3	1.34E+08	133617526	-
TC0300002930.oe.1	2.52	1.33	2.52	up	4.61	3.28	RP11-461M2	LNCipedia lc	lnc-DZIP1L-	---	chr3	1.38E+08	138005122	-
TC0300002981.oe.1	-2.03	-1.02	2.03	down	8.60	9.62	lnc-PAQR9-	NONCODE c	lnc-PAQR9-	NONHSAT09	chr3	1.43E+08	142829855	-
TC0300002994.oe.1	-3.11	-1.64	3.11	down	12.29	13.93	lnc-PLSCR4-	NONCODE c	lnc-PLSCR4-	NONHSAT09	chr3	1.46E+08	146071170	-
TC0300002995.oe.1	-2.61	-1.38	2.61	down	9.50	10.89	lnc-PLSCR4-	LNCipedia lc	lnc-PLSCR4-	NONHSAT09	chr3	1.46E+08	146073657	-
TC0300003172.oe.1	2.05	1.04	2.05	up	6.13	5.10	lnc-ZBBX-6	NONCODE c	lnc-ZBBX-6:	NONHSAT09	chr3	1.67E+08	167183380	-
TC0300003194.oe.1	-2.16	-1.11	2.16	down	8.61	9.72	lnc-LRRC31-	NONCODE c	lnc-LRRC31-	NONHSAT09	chr3	1.7E+08	170088850	-
TC0300003203.oe.1	2.63	1.40	2.63	up	12.72	11.32	lnc-RPL22L1	LNCipedia lc	lnc-RPL22L1	NONHSAT09	chr3	1.71E+08	170908644	-
TC0300003206.oe.1	-2.33	-1.22	2.33	down	13.33	14.55	lnc-TNIK-3	LNCipedia lc	lnc-TNIK-3:1	NONHSAT09	chr3	1.71E+08	171007470	-
TC0300003226.oe.1	2.38	1.25	2.38	up	5.67	4.41	lnc-TNFSF10	LNCipedia lc	lnc-TNFSF10	NONHSAT09	chr3	1.73E+08	172696135	-
TC0300003263.oe.1	-2.64	-1.40	2.64	down	7.88	9.28	lnc-GNB4-1	LNCipedia lc	lnc-GNB4-1	NONHSAT09	chr3	1.79E+08	179398177	-
TC0300003380.oe.1	2.34	1.23	2.34	up	12.89	11.66	lnc-CLDN1-	NONCODE c	lnc-CLDN1-	NONHSAT09	chr3	1.9E+08	189964121	-
TC0300003381.oe.1	2.30	1.20	2.30	up	8.20	6.99	lnc-CLDN1-	LNCipedia lc	lnc-CLDN1-	NONHSAT09	chr3	1.9E+08	189983484	-
TC0300003386.oe.1	2.34	1.23	2.34	up	3.83	2.60	lnc-GMNC-1	gene_id XLC	lnc-GMNC-1	NONHSAT09	chr3	1.91E+08	190793594	-
TC03000082.hg.4	2.03	1.02	2.03	up	4.76	3.73	TPRXL	tetra-peptid	---	---	chr3	13937307	14082811	+
TC03000392.hg.4	-2.30	-1.20	2.30	down	2.96	4.17	C3orf49	chromosome	---	---	chr3	63819362	63848636	+
TC03000966.hg.4	-2.05	-1.03	2.05	down	2.64	3.68	SOX2-OT	SOX2 overla	---	---	chr3	1.81E+08	181790946	+
TC0400000003.oe.1	-2.04	-1.03	2.04	down	4.08	5.10	CH17-262A2	N/A	---	---	chr4	149738	150317	+
TC0400000257.oe.1	3.32	1.73	3.32	up	9.08	7.34	lnc-SOD3-1	LNCipedia lc	lnc-SOD3-1	NONHSAT09	chr4	24328764	24333261	+
TC0400000397.oe.1	-2.48	-1.31	2.48	down	7.97	9.28	lnc-DCAF4L1	LNCipedia lc	lnc-DCAF4L1	NONHSAT09	chr4	41959142	41960556	+
TC0400000442.oe.1	4.90	2.29	4.90	up	6.85	4.56	lnc-CWH43-	NONCODE c	lnc-CWH43-	NONHSAT09	chr4	49579833	49580189	+
TC0400000489.oe.1	2.05	1.03	2.05	up	4.91	3.88	lnc-EXOC1-3	LNCipedia lc	lnc-EXOC1-3	NONHSAT09	chr4	55962735	55963054	+
TC0400000539.oe.1	-2.27	-1.18	2.27	down	3.73	4.91	lnc-LPHN3-1	LNCipedia lc	lnc-LPHN3-1	NONHSAT09	chr4	64608905	64609424	+
TC0400000600.oe.1	2.18	1.12	2.18	up	4.74	3.62	lnc-AFM-2	NONCODE c	lnc-AFM-2:1	NONHSAT09	chr4	73453490	73455777	+
TC0400000606.oe.1	3.31	1.73	3.31	up	11.24	9.51	lnc-CXCL6-2	LNCipedia lc	lnc-CXCL6-2	NONHSAT09	chr4	73740569	73741986	+
TC0400000607.oe.1	5.25	2.39	5.25	up	12.53	10.13	lnc-CXCL6-3	LNCipedia lc	lnc-CXCL6-3	NONHSAT09	chr4	73743139	73743351	+
TC0400000616.oe.1	-2.11	-1.07	2.11	down	9.94	11.02	lnc-EPGN-3	NONCODE c	lnc-EPGN-3	NONHSAT09	chr4	74365173	74382013	+
TC0400000617.oe.1	-2.29	-1.19	2.29	down	11.36	12.55	lnc-AREG-1	NONCODE c	lnc-AREG-1:	NONHSAT09	chr4	74382423	74384952	+
TC0400000618.oe.1	-2.53	-1.34	2.53	down	10.51	11.85	lnc-EREG-1	NONCODE c	lnc-EREG-1:	NONHSAT09	chr4	74448639	74450532	+
TC0400000688.oe.1	-2.64	-1.40	2.64	down	8.82	10.22	lnc-COPS4-1	LNCipedia lc	lnc-COPS4-1	NONHSAT09	chr4	83088777	83089241	+
TC0400000737.oe.1	2.02	1.01	2.02	up	5.40	4.38	lnc-MMRN1	LNCipedia lc	lnc-MMRN1	NONHSAT09	chr4	90312960	90468622	+
TC0400000842.oe.1	-2.40	-1.27	2.40	down	3.86	5.13	lnc-CCDC10	LNCipedia lc	lnc-CCDC10	---	chr4	1.1E+08	109692703	+
TC0400001288.oe.1	-2.24	-1.16	2.24	down	6.13	7.29	lnc-PALLD-8	NONCODE c	lnc-PALLD-8	NONHSAT09	chr4	1.7E+08	169723183	+

TC0400001679.oe.1	-2.15	-1.11	2.15	down	8.54	9.64	lnc-FGFBP2-	LNCipedia lc	lnc-FGFBP2-	NONHSAT0	chr4	15985511	15986037	-
TC0400001815.oe.1	-2.32	-1.21	2.32	down	8.41	9.62	lnc-APBB2-4	LNCipedia lc	lnc-APBB2-4	NONHSAT0	chr4	40432703	40466645	-
TC0400001834.oe.1	-2.67	-1.42	2.67	down	4.46	5.87	lnc-BEND4-4	LNCipedia lc	lnc-BEND4-4	NONHSAT0	chr4	42412975	42415147	-
TC0400001865.oe.1	-2.57	-1.36	2.57	down	2.67	4.03	lnc-OCIAD2	NONCODE (	lnc-OCIAD2	NONHSAT0	chr4	48852008	48860203	-
TC0400001868.oe.1	2.03	1.02	2.03	up	4.66	3.63	lnc-OCIAD2	LNCipedia lc	lnc-OCIAD2	NONHSAT0	chr4	49212251	49212624	-
TC0400002012.oe.1	2.14	1.10	2.14	up	7.85	6.76	lnc-CXCL5-1	LNCipedia lc	lnc-CXCL5-1	NONHSAT0	chr4	73995642	73996738	-
TC0400002101.oe.1	-2.07	-1.05	2.07	down	2.51	3.56	lnc-PLAC8-1	LNCipedia lc	lnc-PLAC8-1	---	chr4	83058642	83059578	-
TC0400002157.oe.1	-2.22	-1.15	2.22	down	5.25	6.40	lnc-NAP1L5-	NONCODE (	lnc-NAP1L5-	NONHSAT0	chr4	88732122	88737639	-
TC0400002166.oe.1	2.02	1.01	2.02	up	8.99	7.98	lnc-RP11-76	LNCipedia lc	lnc-RP11-76	NONHSAT0	chr4	90838501	90839112	-
TC0400002203.oe.1	-2.00	-1.00	2.00	down	6.54	7.55	NONHSAG0	LNCipedia lc	lnc-ADH1A-	NONHSAT0	chr4	99215795	99219246	-
TC0400002231.oe.1	2.30	1.20	2.30	up	9.35	8.15	lnc-UBE2D3	LNCipedia lc	lnc-UBE2D3	NONHSAT0	chr4	1.03E+08	102730171	-
TC0400002241.oe.1	2.26	1.18	2.26	up	9.50	8.32	lnc-BDH2-3	LNCipedia lc	lnc-BDH2-3	NONHSAT0	chr4	1.03E+08	103140407	-
TC0400002242.oe.1	2.09	1.07	2.09	up	8.46	7.40	lnc-BDH2-4	NONCODE (	lnc-BDH2-4	NONHSAT0	chr4	1.03E+08	103145781	-
TC0400002243.oe.1	2.06	1.04	2.06	up	8.04	7.00	lnc-BDH2-5	LNCipedia lc	lnc-BDH2-5	NONHSAT0	chr4	1.03E+08	103181963	-
TC0400002290.oe.1	-2.79	-1.48	2.79	down	5.75	7.23	lnc-CASP6-1	LNCipedia lc	lnc-CASP6-1	NONHSAT0	chr4	1.1E+08	109712045	-
TC0400002313.oe.1	2.46	1.30	2.46	up	6.99	5.69	lnc-NEUROG	NONCODE (	lnc-NEUROG	NONHSAT0	chr4	1.13E+08	112623977	-
TC0400002360.oe.1	2.14	1.10	2.14	up	8.52	7.42	lnc-PDE5A-4	LNCipedia lc	lnc-PDE5A-4	NONHSAT0	chr4	1.2E+08	120061036	-
TC0400002645.oe.1	-2.14	-1.10	2.14	down	3.24	4.34	lnc-TMEM19	LNCipedia lc	lnc-TMEM19	---	chr4	1.65E+08	165071004	-
TC0400002706.oe.1	-2.12	-1.08	2.12	down	3.03	4.11	NONHSAG0	NONCODE (	lnc-FBXO8-3	NONHSAT0	chr4	1.74E+08	174154637	-
TC0400002735.oe.1	2.19	1.13	2.19	up	9.09	7.96	lnc-AGA-4	gene_id XLC	lnc-AGA-4:1	NONHSAT0	chr4	1.79E+08	179170221	-
TC0400002844.oe.1	2.03	1.02	2.03	up	3.81	2.80	lnc-FRG2-13	LNCipedia lc	lnc-FRG2-13	---	chr4	1.9E+08	189704490	-
TC04000297.hg.4	2.04	1.03	2.04	up	9.84	8.81	DANCR	differentiatic	---	---	chr4	52712430	52720351	+
TC0500000029.oe.1	3.62	1.86	3.62	up	8.79	6.93	lnc-RP11-66	NONCODE (	lnc-RP11-66	NONHSAT0	chr5	915928	917900	+
TC0500000159.oe.1	2.90	1.53	2.90	up	7.70	6.17	lnc-MARCH(	LNCipedia lc	lnc-MARCH(	NONHSAT1(	chr5	10250319	10254584	+
TC0500000169.oe.1	-3.17	-1.66	3.17	down	6.85	8.52	lnc-MARCH(	LNCipedia lc	lnc-MARCH(	NONHSAT1(	chr5	10435770	10437849	+
TC0500000322.oe.1	-2.22	-1.15	2.22	down	2.54	3.69	NONHSAG0	LNCipedia lc	lnc-NPR3-2:	NONHSAT1(	chr5	33008628	33025724	+
TC0500000387.oe.1	-2.58	-1.36	2.58	down	5.50	6.87	lnc-C5orf51-	LNCipedia lc	lnc-C5orf51-	NONHSAT1(	chr5	41939298	41941570	+
TC0500000419.oe.1	-2.80	-1.48	2.80	down	9.62	11.10	lnc-AC1149	LNCipedia lc	lnc-AC1149	NONHSAT1(	chr5	43644745	43645665	+
TC0500000426.oe.1	-2.75	-1.46	2.75	down	7.56	9.02	lnc-MRPS30	LNCipedia lc	lnc-MRPS30	NONHSAT1(	chr5	44815219	44820428	+
TC0500000598.oe.1	2.55	1.35	2.55	up	13.33	11.98	lnc-CENPH-	LNCipedia lc	lnc-CENPH-	NONHSAT1(	chr5	69174926	69175546	+
TC0500000599.oe.1	2.22	1.15	2.22	up	11.43	10.28	lnc-CENPH-	LNCipedia lc	lnc-CENPH-	NONHSAT1(	chr5	69177061	69177697	+
TC0500000610.oe.1	-2.13	-1.09	2.13	down	5.72	6.82	lnc-GTF2H2(	NONCODE (	lnc-GTF2H2(	NONHSAT1(	chr5	69593473	69594723	+
TC0500000645.oe.1	2.03	1.02	2.03	up	9.05	8.03	lnc-FCHO2-	LNCipedia lc	lnc-FCHO2-	NONHSAT1(	chr5	72900874	72908913	+
TC0500000679.oe.1	2.35	1.24	2.35	up	15.91	14.68	lnc-POLK-5	LNCipedia lc	lnc-POLK-5:	NONHSAT1(	chr5	75345599	75350821	+
TC0500000680.oe.1	2.47	1.31	2.47	up	13.24	11.94	lnc-POLK-4	NONCODE (	lnc-POLK-4:	NONHSAT1(	chr5	75354308	75355166	+
TC0500000681.oe.1	2.44	1.29	2.44	up	13.21	11.93	lnc-POLK-3	LNCipedia lc	lnc-POLK-3:	NONHSAT1(	chr5	75355180	75356449	+
TC0500000856.oe.1	2.19	1.13	2.19	up	5.57	4.44	lnc-ANKRD3	LNCipedia lc	lnc-ANKRD3	NONHSAT1(	chr5	93594017	93594611	+
TC0500000888.oe.1	-2.05	-1.04	2.05	down	3.15	4.19	lnc-CAST-1(	LNCipedia lc	lnc-CAST-1(	NONHSAT1(	chr5	96211201	96212868	+
TC0500000898.oe.1	2.16	1.11	2.16	up	5.27	4.15	lnc-CAST-5	LNCipedia lc	lnc-CAST-5:	NONHSAT1(	chr5	96777650	96779982	+
TC0500000940.oe.1	-3.07	-1.62	3.07	down	8.72	10.34	lnc-PPIP5K2	NONCODE (	lnc-PPIP5K2	NONHSAT1(	chr5	1.03E+08	103029267	+
TC0500000946.oe.1	2.61	1.38	2.61	up	4.56	3.18	RP11-313L6	N/A	---	---	chr5	1.04E+08	104435499	+
TC0500000964.oe.1	-2.16	-1.11	2.16	down	4.95	6.06	lnc-MAN2A1	LNCipedia lc	lnc-MAN2A1	NONHSAT1(	chr5	1.09E+08	109195058	+
TC0500000982.oe.1	2.01	1.01	2.01	up	6.47	5.46	lnc-TSLP-2	LNCipedia lc	lnc-TSLP-2:1	NONHSAT1(	chr5	1.11E+08	111095209	+
TC0500001004.oe.1	2.15	1.10	2.15	up	8.19	7.08	lnc-ZRSR1-6	LNCipedia lc	lnc-ZRSR1-6	NONHSAT1(	chr5	1.13E+08	113008307	+
TC0500001020.oe.1	2.82	1.49	2.82	up	5.11	3.61	lnc-AP3S1-9	LNCipedia lc	lnc-AP3S1-9	NONHSAT1(	chr5	1.16E+08	115579333	+
TC0500001126.oe.1	2.19	1.13	2.19	up	12.47	11.34	lnc-SLC27A6	LNCipedia lc	lnc-SLC27A6	NONHSAT1(	chr5	1.28E+08	128182921	+
TC0500001242.oe.1	2.87	1.52	2.87	up	7.16	5.63	lnc-WNT8A-	NONCODE (	lnc-WNT8A-	NONHSAT1(	chr5	1.38E+08	138181896	+
TC0500001359.oe.1	2.16	1.11	2.16	up	13.57	12.46	lnc-POU4F3	LNCipedia lc	lnc-POU4F3	NONHSAT1(	chr5	1.46E+08	146263519	+
TC0500001502.oe.1	2.40	1.27	2.40	up	9.13	7.86	lnc-FABP6-4	LNCipedia lc	lnc-FABP6-4	NONHSAT1(	chr5	1.6E+08	160424777	+

TC0500001525.oe.1	4.21	2.08	4.21	up	9.85	7.77	lnc-CCNG1-	NONCODE (	lnc-CCNG1-	NONHSAT1(	chr5	1.63E+08	163469910	+
TC0500001562.oe.1	2.38	1.25	2.38	up	4.49	3.23	lnc-GABRP-	LNCipedia lc	lnc-GABRP-	---	chr5	1.71E+08	170923847	+
TC0500001778.oe.1	2.51	1.33	2.51	up	6.74	5.41	lnc-ZDHHC1	LNCipedia lc	lnc-ZDHHC1	NONHSAT0(	chr5	767397	768931	-
TC0500001831.oe.1	-2.15	-1.10	2.15	down	3.65	4.75	lnc-MED10-	NONCODE (	lnc-MED10-	NONHSAT1(	chr5	5420759	5421806	-
TC0500001902.oe.1	4.29	2.10	4.29	up	5.45	3.35	CTD-2199O	LNCipedia lc	lnc-FAM173	---	chr5	10203600	10204040	-
TC0500002066.oe.1	2.18	1.12	2.18	up	6.46	5.34	RP11-1250I1	N/A	---	---	chr5	34124162	34124385	-
TC0500002082.oe.1	-2.07	-1.05	2.07	down	7.31	8.37	lnc-UGT3A2	LNCipedia lc	lnc-UGT3A2	NONHSAT1(	chr5	36098556	36102381	-
TC0500002115.oe.1	2.00	1.00	2.00	up	5.15	4.15	lnc-AC0914	NONCODE (	lnc-AC0914	NONHSAT1(	chr5	38528672	38530917	-
TC0500002128.oe.1	2.51	1.33	2.51	up	5.27	3.94	lnc-FYB-2	LNCipedia lc	lnc-FYB-2:1	NONHSAT1(	chr5	39374745	39376042	-
TC0500002164.oe.1	2.03	1.02	2.03	up	12.68	11.65	lnc-CCL28-2	LNCipedia lc	lnc-CCL28-2	NONHSAT1(	chr5	43293734	43295857	-
TC0500002168.oe.1	3.05	1.61	3.05	up	7.52	5.91	NONHSAG0	NONCODE (	lnc-C5orf28	NONHSAT1(	chr5	43490329	43494605	-
TC0500002178.oe.1	2.17	1.12	2.17	up	6.64	5.52	lnc-EMB-6	LNCipedia lc	lnc-EMB-6:1	NONHSAT1(	chr5	50403256	50443248	-
TC0500002201.oe.1	3.62	1.85	3.62	up	6.60	4.74	lnc-MOCS2-	LNCipedia lc	lnc-MOCS2-	---	chr5	54011393	54011596	-
TC0500002252.oe.1	2.29	1.19	2.29	up	6.26	5.07	lnc-PDE4D-	NONCODE (	lnc-PDE4D-	NONHSAT1(	chr5	60605684	60628691	-
TC0500002412.oe.1	-2.02	-1.02	2.02	down	8.75	9.76	lnc-WDR41-	LNCipedia lc	lnc-WDR41-	NONHSAT1(	chr5	77073640	77076544	-
TC0500002460.oe.1	2.10	1.07	2.10	up	11.06	9.99	lnc-ANKRD3	NONCODE (	lnc-ANKRD3	NONHSAT1(	chr5	80628538	80654547	-
TC0500002506.oe.1	2.33	1.22	2.33	up	7.17	5.94	NONHSAG0	NONCODE (	lnc-AC0083	NONHSAT1(	chr5	86380660	86381426	-
TC0500002801.oe.1	2.39	1.26	2.39	up	5.76	4.50	lnc-C5orf63	LNCipedia lc	lnc-C5orf63	NONHSAT1(	chr5	1.27E+08	126914990	-
TC0500002872.oe.1	-2.49	-1.32	2.49	down	5.56	6.87	lnc-SAR1B-1	LNCipedia lc	lnc-SAR1B-1	NONHSAT1(	chr5	1.35E+08	134727819	-
TC0500002915.oe.1	2.00	1.00	2.00	up	7.56	6.55	lnc-GFRA3-1	NONCODE l	lnc-GFRA3-1	NONHSAT1(	chr5	1.38E+08	138331087	-
TC0500003066.oe.1	-2.68	-1.42	2.68	down	5.30	6.72	lnc-CSF1R-1	NONCODE (	lnc-CSF1R-1	NONHSAT1(	chr5	1.5E+08	150114958	-
TC0500003068.oe.1	-2.28	-1.19	2.28	down	4.87	6.05	lnc-CSF1R-3	NONCODE (	lnc-CSF1R-3	NONHSAT1(	chr5	1.5E+08	150134110	-
TC0500003211.oe.1	-2.22	-1.15	2.22	down	9.08	10.23	lnc-PANK3-	LNCipedia lc	lnc-PANK3-	NONHSAT1(	chr5	1.69E+08	168553727	-
TC0500003267.oe.1	-2.41	-1.27	2.41	down	9.44	10.71	lnc-NKX2-5	NONCODE (	lnc-NKX2-5	NONHSAT1(	chr5	1.73E+08	173328447	-
TC05002121.hg.4	-2.03	-1.02	2.03	down	2.39	3.41	GMCL1P1	germ cell-le	---	---	chr5	1.78E+08	178187432	-
TC0600000269.oe.1	2.52	1.33	2.52	up	7.44	6.10	HULC//NON	hepatocellul	lnc-BMP6-3	NONHSAT1(	chr6	8652137	8657427	+
TC0600000296.oe.1	-2.71	-1.44	2.71	down	6.10	7.54	lnc-C6orf22	LNCipedia lc	lnc-C6orf22	NONHSAT1(	chr6	11136920	11138738	+
TC0600000468.oe.1	2.58	1.37	2.58	up	9.03	7.66	lnc-PRSS16-	LNCipedia lc	lnc-PRSS16-	NONHSAT1(	chr6	27230398	27230655	+
TC0600000483.oe.1	-2.02	-1.01	2.02	down	3.62	4.63	lnc-HIST1H2	LNCipedia lc	lnc-HIST1H2	NONHSAT1(	chr6	27853720	27855804	+
TC0600000802.oe.1	-2.04	-1.03	2.04	down	9.27	10.30	lnc-RSPH9-	LNCipedia lc	lnc-RSPH9-	NONHSAT1	chr6	43777720	43786439	+
TC0600000888.oe.1	2.11	1.08	2.11	up	10.70	9.62	lnc-MLIP-2	NONCODE (	lnc-MLIP-2:	NONHSAT1	chr6	53919240	53920748	+
TC0600000953.oe.1	2.33	1.22	2.33	up	5.01	3.79	lnc-C6orf57	LNCipedia lc	lnc-C6orf57	NONHSAT1	chr6	70512115	70515953	+
TC0600001041.oe.1	2.96	1.56	2.96	up	13.87	12.31	lnc-BCKDHB	LNCipedia lc	lnc-BCKDHB	NONHSAT1	chr6	80005941	80011553	+
TC0600001042.oe.1	2.77	1.47	2.77	up	7.87	6.40	lnc-BCKDHB	NONCODE (	lnc-BCKDHB	NONHSAT1	chr6	80037553	80040271	+
TC0600001091.oe.1	2.02	1.02	2.02	up	8.65	7.63	lnc-SLC35A1	LNCipedia lc	lnc-SLC35A1	NONHSAT1	chr6	87650930	87653214	+
TC0600001184.oe.1	3.18	1.67	3.18	up	6.68	5.01	RP3-467N11	LNCipedia lc	lnc-GRIK2-2	NONHSAT1	chr6	1.01E+08	100882987	+
TC0600001244.oe.1	-2.41	-1.27	2.41	down	13.89	15.17	lnc-LACE1-7	LNCipedia lc	lnc-LACE1-7	---	chr6	1.09E+08	108681190	+
TC0600001254.oe.1	2.11	1.07	2.11	up	4.39	3.32	lnc-C6orf18	LNCipedia lc	lnc-C6orf18	---	chr6	1.09E+08	109272805	+
TC0600001378.oe.1	-2.20	-1.14	2.20	down	7.47	8.61	lnc-TPD52L1	LNCipedia lc	lnc-TPD52L1	NONHSAT1	chr6	1.25E+08	125086338	+
TC0600001386.oe.1	2.21	1.15	2.21	up	8.56	7.42	lnc-HINT3-1	LNCipedia lc	lnc-HINT3-1	NONHSAT1	chr6	1.26E+08	125998098	+
TC0600001396.oe.1	-2.08	-1.05	2.08	down	2.83	3.89	lnc-RNF146	LNCipedia lc	lnc-RNF146	NONHSAT1	chr6	1.27E+08	127155285	+
TC0600001422.oe.1	2.08	1.06	2.08	up	5.86	4.80	lnc-AKAP7-1	LNCipedia lc	lnc-AKAP7-1	NONHSAT1	chr6	1.31E+08	131309274	+
TC0600001450.oe.1	-2.13	-1.09	2.13	down	3.07	4.16	lnc-EYA4-2/	LNCipedia lc	lnc-EYA4-2:	---	chr6	1.34E+08	133541174	+
TC0600001488.oe.1	2.11	1.08	2.11	up	11.36	10.28	lnc-KIAA124	LNCipedia lc	lnc-KIAA124	NONHSAT1	chr6	1.38E+08	137875744	+
TC0600001525.oe.1	2.27	1.18	2.27	up	9.33	8.14	lnc-VTA1-4	LNCipedia lc	lnc-VTA1-4:	NONHSAT1	chr6	1.42E+08	142408220	+
TC0600001534.oe.1	-2.43	-1.28	2.43	down	6.41	7.69	lnc-PEX3-5	LNCipedia lc	lnc-PEX3-5:	NONHSAT1	chr6	1.43E+08	143187814	+
TC0600001550.oe.1	-2.99	-1.58	2.99	down	6.65	8.23	lnc-UTRN-1	gene_id XLC	lnc-UTRN-1	NONHSAT1	chr6	1.44E+08	144288184	+
TC0600001558.oe.1	-2.74	-1.45	2.74	down	7.76	9.21	lnc-STX11-9	LNCipedia lc	lnc-STX11-9	NONHSAT1	chr6	1.45E+08	144846822	+
TC0600001750.oe.1	3.49	1.80	3.49	up	10.08	8.27	CCR6//lnc-C	C-C chemok	lnc-CCR6-3:	NONHSAT1	chr6	1.67E+08	167139141	+

TC0600001869.oe.1	2.15	1.10	2.15	up	4.04	2.94	lnc-C6orf19!	LNCipedia lc	lnc-C6orf19!	NONHSAT1(	chr6	1726935	1728872	-
TC0600001872.oe.1	2.14	1.10	2.14	up	4.93	3.83	lnc-C6orf19!	LNCipedia lc	lnc-C6orf19!	NONHSAT1(	chr6	1932246	1934181	-
TC0600001874.oe.1	2.14	1.10	2.14	up	4.23	3.13	lnc-C6orf19!	LNCipedia lc	lnc-C6orf19!	NONHSAT1(	chr6	1949255	1949744	-
TC0600001882.oe.1	2.53	1.34	2.53	up	4.77	3.43	lnc-C6orf19!	LNCipedia lc	lnc-C6orf19!	NONHSAT1(	chr6	2037478	2037967	-
TC0600002032.oe.1	2.04	1.03	2.04	up	4.00	2.97	lnc-SSR1-1	LNCipedia lc	lnc-SSR1-1::	NONHSAT1(	chr6	7373953	7389402	-
TC0600002047.oe.1	-2.04	-1.03	2.04	down	4.03	5.06	lnc-TFAP2A-	NONCODE l	lnc-TFAP2A-	NONHSAT1(	chr6	9841932	9864115	-
TC0600002088.oe.1	2.27	1.18	2.27	up	6.15	4.96	lnc-RANBP9	LNCipedia lc	lnc-RANBP9	NONHSAT1(	chr6	13572073	13574214	-
TC0600002200.oe.1	2.34	1.23	2.34	up	6.18	4.95	lnc-HIST1H3	LNCipedia lc	lnc-HIST1H3	---	chr6	26194270	26196134	-
TC0600002213.oe.1	2.01	1.01	2.01	up	6.01	5.00	RP11-239L2	LNCipedia lc	lnc-HIST1H2	---	chr6	27001208	27001648	-
TC0600002229.oe.1	-2.35	-1.24	2.35	down	3.51	4.74	lnc-HIST1H2	LNCipedia lc	lnc-HIST1H2	NONHSAT1(	chr6	27666983	27668148	-
TC0600002375.oe.1	-2.23	-1.16	2.23	down	5.71	6.86	lnc-HLA-DC	LNCipedia lc	lnc-HLA-DC	NONHSAT1(	chr6	32825415	32828345	-
TC0600002515.oe.1	-2.22	-1.15	2.22	down	9.10	10.26	lnc-MRPS18	NONCODE (	lnc-MRPS18	NONHSAT1:	chr6	43624614	43625864	-
TC0600002604.oe.1	2.92	1.55	2.92	up	9.46	7.91	lnc-DST-3	LNCipedia lc	lnc-DST-3:1	NONHSAT1:	chr6	56276529	56276796	-
TC0600002605.oe.1	-2.12	-1.09	2.12	down	6.85	7.94	RP3-445N2.	LNCipedia lc	lnc-DST-1:1	NONHSAT1:	chr6	56331788	56332117	-
TC0600002627.oe.1	2.16	1.11	2.16	up	3.73	2.62	lnc-RAB23-1	LNCipedia lc	lnc-RAB23-1	NONHSAT1:	chr6	58071720	58073134	-
TC0600002637.oe.1	2.44	1.29	2.44	up	9.16	7.87	lnc-LGSN-3	LNCipedia lc	lnc-LGSN-3:	---	chr6	63436392	63440372	-
TC0600002673.oe.1	2.22	1.15	2.22	up	5.78	4.63	lnc-MB21D1	LNCipedia lc	lnc-MB21D1	NONHSAT1:	chr6	73391038	73391767	-
TC0600002853.oe.1	2.62	1.39	2.62	up	4.41	3.02	lnc-BVES-1	LNCipedia lc	lnc-BVES-1::	---	chr6	1.05E+08	105038269	-
TC0600002878.oe.1	2.60	1.38	2.60	up	6.75	5.37	lnc-OSTM1-	LNCipedia lc	lnc-OSTM1-	NONHSAT1:	chr6	1.08E+08	107958042	-
TC0600002993.oe.1	-3.06	-1.61	3.06	down	6.64	8.25	RP3-425C14	LNCipedia lc	lnc-SERINC1	NONHSAT1:	chr6	1.22E+08	122439223	-
TC0600003161.oe.1	-2.22	-1.15	2.22	down	5.47	6.62	lnc-SF3B5-5	LNCipedia lc	lnc-SF3B5-5	NONHSAT1:	chr6	1.45E+08	144708497	-
TC0600003166.oe.1	2.56	1.36	2.56	up	5.15	3.80	lnc-FBXO30-	LNCipedia lc	lnc-FBXO30-	NONHSAT1:	chr6	1.46E+08	145897422	-
TC0600003289.oe.1	3.99	2.00	3.99	up	16.94	14.94	lnc-TCP1-5	LNCipedia lc	lnc-TCP1-5::	NONHSAT1:	chr6	1.6E+08	159692650	-
TC0600003306.oe.1	-2.21	-1.14	2.21	down	2.82	3.96	lnc-LPA-2	LNCipedia lc	lnc-LPA-2:1	NONHSAT1:	chr6	1.61E+08	160817351	-
TC0600003349.oe.1	2.06	1.04	2.06	up	7.06	6.02	lnc-PRR18-2	LNCipedia lc	lnc-PRR18-2	NONHSAT1:	chr6	1.66E+08	166240493	-
TC0600003425.oe.1	-2.16	-1.11	2.16	down	5.79	6.90	lnc-PSMB1-	LNCipedia lc	lnc-PSMB1-	NONHSAT1:	chr6	1.71E+08	170576937	-
TC06001436.hg.4	-2.27	-1.18	2.27	down	4.89	6.07	TOB2P1	transducer o	---	---	chr6	28215338	28218929	-
TC0700000159.oe.1	6.74	2.75	6.74	up	7.02	4.27	lnc-ARL4A-6	LNCipedia lc	lnc-ARL4A-6	NONHSAT1:	chr7	12966846	12967172	+
TC0700000160.oe.1	3.10	1.63	3.10	up	7.53	5.90	lnc-ARL4A-7	LNCipedia lc	lnc-ARL4A-7	---	chr7	13067916	13670674	+
TC0700000164.oe.1	3.18	1.67	3.18	up	7.75	6.08	lnc-ARL4A-9	NONCODE (	lnc-ARL4A-9	NONHSAT1:	chr7	14003297	14009679	+
TC0700000251.oe.1	2.36	1.24	2.36	up	5.83	4.59	lnc-CCDC12	LNCipedia lc	lnc-CCDC12	NONHSAT1:	chr7	23474528	23475589	+
TC0700000261.oe.1	2.04	1.03	2.04	up	7.16	6.13	lnc-NPY-3	LNCipedia lc	lnc-NPY-3:1	---	chr7	24660137	24660535	+
TC0700000283.oe.1	2.25	1.17	2.25	up	6.17	5.00	NONHSAG0	LNCipedia lc	lnc-EVX1-13	NONHSAT1:	chr7	27095647	27100265	+
TC0700000395.oe.1	2.20	1.14	2.20	up	14.81	13.67	lnc-EEPD1-3	NONCODE (	lnc-EEPD1-3	NONHSAT1:	chr7	36403493	36417148	+
TC0700000428.oe.1	2.19	1.13	2.19	up	4.79	3.65	lnc-CDK13-(	LNCipedia lc	lnc-CDK13-(	NONHSAT1:	chr7	39816549	39868140	+
TC0700000604.oe.1	2.17	1.12	2.17	up	10.02	8.91	lnc-ZNF716-	LNCipedia lc	lnc-ZNF716-	NONHSAT1:	chr7	57490211	57496130	+
TC0700000649.oe.1	2.23	1.16	2.23	up	5.32	4.16	lnc-ZNF138-	LNCipedia lc	lnc-ZNF138-	NONHSAT1:	chr7	64681133	64686885	+
TC0700000661.oe.1	2.16	1.11	2.16	up	5.17	4.06	lnc-ZNF273-	NONCODE (	lnc-ZNF273-	NONHSAT1:	chr7	65373838	65398699	+
TC0700000782.oe.1	-2.01	-1.01	2.01	down	4.99	6.00	lnc-MDH2-2	LNCipedia lc	lnc-MDH2-2	NONHSAT1:	chr7	76099440	76102834	+
TC0700000812.oe.1	2.58	1.37	2.58	up	6.59	5.22	lnc-RSBN1L-	LNCipedia lc	lnc-RSBN1L-	---	chr7	77618632	77618950	+
TC0700000833.oe.1	-2.88	-1.52	2.88	down	4.44	5.96	lnc-CD36-3	LNCipedia lc	lnc-CD36-3:	NONHSAT1:	chr7	80218678	80219288	+
TC0700000928.oe.1	2.18	1.12	2.18	up	6.07	4.94	lnc-ASB4-2	gene_id XLC	lnc-ASB4-2:	NONHSAT1:	chr7	95440011	95442401	+
TC0700001287.oe.1	2.57	1.36	2.57	up	4.26	2.90	lnc-CPA1-3	NONCODE (	lnc-CPA1-3:	NONHSAT1:	chr7	1.3E+08	130501069	+
TC0700001309.oe.1	2.12	1.08	2.12	up	6.96	5.87	lnc-AC0093(	LNCipedia lc	lnc-AC0093(	NONHSAT1:	chr7	1.33E+08	133254018	+
TC0700001320.oe.1	5.13	2.36	5.13	up	6.07	3.71	lnc-AKR1B1(	NONCODE (	lnc-AKR1B1(	NONHSAT1:	chr7	1.35E+08	134541408	+
TC0700001375.oe.1	-4.81	-2.27	4.81	down	5.45	7.72	lnc-TTC26-3	LNCipedia lc	lnc-TTC26-3	---	chr7	1.39E+08	139024456	+
TC0700001506.oe.1	5.18	2.37	5.18	up	5.81	3.43	OTTHUMG0(	LNCipedia lc	lnc-LRRC61-	NONHSAT1:	chr7	1.5E+08	150342607	+
TC0700001614.oe.1	2.06	1.04	2.06	up	6.70	5.65	lnc-DNAJB6	LNCipedia lc	lnc-DNAJB6	---	chr7	1.57E+08	157196338	+
TC0700001764.oe.1	2.62	1.39	2.62	up	5.37	3.99	lnc-RPA3-7	LNCipedia lc	lnc-RPA3-7:	NONHSAT1:	chr7	8218441	8218951	-

TC0700001846.oe.1	2.17	1.11	2.17	up	4.93	3.82	lnc-STEAP1E	NONCODE (	lnc-STEAP1E	NONHSAT1:	chr7	22214433	22215408	-
TC0700001904.oe.1	2.51	1.33	2.51	up	5.60	4.27	CTB-119C2.:	NONCODE (	lnc-HNRNPA	NONHSAT1:	chr7	26173533	26185341	-
TC0700002006.oe.1	-2.12	-1.08	2.12	down	2.85	3.93	lnc-ELMO1-	LNCipedia lo	lnc-ELMO1-	NONHSAT12	chr7	36623190	36661300	-
TC0700002072.oe.1	2.23	1.15	2.23	up	5.63	4.47	lnc-MYL7-1	NONCODE (	lnc-MYL7-1:	NONHSAT12	chr7	44122088	44123496	-
TC0700002082.oe.1	2.47	1.30	2.47	up	9.51	8.21	lnc-TMED4-	NONCODE (	lnc-TMED4-	NONHSAT12	chr7	44569752	44570765	-
TC0700002096.oe.1	-3.15	-1.65	3.15	down	15.95	17.60	lnc-AC0112	LNCipedia lo	lnc-AC0112	NONHSAT12	chr7	45913773	45915325	-
TC0700002167.oe.1	2.24	1.16	2.24	up	7.26	6.10	lnc-RP4-725	NONCODE (	lnc-RP4-725	NONHSAT14	chr7	56421857	56448375	-
TC0700002400.oe.1	-2.33	-1.22	2.33	down	5.46	6.68	lnc-C7orf23-	NONCODE (	lnc-C7orf23-	NONHSAT12	chr7	86891195	86892935	-
TC0700002443.oe.1	2.04	1.03	2.04	up	3.62	2.59	lnc-SAMD9L	NONCODE (	lnc-SAMD9L	NONHSAT12	chr7	93145606	93148369	-
TC0700002508.oe.1	3.11	1.64	3.11	up	11.00	9.36	lnc-GATS-1	LNCipedia lo	lnc-GATS-1:	---	chr7	1E+08	100310676	-
TC0700002786.oe.1	-2.00	-1.00	2.00	down	2.47	3.47	lnc-SLC35B4	LNCipedia lo	lnc-SLC35B4	NONHSAT12	chr7	1.34E+08	133733595	-
TC0700002808.oe.1	2.79	1.48	2.79	up	10.49	9.01	lnc-PTN-9	LNCipedia lo	lnc-PTN-9:1	NONHSAT12	chr7	1.37E+08	136938553	-
TC0700002812.oe.1	-2.35	-1.23	2.35	down	2.98	4.22	lnc-PTN-4	LNCipedia lo	lnc-PTN-4:1	NONHSAT12	chr7	1.37E+08	137204840	-
TC07000084.hg.4	2.08	1.05	2.08	up	3.08	2.02	PER4	period circac	---	---	chr7	9634270	9635817	+
TC07000398.hg.4	2.04	1.03	2.04	up	8.16	7.13	CCT6P1	chaperonin (	---	---	chr7	65751105	65763675	+
TC07001489.hg.4	2.74	1.45	2.74	up	9.10	7.65	STAG3L1//TI	stromal antig	---	---	chr7	72968116	73005925	-
TC07001513.hg.4	2.31	1.21	2.31	up	8.95	7.74	STAG3L1//S'	stromal antig	---	---	chr7	74697974	74890612	-
TC07003297.hg.4	2.06	1.04	2.06	up	5.61	4.57	NSUN5P1//N	NOP2/Sun d	---	---	chr7	72948293	72954790	-
TC07003331.hg.4	2.95	1.56	2.95	up	10.35	8.79	STAG3L1//S'	stromal antig	---	---	chr7	75359175	75390545	+
TC0800000109.oe.1	2.16	1.11	2.16	up	6.60	5.48	NONHSAG0	NONCODE (	lnc-C8orf49-	NONHSAT14	chr8	11761256	11763223	+
TC0800000111.oe.1	2.06	1.04	2.06	up	7.19	6.14	lnc-NEIL2-2	LNCipedia lo	lnc-NEIL2-2	NONHSAT12	chr8	11802784	11803815	+
TC0800000135.oe.1	-2.10	-1.07	2.10	down	11.21	12.28	lnc-EFHA2-5	NONCODE (	lnc-EFHA2-5	NONHSAT12	chr8	15673763	15764460	+
TC0800000148.oe.1	-2.51	-1.33	2.51	down	7.95	9.28	lnc-ZDHHC2	LNCipedia lo	lnc-ZDHHC2	NONHSAT12	chr8	17223542	17225779	+
TC0800000150.oe.1	-2.10	-1.07	2.10	down	6.50	7.57	lnc-VPS37A-	NONCODE (	lnc-VPS37A-	NONHSAT12	chr8	17295964	17302427	+
TC0800000155.oe.1	-5.66	-2.50	5.66	down	9.04	11.54	lnc-PDGFRL-	LNCipedia lo	lnc-PDGFRL-	NONHSAT12	chr8	17569140	17570516	+
TC0800000192.oe.1	-2.01	-1.01	2.01	down	3.46	4.47	lnc-ATP6V1E	LNCipedia lo	lnc-ATP6V1E	---	chr8	20997906	21013196	+
TC0800000393.oe.1	-2.23	-1.16	2.23	down	3.46	4.62	lnc-EIF4EBP1	NONCODE (	lnc-EIF4EBP1	NONHSAT12	chr8	38062881	38063791	+
TC0800000444.oe.1	2.21	1.15	2.21	up	7.34	6.19	lnc-GOLGA7	NONCODE (	lnc-GOLGA7	NONHSAT12	chr8	41529230	41544722	+
TC0800000484.oe.1	3.41	1.77	3.41	up	5.96	4.19	lnc-LINC002	LNCipedia lo	lnc-LINC002	NONHSAT12	chr8	45928884	45929667	+
TC0800000501.oe.1	2.24	1.17	2.24	up	4.72	3.56	lnc-UBE2V2-	NONCODE (	lnc-UBE2V2-	NONHSAT12	chr8	47960949	47962149	+
TC0800000953.oe.1	-2.05	-1.04	2.05	down	2.59	3.62	lnc-RIMS2-1	LNCipedia lo	lnc-RIMS2-1	---	chr8	1.03E+08	103498670	+
TC0800000970.oe.1	2.17	1.12	2.17	up	4.90	3.79	lnc-ZFPM2-'	LNCipedia lo	lnc-ZFPM2-'	NONHSAT12	chr8	1.07E+08	106742484	+
TC0800001057.oe.1	2.00	1.00	2.00	up	9.75	8.75	lnc-FER1L6-	NONCODE (	lnc-FER1L6-	NONHSAT12	chr8	1.24E+08	123812760	+
TC0800001215.oe.1	2.18	1.12	2.18	up	4.70	3.58	lnc-GML-3	NONCODE (	lnc-GML-3:1	NONHSAT12	chr8	1.43E+08	142965501	+
TC0800001293.oe.1	2.04	1.03	2.04	up	5.64	4.61	lnc-C8orf42-	LNCipedia lo	lnc-C8orf42-	NONHSAT12	chr8	451917	453183	-
TC0800001513.oe.1	-2.16	-1.11	2.16	down	9.06	10.17	lnc-PHYHIP-	NONCODE (	lnc-PHYHIP-	NONHSAT12	chr8	22419549	22422679	-
TC0800001738.oe.1	-2.16	-1.11	2.16	down	8.40	9.52	lnc-RP11-35	LNCipedia lo	lnc-RP11-35	NONHSAT12	chr8	46614286	46617356	-
TC0800001809.oe.1	2.25	1.17	2.25	up	7.14	5.97	lnc-RPS20-4	NONCODE (	lnc-RPS20-4	NONHSAT12	chr8	55738758	55746047	-
TC0800001810.oe.1	2.23	1.16	2.23	up	8.95	7.80	lnc-RPS20-3	LNCipedia lo	lnc-RPS20-3	NONHSAT12	chr8	55750887	55756402	-
TC0800001857.oe.1	2.19	1.13	2.19	up	11.48	10.35	lnc-GGH-4	NONCODE (	lnc-GGH-4:1	NONHSAT12	chr8	61661814	61689715	-
TC0800002000.oe.1	2.24	1.16	2.24	up	4.83	3.67	lnc-MRPS28	LNCipedia lo	lnc-MRPS28	---	chr8	79931927	80034292	-
TC0800002013.oe.1	-2.25	-1.17	2.25	down	4.10	5.26	lnc-PAG1-1:	LNCipedia lo	lnc-PAG1-1:	NONHSAT12	chr8	80628457	80631612	-
TC0800002015.oe.1	2.91	1.54	2.91	up	11.65	10.11	lnc-PAG1-9	NONCODE (	lnc-PAG1-9:	NONHSAT12	chr8	80644939	80645173	-
TC0800002132.oe.1	2.06	1.05	2.06	up	9.56	8.52	lnc-KIAA142	NONCODE (	lnc-KIAA142	NONHSAT12	chr8	94393423	94399583	-
TC0800002229.oe.1	-2.28	-1.19	2.28	down	3.15	4.34	lnc-RRM2B-	NONCODE (	lnc-RRM2B-	NONHSAT12	chr8	1.02E+08	102242619	-
TC0800002454.oe.1	2.02	1.02	2.02	up	10.17	9.16	lnc-FAM49B	NONCODE (	lnc-FAM49B	NONHSAT12	chr8	1.3E+08	130401915	-
TC0800002477.oe.1	-2.97	-1.57	2.97	down	5.41	6.97	lnc-NDRG1-	NONCODE (	lnc-NDRG1-	NONHSAT12	chr8	1.33E+08	133297304	-
TC08000033.hg.4	2.57	1.36	2.57	up	3.81	2.45	DEFB109P1E	defensin, be	---	---	chr8	7312844	7319951	+
TC08000705.hg.4	-4.13	-2.05	4.13	down	3.38	5.42	HAS2-AS1	HAS2 antise	---	---	chr8	1.22E+08	121645324	+

TC08001136.hg.4	2.19	1.13	2.19	up	6.29	5.15	LOC728024	chromosome	---	---	chr8	37746556	37748046	-
TC0900000156.oe.1	-2.36	-1.24	2.36	down	4.53	5.77	lnc-SH3GL2-	LNCipedia lc	lnc-SH3GL2-	NONHSAT13	chr9	17415818	17462984	+
TC0900000261.oe.1	3.05	1.61	3.05	up	13.70	12.09	lnc-SPINK4-	LNCipedia lc	lnc-SPINK4-	NONHSAT13	chr9	33025286	33038726	+
TC0900000325.oe.1	-2.91	-1.54	2.91	down	5.37	6.91	lnc-CA9-1	LNCipedia lc	lnc-CA9-1:1	NONHSAT13	chr9	35680701	35681159	+
TC0900000365.oe.1	-3.60	-1.85	3.60	down	7.67	9.52	lnc-RG9MTD	LNCipedia lc	lnc-RG9MTD	NONHSAT13	chr9	37865026	37867296	+
TC0900000462.oe.1	2.17	1.12	2.17	up	5.66	4.54	lnc-RP11-26	LNCipedia lc	lnc-RP11-26	NONHSAT13	chr9	62817551	62817849	+
TC0900000506.oe.1	-2.18	-1.13	2.18	down	3.01	4.14	lnc-FOXD4L	LNCipedia lc	lnc-FOXD4L	NONHSAT13	chr9	68396524	68406344	+
TC0900000552.oe.1	2.36	1.24	2.36	up	3.76	2.52	lnc-ANXA1-	NONCODE	lnc-ANXA1-	NONHSAT13	chr9	73168098	73170393	+
TC0900000749.oe.1	2.16	1.11	2.16	up	4.67	3.55	lnc-HIATL1-	NONCODE	lnc-HIATL1-	NONHSAT13	chr9	94346664	94347323	+
TC0900000910.oe.1	-2.10	-1.07	2.10	down	6.28	7.35	lnc-FAM206	LNCipedia lc	lnc-FAM206	NONHSAT13	chr9	1.09E+08	109019566	+
TC0900001061.oe.1	-2.44	-1.29	2.44	down	6.80	8.08	lnc-NEK6-2	NONCODE	lnc-NEK6-2:	NONHSAT13	chr9	1.24E+08	124359186	+
TC0900001111.oe.1	2.41	1.27	2.41	up	5.63	4.36	lnc-ZNF79-1	LNCipedia lc	lnc-ZNF79-1	NONHSAT13	chr9	1.27E+08	127457413	+
TC0900001171.oe.1	2.22	1.15	2.22	up	8.80	7.65	lnc-RP11-10	LNCipedia lc	lnc-RP11-10	NONHSAT13	chr9	1.29E+08	128985262	+
TC0900001221.oe.1	2.20	1.14	2.20	up	9.04	7.91	lnc-AIF1L-5	LNCipedia lc	lnc-AIF1L-5:	NONHSAT13	chr9	1.31E+08	131198332	+
TC0900001350.oe.1	2.19	1.13	2.19	up	12.04	10.91	lnc-SLC34A3	NONCODE	lnc-SLC34A3	NONHSAT13	chr9	1.37E+08	137243706	+
TC0900001579.oe.1	2.73	1.45	2.73	up	8.19	6.74	lnc-APTX-3	LNCipedia lc	lnc-APTX-3:	NONHSAT13	chr9	33025350	33039907	-
TC0900001628.oe.1	2.59	1.37	2.59	up	7.15	5.78	lnc-SIT1-1	LNCipedia lc	lnc-SIT1-1:1	NONHSAT13	chr9	35661838	35662644	-
TC09001199.hg.4	2.00	1.00	2.00	up	5.03	4.03	SMC5-AS1	SMC5 antise	---	---	chr9	70193998	70258874	-
TC09001698.hg.4	2.08	1.06	2.08	up	6.65	5.59	RNU6ATAC	RNA, U6atac	---	---	chr9	1.34E+08	134164564	-
TC0X00000041.oe.1	2.10	1.07	2.10	up	6.44	5.37	lnc-STS-5	NONCODE	lnc-STS-5:1	NONHSAT13	chrX	6275635	6276642	+
TC0X00000148.oe.1	-2.63	-1.39	2.63	down	6.43	7.82	GS1-358P8.4	LNCipedia lc	lnc-PDK3-1:	NONHSAT13	chrX	24545516	24550466	+
TC0X00000183.oe.1	2.53	1.34	2.53	up	10.49	9.15	lnc-GK-6	LNCipedia lc	lnc-GK-6:1	NONHSAT13	chrX	30617454	30617693	+
TC0X00000244.oe.1	-2.58	-1.37	2.58	down	4.47	5.84	NONHSAG0	LNCipedia lc	lnc-USP9X-2	NONHSAT13	chrX	41233633	41234283	+
TC0X00000245.oe.1	-2.53	-1.34	2.53	down	5.15	6.49	lnc-USP9X-3	LNCipedia lc	lnc-USP9X-3	NONHSAT13	chrX	41235856	41236579	+
TC0X00000468.oe.1	-2.36	-1.24	2.36	down	10.28	11.52	lnc-STARD8-	NONCODE l	lnc-STARD8-	NONHSAT13	chrX	68533180	68537282	+
TC0X00001172.oe.1	2.00	1.00	2.00	up	10.09	9.09	lnc-RPS6KA3	LNCipedia lc	lnc-RPS6KA3	NONHSAT13	chrX	20136066	20136413	-
TC0X00001260.oe.1	-2.15	-1.10	2.15	down	3.30	4.41	lnc-MED14-	NONCODE	lnc-MED14-	NONHSAT13	chrX	41660101	41665832	-
TC0X00001660.oe.1	2.40	1.26	2.40	up	7.64	6.37	lnc-KCNE1L-	LNCipedia lc	lnc-KCNE1L-	NONHSAT13	chrX	1.1E+08	109733275	-
TC0X00001676.oe.1	2.53	1.34	2.53	up	4.40	3.07	lnc-IL13RA2	LNCipedia lc	lnc-IL13RA2	---	chrX	1.14E+08	114369434	-
TC0X00001858.oe.1	-2.95	-1.56	2.95	down	7.80	9.37	lnc-MAGEA9	LNCipedia lc	lnc-MAGEA9	NONHSAT13	chrX	1.49E+08	149478981	-
TC0X00001918.oe.1	2.25	1.17	2.25	up	8.75	7.58	lnc-DNASE1	NONCODE	lnc-DNASE1	NONHSAT13	chrX	1.54E+08	154365207	-
TC0X00001930.oe.1	2.64	1.40	2.64	up	9.65	8.25	lnc-GAB3-1	LNCipedia lc	lnc-GAB3-1:	NONHSAT13	chrX	1.55E+08	154781596	-
TC0X00001931.oe.1	2.43	1.28	2.43	up	9.68	8.40	lnc-GAB3-2	NONCODE	lnc-GAB3-2:	NONHSAT13	chrX	1.55E+08	154786283	-
TC0X000910.hg.4	3.30	1.72	3.30	up	9.73	8.01	SCARNA9L	small Cajal b	---	---	chrX	20136066	20136413	-
TC0X001084.hg.4	2.06	1.04	2.06	up	3.89	2.85	UQCRBP1	ubiquinol-cy	---	---	chrX	56736788	56737584	-
TC0Y000030.hg.4	3.21	1.68	3.21	up	5.23	3.55	RNU6-941P	RNA, U6 sma	---	---	chrY	7273972	7381548	+
TC1000000014.oe.1	2.02	1.01	2.02	up	4.39	3.38	lnc-GTPBP4-	LNCipedia lc	lnc-GTPBP4-	---	chr10	1080155	1082179	+
TC1000000331.oe.1	2.19	1.13	2.19	up	7.86	6.74	lnc-LYZL1-1	NONCODE	lnc-LYZL1-1	NONHSAT13	chr10	29697903	29698228	+
TC1000000413.oe.1	-2.20	-1.14	2.20	down	3.22	4.36	lnc-ZNF33A-	LNCipedia lc	lnc-ZNF33A	---	chr10	37661694	37672912	+
TC1000000430.oe.1	2.78	1.48	2.78	up	5.88	4.41	lnc-ZNF37A-	NONCODE	lnc-ZNF37A	NONHSAT01	chr10	38501594	38504708	+
TC1000000563.oe.1	2.65	1.41	2.65	up	13.15	11.75	lnc-DKK1-1	LNCipedia lc	lnc-DKK1-1:	NONHSAT01	chr10	52315057	52318042	+
TC1000000607.oe.1	-2.27	-1.18	2.27	down	4.42	5.60	lnc-ARID5B-	NONCODE	lnc-ARID5B-	NONHSAT01	chr10	62374353	62402458	+
TC1000000615.oe.1	2.22	1.15	2.22	up	14.36	13.22	lnc-NRBF2-3	LNCipedia lc	lnc-NRBF2-3	NONHSAT01	chr10	63521409	63610312	+
TC1000000624.oe.1	-2.03	-1.02	2.03	down	3.62	4.64	NONHSAG0	gene_id XLC	lnc-LRRTM3	NONHSAT01	chr10	65313785	65314970	+
TC1000000915.oe.1	2.67	1.42	2.67	up	10.53	9.12	lnc-IFIT5-2	LNCipedia lc	lnc-IFIT5-2:1	NONHSAT01	chr10	89705290	89729207	+
TC1000000937.oe.1	-3.27	-1.71	3.27	down	6.54	8.25	lnc-HECTD2	LNCipedia lc	lnc-HECTD2	NONHSAT01	chr10	91282008	91284258	+
TC1000000952.oe.1	2.49	1.32	2.49	up	5.74	4.42	lnc-O3FAR1	LNCipedia lc	lnc-O3FAR1	NONHSAT01	chr10	93517207	93520277	+
TC1000000970.oe.1	-2.40	-1.26	2.40	down	6.29	7.55	lnc-CYP2C18	LNCipedia lc	lnc-CYP2C18	NONHSAT01	chr10	94612287	94613904	+
TC1000001176.oe.1	-4.35	-2.12	4.35	down	10.85	12.97	lnc-SHOC2-	LNCipedia lc	lnc-SHOC2-	NONHSAT01	chr10	1.11E+08	110898718	+

TC1000001208.oe.1	-2.27	-1.18	2.27	down	8.38	9.57	lnc-CASP7-1	NONCODE (	lnc-CASP7-1	NONHSAT01	chr10	1.14E+08	113570158	+
TC1000001210.oe.1	-4.81	-2.26	4.81	down	7.72	9.99	lnc-AL16240	LNCipedia lc	lnc-AL16240	NONHSAT01	chr10	1.14E+08	113917190	+
TC1000001297.oe.1	2.24	1.17	2.24	up	4.34	3.17	lnc-TACC2-5	NONCODE (	lnc-TACC2-5	NONHSAT01	chr10	1.22E+08	121934456	+
TC1000001618.oe.1	-2.25	-1.17	2.25	down	4.39	5.56	lnc-C1QL3-1	NONCODE (	lnc-C1QL3-1	NONHSAT01	chr10	16512080	16514516	-
TC1000001834.oe.1	-2.54	-1.35	2.54	down	7.78	9.13	lnc-C10orf10	LNCipedia lc	lnc-C10orf10	NONHSAT01	chr10	44376885	44377100	-
TC1000001926.oe.1	-2.27	-1.18	2.27	down	10.74	11.92	lnc-A1CF-4	LNCipedia lc	lnc-A1CF-4:	NONHSAT01	chr10	50800033	50805968	-
TC1000001933.oe.1	-2.24	-1.16	2.24	down	2.85	4.02	lnc-CSTF2T-	LNCipedia lc	lnc-CSTF2T-	---	chr10	52120883	52140539	-
TC1000002024.oe.1	3.29	1.72	3.29	up	6.69	4.97	lnc-RUFY2-1	LNCipedia lc	lnc-RUFY2-1	NONHSAT01	chr10	68416686	68419423	-
TC1000002028.oe.1	-2.41	-1.27	2.41	down	4.47	5.74	lnc-SLC25A1	NONCODE (	lnc-SLC25A1	NONHSAT01	chr10	68691664	68692941	-
TC1000002083.oe.1	-2.20	-1.14	2.20	down	11.91	13.05	lnc-PLA2G12	NONCODE (	lnc-PLA2G12	NONHSAT01	chr10	73009640	73016861	-
TC1000002084.oe.1	3.04	1.61	3.04	up	4.66	3.06	lnc-ECD-3	LNCipedia lc	lnc-ECD-3:1	NONHSAT01	chr10	73069312	73069787	-
TC1000002101.oe.1	2.00	1.00	2.00	up	5.12	4.12	RP11-574K1	LNCipedia lc	lnc-AGAP5-4	NONHSAT01	chr10	73740871	73744372	-
TC1000002225.oe.1	2.05	1.04	2.05	up	5.40	4.36	lnc-GLUD1-	LNCipedia lc	lnc-GLUD1-	---	chr10	87278422	87332633	-
TC1000002413.oe.1	-2.01	-1.01	2.01	down	6.34	7.34	lnc-ARL3-2	NONCODE (	lnc-ARL3-2:1	NONHSAT01	chr10	1.03E+08	102831703	-
TC1000002487.oe.1	2.25	1.17	2.25	up	7.90	6.73	lnc-AFAP1L2	NONCODE (	lnc-AFAP1L2	NONHSAT01	chr10	1.14E+08	114132094	-
TC1000002645.oe.1	2.00	1.00	2.00	up	3.72	2.72	lnc-EBF3-13	LNCipedia lc	lnc-EBF3-13	---	chr10	1.3E+08	129538281	-
TC10000306.hg.4	3.64	1.86	3.64	up	7.28	5.42	FAM35DP	family with s	---	---	chr10	47689700	47731218	+
TC10001298.hg.4	2.43	1.28	2.43	up	5.14	3.86	PRKG1-AS1	PRKG1 antise	---	---	chr10	52230759	52314128	-
TC10001553.hg.4	2.52	1.33	2.52	up	4.12	2.78	LOC1019271	ENTPD1 anti	---	---	chr10	95753206	96090238	-
TC1100000131.oe.1	2.47	1.31	2.47	up	5.40	4.09	lnc-TRIM6-T	NONCODE (	lnc-TRIM6-T	NONHSAT01	chr11	5634838	5666380	+
TC1100000157.oe.1	2.24	1.17	2.24	up	6.96	5.80	lnc-OR2D3-	LNCipedia lc	lnc-OR2D3-	NONHSAT01	chr11	6943190	6956533	+
TC1100000194.oe.1	2.82	1.50	2.82	up	11.86	10.37	lnc-ZNF143-	LNCipedia lc	lnc-ZNF143-	NONHSAT01	chr11	9585183	9588456	+
TC1100000217.oe.1	2.04	1.03	2.04	up	5.05	4.03	lnc-MICAL2-	LNCipedia lc	lnc-MICAL2-	---	chr11	11862185	11862412	+
TC1100000247.oe.1	2.09	1.07	2.09	up	5.16	4.10	lnc-ARNTL-(	LNCipedia lc	lnc-ARNTL-(	---	chr11	13675686	13675918	+
TC1100000248.oe.1	2.70	1.43	2.70	up	11.71	10.27	lnc-ARNTL-7	LNCipedia lc	lnc-ARNTL-7	NONHSAT01	chr11	13710724	13715843	+
TC1100000568.oe.1	-3.59	-1.84	3.59	down	6.84	8.68	lnc-ZNF408-	NONCODE (	lnc-ZNF408-	NONHSAT02	chr11	46723180	46726064	+
TC1100000643.oe.1	-2.05	-1.04	2.05	down	11.11	12.15	lnc-CLP1-2	LNCipedia lc	lnc-CLP1-2:1	NONHSAT02	chr11	57602045	57614453	+
TC1100000817.oe.1	2.66	1.41	2.66	up	8.27	6.86	CMB9-22P13	N/A	---	---	chr11	65455258	65466720	+
TC1100000920.oe.1	2.20	1.13	2.20	up	8.66	7.52	lnc-NDUFV1	NONCODE I	lnc-NDUFV1	NONHSAT02	chr11	67620004	67627393	+
TC1100001171.oe.1	2.34	1.23	2.34	up	10.00	8.78	lnc-PCF11-2	LNCipedia lc	lnc-PCF11-2	NONHSAT02	chr11	83209399	83209634	+
TC1100001262.oe.1	2.96	1.56	2.96	up	11.07	9.51	lnc-C11orf54	LNCipedia lc	lnc-C11orf54	NONHSAT02	chr11	93691681	93695620	+
TC1100001388.oe.1	2.07	1.05	2.07	up	5.06	4.01	lnc-PTS-5	LNCipedia lc	lnc-PTS-5:1	NONHSAT02	chr11	1.12E+08	112218157	+
TC1100001414.oe.1	3.14	1.65	3.14	up	8.18	6.53	lnc-ZBTB16-	LNCipedia lc	lnc-ZBTB16-	NONHSAT02	chr11	1.14E+08	114309729	+
TC1100001438.oe.1	-2.09	-1.06	2.09	down	11.26	12.32	lnc-PAFAH1	LNCipedia lc	lnc-PAFAH1	NONHSAT02	chr11	1.17E+08	116833067	+
TC1100001446.oe.1	-2.15	-1.10	2.15	down	8.27	9.37	lnc-PAFAH1	LNCipedia lc	lnc-PAFAH1	NONHSAT02	chr11	1.17E+08	117182540	+
TC1100001513.oe.1	3.04	1.60	3.04	up	8.14	6.54	lnc-POU2F3	LNCipedia lc	lnc-POU2F3	NONHSAT02	chr11	1.2E+08	120327641	+
TC1100001524.oe.1	-3.26	-1.71	3.26	down	10.21	11.92	lnc-SC5DL-2	LNCipedia lc	lnc-SC5DL-2	NONHSAT02	chr11	1.22E+08	121558773	+
TC1100001525.oe.1	-4.74	-2.24	4.74	down	6.30	8.55	lnc-SC5DL-3	NONCODE (	lnc-SC5DL-3	NONHSAT02	chr11	1.22E+08	121632240	+
TC1100001538.oe.1	-2.48	-1.31	2.48	down	4.29	5.60	lnc-CRTAM-	NONCODE (	lnc-CRTAM-	NONHSAT02	chr11	1.23E+08	122947524	+
TC1100001561.oe.1	2.27	1.18	2.27	up	5.53	4.35	lnc-OR10D3	LNCipedia lc	lnc-OR10D3	---	chr11	1.24E+08	124271078	+
TC1100001786.oe.1	2.05	1.04	2.05	up	11.63	10.59	lnc-RHOG-3	LNCipedia lc	lnc-RHOG-3	NONHSAT01	chr11	3760141	3762960	-
TC1100001975.oe.1	2.21	1.15	2.21	up	5.97	4.83	lnc-SAA2-1	SAA2-SAA4	lnc-SAA2-1:	---	chr11	18231423	18248635	-
TC1100002033.oe.1	2.05	1.03	2.05	up	9.95	8.92	lnc-BDNF-6	LNCipedia lc	lnc-BDNF-6	NONHSAT01	chr11	28084274	28088580	-
TC1100002034.oe.1	2.20	1.14	2.20	up	7.57	6.43	lnc-BDNF-7	NONCODE (	lnc-BDNF-7	NONHSAT01	chr11	28097332	28108145	-
TC1100002103.oe.1	-2.07	-1.05	2.07	down	3.52	4.57	lnc-ELF5-1	LNCipedia lc	lnc-ELF5-1:1	---	chr11	34462056	34468510	-
TC1100002184.oe.1	-2.11	-1.08	2.11	down	10.86	11.94	lnc-PEX16-2	LNCipedia lc	lnc-PEX16-2	NONHSAT02	chr11	45938219	45953615	-
TC1100002333.oe.1	-2.23	-1.16	2.23	down	3.13	4.28	lnc-CPSF7-5	LNCipedia lc	lnc-CPSF7-5	NONHSAT02	chr11	61547167	61556137	-
TC1100002449.oe.1	-2.04	-1.03	2.04	down	4.08	5.11	lnc-LTBP3-8	NONCODE (	lnc-LTBP3-8	NONHSAT02	chr11	65379458	65380989	-
TC1100002591.oe.1	2.13	1.09	2.13	up	5.35	4.26	lnc-NUMA1-	LNCipedia lc	lnc-NUMA1-	---	chr11	71916326	71916808	-

TC1100002673.oe.1	2.07	1.05	2.07	up	4.48	3.44	lnc-WNT11-	LNCipedia l	lnc-WNT11-	NONHSAT0	chr11	75942129	75942435	-
TC1100002764.oe.1	2.13	1.09	2.13	up	4.67	3.58	lnc-AP00097	NONCODE	lnc-AP00097	NONHSAT0	chr11	85757760	85811140	-
TC1100002796.oe.1	-2.14	-1.10	2.14	down	6.61	7.71	lnc-GRM5-1	LNCipedia l	lnc-GRM5-1	NONHSAT0	chr11	89326361	89336083	-
TC1100002797.oe.1	-3.01	-1.59	3.01	down	8.57	10.17	lnc-TRIM49-	NONCODE	lnc-TRIM49-	NONHSAT0	chr11	89443421	89491394	-
TC1100002827.oe.1	-2.89	-1.53	2.89	down	6.86	8.39	lnc-SLC36A	LNCipedia l	lnc-SLC36A	NONHSAT0	chr11	93144175	93146492	-
TC1100002844.oe.1	2.01	1.01	2.01	up	7.65	6.64	lnc-GPR83-	LNCipedia l	lnc-GPR83-	NONHSAT0	chr11	94478761	94493849	-
TC1100002931.oe.1	2.02	1.02	2.02	up	7.22	6.20	lnc-ALKBH8	NONCODE	lnc-ALKBH8	NONHSAT0	chr11	1.07E+08	107353730	-
TC1100003064.oe.1	2.15	1.11	2.15	up	9.57	8.46	lnc-MPZL3-	NONCODE	lnc-MPZL3-	NONHSAT0	chr11	1.18E+08	118257660	-
TC1100003065.oe.1	2.27	1.18	2.27	up	7.98	6.80	lnc-MPZL3-	LNCipedia l	lnc-MPZL3-	NONHSAT0	chr11	1.18E+08	118264386	-
TC1100003141.oe.1	3.39	1.76	3.39	up	17.67	15.91	lnc-CLMP-3	LNCipedia l	lnc-CLMP-3	NONHSAT0	chr11	1.23E+08	123062136	-
TC1100003216.oe.1	2.75	1.46	2.75	up	8.33	6.88	lnc-RP11-8	LNCipedia l	lnc-RP11-8	NONHSAT0	chr11	1.31E+08	130758600	-
TC1200000113.oe.1	2.17	1.12	2.17	up	11.18	10.07	lnc-GAPDH-	LNCipedia l	lnc-GAPDH-	NONHSAT0	chr12	6529260	6530730	+
TC1200000289.oe.1	-2.03	-1.02	2.03	down	3.04	4.06	lnc-MGST1-	LNCipedia l	lnc-MGST1-	---	chr12	16311894	16338471	+
TC1200000323.oe.1	2.04	1.03	2.04	up	4.57	3.54	lnc-PLEKHA	gene_id XLC	lnc-PLEKHA	NONHSAT0	chr12	19671159	19673258	+
TC1200000327.oe.1	-3.97	-1.99	3.97	down	6.16	8.15	lnc-SLCO1C	LNCipedia l	lnc-SLCO1C	NONHSAT0	chr12	20682297	20684072	+
TC1200000331.oe.1	-2.26	-1.18	2.26	down	4.61	5.79	lnc-RP11-12	LNCipedia l	lnc-RP11-12	NONHSAT0	chr12	21131207	21197096	+
TC1200000342.oe.1	-2.23	-1.15	2.23	down	2.91	4.07	lnc-ETNK1-	LNCipedia l	lnc-ETNK1-	---	chr12	22557239	22557503	+
TC1200000370.oe.1	2.14	1.10	2.14	up	4.01	2.91	RP11-295I5.	N/A	---	---	chr12	25225103	25225665	+
TC1200000536.oe.1	-2.09	-1.06	2.09	down	10.09	11.16	lnc-ARID2-1	LNCipedia l	lnc-ARID2-1	NONHSAT0	chr12	46358351	46360617	+
TC1200000641.oe.1	2.04	1.03	2.04	up	4.41	3.39	lnc-ANKRD3	LNCipedia l	lnc-ANKRD3	---	chr12	51751554	51751904	+
TC1200000672.oe.1	2.43	1.28	2.43	up	14.83	13.55	lnc-EIF4B-5	LNCipedia l	lnc-EIF4B-5	NONHSAT0	chr12	52951496	52952278	+
TC1200000707.oe.1	2.04	1.03	2.04	up	5.54	4.51	RP11-834C1	uncharacteri	lnc-HOXC4-	NONHSAT0	chr12	54126071	54132909	+
TC1200000712.oe.1	-2.32	-1.22	2.32	down	2.50	3.71	NONHSAG0	NONCODE	lnc-HNRNP	NONHSAT0	chr12	54262615	54279063	+
TC1200000755.oe.1	-2.03	-1.02	2.03	down	6.89	7.91	lnc-RP11-60	NONCODE	lnc-RP11-60	NONHSAT0	chr12	56094522	56095527	+
TC1200000783.oe.1	-2.26	-1.17	2.26	down	8.22	9.39	lnc-NXPH4-	LNCipedia l	lnc-NXPH4-	NONHSAT0	chr12	57200274	57201153	+
TC1200000920.oe.1	2.07	1.05	2.07	up	12.49	11.44	lnc-SLC35E3	NONCODE	lnc-SLC35E3	NONHSAT0	chr12	68709223	68714378	+
TC1200000954.oe.1	5.03	2.33	5.03	up	6.80	4.47	lnc-THAP2-	NONCODE	lnc-THAP2-	NONHSAT0	chr12	71567262	71573027	+
TC1200001127.oe.1	2.77	1.47	2.77	up	7.92	6.45	lnc-VEZT-2	NONCODE	lnc-VEZT-2:	NONHSAT0	chr12	95308515	95310637	+
TC1200001134.oe.1	2.02	1.02	2.02	up	4.41	3.39	lnc-RP11-53	LNCipedia l	lnc-RP11-53	NONHSAT0	chr12	95865079	95866451	+
TC1200001178.oe.1	-2.08	-1.06	2.08	down	5.83	6.89	lnc-UTP20-	LNCipedia l	lnc-UTP20-	NONHSAT0	chr12	1.01E+08	101086813	+
TC1200001194.oe.1	2.05	1.04	2.05	up	9.60	8.56	lnc-DRAM1-	NONCODE	lnc-DRAM1-	NONHSAT0	chr12	1.02E+08	102197361	+
TC1200001272.oe.1	-2.15	-1.11	2.15	down	6.48	7.58	lnc-UBE3B-1	NONCODE	lnc-UBE3B-1	NONHSAT0	chr12	1.1E+08	109587099	+
TC1200001330.oe.1	-2.06	-1.05	2.06	down	3.34	4.39	lnc-RPH3A-	LNCipedia l	lnc-RPH3A-	---	chr12	1.13E+08	112580012	+
TC1200001653.oe.1	2.14	1.09	2.14	up	7.66	6.56	lnc-PXMP2-	LNCipedia l	lnc-PXMP2-	NONHSAT0	chr12	1.33E+08	132721084	+
TC1200001825.oe.1	-3.65	-1.87	3.65	down	14.92	16.79	lnc-SLC2A1	LNCipedia l	lnc-SLC2A1	NONHSAT0	chr12	7919231	7919750	-
TC1200001827.oe.1	-2.82	-1.49	2.82	down	10.68	12.18	lnc-SLC2A1	NONCODE	lnc-SLC2A1	NONHSAT0	chr12	7932646	7936187	-
TC1200001843.oe.1	2.32	1.21	2.32	up	4.86	3.65	NONHSAG0	A2ML1 antis	lnc-M6PR-3	NONHSAT0	chr12	8776219	8830947	-
TC1200001870.oe.1	3.27	1.71	3.27	up	4.61	2.90	lnc-CLEC2A	NONCODE	lnc-CLEC2A	NONHSAT0	chr12	9856901	9869386	-
TC1200001887.oe.1	2.93	1.55	2.93	up	9.40	7.85	lnc-STYK1-1	LNCipedia l	lnc-STYK1-1	NONHSAT0	chr12	10699317	10704438	-
TC1200001892.oe.1	2.21	1.15	2.21	up	5.08	3.93	lnc-TAS2R4	LNCipedia l	lnc-TAS2R4	NONHSAT0	chr12	11048332	11049256	-
TC1200001905.oe.1	2.02	1.02	2.02	up	9.48	8.47	lnc-AC0076	NONCODE	lnc-AC0076	NONHSAT0	chr12	12477886	12506187	-
TC1200002098.oe.1	2.01	1.01	2.01	up	9.88	8.87	lnc-ABCD2-	NONCODE	lnc-ABCD2-	NONHSAT0	chr12	39333037	39337541	-
TC1200002130.oe.1	-2.25	-1.17	2.25	down	2.97	4.14	NONHSAG0	NONCODE	lnc-NELL2-1	NONHSAT0	chr12	44244394	44263982	-
TC1200002149.oe.1	-2.08	-1.06	2.08	down	13.57	14.63	lnc-SLC38A1	LNCipedia l	lnc-SLC38A1	NONHSAT0	chr12	46187791	46188040	-
TC1200002220.oe.1	2.31	1.21	2.31	up	8.11	6.91	lnc-LMBR1L	LNCipedia l	lnc-LMBR1L	NONHSAT0	chr12	49128981	49131395	-
TC1200002263.oe.1	-2.09	-1.06	2.09	down	3.29	4.36	NONHSAG0	NONCODE	lnc-KRT80-6	NONHSAT0	chr12	51809705	51810600	-
TC1200002339.oe.1	2.11	1.07	2.11	up	8.78	7.70	lnc-GTSF1-2	LNCipedia l	lnc-GTSF1-2	NONHSAT0	chr12	54464697	54473602	-
TC1200002394.oe.1	2.22	1.15	2.22	up	7.03	5.88	lnc-MIP-3	LNCipedia l	lnc-MIP-3:1	NONHSAT0	chr12	56418290	56421090	-
TC1200002413.oe.1	-2.67	-1.42	2.67	down	3.13	4.54	lnc-TMEM1	LNCipedia l	lnc-TMEM1	NONHSAT0	chr12	57096243	57097290	-

TC1200002490.oe.1	-2.29	-1.20	2.29	down	9.83	11.03	lnc-LLPH-3	NONCODE (	lnc-LLPH-3::	NONHSAT02	chr12	66137829	66138937	-
TC1200002547.oe.1	2.23	1.16	2.23	up	7.13	5.97	lnc-TSPAN8	LNCipedia lc	lnc-TSPAN8	NONHSAT02	chr12	71610110	71611286	-
TC1200002586.oe.1	-2.29	-1.20	2.29	down	2.79	3.99	lnc-OSBPL8-	LNCipedia lc	lnc-OSBPL8-	NONHSAT02	chr12	76581285	76581949	-
TC1200002714.oe.1	2.51	1.33	2.51	up	5.24	3.92	lnc-NTN4-2	LNCipedia lc	lnc-NTN4-2	NONHSAT03	chr12	95524520	95529000	-
TC1200002767.oe.1	-2.28	-1.19	2.28	down	3.48	4.67	lnc-ANKS1B	LNCipedia lc	lnc-ANKS1B	NONHSAT03	chr12	1E+08	100054526	-
TC1200002960.oe.1	-2.12	-1.08	2.12	down	7.42	8.51	lnc-C12orf49	NONCODE (	lnc-C12orf49	NONHSAT03	chr12	1.16E+08	115972465	-
TC1200002963.oe.1	2.07	1.05	2.07	up	4.42	3.37	OTTHUMG0	LNCipedia lc	lnc-C12orf49	NONHSAT03	chr12	1.16E+08	116181295	-
TC1200003017.oe.1	2.50	1.32	2.50	up	7.59	6.27	lnc-RAB35-5	LNCipedia lc	lnc-RAB35-5	NONHSAT03	chr12	1.2E+08	119711011	-
TC1200003066.oe.1	-2.19	-1.13	2.19	down	8.75	9.88	lnc-RSRC2-1	NONCODE (	lnc-RSRC2-1	NONHSAT03	chr12	1.22E+08	122483690	-
TC12000540.hg.4	2.11	1.08	2.11	up	5.07	3.99	AGAP2-AS1	AGAP2 antis	---	---	chr12	57726240	57728356	+
TC12001418.hg.4	2.41	1.27	2.41	up	4.50	3.23	RACGAP1P	Rac GTPase	---	---	chr12	45062618	45065411	-
TC1300000023.oe.1	2.65	1.41	2.65	up	8.35	6.94	lnc-MPHOSI	LNCipedia lc	lnc-MPHOSI	NONHSAT03	chr13	19605592	19624904	+
TC1300000168.oe.1	-2.44	-1.28	2.44	down	4.67	5.95	lnc-MTUS2-	LNCipedia lc	lnc-MTUS2-	---	chr13	29509415	29514078	+
TC1300000198.oe.1	2.29	1.20	2.29	up	8.10	6.90	lnc-FRY-3	LNCipedia lc	lnc-FRY-3:1	NONHSAT03	chr13	32396809	32398448	+
TC1300000236.oe.1	2.07	1.05	2.07	up	7.45	6.40	lnc-EXOSC8	NONCODE (	lnc-EXOSC8	NONHSAT03	chr13	37009511	37009732	+
TC1300000266.oe.1	-2.01	-1.01	2.01	down	3.09	4.09	lnc-SLC25A1	LNCipedia lc	lnc-SLC25A1	NONHSAT03	chr13	40298223	40299505	+
TC1300000501.oe.1	-2.02	-1.01	2.02	down	2.36	3.37	LINC01052/	NONCODE (	lnc-AL44598	NONHSAT03	chr13	65866063	65878219	+
TC1300000672.oe.1	2.04	1.03	2.04	up	3.91	2.88	lnc-GPC6-3	LNCipedia lc	lnc-GPC6-3:	---	chr13	91854657	91855926	+
TC1300000704.oe.1	2.02	1.01	2.02	up	7.17	6.16	RP11-128N1	N/A	---	---	chr13	97435946	97436168	+
TC1300000934.oe.1	-2.25	-1.17	2.25	down	3.28	4.45	lnc-ZMYM5-	LNCipedia lc	lnc-ZMYM5-	NONHSAT03	chr13	19899833	19919690	-
TC1300000988.oe.1	2.26	1.18	2.26	up	4.86	3.68	lnc-FAM123	LNCipedia lc	lnc-FAM123	NONHSAT03	chr13	24885290	24886079	-
TC1300001028.oe.1	-4.61	-2.21	4.61	down	4.16	6.37	lnc-SLC46A3	LNCipedia lc	lnc-SLC46A3	NONHSAT03	chr13	28700064	28704557	-
TC1300001125.oe.1	2.13	1.09	2.13	up	14.08	12.99	lnc-STOML3	NONCODE (	lnc-STOML3	NONHSAT03	chr13	39029032	39037294	-
TC1300001179.oe.1	-2.03	-1.02	2.03	down	14.27	15.30	RP11-5G9.6	N/A	---	---	chr13	43894725	43895182	-
TC1300001234.oe.1	2.85	1.51	2.85	up	7.41	5.89	lnc-RCBTB2-	LNCipedia lc	lnc-RCBTB2-	NONHSAT03	chr13	48411741	48412489	-
TC1300001249.oe.1	2.30	1.20	2.30	up	5.37	4.17	lnc-AL13621	LNCipedia lc	lnc-AL13621	NONHSAT03	chr13	49418934	49425841	-
TC1300001389.oe.1	2.10	1.07	2.10	up	9.38	8.31	lnc-DIS3-4	LNCipedia lc	lnc-DIS3-4:1	NONHSAT03	chr13	73093626	73100948	-
TC1300001580.oe.1	-2.48	-1.31	2.48	down	4.19	5.50	lnc-C13orf2	LNCipedia lc	lnc-C13orf2	NONHSAT03	chr13	1.03E+08	102793678	-
TC13000354.hg.4	2.39	1.26	2.39	up	4.33	3.07	FKSG29	FKSG29	---	---	chr13	99351420	99352027	+
TC13000675.hg.4	2.01	1.00	2.01	up	4.51	3.51	GUCY1B2	guanylate cy	---	---	chr13	50994511	51080862	-
TC1400000032.oe.1	2.79	1.48	2.79	up	8.61	7.13	lnc-APEX1-3	LNCipedia lc	lnc-APEX1-3	NONHSAT03	chr14	20476489	20477087	+
TC1400000353.oe.1	-3.12	-1.64	3.12	down	8.01	9.65	lnc-ARF6-3	LNCipedia lc	lnc-ARF6-3::	NONHSAT03	chr14	49894417	49897054	+
TC1400000511.oe.1	-2.36	-1.24	2.36	down	7.10	8.34	lnc-C14orf1	LNCipedia lc	lnc-C14orf1	NONHSAT03	chr14	60296402	60299085	+
TC1400000542.oe.1	2.03	1.02	2.03	up	13.27	12.25	lnc-SYNE2-4	NONCODE (	lnc-SYNE2-4	NONHSAT03	chr14	63601509	63601814	+
TC1400000600.oe.1	-2.23	-1.16	2.23	down	3.50	4.66	RP11-817G1	N/A	---	---	chr14	69154311	69154804	+
TC1400000623.oe.1	-2.19	-1.13	2.19	down	4.51	5.64	lnc-ADAM21	LNCipedia lc	lnc-ADAM21	NONHSAT03	chr14	70366854	70373093	+
TC1400000676.oe.1	-2.03	-1.02	2.03	down	3.57	4.59	lnc-PTGR2-2	LNCipedia lc	lnc-PTGR2-2	---	chr14	73904552	73904850	+
TC1400000847.oe.1	-2.14	-1.10	2.14	down	2.81	3.91	lnc-GOLGA5	LNCipedia lc	lnc-GOLGA5	NONHSAT03	chr14	92926101	92927589	+
TC1400000858.oe.1	2.44	1.29	2.44	up	4.64	3.36	lnc-IFI27-2	LNCipedia lc	lnc-IFI27-2:1	---	chr14	94227390	94249686	+
TC1400000888.oe.1	2.04	1.03	2.04	up	9.10	8.07	lnc-BDKRB1	NONCODE (	lnc-BDKRB1	NONHSAT03	chr14	96223108	96223347	+
TC1400001139.oe.1	2.16	1.11	2.16	up	5.77	4.66	NONHSAG0	LNCipedia lc	lnc-SALL2-1	NONHSAT03	chr14	21507893	21511342	-
TC1400001161.oe.1	2.02	1.01	2.02	up	12.43	11.42	lnc-RBM23-	NONCODE (	lnc-RBM23-	NONHSAT03	chr14	22926481	22929394	-
TC1400001337.oe.1	2.28	1.19	2.28	up	4.03	2.84	lnc-CLEC14A	LNCipedia lc	lnc-CLEC14A	---	chr14	37907394	37960543	-
TC1400001404.oe.1	-2.15	-1.10	2.15	down	4.06	5.17	lnc-NEMF-1	gene_id XLC	lnc-NEMF-1	NONHSAT03	chr14	49861876	49864465	-
TC1400001716.oe.1	-2.69	-1.43	2.69	down	9.72	11.15	lnc-FAM161	LNCipedia lc	lnc-FAM161	NONHSAT03	chr14	73963230	73965055	-
TC1400002034.oe.1	2.72	1.44	2.72	up	15.85	14.41	lnc-MOK-7	NONCODE (	lnc-MOK-7::	NONHSAT04	chr14	1.02E+08	102084798	-
TC1400002035.oe.1	2.28	1.19	2.28	up	4.86	3.67	lnc-MOK-6	LNCipedia lc	lnc-MOK-6::	NONHSAT04	chr14	1.02E+08	102085707	-
TC1500000004.oe.1	2.08	1.05	2.08	up	6.36	5.30	lnc-OR4M2-	NONCODE (	lnc-OR4M2-	NONHSAT04	chr15	20282741	20291586	+
TC1500000071.oe.1	-2.46	-1.30	2.46	down	9.44	10.74	lnc-SNRPN-	NONCODE (	lnc-SNRPN-	NONHSAT04	chr15	24991486	24991753	+

TC1500000072.oe.1	-2.35	-1.23	2.35	down	7.29	8.52	lnc-SNRPN-	NONCODE (	lnc-SNRPN-	NONHSAT04	chr15	24993775	24994849	+
TC1500000233.oe.1	2.05	1.04	2.05	up	3.79	2.75	lnc-C15orf5	NONCODE (	lnc-C15orf5	NONHSAT04	chr15	39588848	39589843	+
TC1500000235.oe.1	2.48	1.31	2.48	up	8.24	6.93	lnc-EIF2AK4	LNCipedia l	lnc-EIF2AK4	NONHSAT04	chr15	39594068	39595551	+
TC1500000247.oe.1	2.18	1.12	2.18	up	11.78	10.65	lnc-PAK6-2	NONCODE (	lnc-PAK6-2:	NONHSAT04	chr15	40200038	40206274	+
TC1500000261.oe.1	2.48	1.31	2.48	up	10.77	9.46	lnc-IVD-2	LNCipedia l	lnc-IVD-2:1	NONHSAT04	chr15	40389808	40392000	+
TC1500000341.oe.1	-2.06	-1.04	2.06	down	8.75	9.79	lnc-CTDSPL2	NONCODE (	lnc-CTDSPL2	NONHSAT04	chr15	44380864	44382229	+
TC1500000367.oe.1	2.57	1.36	2.57	up	4.69	3.32	lnc-C15orf4	LNCipedia l	lnc-C15orf4	NONHSAT04	chr15	45507404	45508017	+
TC1500000464.oe.1	2.19	1.13	2.19	up	5.44	4.31	lnc-UNC13C	LNCipedia l	lnc-UNC13C	---	chr15	54920619	54920916	+
TC1500000514.oe.1	-2.18	-1.13	2.18	down	7.36	8.49	lnc-RNF111-	LNCipedia l	lnc-RNF111-	NONHSAT04	chr15	58855827	58857551	+
TC1500000611.oe.1	2.57	1.36	2.57	up	11.51	10.14	lnc-MAP2K1	LNCipedia l	lnc-MAP2K1	NONHSAT04	chr15	66523511	66528909	+
TC1500000623.oe.1	2.10	1.07	2.10	up	4.21	3.13	lnc-SMAD3-	LNCipedia l	lnc-SMAD3-	NONHSAT04	chr15	67255037	67279451	+
TC1500000841.oe.1	2.97	1.57	2.97	up	6.41	4.84	lnc-MESDC1	LNCipedia l	lnc-MESDC1	NONHSAT04	chr15	80946944	80949162	+
TC1500000999.oe.1	2.35	1.24	2.35	up	8.98	7.75	lnc-CRTC3-7	NONCODE (	lnc-CRTC3-7	NONHSAT04	chr15	90747390	90749960	+
TC1500001114.oe.1	2.09	1.06	2.09	up	5.06	4.00	RP11-35O15	N/A	---	---	chr15	98660210	98660668	+
TC1500001274.oe.1	2.38	1.25	2.38	up	6.17	4.92	NONHSAG0	NONCODE (	lnc-RP11-38	NONHSAT04	chr15	30564940	30625773	-
TC15000013.hg.4	-2.12	-1.08	2.12	down	3.47	4.55	LINC01193	long interge	---	---	chr15	20940438	20993304	+
TC1500001384.oe.1	2.45	1.29	2.45	up	5.12	3.83	lnc-FAM82A	LNCipedia l	lnc-FAM82A	NONHSAT04	chr15	40547432	40548964	-
TC1500001563.oe.1	2.35	1.23	2.35	up	5.57	4.34	CTD-2650P2	N/A	---	---	chr15	52017167	52018032	-
TC1500001611.oe.1	2.44	1.29	2.44	up	7.40	6.11	lnc-MNS1-4	LNCipedia l	lnc-MNS1-4	NONHSAT04	chr15	56421544	56434207	-
TC1500001783.oe.1	2.14	1.10	2.14	up	6.33	5.23	NONHSAG0	NONCODE (	lnc-AAGAB-	NONHSAT04	chr15	67540787	67542604	-
TC1500001825.oe.1	2.86	1.52	2.86	up	6.80	5.28	RP11-138H8	N/A	---	---	chr15	70758269	70758856	-
TC1500001943.oe.1	2.08	1.05	2.08	up	5.49	4.44	lnc-PEAK1.1	LNCipedia l	lnc-PEAK1.1	NONHSAT04	chr15	77069867	77071092	-
TC1500002126.oe.1	-2.36	-1.24	2.36	down	4.77	6.00	OTTHUMG0	LNCipedia l	lnc-IDH2-1:	NONHSAT04	chr15	90074512	90082207	-
TC1500002142.oe.1	2.08	1.05	2.08	up	11.23	10.18	lnc-VPS33B-	LNCipedia l	lnc-VPS33B-	NONHSAT04	chr15	90981510	90994510	-
TC15000030.hg.4	2.72	1.44	2.72	up	5.63	4.19	GOLGA8I	golgin A8 fa	---	---	chr15	22604272	22617853	-
TC15000393.hg.4	3.19	1.67	3.19	up	8.41	6.74	GABPB1-AS1	GABPB1 anti	---	---	chr15	50354174	50367439	+
TC1600000003.oe.1	2.07	1.05	2.07	up	7.58	6.53	lnc-MPG-3	NONCODE (	lnc-MPG-3:	NONHSAT05	chr16	53899	57453	+
TC1600000122.oe.1	-2.09	-1.06	2.09	down	9.20	10.26	lnc-AMDHD	LNCipedia l	lnc-AMDHD	NONHSAT14	chr16	2565553	2571341	+
TC1600000237.oe.1	-2.76	-1.47	2.76	down	6.99	8.45	lnc-PMM2-5	N/A//LNCipe	lnc-PMM2-6	NONHSAT14	chr16	9104848	9113181	+
TC1600000419.oe.1	2.05	1.04	2.05	up	6.31	5.27	lnc-C16orf52	NONCODE (	lnc-C16orf52	NONHSAT14	chr16	22008135	22087534	+
TC1600000447.oe.1	2.49	1.32	2.49	up	6.82	5.50	lnc-DCTN5-	LNCipedia l	lnc-DCTN5-	NONHSAT14	chr16	23687137	23688732	+
TC1600000889.oe.1	2.19	1.13	2.19	up	6.68	5.55	lnc-CETP-1/	NONCODE (	lnc-CETP-1:	NONHSAT14	chr16	56940278	56941726	+
TC1600001069.oe.1	-2.24	-1.16	2.24	down	11.86	13.03	lnc-WWP2-2	LNCipedia l	lnc-WWP2-2	NONHSAT14	chr16	69703065	69704652	+
TC1600001498.oe.1	2.51	1.33	2.51	up	5.55	4.23	lnc-C16orf42	LNCipedia l	lnc-C16orf42	NONHSAT05	chr16	1364174	1366148	-
TC1600001664.oe.1	-2.81	-1.49	2.81	down	3.66	5.15	NONHSAG0	LNCipedia l	lnc-USP7-1:	NONHSAT14	chr16	9105834	9107174	-
TC1600001816.oe.1	4.91	2.30	4.91	up	5.65	3.36	lnc-C16orf8	LNCipedia l	lnc-C16orf8	NONHSAT14	chr16	19858971	19862340	-
TC1600001856.oe.1	-2.06	-1.04	2.06	down	4.50	5.54	lnc-COG7-2	NONCODE (	lnc-COG7-2	NONHSAT14	chr16	23061406	23064173	-
TC1600002366.oe.1	2.22	1.15	2.22	up	5.44	4.29	NONHSAG0	LNCipedia l	lnc-ESRP2-3	NONHSAT14	chr16	68290087	68292790	-
TC1600002447.oe.1	2.20	1.14	2.20	up	4.92	3.78	AC009120.1	N/A	---	---	chr16	74289593	74291052	-
TC1600002455.oe.1	2.44	1.29	2.44	up	9.20	7.92	lnc-MLKL-4	NONCODE (	lnc-MLKL-4:	NONHSAT14	chr16	74643979	74644637	-
TC1600002457.oe.1	2.03	1.02	2.03	up	6.56	5.54	lnc-MLKL-1	LNCipedia l	lnc-MLKL-1:	NONHSAT14	chr16	74671857	74678413	-
TC1600002492.oe.1	-2.58	-1.37	2.58	down	3.23	4.60	RP11-264M1	N/A//NONC	lnc-ADAMTS	NONHSAT14	chr16	77741468	77763555	-
TC1600002518.oe.1	-2.06	-1.04	2.06	down	3.06	4.10	NONHSAG0	NONCODE (	lnc-C16orf6	NONHSAT14	chr16	80627551	80628379	-
TC1700000096.oe.1	-2.06	-1.04	2.06	down	10.74	11.78	lnc-TM4SF5-	NONCODE (	lnc-TM4SF5-	NONHSAT14	chr17	4771884	4783211	+
TC1700000422.oe.1	-2.40	-1.27	2.40	down	3.39	4.65	lnc-DHRS7B	LNCipedia l	lnc-DHRS7B	---	chr17	20878705	20880110	+
TC1700000443.oe.1	2.20	1.14	2.20	up	7.98	6.84	lnc-FAM27L	LNCipedia l	lnc-FAM27L	NONHSAT05	chr17	22331597	22332410	+
TC1700000755.oe.1	2.24	1.16	2.24	up	10.74	9.58	lnc-WIPF2-1	LNCipedia l	lnc-WIPF2-1	NONHSAT05	chr17	40291467	40295506	+
TC1700000826.oe.1	-2.64	-1.40	2.64	down	4.10	5.50	lnc-G6PC-1	LNCipedia l	lnc-G6PC-1:	NONHSAT05	chr17	42914523	42915335	+
TC1700000847.oe.1	2.39	1.26	2.39	up	5.28	4.02	lnc-C17orf1	LNCipedia l	lnc-C17orf1	NONHSAT05	chr17	43680273	43705884	+

TC1700000979.oe.1	2.01	1.01	2.01	up	4.65	3.64	lnc-UBE2Z-2	LNCipedia lc	lnc-UBE2Z-2	NONHSAT0!	chr17	48944950	48945606	+
TC1700001006.oe.1	2.33	1.22	2.33	up	5.14	3.92	lnc-PDK2-1	LNCipedia lc	lnc-PDK2-1:	NONHSAT0!	chr17	50086329	50090481	+
TC1700001037.oe.1	2.18	1.12	2.18	up	10.61	9.49	lnc-ABCC3-!	LNCipedia lc	lnc-ABCC3-!	NONHSAT0!	chr17	50751051	50752550	+
TC1700001070.oe.1	2.13	1.09	2.13	up	6.37	5.27	lnc-TOM1L1	LNCipedia lc	lnc-TOM1L1	---	chr17	55035765	55036638	+
TC1700001073.oe.1	-2.33	-1.22	2.33	down	6.90	8.12	lnc-STXBP4-	LNCipedia lc	lnc-STXBP4-	NONHSAT0!	chr17	55161693	55164296	+
TC1700001074.oe.1	-2.22	-1.15	2.22	down	4.51	5.66	lnc-STXBP4-	N/A//NONC	lnc-STXBP4-	NONHSAT0!	chr17	55167759	55173723	+
TC1700001123.oe.1	2.30	1.20	2.30	up	10.95	9.74	lnc-RAD51C	NONCODE (	lnc-RAD51C	NONHSAT0!	chr17	58709639	58734342	+
TC1700001174.oe.1	-2.25	-1.17	2.25	down	3.46	4.63	lnc-TBX4-2	LNCipedia lc	lnc-TBX4-2:	---	chr17	61761588	61761801	+
TC1700001284.oe.1	-2.94	-1.56	2.94	down	10.80	12.36	lnc-PRKAR1/	LNCipedia lc	lnc-PRKAR1/	NONHSAT0!	chr17	68532540	68532769	+
TC1700001295.oe.1	2.00	1.00	2.00	up	6.32	5.32	lnc-KCNJ16-	LNCipedia lc	lnc-KCNJ16-	NONHSAT0!	chr17	69536066	69542011	+
TC1700001381.oe.1	-2.13	-1.09	2.13	down	4.69	5.78	lnc-C17orf1	LNCipedia lc	lnc-C17orf1	---	chr17	75646566	75647975	+
TC1700001437.oe.1	2.41	1.27	2.41	up	7.07	5.80	lnc-AFMID-1	NONCODE (	lnc-AFMID-1	NONHSAT0!	chr17	78214365	78223856	+
TC1700001746.oe.1	2.01	1.01	2.01	up	8.71	7.70	lnc-KIAA075	NONCODE (	lnc-KIAA075	NONHSAT14	chr17	6649889	6651575	-
TC1700001804.oe.1	2.29	1.19	2.29	up	11.29	10.09	lnc-C17orf5!	LNCipedia lc	lnc-C17orf5!	NONHSAT14	chr17	8206493	8208225	-
TC1700001988.oe.1	-2.14	-1.10	2.14	down	5.13	6.23	RP11-815I9.	NONCODE (	lnc-TBC1D2!	NONHSAT14	chr17	18667629	18669461	-
TC1700002069.oe.1	2.04	1.03	2.04	up	7.34	6.31	lnc-NOS2-1	NONCODE (	lnc-NOS2-1	NONHSAT0!	chr17	27350232	27353832	-
TC1700002471.oe.1	2.14	1.10	2.14	up	5.40	4.30	lnc-CCDC43	LNCipedia lc	lnc-CCDC43	NONHSAT0!	chr17	44673688	44676257	-
TC1700002622.oe.1	-3.62	-1.85	3.62	down	6.13	7.98	lnc-TOB1-4	LNCipedia lc	lnc-TOB1-4:	NONHSAT0!	chr17	50964149	50965494	-
TC1700002679.oe.1	2.31	1.21	2.31	up	6.14	4.93	RP11-159D1	gene_id XLC	lnc-VEZF1-1	NONHSAT0!	chr17	57989039	57994850	-
TC1700002693.oe.1	2.03	1.02	2.03	up	8.60	7.57	lnc-RNF43-1	LNCipedia lc	lnc-RNF43-1	NONHSAT0!	chr17	58417013	58417595	-
TC1700002777.oe.1	-2.37	-1.24	2.37	down	6.39	7.63	lnc-ERN1-1	NONCODE (	lnc-ERN1-1:	NONHSAT0!	chr17	64039150	64041843	-
TC1700002778.oe.1	-2.05	-1.04	2.05	down	5.78	6.81	lnc-ICAM2-3	LNCipedia lc	lnc-ICAM2-3	NONHSAT0!	chr17	64053967	64054767	-
TC1700002812.oe.1	-2.39	-1.26	2.39	down	7.25	8.51	lnc-HELZ-4	LNCipedia lc	lnc-HELZ-4:	NONHSAT0!	chr17	67070438	67077374	-
TC1700002911.oe.1	2.06	1.05	2.06	up	12.22	11.17	lnc-HN1-3	LNCipedia lc	lnc-HN1-3:1	NONHSAT0!	chr17	75147538	75168261	-
TC1700002941.oe.1	-2.26	-1.18	2.26	down	9.03	10.20	lnc-FBF1-3	LNCipedia lc	lnc-FBF1-3:1	NONHSAT0!	chr17	75942879	75943576	-
TC17000352.hg.4	-2.04	-1.03	2.04	down	9.87	10.90	AC005562.1,	leucine rich r	---	---	chr17	30576465	30665698	+
TC1800000040.oe.1	2.89	1.53	2.89	up	11.82	10.29	lnc-EMILIN2	LNCipedia lc	lnc-EMILIN2	NONHSAT0!	chr18	2728414	2739465	+
TC1800000061.oe.1	2.13	1.09	2.13	up	7.41	6.31	lnc-AP00247	LNCipedia lc	lnc-AP00247	NONHSAT0!	chr18	3653410	3656282	+
TC1800000259.oe.1	-2.40	-1.26	2.40	down	4.26	5.52	NONHSAG0	NONCODE (	lnc-CABYR-1	NONHSAT0!	chr18	24114340	24115032	+
TC1800000348.oe.1	2.26	1.18	2.26	up	4.92	3.74	lnc-ZNF397-	LNCipedia lc	lnc-ZNF397-	NONHSAT0!	chr18	35322431	35326981	+
TC1800000495.oe.1	2.67	1.42	2.67	up	8.91	7.50	lnc-C18orf2(	LNCipedia lc	lnc-C18orf2(	NONHSAT0!	chr18	54828481	54888113	+
TC1800000627.oe.1	-2.39	-1.25	2.39	down	8.11	9.36	lnc-DOK6-3	LNCipedia lc	lnc-DOK6-3	NONHSAT0!	chr18	70327517	70330195	+
TC1800000672.oe.1	2.18	1.12	2.18	up	3.75	2.63	lnc-CNDP1-	LNCipedia lc	lnc-CNDP1-	---	chr18	74694422	74739974	+
TC1800000907.oe.1	-2.12	-1.08	2.12	down	4.37	5.45	lnc-POTEC-1	coxsackie vir	lnc-POTEC-1	---	chr18	14477955	14499278	-
TC1800000969.oe.1	-3.22	-1.69	3.22	down	3.71	5.39	RP11-799B1	N/A	---	---	chr18	24135468	24135829	-
TC1800000994.oe.1	-2.03	-1.02	2.03	down	4.25	5.27	RP11-9E17.1	N/A	---	---	chr18	26542971	26545791	-
TC1800001013.oe.1	3.77	1.91	3.77	up	9.29	7.38	lnc-DSC3-2	NONCODE (	lnc-DSC3-2:	NONHSAT0!	chr18	30157571	30175990	-
TC1800001034.oe.1	2.29	1.20	2.29	up	4.36	3.17	lnc-C18orf3	LNCipedia lc	lnc-C18orf3	---	chr18	33516789	33517037	-
TC1800001106.oe.1	2.41	1.27	2.41	up	8.08	6.82	lnc-SYT4-7	LNCipedia lc	lnc-SYT4-7:	NONHSAT0!	chr18	44320551	44321406	-
TC1800001246.oe.1	-2.55	-1.35	2.55	down	5.66	7.01	lnc-PIGN-6	LNCipedia lc	lnc-PIGN-6:	NONHSAT0!	chr18	61808067	61809720	-
TC1800001264.oe.1	-2.64	-1.40	2.64	down	7.65	9.06	lnc-KDSR-1	LNCipedia lc	lnc-KDSR-1:	NONHSAT0!	chr18	63327738	63328319	-
TC1800001294.oe.1	2.32	1.21	2.32	up	4.75	3.54	lnc-CD226-(	LNCipedia lc	lnc-CD226-(	NONHSAT0!	chr18	68697376	68700423	-
TC1800001368.oe.1	-2.08	-1.06	2.08	down	10.07	11.13	lnc-MBP-12	LNCipedia lc	lnc-MBP-12	NONHSAT0!	chr18	76360586	76360989	-
TC18000077.hg.4	2.32	1.22	2.32	up	6.37	5.15	ANKRD20A5	ankyrin repe	---	---	chr18	14179097	14227050	+
TC1900000209.oe.1	4.00	2.00	4.00	up	10.06	8.06	lnc-TRIP10-4	LNCipedia lc	lnc-TRIP10-4	NONHSAT0(	chr19	6680178	6720562	+
TC1900000272.oe.1	2.17	1.12	2.17	up	5.93	4.81	NONHSAG0	NONCODE (	lnc-ZNF559-	NONHSAT0(	chr19	9344784	9345898	+
TC1900000568.oe.1	2.33	1.22	2.33	up	5.78	4.57	lnc-ZNF85-(	LNCipedia lc	lnc-ZNF85-(	NONHSAT0(	chr19	20964835	20965194	+
TC1900000581.oe.1	2.03	1.02	2.03	up	6.31	5.29	lnc-ZNF738-	LNCipedia lc	lnc-ZNF738-	NONHSAT0(	chr19	21411940	21414599	+
TC1900000642.oe.1	3.05	1.61	3.05	up	6.46	4.85	lnc-ZNF726-	NONCODE (	lnc-ZNF726-	NONHSAT0(	chr19	24104186	24107352	+

TC1900000742.oe.1	2.11	1.08	2.11	up	4.19	3.11	lnc-ZNF30-4	LNCipedia lo	lnc-ZNF30-4	---	chr19	34948790	34949061	+
TC1900000760.oe.1	-2.00	-1.00	2.00	down	2.93	3.93	lnc-MAG-1	LNCipedia lo	lnc-MAG-1:1	NONHSAT0(	chr19	35329187	35329928	+
TC1900000817.oe.1	2.02	1.02	2.02	up	6.79	5.78	lnc-ZNF382-	LNCipedia lo	lnc-ZNF382-	---	chr19	36580255	36583605	+
TC1900000844.oe.1	2.14	1.10	2.14	up	6.56	5.47	lnc-ZNF570-	LNCipedia lo	lnc-ZNF570-	NONHSAT0(	chr19	37508265	37543296	+
TC1900001026.oe.1	-2.37	-1.25	2.37	down	8.47	9.72	lnc-APOC4-	NONCODE (	lnc-APOC4-	NONHSAT0(	chr19	44942238	44949565	+
TC1900001146.oe.1	2.20	1.14	2.20	up	9.62	8.48	lnc-LIN7B-2	LNCipedia lo	lnc-LIN7B-2	NONHSAT0(	chr19	49085433	49086443	+
TC1900001604.oe.1	2.14	1.10	2.14	up	7.10	6.01	NONHSAG0	NONCODE (	lnc-C19orf1(	NONHSAT0(	chr19	4654964	4655524	-
TC1900001653.oe.1	8.20	3.04	8.20	up	10.51	7.47	CTD-3128G:	N/A	---	---	chr19	6716386	6717742	-
TC1900001739.oe.1	2.33	1.22	2.33	up	9.76	8.54	lnc-ANGPTL	LNCipedia lo	lnc-ANGPTL	NONHSAT0(	chr19	10116901	10117590	-
TC1900001958.oe.1	2.01	1.01	2.01	up	6.46	5.45	lnc-HOMER3	NONCODE (	lnc-HOMER3	NONHSAT0(	chr19	18990893	18993713	-
TC1900002024.oe.1	2.28	1.19	2.28	up	7.32	6.13	lnc-ZNF43-1	NONCODE (	lnc-ZNF43-1	NONHSAT0(	chr19	21843320	21852030	-
TC1900002040.oe.1	2.02	1.02	2.02	up	5.93	4.91	lnc-ZNF98-7	NONCODE (	lnc-ZNF98-7	NONHSAT0(	chr19	22501699	22501948	-
TC1900002124.oe.1	-2.02	-1.01	2.02	down	7.89	8.91	lnc-CEP89-4	LNCipedia lo	lnc-CEP89-4	NONHSAT0(	chr19	32994022	32996461	-
TC1900002225.oe.1	2.82	1.49	2.82	up	7.75	6.25	lnc-ZNF790-	NONCODE (	lnc-ZNF790-	NONHSAT0(	chr19	36761495	36762617	-
TC1900002398.oe.1	2.00	1.00	2.00	up	4.81	3.81	lnc-ZNF235-	NONCODE (	lnc-ZNF235-	NONHSAT0(	chr19	44140601	44141494	-
TC1900002411.oe.1	-3.36	-1.75	3.36	down	8.89	10.64	lnc-ZNF296-	LNCipedia lo	lnc-ZNF296-	NONHSAT0(	chr19	44907907	44909013	-
TC1900002628.oe.1	-2.24	-1.16	2.24	down	3.52	4.68	NONHSAG0	LNCipedia lo	lnc-ZNF841-	NONHSAT0(	chr19	52058490	52063703	-
TC19000167.hg.4	-2.95	-1.56	2.95	down	3.54	5.10	C3P1	complement	---	---	chr19	10041356	10074137	+
TC19001172.hg.4	2.15	1.10	2.15	up	6.84	5.73	ILF3-AS1	ILF3 antisens	---	---	chr19	10651862	10653872	-
TC19001374.hg.4	-2.14	-1.10	2.14	down	8.33	9.43	HAVCR1P1	hepatitis A v	---	---	chr19	24162193	24163447	-
TC2000000172.oe.1	2.30	1.20	2.30	up	4.46	3.26	lnc-ZNF133-	LNCipedia lo	lnc-ZNF133-	---	chr20	18326151	18329543	+
TC2000000188.oe.1	-2.49	-1.32	2.49	down	4.40	5.72	lnc-NAA20-	LNCipedia lo	lnc-NAA20-	NONHSAT07	chr20	19889410	19935550	+
TC2000000205.oe.1	2.30	1.20	2.30	up	6.33	5.13	RP11-227D2	N/A//NONC	lnc-XRN2-1:	NONHSAT07	chr20	21397818	21400391	+
TC2000000263.oe.1	-2.01	-1.01	2.01	down	3.60	4.61	lnc-GINS1-3	LNCipedia lo	lnc-GINS1-3	NONHSAT07	chr20	25443024	25452628	+
TC2000000268.oe.1	2.07	1.05	2.07	up	6.49	5.44	lnc-GINS1-6	LNCipedia lo	lnc-GINS1-6	---	chr20	25699218	25700761	+
TC2000000316.oe.1	2.09	1.06	2.09	up	8.25	7.18	lnc-DNMT3B	LNCipedia lo	lnc-DNMT3B	NONHSAT07	chr20	32809039	32809357	+
TC2000000633.oe.1	-3.34	-1.74	3.34	down	5.57	7.31	lnc-RAB22A	LNCipedia lo	lnc-RAB22A	NONHSAT08	chr20	58447405	58451094	+
TC2000001022.oe.1	2.18	1.12	2.18	up	7.24	6.12	lnc-ZNF337-	NONCODE (	lnc-ZNF337-	NONHSAT07	chr20	25835985	25836832	-
TC2000001109.oe.1	2.66	1.41	2.66	up	9.03	7.62	lnc-FAM83C	LNCipedia lo	lnc-FAM83C	NONHSAT07	chr20	35345716	35347189	-
TC2000001173.oe.1	-2.03	-1.02	2.03	down	3.20	4.22	lnc-MAFB-7	LNCipedia lo	lnc-MAFB-7	NONHSAT07	chr20	39932922	39933239	-
TC2000001331.oe.1	2.32	1.22	2.32	up	8.61	7.39	lnc-BCAS1-4	LNCipedia lo	lnc-BCAS1-4	NONHSAT08	chr20	54162580	54164548	-
TC20000747.hg.4	-2.06	-1.04	2.06	down	4.02	5.06	MLLT10P1	myeloid/lym	---	---	chr20	30402908	30403462	-
TC2100000015.oe.1	3.84	1.94	3.84	up	10.40	8.46	CH507-513H	N/A	---	---	chr21	8197620	8227646	+
TC2100000016.oe.1	2.05	1.04	2.05	up	9.43	8.39	CH507-528H	N/A	---	---	chr21	8383777	8410645	+
TC2100000032.oe.1	2.87	1.52	2.87	up	4.66	3.13	lnc-POTED-	LNCipedia lo	lnc-POTED-	NONHSAT14	chr21	13065573	13066975	+
TC2100000153.oe.1	-2.15	-1.10	2.15	down	2.58	3.69	lnc-GABPA-	LNCipedia lo	lnc-GABPA-	---	chr21	26935234	26945104	+
TC2100000303.oe.1	-2.07	-1.05	2.07	down	5.62	6.67	lnc-DSCR8-2	NONCODE (	lnc-DSCR8-2	NONHSAT08	chr21	38121451	38188016	+
TC2100000383.oe.1	2.14	1.10	2.14	up	4.21	3.11	lnc-CRYAA-	NONCODE (	lnc-CRYAA-	NONHSAT08	chr21	43111995	43112200	+
TC2100000465.oe.1	2.46	1.30	2.46	up	9.12	7.82	lnc-PCBP3-5	LNCipedia lo	lnc-PCBP3-5	NONHSAT08	chr21	45483805	45484746	+
TC2100000490.oe.1	2.38	1.25	2.38	up	6.38	5.13	lnc-PCNT-2	NONCODE l	lnc-PCNT-2:	NONHSAT08	chr21	46292765	46294797	+
TC2100000566.oe.1	2.27	1.18	2.27	up	4.95	3.77	lnc-AF16513	LNCipedia lo	lnc-AF16513	NONHSAT08	chr21	14979232	14990989	-
TC2100000588.oe.1	2.19	1.13	2.19	up	4.52	3.39	lnc-BTG3-6	LNCipedia lo	lnc-BTG3-6:	NONHSAT08	chr21	17500477	17512529	-
TC2100000815.oe.1	2.23	1.16	2.23	up	4.17	3.01	lnc-KCNJ6-2	LNCipedia lo	lnc-KCNJ6-2	---	chr21	37928141	37932822	-
TC21000116.hg.4	3.42	1.77	3.42	up	5.76	3.98	C21orf119	chromosome	---	---	chr21	32393130	32393960	+
TC2200000359.oe.1	2.49	1.31	2.49	up	13.97	12.66	lnc-AC0050(	LNCipedia lo	lnc-AC0050(	NONHSAT08	chr22	31399633	31411060	+
TC2200000420.oe.1	2.81	1.49	2.81	up	7.15	5.65	lnc-MCM5-2	LNCipedia lo	lnc-MCM5-2	NONHSAT08	chr22	35381143	35386850	+
TC2200000424.oe.1	2.29	1.19	2.29	up	5.87	4.68	lnc-APOL6-2	LNCipedia lo	lnc-APOL6-2	NONHSAT08	chr22	35666257	35668386	+
TC2200000580.oe.1	3.01	1.59	3.01	up	8.94	7.35	lnc-SERHL2-	NONCODE (	lnc-SERHL2-	NONHSAT08	chr22	42614914	42616588	+
TC2200000645.oe.1	-2.46	-1.30	2.46	down	7.20	8.50	lnc-PPARA-4	LNCipedia lo	lnc-PPARA-4	NONHSAT08	chr22	46242515	46243756	+

TC2200000772.oe.1	-3.28	-1.71	3.28	down	4.17	5.88	lnc-AC0081(	LNCipedia lc	lnc-AC0081(	---	chr22	18729353	18733601	-
TC2200000958.oe.1	2.09	1.06	2.09	up	5.61	4.54	lnc-TTC28-1	LNCipedia lc	lnc-TTC28-1	NONHSAT0{	chr22	28687749	28689410	-
