# Supplementary material for: Differentially Expressed Long Noncoding RNAs Involved in FUBP1 Promoting Hepatocellular Carcinoma Cells Proliferation
Source: Biomed Res Int. 2021 Apr 14;2021:6664519. doi: 10.1155/2021/6664519 (PMC8063849; doi:10.1155/2021/6664519)
Supplement: Supplementary 4 — Pearson's test result on lncRNAs-mRNA coexpression network analysis. [file 6664519.f4.pdf]

Pvalue	correlation	Probe Set ID	p	FC (abs)	Regulati	p	FC (abs)	Regulation	GeneSymb	Probe Set ID	p	FC (abs)	Regulation	p	FC (abs)	Regulatic
0.05	0.82	TC01002803.hg.4	0.01	8.12	up	0.01	8.12	up	FUBP1	TC01000042	0.01	2.25	up	0.01	2.25	up
0.02	-0.89	TC01002803.hg.4	0.01	8.12	up	0.01	8.12	up	FUBP1	TC02000044	0.03	2.06	down	0.03	2.06	down
0.04	-0.84	TC01002803.hg.4	0.01	8.12	up	0.01	8.12	up	FUBP1	TC0X000002	0.01	2.11	down	0.01	2.11	down
0.00	0.96	TC01002803.hg.4	0.01	8.12	up	0.01	8.12	up	FUBP1	TC12000009	0.01	2.22	up	0.01	2.22	up
0.04	-0.83	TC01002803.hg.4	0.01	8.12	up	0.01	8.12	up	FUBP1	TC14000005	0.03	2.34	down	0.03	2.34	down
0.03	-0.86	TC02000705.hg.4	0.03	2.07	down	0.03	2.07	down	ZC3H6//AC	TC01000042	0.01	2.25	up	0.01	2.25	up
0.02	0.87	TC02000705.hg.4	0.03	2.07	down	0.03	2.07	down	ZC3H6//AC	TC02000044	0.03	2.06	down	0.03	2.06	down
0.04	0.82	TC02000705.hg.4	0.03	2.07	down	0.03	2.07	down	ZC3H6//AC	TC14000003	0.02	2.51	down	0.02	2.51	down
0.00	-0.96	TC03000434.hg.4	0.03	2.21	down	0.03	2.21	down	LOC101927	TC01000042	0.01	2.25	up	0.01	2.25	up
0.01	0.91	TC04000437.hg.4	0.00	2.70	up	0.00	2.70	up	ANXA3	TC01000042	0.01	2.25	up	0.01	2.25	up
0.01	-0.94	TC04000516.hg.4	0.02	2.42	down	0.02	2.42	down	MTTP	TC10000015	0.02	2.00	down	0.02	2.00	down
0.03	0.87	TC04000859.hg.4	0.04	2.01	down	0.04	2.01	down	CEP44	TC02000044	0.03	2.06	down	0.03	2.06	down
0.03	0.85	TC04000859.hg.4	0.04	2.01	down	0.04	2.01	down	CEP44	TC05000013	0.03	2.08	down	0.03	2.08	down
0.00	0.97	TC04000859.hg.4	0.04	2.01	down	0.04	2.01	down	CEP44	TC0X000002	0.01	2.11	down	0.01	2.11	down
0.02	0.88	TC04000859.hg.4	0.04	2.01	down	0.04	2.01	down	CEP44	TC11000028	0.04	2.11	down	0.04	2.11	down
0.04	0.83	TC04000859.hg.4	0.04	2.01	down	0.04	2.01	down	CEP44	TC14000003	0.02	2.51	down	0.02	2.51	down
0.00	0.95	TC04000859.hg.4	0.04	2.01	down	0.04	2.01	down	CEP44	TC14000005	0.03	2.34	down	0.03	2.34	down
0.01	0.91	TC05001596.hg.4	0.00	2.13	down	0.00	2.13	down	NR2F1-AS1	TC10000015	0.02	2.00	down	0.02	2.00	down
0.00	0.97	TC05001596.hg.4	0.00	2.13	down	0.00	2.13	down	NR2F1-AS1	TC14000003	0.02	2.51	down	0.02	2.51	down
0.01	0.93	TC05001973.hg.4	0.01	2.50	down	0.01	2.50	down	FAXDC2	TC01000021	0.01	2.13	down	0.01	2.13	down
0.02	0.88	TC05001973.hg.4	0.01	2.50	down	0.01	2.50	down	FAXDC2	TC0X000001	0.04	2.01	down	0.04	2.01	down
0.05	-0.81	TC05001973.hg.4	0.01	2.50	down	0.01	2.50	down	FAXDC2	TC12000009	0.01	2.22	up	0.01	2.22	up
0.04	-0.84	TC05001973.hg.4	0.01	2.50	down	0.01	2.50	down	FAXDC2	TC15000002	0.01	2.59	up	0.01	2.59	up
0.03	-0.86	TC07001758.hg.4	0.02	2.88	down	0.02	2.88	down	IMMP2L	TC01000042	0.01	2.25	up	0.01	2.25	up
0.00	0.95	TC0X001416.hg.4	0.02	2.28	down	0.02	2.28	down	ARHGEF6	TC10000015	0.02	2.00	down	0.02	2.00	down
0.01	0.93	TC0X001416.hg.4	0.02	2.28	down	0.02	2.28	down	ARHGEF6	TC14000003	0.02	2.51	down	0.02	2.51	down
0.02	0.88	TC10000798.hg.4	0.00	2.03	down	0.00	2.03	down	MXI1	TC0X000001	0.04	2.01	down	0.04	2.01	down
0.02	-0.87	TC10000798.hg.4	0.00	2.03	down	0.00	2.03	down	MXI1	TC10000015	0.02	2.00	down	0.02	2.00	down
0.00	-0.96	TC10000798.hg.4	0.00	2.03	down	0.00	2.03	down	MXI1	TC15000002	0.01	2.59	up	0.01	2.59	up
0.00	0.94	TC12000779.hg.4	0.03	2.20	down	0.03	2.20	down	GAS2L3	TC01000021	0.01	2.13	down	0.01	2.13	down
0.04	0.82	TC12000779.hg.4	0.03	2.20	down	0.03	2.20	down	GAS2L3	TC05000013	0.03	2.08	down	0.03	2.08	down
0.00	0.98	TC12000779.hg.4	0.03	2.20	down	0.03	2.20	down	GAS2L3	TC0X000001	0.04	2.01	down	0.04	2.01	down
0.00	-0.96	TC12000779.hg.4	0.03	2.20	down	0.03	2.20	down	GAS2L3	TC15000002	0.01	2.59	up	0.01	2.59	up
0.01	0.93	TC14002295.hg.4	0.05	2.11	down	0.05	2.11	down	MIA2	TC01000021	0.01	2.13	down	0.01	2.13	down
0.05	0.81	TC14002295.hg.4	0.05	2.11	down	0.05	2.11	down	MIA2	TC05000013	0.03	2.08	down	0.03	2.08	down
0.02	-0.89	TC14002295.hg.4	0.05	2.11	down	0.05	2.11	down	MIA2	TC12000009	0.01	2.22	up	0.01	2.22	up
0.05	-0.82	TC15000270.hg.4	0.04	2.33	up	0.04	2.33	up	THBS1	TC01000021	0.01	2.13	down	0.01	2.13	down
0.00	-0.98	TC15000270.hg.4	0.04	2.33	up	0.04	2.33	up	THBS1	TC0X000001	0.04	2.01	down	0.04	2.01	down
0.00	1.00	TC15000270.hg.4	0.04	2.33	up	0.04	2.33	up	THBS1	TC15000002	0.01	2.59	up	0.01	2.59	up
0.00	-0.95	TC17001786.hg.4	0.03	2.11	up	0.03	2.11	up	FTSJ3	TC01000021	0.01	2.13	down	0.01	2.13	down
0.04	-0.83	TC17001786.hg.4	0.03	2.11	up	0.03	2.11	up	FTSJ3	TC02000044	0.03	2.06	down	0.03	2.06	down
0.01	-0.93	TC17001786.hg.4	0.03	2.11	up	0.03	2.11	up	FTSJ3	TC05000013	0.03	2.08	down	0.03	2.08	down
0.01	-0.90	TC17001786.hg.4	0.03	2.11	up	0.03	2.11	up	FTSJ3	TC0X000002	0.01	2.11	down	0.01	2.11	down
0.02	-0.88	TC17001786.hg.4	0.03	2.11	up	0.03	2.11	up	FTSJ3	TC11000028	0.04	2.11	down	0.04	2.11	down
0.01	0.92	TC17001786.hg.4	0.03	2.11	up	0.03	2.11	up	FTSJ3	TC12000009	0.01	2.22	up	0.01	2.22	up
0.01	-0.92	TC17001786.hg.4	0.03	2.11	up	0.03	2.11	up	FTSJ3	TC14000005	0.03	2.34	down	0.03	2.34	down
